# Causality of genetically determined serum metabolites on lower back pain or/and sciatica: a comprehensive Mendelian randomized study

**DOI:** 10.3389/fpain.2024.1370704

**Published:** 2024-09-25

**Authors:** Yi-Ming Ren, Wei-Yu Hou, Bao-You Fan, Yuan-Hui Duan, Yun-Bo Sun, Tao Yang, Han-Ji Zhang, Tian-Wei Sun, Meng-Qiang Tian

**Affiliations:** ^1^ Department of Orthopaedics, International Science and Technology Cooperation Base of Spinal Cord Injury, Tianjin Key Laboratory of Spine and Spinal Cord Injury, Tianjin Medical University General Hospital, Tianjin, China; ^2^ Department of Joint and Sport Medicine, Tianjin Union Medical Center, Nankai University Affiliated People’s Hospital, Tianjin, China; ^3^ Department of Spine Surgery, Tianjin Union Medical Center, Nankai University Affiliated People’s Hospital, Tianjin, China

**Keywords:** lower back pain, sciatica, Mendelian randomization, serum metabolites, single nucleotide polymorphisms

## Abstract

**Background:**

There is an urgent need to confirm biomarkers reflecting the pathogenesis and targeted drugs of lower back pain or/and sciatica in clinical practice. This study aimed to conduct a two sample bidirectional Mendelian randomization (MR) analysis to explore the causal link between 486 serum metabolites and lower back pain or/and sciatica.

**Methods:**

All data come from two public shared databases of European ancestry and single nucleotide polymorphisms (SNPs) for lower back pain or/and sciatica acted as instrumental variables. The traditional inverse variance weighting (IVW) method, weighted-median method, MR-Egger methodand other methods were used to estimate causality. The horizontal pleiotropy, heterogeneities were also verified through the MR-Egger intercept test, Cochran's Q test, MR-PRESSO test and the leave-one-out sensitivity analysis. Reverse MR analysis was employed to evaluate the direct impact of metabolites on lower back pain or/and sciatica. Additionally, we conducted the colocalization analysis to reflect the causality deeply. Furthermore, metabolic pathway analysis was performed.

**Results:**

28 metabolites (18 known metabolites, 1 identified metabolites and 9 unknown metabolites) relevant to the risk of sciatica or/and lower back pain after using genetic variants as probes at P_IVW_ < 0.05 were identifed. Among them, 8 serum metabolites decreased risk of sciatica or/and lower back pain significantly (*P* < 0.05), and 14 serum metabolites increased risk of sciatica or/and lower back pain significantly (*P* < 0.05). No reverse causal association was found between 28 metabolites and sciatica or/and lower back pain. Colocalization analysis results showed that the associations between sciatica or/and lower back pain and the 28 identified metabolites were not due to shared causal variant sites. Moreover, pathway enrichment analysis identifed 11 signifcant metabolic pathways, which are mainly involved in the pathological mechanism of sciatica or/and lower back pain (*P* < 0.05). There was no horizontal pleiotropy or heterogeneity in the other analyses.

**Conclusion:**

Our analyses provided robust evidence of causal associations between blood metabolites on sciatica or/and lower back pain. However, the underlying mechanisms remain to be further investigated.

## Introduction

Sciatica or/and lower back pain is not a medical diagnosis, but rather a symptom secondary to degenerative diseases of the lumbar spine such as disc herniation and spinal stenosis. In clinical practice, the vast majority of sciatica is related to nerve root compression caused by intervertebral disc herniation (IVDD), but mechanical compression alone cannot explain all phenomena. Sciatica, as a secondary symptom, involves complex processes such as decreased muscle strength, sensory loss and numbness, decreased reflexes, and abnormal sensations. It is currently believed that the inflammatory response between the nucleus pulposus and nerve roots plays an important role in discogenic sciatica or/and lower back pain (1, 2). Cytokines are a type of glycoprotein molecules produced by various cells and secreted into the extracellular space, possessing various biological functions. The role of cytokine mediated neuroimmune responses in sciatica has been widely studied (3, 4), pro-inflammatory cytokines can induce pain related behaviors (5), and anti-inflammatory cytokines can effectively alleviate hyperalgesia (6). However, it is unknown whether the differences in cytokine profiles are related to sciatica and pain intensity, and there is an urgent need to confirm biomarkers reflecting the intensity of sciatica in clinical practice.

However, with the development of metabolomics, the idea of identifying specific small molecule biomarkers in the serum of patients with sciatica or/and lower back pain and identifying potential causes has become possible (7, 8). Metabolomics is the systematic study of metabolic profiles in biological samples (cells, tissues, and body fluids). Usually, the low molecular weight of metabolites (<1,500 Da), which represent intermediate and/or final products of cell metabolism and cell cycle, may have different functions in different organisms. Metabolomics is an effective method for revealing the phenotype of biological molecules. This method can identify changes in small molecule metabolites in various diseases, which is of great help in understanding and diagnosing diseases. Several recent metabolic studies have documented numerous circulating markers, such as amino acids, sugars, and fats, in both human and animal models (9–11). However, due to restrictions in sample size and the presence of confounding factors, the direct impact of blood markers on sciatica or/and lower back pain remains unverified.

Mendelian randomization (MR) is a form of instrumental variable (IV) analysis that employs genetic variations as an IV to identify and measure causality (12). Due to its ability to mitigate the impact of potential confounding variables and reverse causality, MR has gained popularity in observational research in recent times. Moreover, non-experimental investigations frequently incorporate possible variables that may distort the results and the possibility of cause and effect being mistakenly identified (13–15). Thus, the objective of this study was to elucidate the genetic connection and potential causative link between 486 human serum metabolites and risks of sciatica or/and lower back pain through a bidirectional MR validation. Genome-wide association studies (GWAS) can uncover numerous genetic links, yet pinpointing causal variants as opposed to mere correlations continues to be a major hurdle. To refine these results, colocalization analysis provides evidence to ascertain if a single causal variant can affect multiple traits. Therefore, we carried out GWAS-GWAS colocalization analysis to detect and reduce false positives. This was accomplished by comparing gene loci across various GWAS, thereby ensuring a more precise identification of true genetic associations.

## Methods

Our study is a Mendelian randomization study and we have conformed to the STROBE guidelines (see STROBE-MR-checklist in Supplementary Table S1). All published GWAS received ethical approval from the appropriate institutional review boards. This study utilized only summary-level data, eliminating the need for additional ethical approval.

### Study design

This study used two sample bidirectional Mendelian randomization method to explore the causal relationship between 486 human serum metabolites and risks of sciatica or/and lower back pain. First, the association data of human serum metabolites (exposure factor) were obtained from a GWAS, so as to clarify the single nucleotide polymorphism (SNP) site associated with human serum metabolites, and treat it as gene instrumental variables estimation. Subsequently, another GWAS study was conducted to obtain association data with sciatica or/and lower back pain (outcome) and clarify the existence of relevant SNPs; Finally, through the screened SNPs and various statistical methods, we can comprehensively judge the causal relationship between 486 human serum metabolites and risks of sciatica or/and lower back pain.

#### Data sources

We obtained summary association data from the most extensive genetic investigation into human metabolism, which was openly accessible on the Metabolomics GWAS Server (website: http://metabolomics.helmholtz-muenchen.de/gwas/) (16). Utilizing a dataset comprising 7,824 individuals from two European population cohorts, a comprehensive set of 486 metabolites was measured using the MS (Metabolon) platform. Subsequently, GWAS analyses were conducted utilizing the genotype dataset imputed based on HapMap2. In the current study, we included 177 metabolites whose chemical identity had not been definitively determined, along with 309 confirmed metabolites. Additionally, these 309 known metabolites were further categorized into eight biochemical classes (peptides, energy, nucleotides, lipids, amino acids, cofactors and vitamins, carbohydrates, and xenobiotics) based on the Kyoto Encyclopedia of Genes and Genomes (KEGG) database. It is worth mentioning that comprehensive genotyping information for the two cohorts has been extensively described in previous research (17). Finally, the GWAS meta-analysis encompassed approximately 2.1 million SNPs. Data related to sciatica or/and lower back pain is derived from the GWAS dataset (https://gwas.mrcieu.ac.uk/datasets/finn-b-M13_LOWBACKPAINORANDSCIATICA/). The sample size of this dataset is 218,792 (ncase = 19,509, ncontrol = 199,283), including Europeans with a SNP count of 16,380,466.

#### Genetic variants

**Figure 1:**
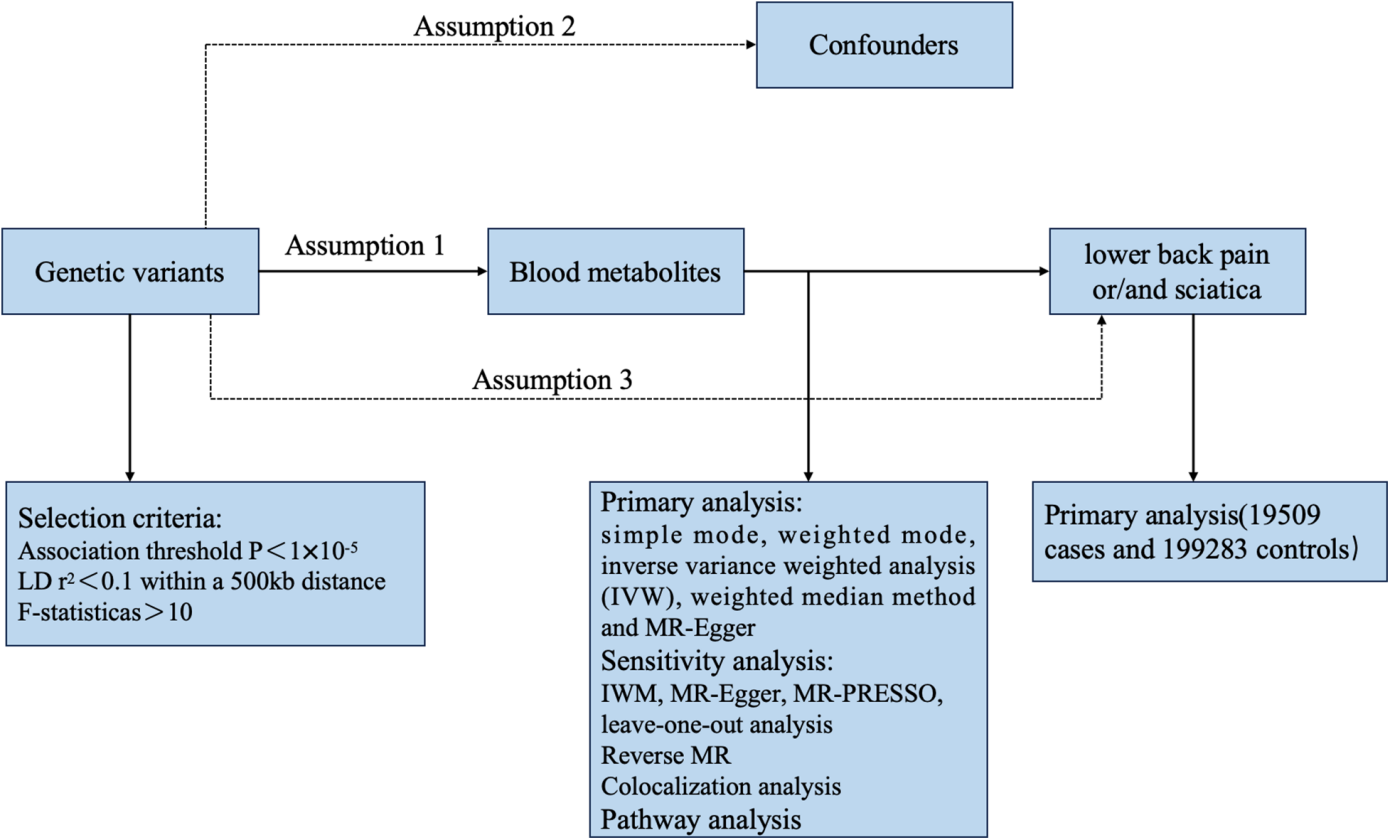
The overview of the research workflow.

Based on the requirements of MR analysis, genetic variants as instrumental variables (IVs) need to fulfill the following three fundamental assumptions: (I) a robust correlation exists between instrumental variables and the exposure factor X; (II) instrumental variables are not linked to any confounding factors affecting the association between exposure and outcome; and (III) instrumental variables do not influence outcome Y, except for a possible indirect impact through their association with exposure X (18–20). Figure 1 depicts the fundamental assumptions and workflow of Mendelian randomization (MR). To identify instrumental variables (IVs) for the 486 metabolites, certain procedures were undertaken to ensure the validity of the first assumption. Initially, genetic variants were extracted using association thresholds of *P* < 1 × 10^–5^, which are commonly employed in MR analysis to capture greater variability when limited SNPs are available for exposure. Subsequently, independent variants were identified through a clumping procedure implemented in R software, with a linkage-disequilibrium threshold of *r*^2^ < 0.01 within a 500 kb window based on the European 1,000 Genomes Project Phase 3 reference panel. Instrumental SNPs were selected by excluding palindromic SNPs with a middle allele frequency (MAF). MR Steiger filters were used to exclude SNPs with an incorrect causal direction. Finally, in order to assess the strength of the selected instruments quantitatively, we calculated the explained variance (R2) and the F statistic for each metabolite. Typically, a threshold of F > 10 is recommended for further MR analysis.

#### MR statistical analyses

In this study, five statistical methods, simple mode, weighted mode, inverse varianceweighted analysis (IVW), weighted median method and MR-Egger regression, were used to analyze the causal association between human serum metabolites and risks of sciatica or/and lower back pain. As the most well-established research approach in Mendelian randomization, the IVW analysis method (utilizing a random effects model when heterogeneity exists and a fixed effects model when there is no heterogeneity) conducts weighted analysis on genetic variants that satisfy the criteria of valid instrumental variables. The weighted midpoint approach necessitates that a minimum of 50% of SNPs fulfill the requirement of being effective instrumental variables. Once the SNPs are arranged in order of their weights, the analysis outcome is derived from the median of the associated distribution function. MR-Egger regression analysis can still gauge the causal impact of the outcome on the condition that the incorporated SNPs exhibit gene pleiotropy. The slope of the regression is the causal effect estimate of human serum metabolites on the trend of sciatica or/and lower back pain. However, due to its low test efficiency and wide confidence interval results, MR-Egger analysis is often used as a sensitivity analysis for other statistical results. MR-PRESSO is a different innovative MR technique that has the capability to identify outliers and adjust for horizontal pleiotropy, thus yielding an accurate estimation (15). All outcomes are presented as odds ratios (OR) along with their corresponding 95% confidence intervals (CI), and a *p*-value of less than 0.05 is deemed statistically significant. The statistical analysis, employing the Two Sample MR 0.5.6 and MR-PRESSO packages, was conducted using R software version 3.6.0. The leave-one-out method was employed to assess the sensitivity of the Mendelian randomization findings. After sequentially removing the incorporated SNPs, the IVW technique was employed to compute the effect of analysis on the remaining all SNPs, and the impact of an individual SNP on the estimation of causal judgment was determined accordingly. The asymmetry plot from the funnel plot was utilized to identify any heterogeneity, and a symmetric plot confirmed the lack of heterogeneity. An additional indication that horizontal multiplicity is absent can be observed through the symmetry of the funnel plot. Additionally, the false positive rate rose because of the numerous exposure factors and various exposure phenotypes in GWAS originating from the same sample set. To mitigate this, we utilized the false discovery rate (FDR < 0.05) to adjust the MR results.

#### Reverse MR analysis

A reverse MR analysis was performed to evaluate if sciatica or/and lower back pain influences the levels of candidate serum metabolites. The MR analysis was conducted following the previously described methodology.

#### Colocalization analysis

We performed a colocalization analysis using the coloc R package to investigate whether the association between the identified metabolites and sciatica or/and lower back pain was due to a shared causal variant (21). This involved analyzing regional loci within 1,000 kb on either side of the lead SNP in the exposure data, thereby reducing the likelihood of reinforcing false associations between the two phenotypes. Within a Bayesian framework, the coloc method assessed posterior probabilities for five hypotheses (H0, H1, H2, H3, H4) at each variant locus: (1) no association with either trait; (2) association with only trait 1; (3) association with only trait 2; (4) associations with both traits due to different causal variants; and (5) both traits sharing a common causal variant (22). We used default priors (p1 = 1 × 10^–4^, p2 = 1 × 10^–4^, p12 = 1 × 10^–5^) for the colocalization analysis. The presence of a posterior probability over 80% for H4 (PP4) under various prior and window conditions was considered strong evidence of colocalization.

#### Metabolic pathway analysis

To investigate the roles of the identified metabolites, MetaboAnalyst 5.0 (https://www.metaboanalyst.ca/) (23) was employed for conducting metabolic pathway analysis. In order to obtain extensive and reliable pathway analysis outcomes, all metabolites significantly associated with sciatica or/and lower back pain (with a *P*-value < 0.05) were included. For this purpose, two databases, namely the Small Molecule Pathway database (SMPDB) and the KEGG database, were utilized. The significance level for the pathway analysis was set at 0.10.

## Results

### Influence of 486 serum metabolites on sciatica or/and lower back pain (forward MR)

#### Determination of IVs

We conducted a two-sample MR analysis to assess the causal relationship between genetically determined metabolites and sciatica or/and lower back pain using four distinct pairs of GWAS summary data. The instrumental variables (IVs) generated for the 480 metabolites ranged from 3 to 481 SNPs, with fructose having the fewest IVs (3 SNPs), and 2-methoxyacetaminophen sulfate* having the most IVs (481 SNPs). Furthermore, all IVs were adequately effective for conducting MR analysis on the 480 metabolites (F-statistic >10) (see Supplementary Table S2).

#### Association between serum metabolites and sciatica or/and lower back pain

The IVW approach was utilized to validate the causal relationship between the 480 metabolites and sciatica or/and lower back pain. In total, 28 distinct metabolites were confirmed with a P_IVW_ value of less than 0.05 and FDR < 0.05, out of which 18 were previously identified metabolites (tyrosine, malate, pentadecanoate (15:0), benzoate, aspartate, 1,5-anhydroglucitol (1,5-AG), 1-palmitoylglycerol (1-monopalmitin), levulinate (4-oxovalerate), glycine, 3-methylxanthine, C-glycosyltryptophan*, adrenate (22:4n6), alpha-hydroxyisovalerate, N-acetylthreonine, 1-stearoylglycerophosphocholine, 2-stearoylglycerophosphocholine*, hydroquinone sulfate, 1-myristoylglycerophosphocholine), 1 identified metabolites (X-11445–5-alpha-pregnan-3beta,20alpha-disulfate) and 9 unknown metabolites (X-03088, X-11820, X-11852, X-12040, X-12189, X-12261, X-12726, X-12850, X-14632) in Table 1. The *P* values obtained from IVW analysis for the aforementioned estimates were below 0.05, and the findings from MREgger and WM methods exhibited consistent effects. This suggests the robustness of the two causal discoveries mentioned above (X-12189 and X-12261). To be more specific, X-03088 (OR: 0.66, 95% CI: 0.48–0.91, *P* = 0.0105), 3-methylxanthine (OR: 0.81, 95% CI: 0.66–0.99, *P* = 0.0424), adrenate (22:4n6) (OR: 0.59, 95% CI: 0.38–0.93, *P* = 0.0214), X-11820 (OR: 0.76, 95% CI: 0.60–0.96, *P* = 0.0227), X-12189 (OR: 0.95, 95% CI: 0.90–0.99, *P* = 0.0289), X-12261 (OR: 0.88, 95% CI: 0.82–0.95, *P* = 0.0013), N-acetylthreonine (OR: 0.64, 95% CI: 0.41–1.00, *P* = 0.0486), and X-12726 (OR: 0.86, 95% CI: 0.76–0.98, *P* = 0.0193) decreased risk of sciatica or/and lower back pain significantly; tyrosine (OR: 1.94, 95% CI: 1.15–3.25, *P* = 0.0127), pentadecanoate (15:0) (OR: 0.66, 95% CI: 0.48–0.91, *P* = 0.0373), aspartate (OR: 1.72, 95% CI: 1.01–2.92, *P* = 0.0472), 1,5-anhydroglucitol (1,5-AG) (OR: 1.38, 95% CI: 1.04–1.84, *P* = 0.0251), 1-palmitoylglycerol (1-monopalmitin) (OR: 1.55, 95% CI: 1.01–2.36, *P* = 0.0438), glycine (OR: 1.38, 95% CI: 1.10–1.74, *P* = 0.0064), C-glycosyltryptophan* (OR: 2.25, 95% CI: 1.17–4.29, *P* = 0.0144), X-11445-5-alpha-pregnan-3beta,20alpha-disulfate (OR: 1.14, 95% CI: 1.01–1.29, *P* = 0.0328), X-12040 (OR: 1.04, 95% CI: 1.00–1.09, *P* = 0.0497), 1-stearoylglycerophosphocholine (OR: 1.61, 95% CI: 1.12–2.30, *P* = 0.0093), X-12850 (OR: 1.42, 95% CI: 1.02–1.97, *P* = 0.0399), hydroquinone sulfate (OR: 1.09, 95% CI: 1.00–1.18, *P* = 0.0426), 1-myristoylglycerophosphocholine (OR: 1.53, 95% CI: 1.02–2.28, *P* = 0.0382), and X-14632 (OR: 1.17, 95% CI: 1.02–1.34, *P* = 0.0261) increased risk of sciatica or/and lower back pain significantly.

**Table 1:** Causal effect of 28 metabolites on the risk of sciatica or/and lower back pain derived from IVW.

Metabolite	Status	ID	Super-pathway	nsnp	Methods	*P*-value	OR(95% CI)	P_ivw_fdr
Tyrosine	Known	M01299	Amino acid	34	IVW	0.012722851	1.94 (1.15–3.25)	0.04971104
Malate	Known	M01303	Energy	17	IVW	0.031354846	1.62 (1.04–2.51)	0.04971104
Pentadecanoate (15:0)	Known	M01361	Lipid	19	IVW	0.037335145	1.52 (1.02–2.26)	0.04971104
X-03088	Unknown	M12768		18	IVW	0.010528692	0.66 (0.48–0.91)	0.04971104
Benzoate	Known	M15778	Xenobiotics	41	IVW	0.022693254	1.58 (1.07–2.34)	0.04971104
Aspartate	Known	M15996	Amino acid	4	IVW	0.047249796	1.72 (1.01–2.92)	0.04971104
1,5-anhydroglucitol (1,5-AG)	Known	M20675	Carbohydrate	31	IVW	0.025052913	1.38 (1.04–1.84)	0.04971104
1-palmitoylglycerol (1-monopalmitin)	Known	M21127	Lipid	13	IVW	0.043766282	1.55 (1.01–2.36)	0.04971104
Levulinate (4-oxovalerate)	Known	M22177	Amino acid	58	IVW	0.041658466	0.71 (0.52–0.99)	0.04971104
glycine	Known	M32338	Amino acid	26	IVW	0.006365201	1.38 (1.10–1.74)	0.04971104
3-methylxanthine	Known	M32445	Xenobiotics	14	IVW	0.042353222	0.81 (0.66–0.99)	0.04971104
C-glycosyltryptophan*	Known	M32675	Amino acid	23	IVW	0.014432888	2.25 (1.17–4.29)	0.04971104
X-11445-5-alpha-pregnan-3beta, 20alpha-disulfate	Identified	M32762	Lipid	15	IVW	0.032827454	1.14 (1.01–1.29)	0.04971104
Adrenate (22:4n6)	Known	M32980	Lipid	11	IVW	0.021400436	0.59 (0.38–0.93)	0.04971104
X-11820	Unknown	M33165		13	IVW	0.022723437	0.76 (0.60–0.96)	0.04971104
X-11852	Unknown	M33197		9	IVW	0.028584098	0.88 (0.78–0.99)	0.04971104
X-12040	Unknown	M33391		16	IVW	0.049711039	1.04 (1.00–1.09)	0.04971104
X-12189	Unknown	M33610		29	IVW	0.028910647	0.95 (0.90–0.99)	0.04971104
X-12261	Unknown	M33683		12	IVW	0.001304213	0.88 (0.82–0.95)	0.03651797
Alpha-hydroxyisovalerate	Known	M33937	Amino acid	15	IVW	0.049135231	1.30 (1.00–1.69)	0.04971104
N-acetylthreonine	Known	M33939	Amino acid	13	IVW	0.048624068	0.64 (0.41–1.00)	0.04971104
1-stearoylglycerophosphocholine	Known	M33961	Lipid	13	IVW	0.009284667	1.61 (1.12–2.30)	0.04971104
X-12726	Unknown	M34336		20	IVW	0.019264956	0.86 (0.76–0.98)	0.04971104
X-12850	Unknown	M34533		15	IVW	0.022709535	1.24 (1.03–1.49)	0.04971104
2-stearoylglycerophosphocholine*	Known	M35255	Lipid	13	IVW	0.039937189	1.42 (1.02–1.97)	0.04971104
Hydroquinone sulfate	Known	M35322	Xenobiotics	17	IVW	0.04261585	1.09 (1.00–1.18)	0.04971104
1-myristoylglycerophosphocholine	Known	M35626	Lipid	6	IVW	0.038201587	1.53 (1.02–2.28)	0.04971104
X-14632	Unknown	M36559		18	IVW	0.026127176	1.17 (1.02–1.34)	0.04971104

IVW, inverse variance weighted; OR, odds ratio; CI, confidence interval; fdr, false discovery rate.

#### Heterogeneity analysis and pluripotency analysis

**Figure 2:**
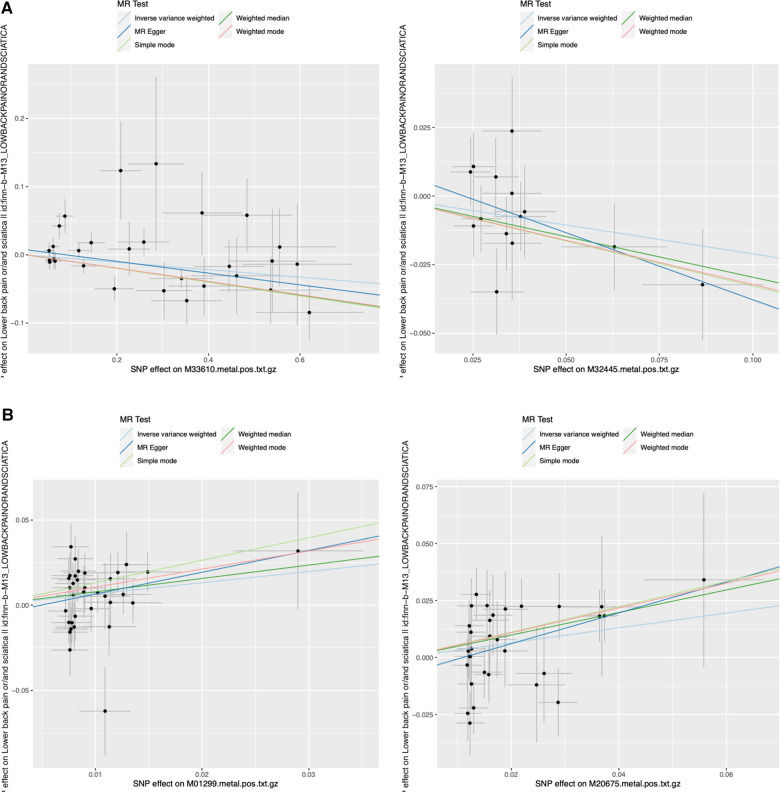
Representative scatter plots of the 5MR models for 22 screened metabolites with potential causal relationship with sciatica or/and lower back pain. MR, Mendelian randomization; SNP, single nucleotide polymorphism. **(A)** 2 metabolites (M33610 and M32445) decreased risk of sciatica or/and lower back pain significantly; **(B)** 2 metabolites (M01299 and M20675) increased risk of sciatica or/and lower back pain significantly.

**Figure 3:**
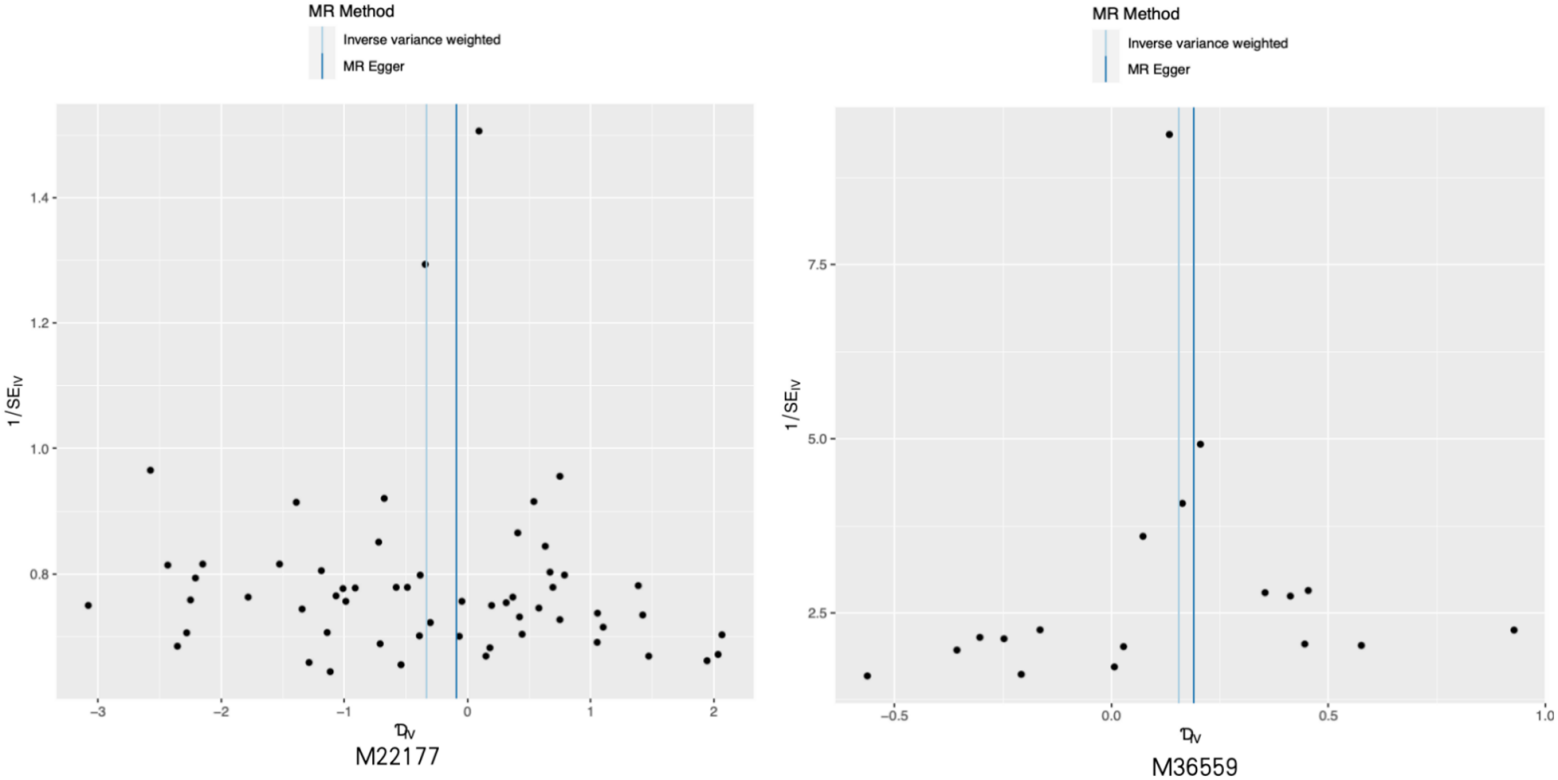
Representative funnel plot maps of 28 metabolites with potential causal relationship with sciatica or/and lower back pain. MR, Mendelian randomization.

The comparison between serum metabolites and sciatica or/and lower back pain was examined using Cochran's Q test for heterogeneity. The results indicated that there was no significant heterogeneity, with IVW and MR Egger. Consequently, the fixed-effects model was chosen for MR analysis. Only two comparisons [adrenate (22:4n6) and X-12189 on sciatica or/and lower back pain] showed horizontal pleiotropy causing that *P* values were <0.05 in MR-Egger and IVW Cochran's Q tests. The analysis outcomes for heterogeneity and pluripotency are displayed in Table 2. To evaluate the MR analysis results, five methods were employed, and scatter plots were produced (Figure 2; Supplementary Figure S1). Among these methods, MR-Egger was utilized to assess the pluripotency of IVs. The intercept of MR-Egger did not significantly differ from the zero intercept of IVW, indicating the absence of horizontal pluripotency. Furthermore, as demonstrated by the funnel plot (Figure 3; Supplementary Figure S2), there was no apparent symmetry in the variation of effect size around the estimated point. This lack of symmetry can be attributed to the limited sample size of the SNP and should not be interpreted as an absence of horizontal pleiotropy.

**Table 2:** Five MR models estimate the causal relationships between 17 known metabolites and the risk of sciatica or/and lower back pain and tests for heterogeneity and horizontal pleiotropy.

Metabolite	Methods	SNP (*N*)	OR (95% CI)	*P*	Heterogeneity	*P*	Pleiotropy	*P*
					Q value (*I*^2^)		Intercept	
Tyrosine	IVW	34	1.94 (1.15–3.25)	0.0127	41.137532	0.1562	−0.00624461	0.5391
	WM	34	2.19 (1.11–4.32)	0.0233				
	MR Egger	34	3.60 (0.47–27.25)	0.2245	40.64795696	0.1404		
	Simple mode	34	3.74 (1.16–12.10)	0.0345				
	Weighted mode	34	2.89 (1.06–7.86)	0.0458				
Malate	IVW	17	1.62 (1.04–2.51)	0.0313	16.94279894	0.3893	−0.00428789	0.5391
	WM	17	1.38 (0.74–2.57)	0.3099				
	MR Egger	17	2.13 (0.38–11.97)	0.4035	16.82488428	0.3294		
	Simple mode	17	0.79 (0.22–2.83)	0.7202				
	Weighted mode	17	0.75 (0.22–2.57)	0.6478				
Pentadecanoate (15:0)	IVW	19	1.52 (1.02–2.26)	0.0373	16.82066286	0.5354	0.00390550	0.6647
	WM	19	1.38 (0.78–2.44)	0.2747				
	MR Egger	19	1.22 (0.41–3.56)	0.7260	16.62615904	0.4799		
	Simple mode	19	1.30 (0.50–3.38)	0.5937				
	Weighted mode	19	1.27 (0.55–2.91)	0.5792				
Benzoate	IVW	41	1.58 (1.07–2.34)	0.0226	43.06676712	0.3414	0.00487946	0.4455
	WM	41	1.07 (0.60–1.91)	0.8187				
	MR Egger	41	1.05 (0.34–3.20)	0.9383	42.42081944	0.3257		
	Simple mode	41	1.02 (0.27–3.85)	0.9743				
	Weighted mode	41	0.92 (0.36–2.37)	0.8642				
Aspartate	IVW	4	1.72 (1.01–2.92)	0.0472	1.677104801	0.6420	−0.01298943	0.6053
	WM	4	1.80 (0.88–3.68)	0.1093				
	MR Egger	4	2.66 (0.59–12.04)	0.3322	1.308132089	0.5199		
	Simple mode	4	1.8 (0.71–4.63)	0.3009				
	Weighted mode	4	2.05 (0.86–4.87)	0.2035				
1,5-anhydroglucitol (1,5-AG)	IVW	31	1.38 (1.04–1.84)	0.0250	40.20802274	0.1008	−0.00746007	0.2695
	WM	31	1.64 (1.16–2.32)	0.0053				
	MR Egger	31	1.97 (1.00–3.86)	0.0587	38.52508271	0.1110		
	Simple mode	31	1.75 (0.89–3.40)	0.1127				
	Weighted mode	31	1.72 (1.14–2.61)	0.0158				
1-palmitoylglycerol (1-monopalmitin)	IVW	13	1.55 (1.01–2.36)	0.0437	9.177034605	0.6877	−0.01080479	0.3418
	WM	13	1.95 (1.08–3.53)	0.0278				
	MR Egger	13	2.96 (0.77–11.43)	0.1432	8.190221082	0.6961		
	Simple mode	13	1.98 (0.89–4.43)	0.1218				
	Weighted mode	13	1.98 (1.00–3.92)	0.0735				
Levulinate (4-oxovalerate)	IVW	58	0.71 (0.52–0.99)	0.0416	52.28226369	0.6523	−0.00262907	0.6129
	WM	58	0.74 (0.46–1.20)	0.2175				
	MR Egger	58	0.91 (0.34–2.45)	0.8540	52.02351508	0.6261		
	Simple mode	58	1.37 (0.48–3.92)	0.5590				
	Weighted mode	58	1.21 (0.49–3.00)	0.6879				
Glycine	IVW	26	1.38 (1.10–1.74)	0.0063	23.82460719	0.5295	−0.00230829	0.5915
	WM	26	1.29 (0.96–1.72)	0.0924				
	MR Egger	26	1.50 (1.03–2.17)	0.0436	23.52878324	0.4887		
	Simple mode	26	1.50 (0.72–3.12)	0.2836				
	Weighted mode	26	1.31 (0.98–1.76)	0.0830				
3-methylxanthine	IVW	14	0.81 (0.66–0.99)	0.0423	11.22630354	0.5918	0.01109903	0.3165
	WM	14	0.74 (0.56–0.98)	0.0357				
	MR Egger	14	0.61 (0.35–1.07)	0.1128	10.13397786	0.6042		
	Simple mode	14	0.72 (0.47–1.11)	0.1598				
	Weighted mode	14	0.72 (0.53–1.00)	0.0708				
C-glycosyltryptophan*	IVW	23	2.25 (1.17–4.29)	0.0144	25.18286258	0.2884	−0.00822356	0.4247
	WM	23	4.27 (1.77–10.29)	0.0012				
	MR Egger	23	4.61 (0.73–29.25)	0.1203	24.41234694	0.2735		
	Simple mode	23	3.46 (0.72–16.71)	0.1361				
	Weighted mode	23	4.04 (1.49–10.99)	0.0120				
Alpha-hydroxyisovalerate	IVW	15	1.30 (1.00–1.69)	0.0491	10.22633811	0.7454	0.01512764	0.1892
	WM	15	1.22 (0.85–1.74)	0.2789				
	MR Egger	15	0.79 (0.38–1.67)	0.5547	8.307204266	0.8230		
	Simple mode	15	0.95 (0.55–1.66)	0.8710				
	Weighted mode	15	1.17 (0.77–1.80)	0.4736				
N-acetylthreonine	IVW	13	0.64 (0.41–1.00)	0.0486	4.628821118	0.9692	0.01067013	0.3897
	WM	13	0.72 (0.39–1.32)	0.2853				
	MR Egger	13	0.37 (0.10–1.33)	0.1549	3.82715867	0.9747		
	Simple mode	13	0.71 (0.30–1.72)	0.4661				
	Weighted mode	13	0.73 (0.34–1.56)	0.4304				
1-stearoylglycerophosphocholine	IVW	13	1.61 (1.12–2.30)	0.0092	5.960087592	0.9180	−0.00420964	0.6361
	WM	13	1.63 (0.98–2.71)	0.0597				
	MR Egger	13	1.89 (0.90–3.94)	0.1197	5.723386705	0.8911		
	Simple mode	13	1.51 (0.71–3.20)	0.3076				
	Weighted mode	13	1.74 (0.92–3.28)	0.1132				
2-stearoylglycerophosphocholine*	IVW	13	1.42 (1.02–1.97)	0.0399	13.83377936	0.3114	−0.00361926	0.8609
	WM	13	1.34 (0.86–2.10)	0.2005				
	MR Egger	13	1.65 (0.30–9.14)	0.5772	13.79345246	0.2446		
	Simple mode	13	1.04 (0.45–2.40)	0.9287				
	Weighted mode	13	0.89 (0.39–2.05)	0.7973				
Hydroquinone sulfate	IVW	17	1.09 (1.00–1.18)	0.0426	16.28328708	0.4333	0.00265168	0.6987
	WM	17	1.09 (0.97–1.23)	0.1294				
	MR Egger	17	1.06 (0.92–1.23)	0.4230	16.11607698	0.3743		
	Simple mode	17	1.24 (1.04–1.48)	0.0297				
	Weighted mode	17	1.08 (0.96–1.22)	0.2353				
1-myristoylglycerophosphocholine	IVW	6	1.53 (1.02–2.28)	0.0382	5.957023329	0.3104	0.00578761	0.7165
	WM	6	1.43 (0.90–2.30)	0.1334				
	MR Egger	6	1.27 (0.46–3.53)	0.6695	5.73908646	0.2194		
	Simple mode	6	1.20(0.65–2.24)	0.5836				
	Weighted mode	6	1.37(0.88–2.14)	0.2238				

MR, Mendelian randomization; SNP, single nucleotide polymorphism; WM, weighted median; IVW, inverse variance weighted; OR, odds ratio; CI, confidence interval.

#### Leave-one-out analysis

**Figure 4:**
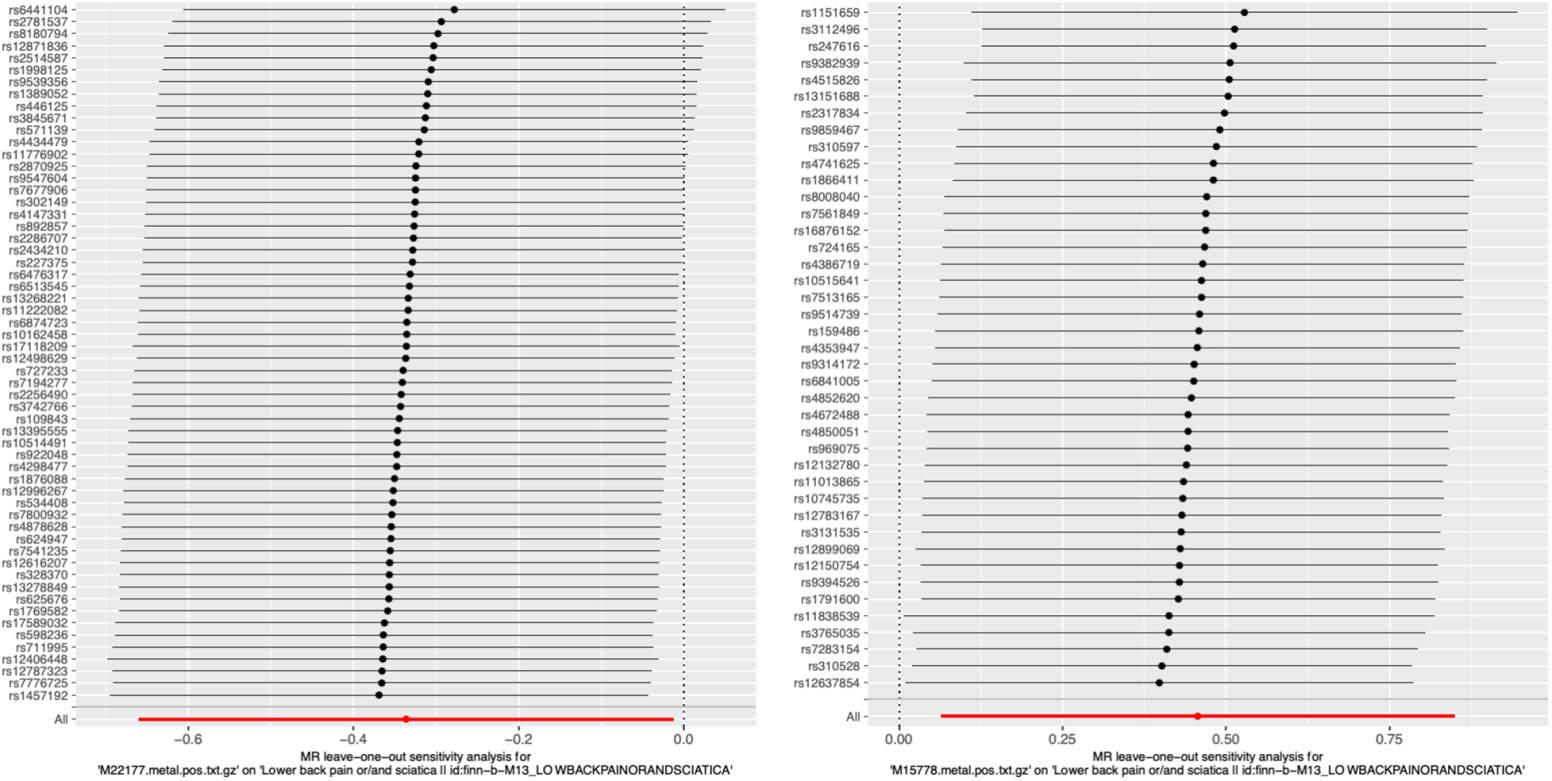
Representative leave-one-out forest maps of 28 metabolites with potential causal relationship with sciatica or/and lower back pain. MR, Mendelian randomization.

We performed an analysis called leave-one-out to compute the MR result of the remaining IVs when each one was removed individually. Removing each SNP did not have a significant impact on the overall error line, as shown in Figure 4; Supplementary Figure S3. Hence, the correlation analysis results from the two-sample MR study were relatively dependable.

### Influence of sciatica or/and lower back pain on the candidate serum metabolites (reverse MR)

Reverse MR analysis was subsequently conducted to assess the impact of sciatica and/or lower back pain on the 28 candidate serum metabolites identified in the forward MR analysis. SNPs that met a significance threshold of *P* < 1 × 10^−5^ were selected as genetic instruments closely linked to sciatica and/or lower back pain and utilized for LD clumping. However, no association was found between genetic predisposition to sciatica and/or lower back pain and changes in the concentrations of the candidate metabolites in the IVW analysis, as well as in the simple mode, weighted mode, weighted median, and MR-Egger analyses (Table 3).

**Table 3:** Complementary reverse MR analyses of sciatica or/and lower back pain for causal association with blood metabolites.

Method	nsnp	b	se	*p*-val	or	or_lci95	or_uci95	Metabolite
MR egger	5	−0.0854972	0.19588729	0.69200543	0.91805572	0.6253547	1.34775721	Tyrosine
Weighted median	5	−0.0023432	0.02254382	0.91721674	0.99765953	0.95453679	1.04273042	Tyrosine
Inverse variance weighted	5	−0.0039803	0.01796736	0.82467866	0.99602758	0.96156187	1.03172865	Tyrosine
Simple mode	5	−0.0358256	0.0349957	0.36384022	0.96480855	0.90084941	1.0333087	Tyrosine
Weighted mode	5	0.00381592	0.02988523	0.9045594	1.00382321	0.94671316	1.06437841	Tyrosine
MR egger	5	0.33504399	0.3862299	0.44950429	1.39800189	0.65575593	2.98039131	Malate
Weighted median	5	−0.0044629	0.03783116	0.90609198	0.99554703	0.92439853	1.07217164	Malate
Inverse variance weighted	5	−0.0155535	0.0398748	0.69649392	0.98456686	0.91054852	1.06460213	Malate
Simple mode	5	−0.021905	0.04830062	0.67369343	0.97833319	0.88996406	1.07547693	Malate
Weighted mode	5	0.00166031	0.04030993	0.96911937	1.00166169	0.92556848	1.08401071	Malate
MR egger	5	−0.177975	0.31347336	0.60991571	0.83696337	0.45276468	1.54717829	Pentadecanoate (15:0)
Weighted median	5	−0.0511608	0.03564277	0.15117991	0.95012589	0.88601569	1.01887497	Pentadecanoate (15:0)
Inverse variance weighted	5	−0.0211619	0.0301038	0.48207703	0.9790604	0.92296369	1.03856661	Pentadecanoate (15:0)
Simple mode	5	−0.0466761	0.05043052	0.40706366	0.95439647	0.86457266	1.05355243	Pentadecanoate (15:0)
Weighted mode	5	−0.0434617	0.04680136	0.40563986	0.95746927	0.8735479	1.04945292	Pentadecanoate (15:0)
MR Egger	5	−0.4961554	0.32110831	0.22002515	0.60886701	0.3244812	1.14249775	X-03088
Weighted median	5	−0.0557931	0.03804009	0.14246014	0.94573483	0.87778673	1.0189427	X-03088
Inverse variance weighted	5	−0.0456434	0.03178195	0.15096226	0.95538263	0.89768507	1.01678863	X-03088
Simple mode	5	−0.0589901	0.05266659	0.32539219	0.94271606	0.85025697	1.04522938	X-03088
Weighted mode	5	−0.0589901	0.05071665	0.30943361	0.94271606	0.85351277	1.04124226	X-03088
MR egger	5	−0.4991369	0.3160821	0.21242377	0.60705437	0.32671801	1.12792988	Benzoate
Weighted median	5	−0.0507307	0.03268731	0.12066189	0.95053457	0.89154633	1.0134257	Benzoate
Inverse variance weighted	5	−0.0402525	0.03862357	0.29733051	0.96054685	0.89051556	1.0360855	Benzoate
Simple mode	5	−0.0690743	0.05442734	0.27322199	0.93325736	0.8388261	1.03831927	Benzoate
Weighted mode	5	−0.0608969	0.04737793	0.2680446	0.94092023	0.85747979	1.03248016	Benzoate
MR egger	5	0.6209912	0.53332726	0.32845148	1.86077153	0.65420781	5.29261595	Aspartate
Weighted median	5	0.08706447	0.05780041	0.13199141	1.09096702	0.97411655	1.22183432	Aspartate
Inverse variance weighted	5	0.06567136	0.05454746	0.22861637	1.06787571	0.95959721	1.18837209	Aspartate
Simple mode	5	0.11205619	0.08097641	0.23862332	1.11857571	0.95441416	1.31097345	Aspartate
Weighted mode	5	0.09798253	0.07807862	0.27782555	1.10294352	0.94643635	1.28533144	Aspartate
MR egger	5	0.33547239	0.33755401	0.39356003	1.39860091	0.72170886	2.71035126	1,5-anhydroglucitol (1,5-AG)
Weighted median	5	0.05197192	0.04177948	0.21351505	1.05334616	0.97052718	1.14323242	1,5-anhydroglucitol (1,5-AG)
Inverse variance weighted	5	0.04379101	0.03249313	0.17775477	1.04476398	0.98030108	1.11346586	1,5-anhydroglucitol (1,5-AG)
Simple mode	5	0.08387898	0.06064425	0.23881558	1.08749727	0.96562109	1.22475609	1,5-anhydroglucitol (1,5-AG)
Weighted mode	5	0.07372053	0.0565583	0.26238365	1.07650591	0.96354727	1.20270693	1,5-anhydroglucitol (1,5-AG)
MR egger	5	0.6485749	0.33090886	0.14485497	1.91281293	0.99999354	3.65887694	1-palmitoylglycerol (1-monopalmitin)
Weighted median	5	0.02532746	0.04437735	0.56818307	1.02565092	0.94020989	1.11885636	1-palmitoylglycerol (1-monopalmitin)
Inverse variance weighted	5	0.06325774	0.03547732	0.07457862	1.06530138	0.99374185	1.14201392	1-palmitoylglycerol (1-monopalmitin)
Simple mode	5	0.02588095	0.05395567	0.65651051	1.02621876	0.92323435	1.14069082	1-palmitoylglycerol (1-monopalmitin)
Weighted mode	5	0.0165372	0.04994652	0.75718925	1.01667469	0.92186363	1.12123681	1-palmitoylglycerol (1-monopalmitin)
MR egger	5	0.24073975	0.3003398	0.48142156	1.2721899	0.70615095	2.29195634	Levulinate (4-oxovalerate)
Weighted median	5	−0.0118012	0.02996496	0.69370522	0.98826821	0.93189748	1.04804882	Levulinate (4-oxovalerate)
Inverse variance weighted	5	−0.0122676	0.02743208	0.65473076	0.98780732	0.93609857	1.04237238	Levulinate (4-oxovalerate)
Simple mode	5	0.00693379	0.03506074	0.85287241	1.00695789	0.94008473	1.07858808	Levulinate (4-oxovalerate)
Weighted mode	5	−0.0051937	0.03484832	0.88873592	0.99481975	0.92913947	1.06514293	Levulinate (4-oxovalerate)
MR egger	5	−0.6388703	0.28739732	0.11273307	0.52788846	0.30054162	0.92721341	Glycine
Weighted median	5	−0.0192975	0.03796007	0.61119866	0.9808875	0.91055658	1.05665075	Glycine
Inverse variance weighted	5	0.00860266	0.03951999	0.82767951	1.00863977	0.9334606	1.08987372	Glycine
Simple mode	5	−0.036068	0.05403701	0.54100761	0.96457468	0.86763812	1.07234145	Glycine
Weighted mode	5	−0.0338114	0.04423844	0.48728637	0.9667538	0.88646046	1.0543199	Glycine
MR egger	5	1.10992895	0.60460703	0.16372331	3.03414281	0.92764994	9.92402648	3-methylxanthine
Weighted median	5	−0.0910748	0.07700952	0.23695066	0.91294945	0.78504565	1.06169203	3-methylxanthine
Inverse variance weighted	5	−0.0441794	0.06484606	0.49568352	0.95678227	0.84258755	1.08645363	3-methylxanthine
Simple mode	5	−0.13045	0.11589221	0.32329504	0.87770041	0.69935367	1.10152852	3-methylxanthine
Weighted mode	5	−0.1134378	0.10929198	0.35792231	0.89275969	0.72061511	1.10602714	3-methylxanthine
MR egger	5	0.29521241	0.17356194	0.1875181	1.34341168	0.95602713	1.88776541	C-glycosyltryptophan*
Weighted median	5	0.01994233	0.02046947	0.32993367	1.0201425	0.98002438	1.0619029	C-glycosyltryptophan*
Inverse variance weighted	5	0.00221394	0.01768024	0.90034839	1.00221639	0.96808118	1.03755525	C-glycosyltryptophan*
Simple mode	5	0.02099756	0.027108	0.48182146	1.02121956	0.96837669	1.07694598	C-glycosyltryptophan*
Weighted mode	5	0.02131383	0.02519105	0.44515055	1.02154259	0.97232941	1.07324663	C-glycosyltryptophan*
MR egger	5	0.63072079	1.07913247	0.59995231	1.87896442	0.22664308	15.5773887	X-11445-5-alpha-pregnan-3beta,20alpha-disulfate
Weighted median	5	0.16613334	0.13232569	0.20930159	1.18073053	0.91098849	1.5303427	X-11445-5-alpha-pregnan-3beta,20alpha-disulfate
Inverse variance weighted	5	0.10063878	0.10364484	0.33155002	1.1058771	0.90257354	1.35497453	X-11445-5-alpha-pregnan-3beta,20alpha-disulfate
Simple mode	5	0.15340235	0.17484278	0.42982117	1.16579394	0.8275469	1.64229426	X-11445-5-alpha-pregnan-3beta, 20alpha-disulfate
Weighted mode	5	0.1598392	0.1498807	0.34631408	1.17332218	0.87465394	1.57397672	X-11445-5-alpha-pregnan-3beta,20alpha-disulfate
MR egger	5	0.48584294	0.35953189	0.26946376	1.62554467	0.80345061	3.28880884	Adrenate (22:4n6)
Weighted median	5	0.00386839	0.045319	0.93197596	1.00387588	0.91855191	1.09712555	Adrenate (22:4n6)
Inverse variance weighted	5	0.00090973	0.0385908	0.9811926	1.00091014	0.92799565	1.07955368	Adrenate (22:4n6)
Simple mode	5	0.00163621	0.05976755	0.97947099	1.00163755	0.89091328	1.12612283	Adrenate (22:4n6)
Weighted mode	5	0.01317398	0.0551778	0.82302885	1.01326114	0.90939613	1.12898891	Adrenate (22:4n6)
MR egger	5	−0.4643047	0.56018623	0.46798065	0.62857198	0.20965966	1.88449576	X-11820
Weighted median	5	0.00763336	0.05865949	0.89646358	1.00766257	0.89822091	1.13043889	X-11820
Inverse variance weighted	5	−0.0336233	0.05295978	0.52550455	0.9669357	0.87160019	1.07269899	X-11820
Simple mode	5	0.03928611	0.08875141	0.68089993	1.04006801	0.87400721	1.23768026	X-11820
Weighted mode	5	0.04758269	0.07346755	0.55249607	1.04873291	0.90808826	1.2111606	X-11820
MR egger	5	−1.3506516	1.1660258	0.33056242	0.25907139	0.02635593	2.54659954	X-11852
Weighted median	5	0.10292783	0.1413394	0.46647178	1.10841141	0.84021506	1.46221593	X-11852
Inverse variance weighted	5	0.06101057	0.11801931	0.60518816	1.06291015	0.84340562	1.33954288	X-11852
Simple mode	5	0.08582335	0.20889075	0.70223727	1.08961383	0.72353777	1.64090714	X-11852
Weighted mode	5	0.11923981	0.18863434	0.56163553	1.12664006	0.77842432	1.63062457	X-11852
MR Egger	5	0.11505894	2.25374941	0.96249296	1.12193956	0.01353752	92.9821733	X-12040
Weighted median	5	−0.1471823	0.20516339	0.47313294	0.86313657	0.57735233	1.29038148	X-12040
Inverse variance weighted	5	−0.0526043	0.20035729	0.79289553	0.94875536	0.64062912	1.40508244	X-12040
Simple mode	5	−0.1550528	0.27922501	0.60828134	0.85636995	0.49542771	1.48027547	X-12040
Weighted mode	5	−0.1711649	0.25239378	0.534871	0.84268262	0.51383314	1.38199336	X-12040
MR Egger	5	−0.0882998	2.25048741	0.97116737	0.91548635	0.01111727	75.3885571	X-12189
Weighted median	5	−0.1133583	0.1543023	0.4625522	0.89283069	0.65981828	1.20813059	X-12189
Inverse variance weighted	5	−0.1549373	0.19972658	0.43789831	0.85646886	0.57902976	1.26684147	X-12189
Simple mode	5	−0.0958242	0.17866862	0.62017194	0.9086237	0.64017433	1.28964406	X-12189
Weighted mode	5	−0.1019418	0.1643682	0.56871028	0.90308207	0.65435614	1.24635069	X-12189
MR Egger	5	−0.7382044	1.10346773	0.55138214	0.47797138	0.05496816	4.15616314	X-12261
Weighted median	5	−0.0943967	0.14542014	0.51625365	0.90992169	0.68425802	1.21000772	X-12261
Inverse variance weighted	5	−0.0687457	0.11458425	0.54853428	0.93356403	0.74577536	1.1686385	X-12261
Simple mode	5	0.02713586	0.19351119	0.89525696	1.0275074	0.7031774	1.50142972	X-12261
Weighted mode	5	−0.1658632	0.17136157	0.38789831	0.84716211	0.60548113	1.18531132	X-12261
MR egger	5	0.24950691	0.37903353	0.55737107	1.28339244	0.61054773	2.69773526	Alpha-hydroxyisovalerate
Weighted median	5	0.01250205	0.047939	0.79425385	1.01258053	0.92177106	1.11233621	Alpha-hydroxyisovalerate
Inverse variance weighted	5	−0.0038674	0.04021529	0.9233885	0.99614011	0.92063713	1.07783522	Alpha-hydroxyisovalerate
Simple mode	5	0.01460613	0.06235864	0.82630846	1.01471332	0.89797164	1.14663213	Alpha-hydroxyisovalerate
Weighted mode	5	0.01602177	0.05829982	0.79706734	1.01615081	0.90642601	1.13915803	Alpha-hydroxyisovalerate
MR egger	5	−0.1848115	0.26889881	0.54126594	0.831261	0.49073396	1.40808441	N-acetylthreonine
Weighted median	5	−0.0196203	0.03543331	0.57976796	0.98057096	0.91478196	1.05109136	N-acetylthreonine
Inverse variance weighted	5	−0.0326356	0.02748315	0.23503973	0.9678912	0.9171332	1.02145837	N-acetylthreonine
Simple mode	5	−0.0129995	0.04539257	0.7888086	0.98708467	0.90305763	1.07893019	N-acetylthreonine
Weighted mode	5	−0.0164282	0.04286705	0.72104061	0.98370596	0.90443245	1.0699278	N-acetylthreonine
MR egger	5	−0.4286023	0.37742755	0.33864412	0.65141896	0.31087628	1.36500174	1-stearoylglycerophosphocholine
Weighted median	5	−0.0167922	0.05167574	0.74521578	0.98334796	0.88862789	1.08816437	1-stearoylglycerophosphocholine
Inverse variance weighted	5	−0.0288126	0.03902191	0.4602901	0.97159855	0.9000585	1.04882487	1-stearoylglycerophosphocholine
Simple mode	5	0.01787019	0.07482616	0.8229799	1.01803082	0.87915938	1.1788383	1-stearoylglycerophosphocholine
Weighted mode	5	0.01103125	0.0723071	0.8861306	1.01109232	0.87748918	1.16503737	1-stearoylglycerophosphocholine
MR egger	5	−0.1544371	0.36988976	0.70437803	0.8568974	0.41502313	1.76923428	X-12726
Weighted median	5	−0.0617819	0.04398202	0.16010816	0.9400879	0.86244262	1.02472355	X-12726
Inverse variance weighted	5	−0.0517978	0.03419749	0.12985665	0.94952082	0.88796328	1.01534581	X-12726
Simple mode	5	−0.0693098	0.05335253	0.26373407	0.93303757	0.8403971	1.03589018	X-12726
Weighted mode	5	−0.0732971	0.0419769	0.15571874	0.92932469	0.8559256	1.00901806	X-12726
MR Egger	5	1.3530209	0.81860035	0.1969322	3.86909604	0.77768339	19.2493557	X−12850
Weighted median	5	0.13693158	0.08734561	0.1169515	1.14674969	0.96631461	1.3608765	X-12850
Inverse variance weighted	5	0.07485632	0.09815148	0.4456655	1.07772929	0.88912222	1.30634507	X-12850
Simple mode	5	0.17305012	0.11938774	0.22079862	1.18892569	0.94087043	1.50237936	X-12850
Weighted mode	5	0.17817705	0.10452879	0.16347647	1.19503688	0.9736539	1.46675645	X-12850
MR egger	5	−0.2510884	0.40019817	0.57489627	0.77795356	0.35505703	1.704548	2-stearoylglycerophosphocholine*
Weighted median	5	0.0055393	0.04994732	0.91169335	1.00555467	0.91177918	1.10897487	2-stearoylglycerophosphocholine*
Inverse variance weighted	5	0.01253503	0.04129185	0.76145423	1.01261393	0.93388966	1.09797443	2-stearoylglycerophosphocholine*
Simple mode	5	−0.0429219	0.07713854	0.60757805	0.95798618	0.82356445	1.11434815	2-stearoylglycerophosphocholine*
Weighted mode	5	−0.0087549	0.07052316	0.90719113	0.99128332	0.86331099	1.13822554	2-stearoylglycerophosphocholine*
MR egger	5	−0.8312457	1.15281263	0.5229851	0.43550642	0.04546745	4.17146383	Hydroquinone sulfate
Weighted median	5	−0.0307505	0.1503818	0.83797601	0.96971745	0.722167	1.30212533	Hydroquinone sulfate
Inverse variance weighted	5	0.01299601	0.1232785	0.91604261	1.01308082	0.79562298	1.28997374	Hydroquinone sulfate
Simple mode	5	−0.0273272	0.17153903	0.88114802	0.97304281	0.69520834	1.36191161	Hydroquinone sulfate
Weighted mode	5	−0.0298236	0.18318561	0.87856561	0.97061674	0.67782416	1.38988387	Hydroquinone sulfate
MR egger	5	0.18616996	0.45615675	0.71056165	1.20462698	0.49267908	2.94537804	1-myristoylglycerophosphocholine
Weighted median	5	0.02206363	0.04878892	0.65110604	1.02230883	0.92907794	1.12489523	1-myristoylglycerophosphocholine
Inverse variance weighted	5	0.01007614	0.04246149	0.81242362	1.01012707	0.9294629	1.09779174	1-myristoylglycerophosphocholine
Simple mode	5	0.01356518	0.05666959	0.82258151	1.0136576	0.90709579	1.13273786	1-myristoylglycerophosphocholine
Weighted mode	5	0.02290476	0.0535431	0.69083529	1.02316909	0.92123537	1.13638167	1-myristoylglycerophosphocholine
MR egger	5	0.94831621	0.58697287	0.20459493	2.58135954	0.81697188	8.15623799	X-14632
Weighted median	5	−0.0544851	0.08240836	0.50851008	0.94697265	0.80573091	1.11297357	X-14632
Inverse variance weighted	5	−0.0270763	0.068096	0.69091093	0.973287	0.85167998	1.11225767	X-14632
Simple mode	5	−0.0981962	0.12411776	0.4731228	0.90647105	0.71072693	1.15612585	X-14632
Weighted mode	5	−0.1148048	0.11591728	0.37804111	0.89154016	0.71034631	1.11895261	X-14632

MR, Mendelian randomization; se, standard error; or, odds ratio; ci, confidence interval.

#### Colocalization analysis

For the 28 identified metabolites linked to sciatica or/and lower back pain risk in this study, we performed a colocalization analysis to evaluate their relationship with sciatica or/and lower back pain outcomes. This analysis used exposure data from a 1,000 kb region around the lead SNP to ensure that the observed associations were not influenced by the same causal variant within that area. This strategy aimed to reduce the chance of a false association between the two phenotypes (see Table 4). The results of the colocalization analysis showed that the associations between sciatica or/and lower back pain and the 28 identified metabolites were not due to shared causal variant sites.

**Table 4:** The colocalization analysis illustrating the associations between 28 metabolites and sciatica or/and lower back pain.

Metabolites	Leadsnp	Leadsnp-pos	n-snps	PP.H0.abf	PP.H1.abf	PP.H2.abf	PP.H3.abf	PP.H4.abf
Tyrosine	rs9400467	111530708	22636	2.33 × 10^−8^	7.38 × 10^−8^	0.23837271	0.75620936	0.00541784
Malate	rs7503429	77784714	35386	6.16 × 10^−5^	0.00034437	0.15128202	0.84623243	0.00207962
Pentadecanoate (15:0)	rs6887589	107039458	26230	0.02758996	0.0172441	0.58033314	0.36271448	0.01211832
X-03088	rs7915053	122897295	21047	0.27629586	0.11711713	0.40023426	0.16964433	0.03670841
Benzoate	rs247616	55547091	36802	0.08423923	0.12862998	0.29845603	0.45572821	0.03294654
Aspartate	rs7150776	92183574	24425	0.14165978	0.21887516	0.2458586	0.37986799	0.01373847
1,5-anhydroglucitol (1,5-AG)	rs7570971	135554376	15681	4.45 × 10^−38^	2.06 × 10^−38^	0.66915196	0.30928693	0.02156111
1-palmitoylglycerol (1-monopalmitin)	rs4774615	50320652	30195	0.01079206	0.13472718	0.0315126	0.39335417	0.42961399
Levulinate (4-oxovalerate)	rs13278849	26770791	38663	7.52 × 10^−7^	0.00010635	0.00702298	0.99271482	0.0001551
glycine	rs715	211251300	3598	3.78 × 10^−139^	2.94 × 10^−140^	0.92170244	0.07170822	0.00658935
3-methylxanthine	rs10754948	15223189	36656	0.01716998	0.03205128	0.32556308	0.60772877	0.0174869
C-glycosyltryptophan*	rs6867478	92148101	24565	0.04908301	0.07591868	0.29987615	0.46381238	0.11130978
X-11445-5-alpha-pregnan-3beta, 20alpha-disulfate	rs10491431	36003757	37681	4.56 × 10^−6^	1.39 × 10^−5^	0.2455182	0.74882144	0.00564188
Adrenate (22:4n6)	rs174550	61328054	31601	8.93 × 10^−18^	1.02 × 10^−17^	0.45886563	0.52313537	0.017999
X-11820	rs439401	50106291	29657	9.98 × 10^−13^	4.67 × 10^−10^	0.00196463	0.91885843	0.07917694
X-11852	rs7808781	52200991	33618	0.24172243	0.15339929	0.34379363	0.21817088	0.04291377
X-12040	rs11736351	81820692	32291	0.03218087	0.00699219	0.70630957	0.15345576	0.10106161
X-12189	rs8082167	49849155	29342	5.98 × 10^−6^	0.01547868	0.0003801	0.98361662	0.00051862
X-12261	rs8085059	13482909	38035	0.05475098	0.01621125	0.20419575	0.0604152	0.66442682
alpha-hydroxyisovalerate	rs2403254	18281722	37686	2.51 × 10^−24^	6.92 × 10^−24^	0.26079274	0.72025696	0.0189503
N-acetylthreonine	rs12857329	19736486	41762	0.38555856	0.07835232	0.42810682	0.08699769	0.02098461
1-stearoylglycerophosphocholine	rs476092	127624474	21498	0.02083686	0.14402321	0.10301523	0.71203131	0.0200934
X-12726	rs13419959	208457252	3608	0.047941	0.01245081	0.73115353	0.18987013	0.01858453
X-12850	rs2547231	53076869	35661	9.16 × 10^−10^	6.15 × 10^−10^	0.59209697	0.39771701	0.01018601
2-stearoylglycerophosphocholine*	rs2121073	75735493	31124	0.00644736	0.00215302	0.73144399	0.24425555	0.01570008
Hydroquinone sulfate	rs10514235	73039466	32355	0.00231155	0.15100163	0.01265641	0.8267767	0.0072537
1-myristoylglycerophosphocholine	rs780093	27596107	36086	0.00208826	0.10347737	0.01755898	0.87007992	0.00679546
X-14632	rs17152472	13000217	36939	0.40237235	0.15492452	0.30319159	0.11673553	0.02277602

snp, single nucleotide polymorphism; pos, position; PP, posterior probability.

#### Metabolic pathway analysis

**Figure 5:**
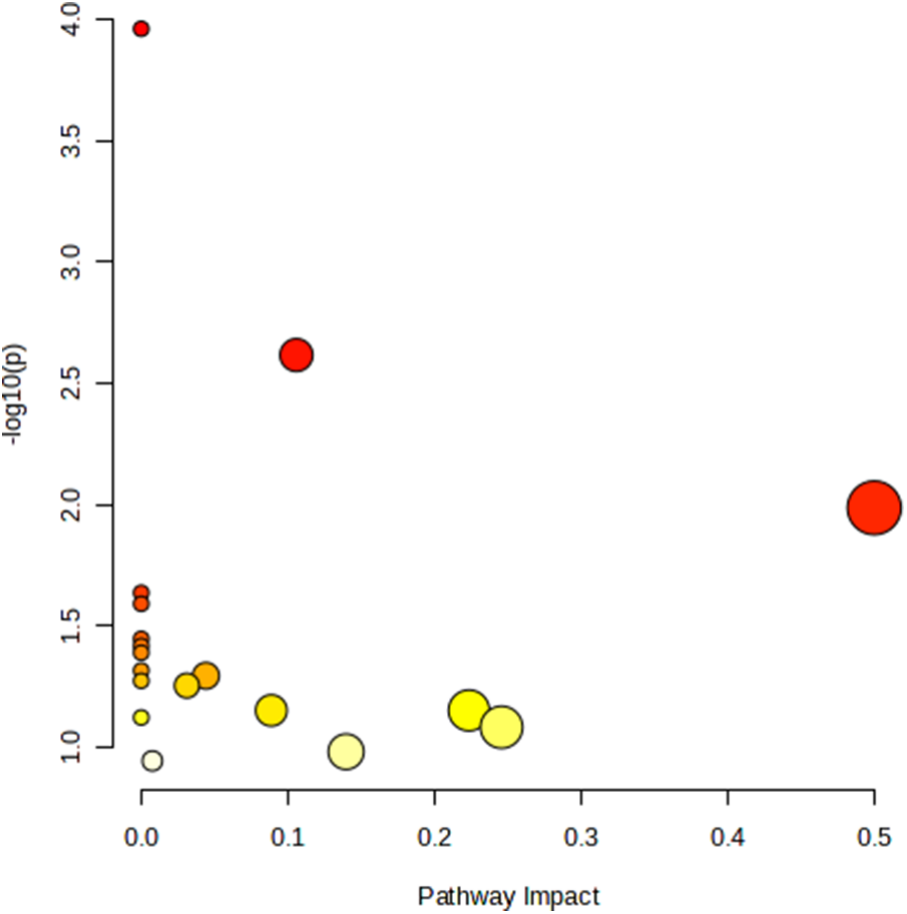
Metabolic pathway analysis and KEGG pathway enrichment analysis of 18 known metabolites by MetaboAnalyst5.0.

The metabolic pathway analysis identifed 11 signifcant metabolic pathways in sciatica or/and lower back pain (Figure 5). Our results show that the Aminoacyl-tRNA biosynthesis (*p* = 0.00010925), Glyoxylate and dicarboxylate metabolism (*p* = 0.0024154), Phenylalanine, tyrosine and tryptophan biosynthesis (*p* = 0.010293), Ubiquinone and other terpenoid-quinone biosynthesis (0.023046), Phenylalanine metabolism (*p* = 0.025582), Arginine biosynthesis (*p* = 0.035677), Nicotinate and nicotinamide metabolism (*p* = 0.038188), Histidine metabolism (*p* = 0.040694), Pantothenate and CoA biosynthesis (*p* = 0.048184) pathways were identified to be linked with the underlying mechanism of sciatica or/and lower back pain (Table 5).

**Table 5:** Signifcant metabolic pathways involved in sciatica or/and lower back pain.

Metabolite set	Total	Expected	Hits	*p*-value	Holm P	FDR
Aminoacyl-tRNA biosynthesis	48	0.12387	3	0.00010925	0.009177	0.009177
Glyoxylate and dicarboxylate metabolism	32	0.082581	2	0.0024154	0.20048	0.10145
Phenylalanine, tyrosine and tryptophan biosynthesis	4	0.010323	1	0.010293	0.84399	0.28819
Ubiquinone and other terpenoid-quinone biosynthesis	9	0.023226	1	0.023046	1	0.38941
Phenylalanine metabolism	10	0.025806	1	0.025582	1	0.38941
Arginine biosynthesis	14	0.036129	1	0.035677	1	0.38941
Nicotinate and nicotinamide metabolism	15	0.03871	1	0.038188	1	0.38941
Histidine metabolism	16	0.04129	1	0.040694	1	0.38941
Pantothenate and CoA biosynthesis	19	0.049032	1	0.048184	1	0.38941

FDR, false discovery rate.

## Discussion

### Principal findings

In this bidirectional comparison between two sets of samples, sciatica or/and lower back pain was found to have a suggestive connection with levels of some serum metabolites. We identifed 28 metabolites (18 known metabolites, 1 identified metabolites and 9 unknown metabolites) relevant to the risk of sciatica or/and lower back pain after using genetic variants as probes at P_IVW_ < 0.05 and FDR < 0.05. Among them, 8 serum metabolites decreased risk of sciatica or/and lower back pain significantly (*P* < 0.05), and 14 serum metabolites increased risk of sciatica or/and lower back pain significantly (*P* < 0.05). Our study did not identify any blood metabolites with bidirectional effects. Furthermore, such GWAS-GWAS co-localization analysis provided robust evidence of the causal relationships unaffected by overlapping SNPs. Pathway enrichment analysis identifed 11 signifcant metabolic pathways, which are mainly involved in the pathological mechanism of sciatica or/and lower back pain (*P* < 0.05).

### Previous studies and potential mechanisms

The pathological mechanism of sciatica or/and lower back pain is complex. On one hand, previous research has shown that pro-inflammatory cytokines can induce and exacerbate inflammatory reactions and neurogenic pain. Animal experiments have confirmed that the imbalance between pro-inflammatory and anti-inflammatory factors, as well as the conversion of anti-inflammatory cytokines to pro-inflammatory cytokines, can also increase the sensitivity of neurogenic pain (24). The characteristic of inflammatory pain is an increase in sensitivity to mechanical or thermal stimulation of the affected tissue. After tissue damage, local macrophages produce inflammatory reactions and further expand through migration of blood cells. Different inflammatory agents and tissue acidification work together to induce and sustain the progression of pain and hyperalgesia. On the other hand, discogenic sciatica or/and lower back pain is more likely due to the protrusion of the nucleus pulposus into the epidural space, which increases the sensitivity of sensory neurons to various stimuli. Pain is not always caused by nerve pain receptors, damage to nerve axons, and abnormal stimulation of the dorsal root ganglia (DRG) and dorsal horn of the spinal cord can also generate ectopic nerve impulses to induce pain. The sensory neurons of DRG are stimulated, increasing their overall activity while also lowering their threshold for various stimuli (25). DRG is adjacent to the spinal cord, innervating the limbs and core tissues, and transmitting electrical signals to the central nervous system (CNS) (26). These neurons can detect various stimuli and transmit proprioceptions, cold and hot sensations, mechanical pain, and injury sensations. The heightened function of primary sensory neurons enhances the release of neurotransmitters (like glutamate) and neuromodulators (including substance P, calcitonin gene-related peptide, and brain-derived neurotrophic factor) within the spinal cord. This results in an excessive activation of postsynaptic nociceptive neurons, a phenomenon known as central sensitization (27). Central sensitization is accountable for secondary pain experienced beyond the original injury site. Additionally, the activation of mitogen-activated protein kinase (MAPK) plays a crucial role in central sensitization and contributes to the development of pain hypersensitivity responses (28).

It can be observed from this that amino acid neurotransmitters play a crucial role in influencing the development of neuropathic pain in specific contexts. For instance, in a rat model of chronic constrictive injury, electroacupuncture (EA) relieves neuropathic pain by decreasing the release of excitatory amino acid transmitters like glutamate, glutamine, and aspartate, while enhancing the release of inhibitory amino acid transmitters such as glycine, gamma-aminobutyric acid (GABA), and taurine within the spinal cord (29). Glutamate, being one of the most abundant neurotransmitters in the spinal dorsal horn, assumes a key role in facilitating excitatory signaling associated with pain (30, 31). When utilizing a microdialysis method to collect spinal cord dialysates from rats with a spinal nerve ligation model, researchers noticed reduced levels of aspartate and glutamate in the dialysates obtained from rats treated with EA compared to sham EA controls. Based on these findings, Ma et al. concluded that EA has the ability to alleviate neuropathic pain in this experimental model by partially inhibiting the levels of aspartate and glutamate within the spinal dorsal horn (32).

In this MR analysis, specific amino acid metabolites such as tyrosine, aspartate, and glycine were identified as neurotransmitters associated with sciatica or/and lower back pain—findings consistent with previous research. Kobayashi et al. emphasize the significance of comprehending the vascular system's morphological characteristics that supply the dorsal root ganglion (DRG), as well as the neurogenic regulation of intraganglionic blood flow, in understanding the mechanisms behind low back pain and sciatica. This study confirmed the presence of dopaminergic nerves within the vasculature of the DRGs, which rely on the amino acid tyrosine from the bloodstream (33). Abnormalities in neurotransmitters consisted of elevated levels of glutamate and reduced levels of GABA. Watson's findings provide evidence that an imbalanced ratio of excitatory and inhibitory neurotransmitters, particularly in brain regions such as the insula, is present in chronic pain conditions. This imbalance contributes to heightened central pain processing and heightened sensitivity to pain (34). Maintaining glutamate homeostasis and regulating microglia activation are both significant factors in the progression and persistence of neuropathic pain. Yang et al. conducted a study and validated that the administration of ultra-low dose naloxone, either alone or in conjunction with morphine, had the ability to modify the levels of excitatory amino acids (EAAs) such as glutamate and aspartate. Additionally, they observed changes in the expression of tumor necrosis factor-α (TNF-α) and its receptors (TNFR1 and TNFR2) in the dorsal horn of the spinal cord in rats with partial transection of the sciatic nerve (35). Wang et al. discovered that the levels of certain serum metabolites related to linoleic acid metabolism, alanine, aspartate, and glutamate, glycerophospholipids, and the citrate cycle, exhibited a significant decrease following treatment with Lactobacillus paracasei S16 in LDH mice. Conversely, the metabolite associated with purine metabolism demonstrated a significant increase. These findings indicate that the administration of Lactobacillus paracasei S16 can ameliorate inflammatory responses, modulate gut microbiota, and influence serum metabolomics in an LDH mouse model, thereby contributing to the alleviation of low back pain (36). Multiple investigations have demonstrated that the natural neurosteroid allopregnanolone (AP) exhibits analgesic, neuroprotective, antidepressant, and anxiolytic effects. These beneficial effects are attributed to AP's ability to modulate GABAA receptors, glycine receptors, and L- and T-type calcium channels. This indicates that AP, with its high therapeutic potential and favorable toxicological profile, holds promise in developing effective and safe strategies for managing chronic neuropathic pain (37). Dysfunction of spinal strychnine-sensitive glycine receptors is associated with the development of chronic pain conditions and movement disorders. Therefore, enhancing the activity of glycine receptors could potentially serve as a treatment approach for such disorders. The ventral horn of the spinal cord, which is responsible for pain-induced motor reflexes and muscle tone regulation, contains the highest levels of glycine in the central nervous system. 4-Bromopropofol, acting through glycine receptors, has the potential to serve as a starting point for developing non-sedative, non-addictive muscle relaxants and analgesics for the treatment of low back pain (38,38). Furthermore, the correlation between the aforementioned serum metabolites, as confirmed by our MR study, aligns with previous research findings. Additionally, our MR study identified another 25 serum metabolites that have not been reported in the existing literature but hold potential as biomarkers for future investigations into the mechanisms and drug targets associated with lower back pain and/or sciatica.

In this study, the metabolic pathway analysis showed that Aminoacyl-tRNA biosynthesis, Glyoxylate and dicarboxylate metabolism, Phenylalanine, tyrosine and tryptophan biosynthesis, Phenylalanine metabolism, Ubiquinone and other terpenoid-quinone biosynthesis, Arginine biosynthesis, Nicotinate and nicotinamide metabolism, Histidine metabolism, Pantothenate and CoA biosynthesis pathways are mainly associated with sciatica or/and lower back pain (*P* < 0.05). Of them, Phenylalanine metabolism, Phenylalanine, tyrosine and tryptophan biosynthesis, Glycine, serine and threonine metabolism (*P* > 0.05), Tyrosine metabolism (*P* > 0.05), and Alanine, aspartate and glutamate metabolism (*P* > 0.05) were all belongs to amino acid neurotransmitter pathway which had been proven to be closely related to the pathogenesis of sciatica by our MR results and previous literatures. Altogether, these findings suggest that amino acid neurotransmitter pathway played an important role in the biological mechanism of asciatica or/and lower back pain.

### Strengths and limitations

Our research has many positive aspects. This analysis is the initial thorough MR investigation of lower back pain or/and sciatica with blood metabolites, examining proof of the bidirectional cause-and-effect relationship between blood metabolites on lower back pain or/and sciatica. Additionally, the MR methodology minimized the possibility of confounding variables and other biases influencing the reported bias. A substantial sample size and GWAS SNPs provided statistical reliability for evaluating causality. These measures enhance the validity of our conclusions. Third, both exposure and outcome were from the European population, avoiding population bias. Moreover, the samples of exposure and outcome in this study are from different databases, which reduces the possible bias in the effect sizes caused by the overlap of exposure and outcome samples. Co-localization analysis was also undertaken, reinforcing the credibility of our results.

However, our research does have certain limitations. Primarily, the majority of these GWAS data originate from populations in Europe. It is crucial to ascertain whether the conclusions we outlined remain applicable to other individuals. Secondly, we employed FDR multiple analysis correction for all 486 serum metabolites, and the all *p*-values after FDR corrections are greater than 0.05 (see Supplementary Table S3), which is because a large number of samples undergoing FDR correction simultaneously can result in false negatives. The primary objective of our study was to identify potential biomarkers for lower back pain and/or sciatica, as well as potential targets for treatment. However, implementing strict corrections such as Bonferroni or FDR correction could have resulted in the exclusion of significant findings. Therefore, this study only adjusted the *P*-values of 28 identified serum metabolites for FDR, and the adjusted *P*-values were all less than 0.05, which is consistent with previous results. Thus, we believe that such positive results are meaningful. Lastly, while this study encompassed a relatively extensive range of metabolites, the functions and mechanisms of some of these metabolites in the context of the disease were not entirely elucidated. Consequently, our interpretation of the results obtained from this MR analysis was limited.

## Conclusions

In conclusion, the comprehensive findings from this study indicate that there are 28 serum metabolites, including 18 known metabolites, 1 identified metabolite, and 9 unknown metabolites, which might have a causal role in the development of lower back pain and/or sciatica. These findings were derived from a large GWAS summary statistics database. Additionally, this research identified 11 significant metabolic pathways that could be relevant to the underlying pathology of lower back pain and/or sciatica. Overall, our results provide valuable insights into the potential use of certain metabolites as biomarkers for investigating targeted drug therapies for human diseases. However, further studies are necessary to validate these findings.

## Data Availability

The original contributions presented in the study are included in the article/Supplementary Material, further inquiries can be directed to the corresponding authors.

## Supplementary material

Supplementary Figure S1 All rest scatter plots of the 5MR models for 22 screened metabolites with potential causal relationship with sciatica or/and lower back pain.

Supplementary Figure S2 All rest funnel plot maps of 28 metabolites with potential causal relationship with sciatica or/and lower back pain.

Supplementary Figure S3 All rest leave-one-out forest maps of 28 metabolites with potential causal relationship with sciatica or/and lower back pain.

Supplementary Table S1 The STROBE-MR reporting specification.

Supplementary Table S2 The information on instrumental variables and SNPs identified by the Mendelian randomization estimates.

Supplementary Table S3 The all *p*-values after FDR corrections of all 486 serum metabolites.
