# Supplementary material for: Causality of genetically determined serum metabolites on lower back pain or/and sciatica: a comprehensive Mendelian randomized study
Source: Front Pain Res (Lausanne). 2024 Sep 25;5:1370704. doi: 10.3389/fpain.2024.1370704 (PMC11461461; doi:10.3389/fpain.2024.1370704)
Supplement: Supplementary Figure S1 — All rest scatter plots of the 5MR models for 22 screened metabolites with potential causal relationship with sciatica or/and lower back pain. [file Datasheet4.pdf]

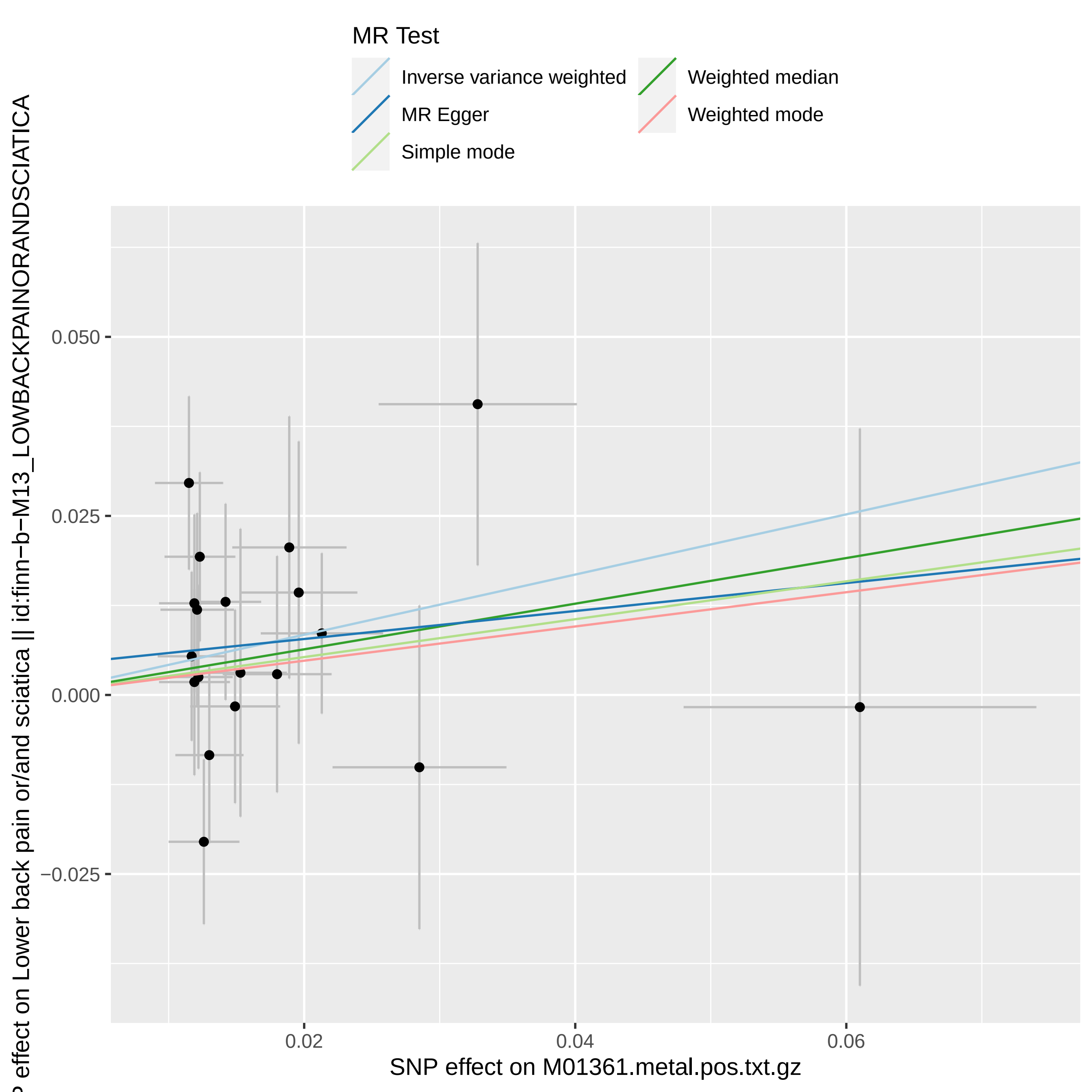
MR Test
Inverse variance weighted
MR Egger
Simple mode
Weighted median
Weighted mode
0.050
0.025
0.000
−0.025
0.02
0.04
0.06
SNP effect on M01361.metal.pos.txt.gz
SNP effect on Lower back pain or/and sciatica || id:finn−b−M13_LOWBACKPAINORANDSCIATICA

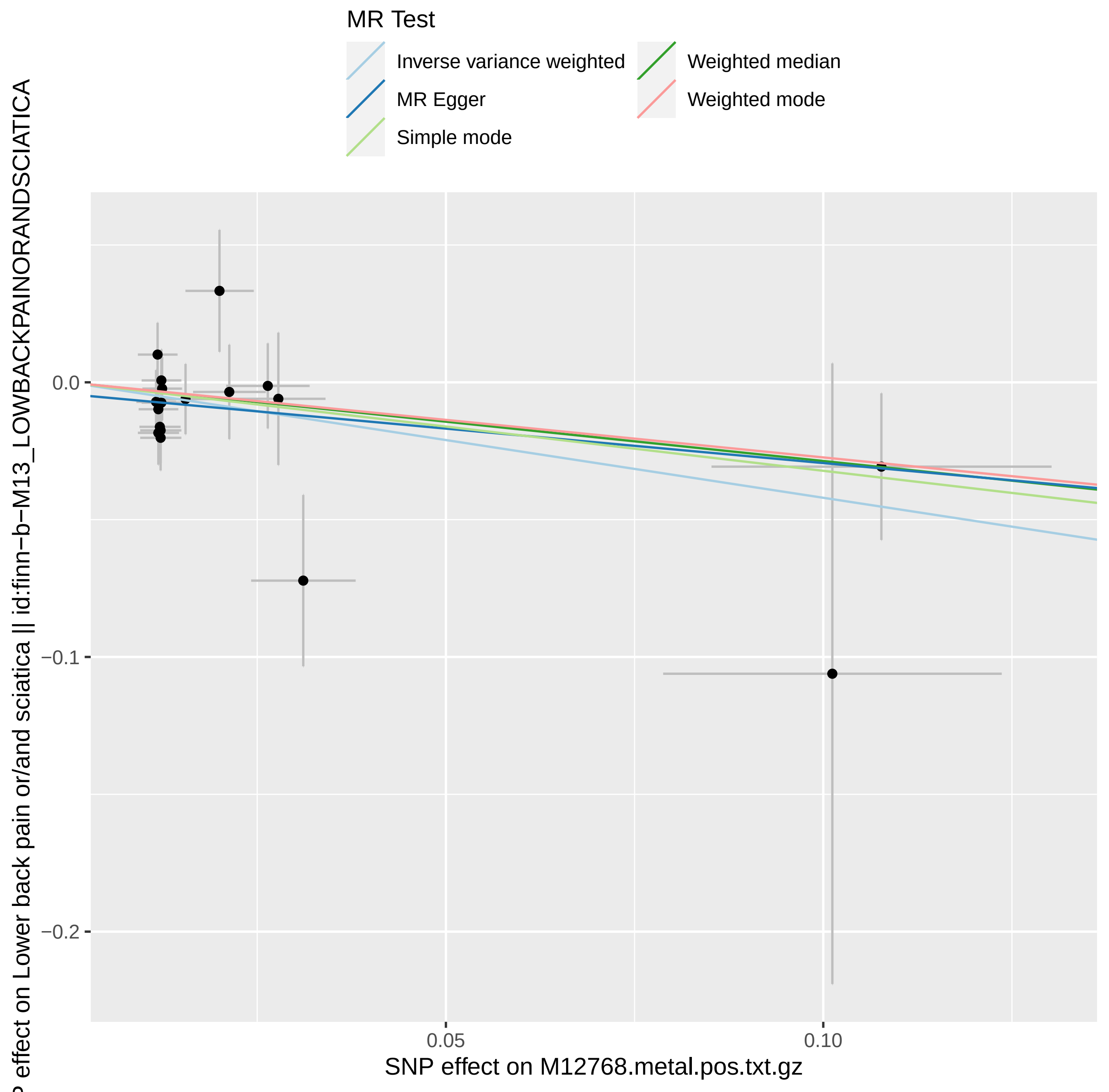

MR Test
Inverse variance weighted
MR Egger
Simple mode
Weighted median
Weighted mode
0.0
−0.1
−0.2
0.05
0.10
SNP effect on M12768.metal.pos.txt.gz
effect on Lower back pain or/and sciatica || id:finn−b−M13_LOWBACKPAINORANDSCIATICA

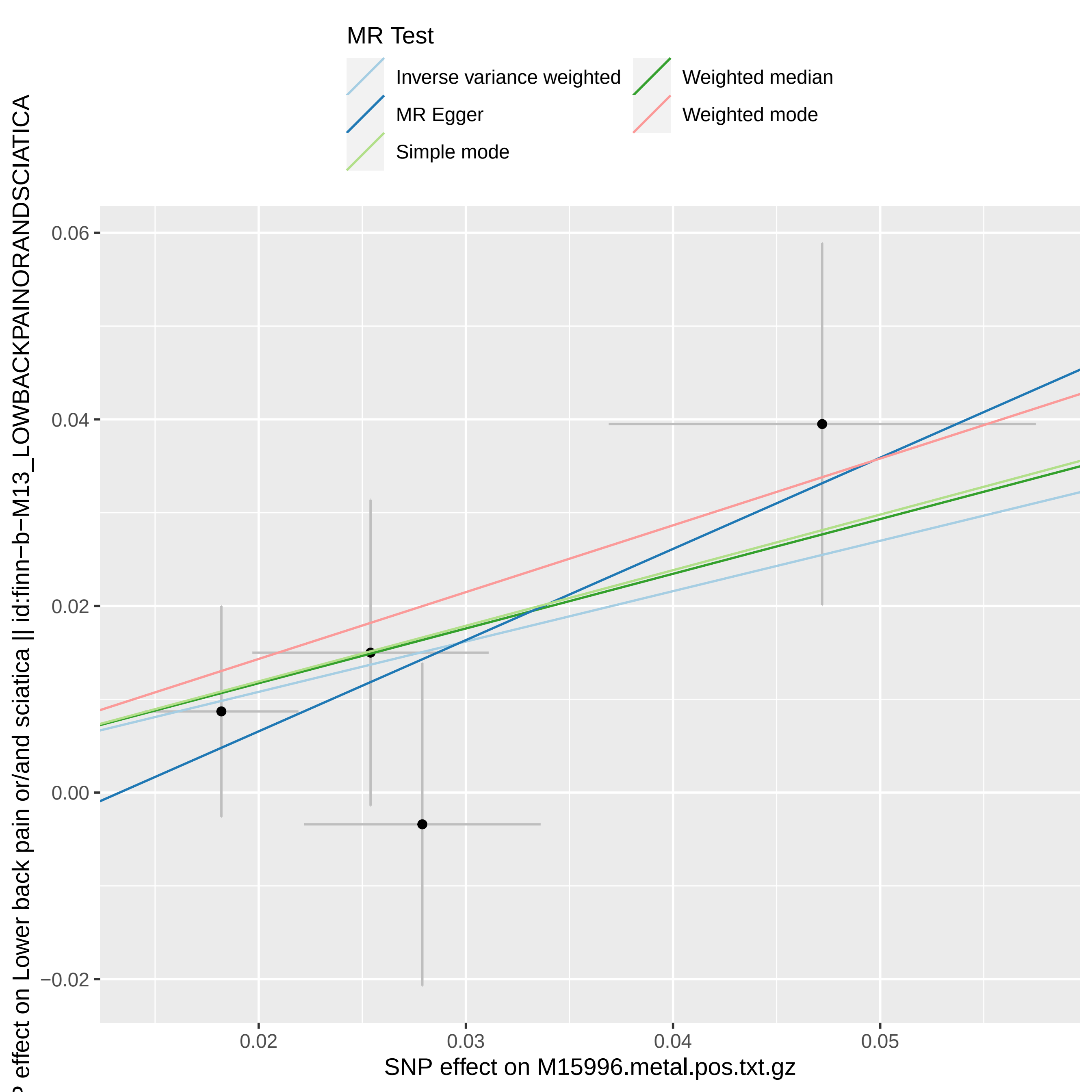

MR Test
Inverse variance weighted
MR Egger
Simple mode
Weighted median
Weighted mode
0.06
0.04
0.02
0.00
−0.02
0.02
0.03
0.04
0.05
SNP effect on M15996.metal.pos.txt.gz
P effect on Lower back pain or/and sciatica || id:finn−b−M13_LOWBACKPAINORANDSCIATICA

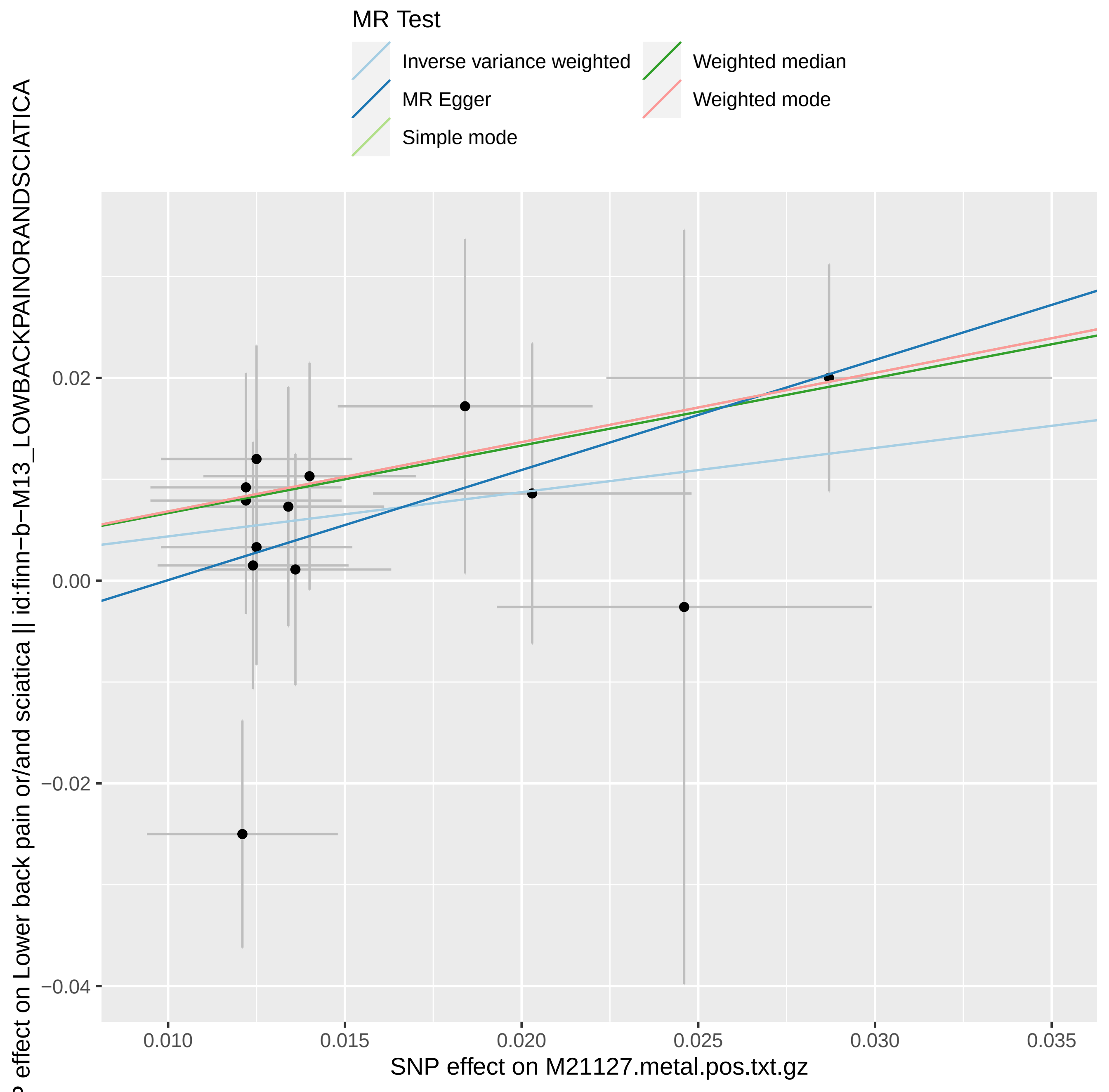

MR Test
Inverse variance weighted
MR Egger
Simple mode
Weighted median
Weighted mode
SNP effect on Lower back pain or/and sciatica || id:finn-b-M13_LOWBACKPAINORANDSCIATICA
0.02
0.00
−0.02
−0.04
0.010
0.015
0.020
0.025
0.030
0.035
SNP effect on M21127.metal.pos.txt.gz

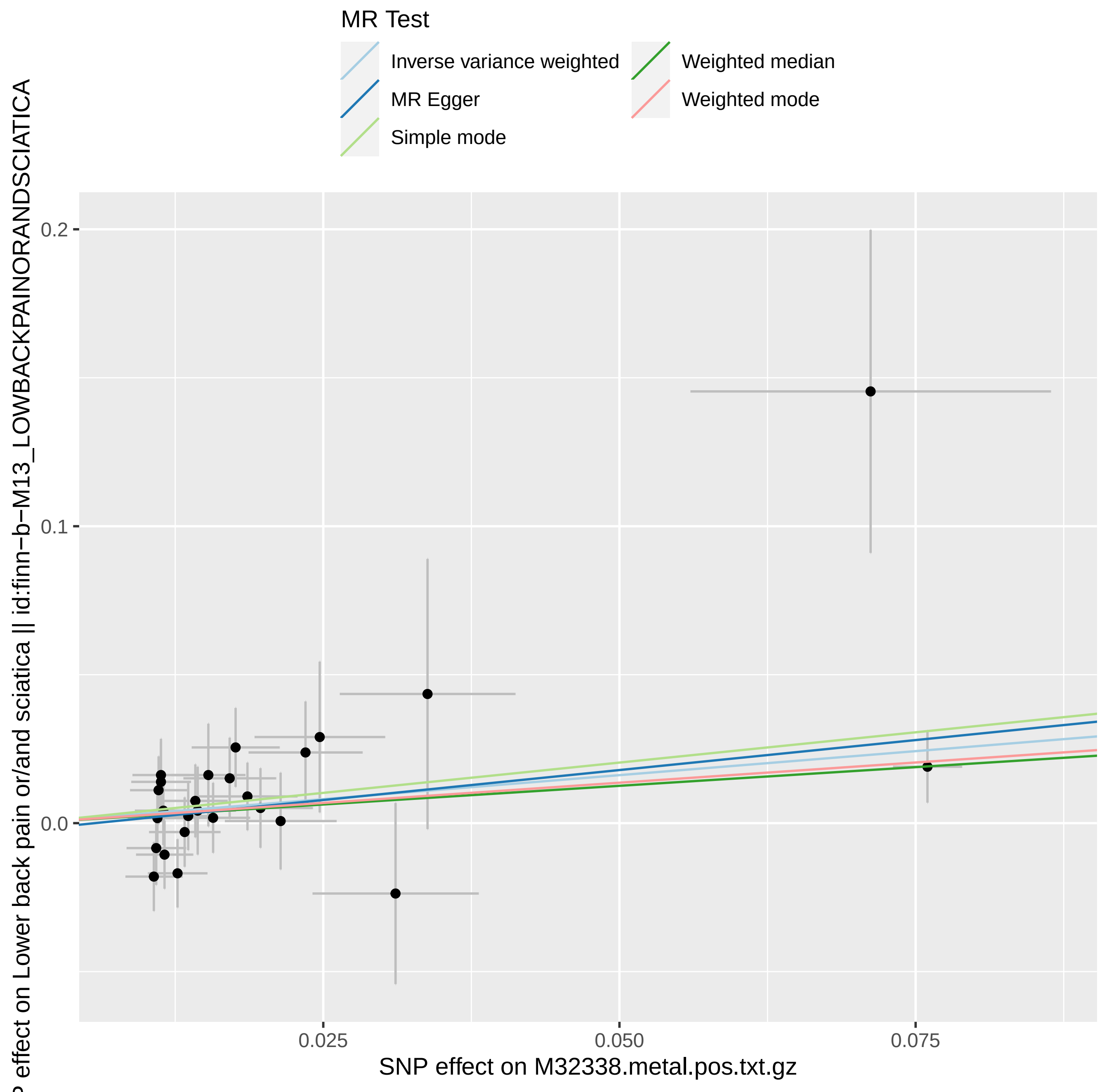

MR Test
Inverse variance weighted
MR Egger
Simple mode
Weighted median
Weighted mode
0.2
0.1
0.0
0.025
0.050
0.075
SNP effect on M32338.metal.pos.txt.gz
effect on Lower back pain or/and sciatica || id:finn-b-M13_LOWBACKPAINORANDSCIATICA

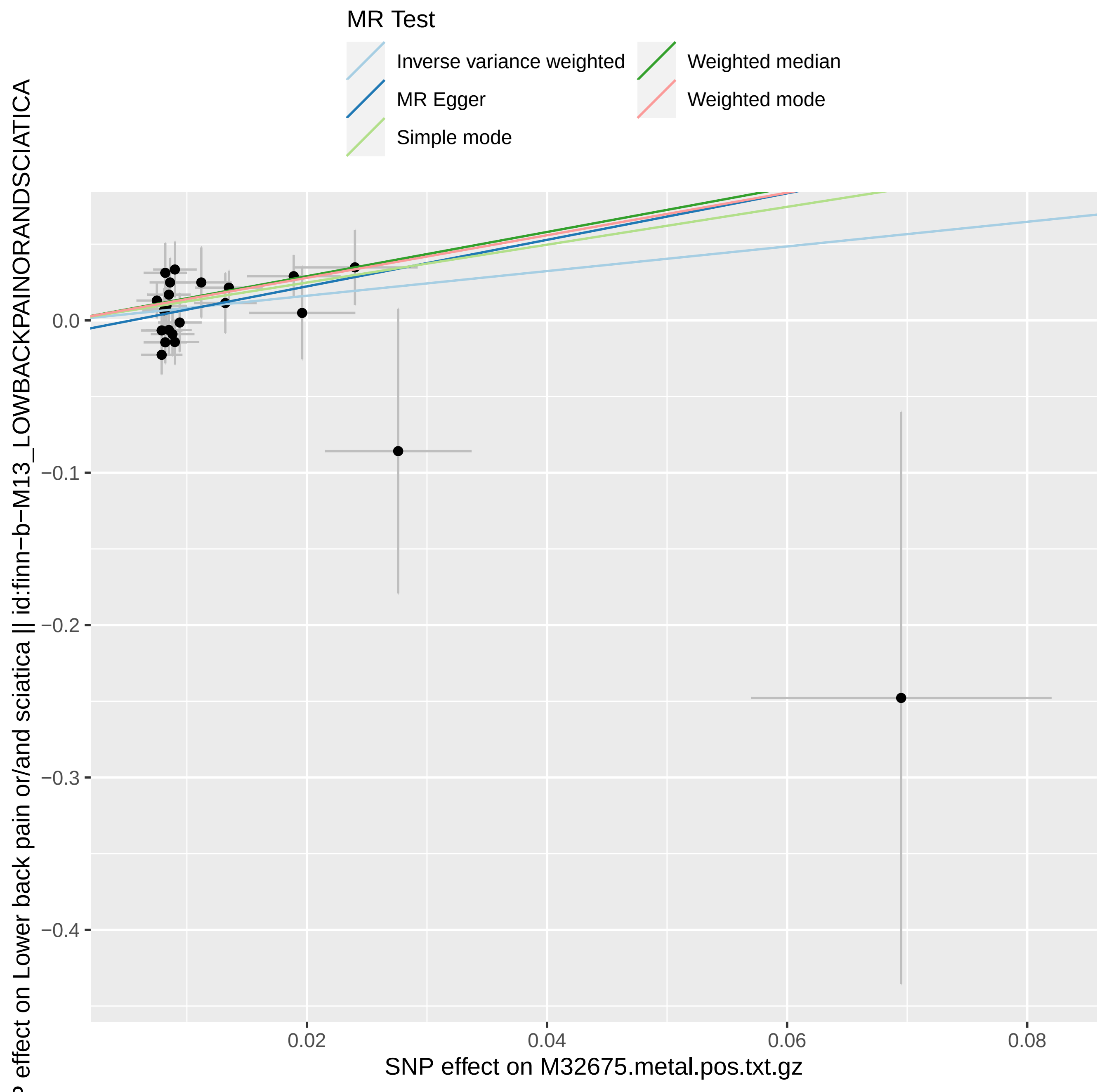

MR Test
Inverse variance weighted
MR Egger
Simple mode
Weighted median
Weighted mode
0.0
−0.1
−0.2
−0.3
−0.4
0.02
0.04
0.06
0.08
SNP effect on M32675.metal.pos.txt.gz
P effect on Lower back pain or/and sciatica || id:finn−b−M13_LOWBACKPAINORANDSCIATICA

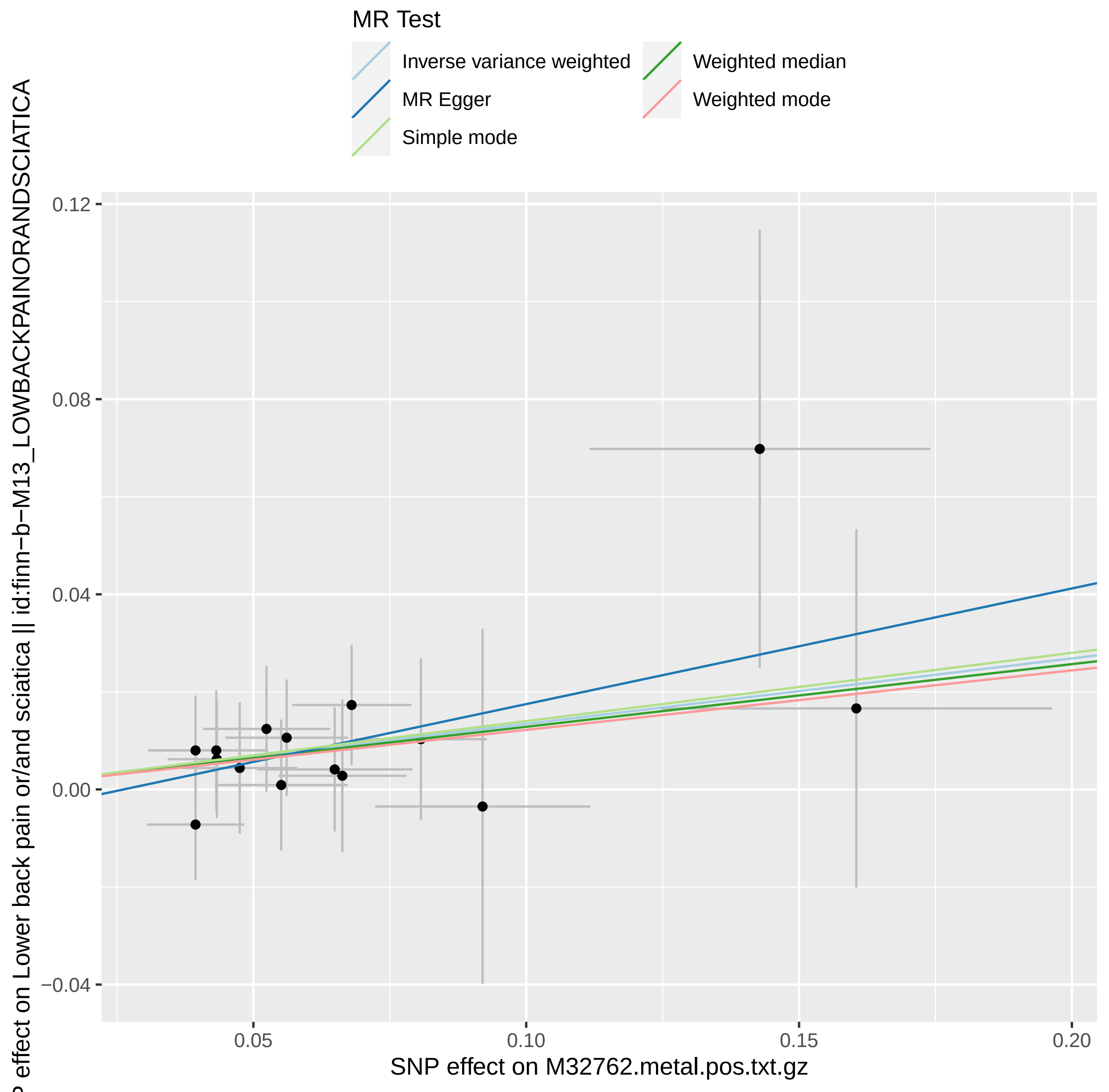
MR Test
Inverse variance weighted
MR Egger
Simple mode
Weighted median
Weighted mode
0.12
0.08
0.04
0.00
−0.04
0.05
0.10
0.15
0.20
SNP effect on M32762.metal.pos.txt.gz
effect on Lower back pain or/and sciatica || id:finn-b-M13_LOWBACKPAINORANDSCIATICA

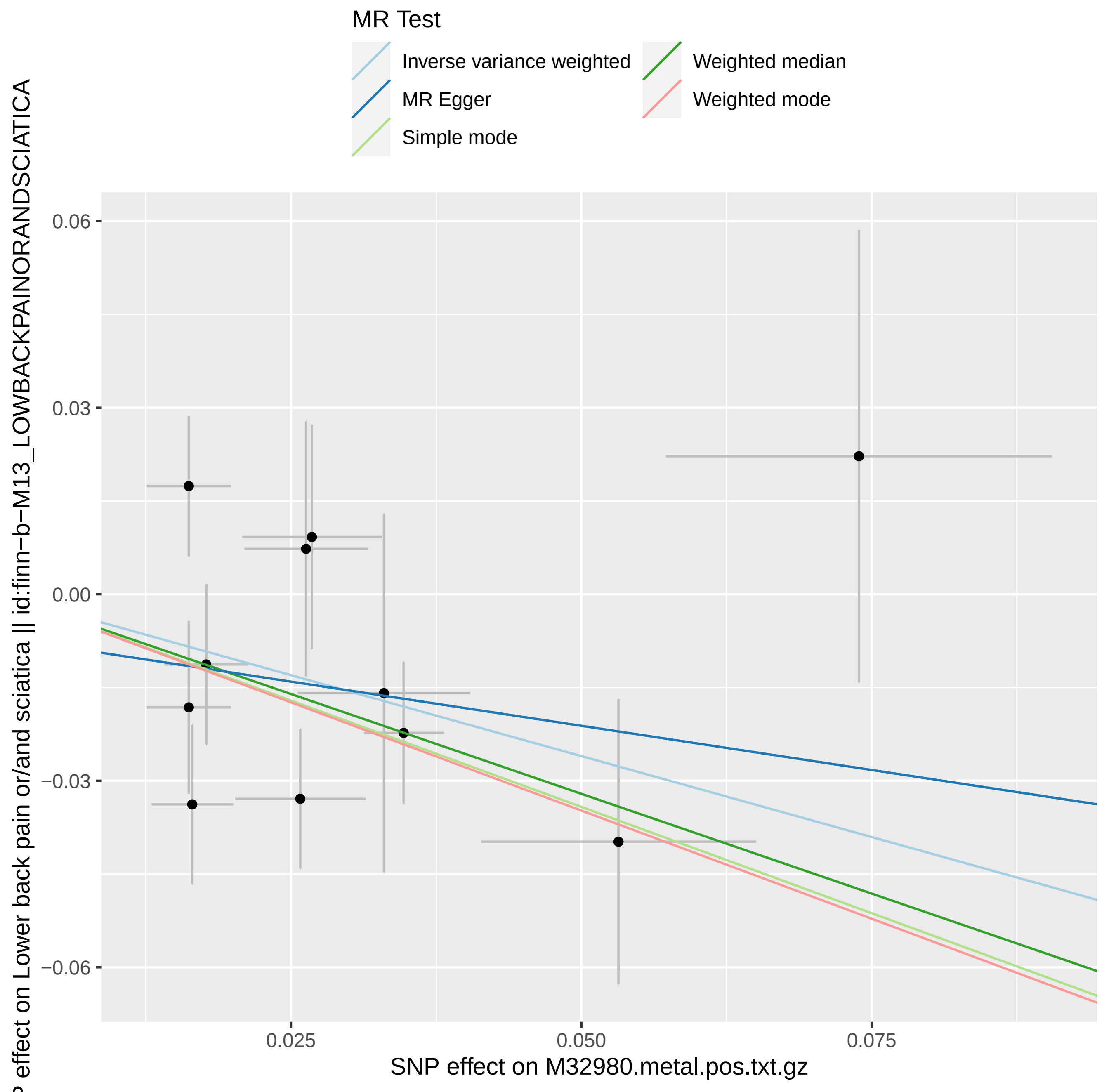

MR Test
Inverse variance weighted
MR Egger
Simple mode
Weighted median
Weighted mode
0.06
0.03
0.00
−0.03
−0.06
0.025
0.050
0.075
SNP effect on M32980.metal.pos.txt.gz
SNP effect on Lower back pain or/and sciatica || id:finn−b−M13_LOWBACKPAINORANDSCIATICA

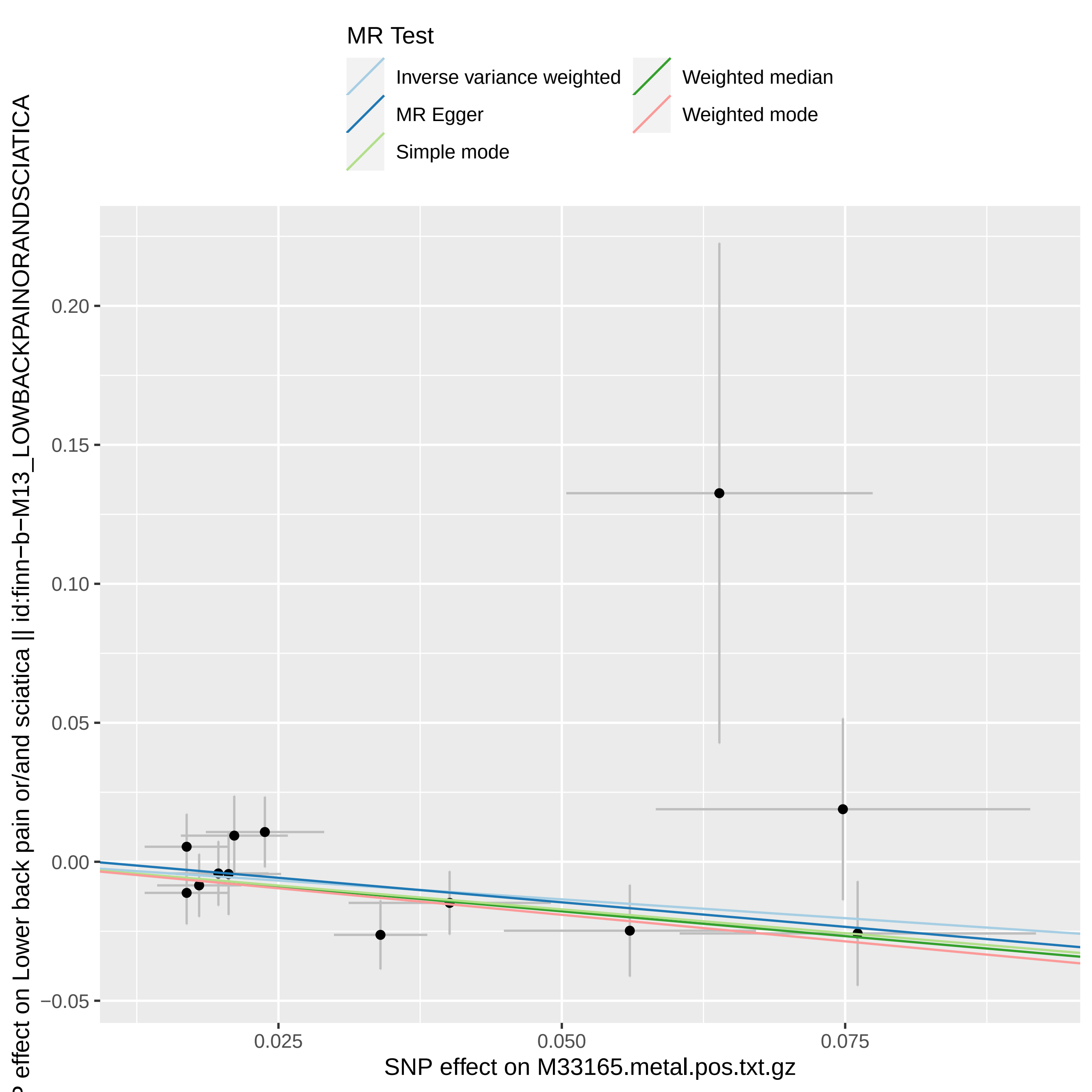
MR Test
Inverse variance weighted
MR Egger
Simple mode
Weighted median
Weighted mode
0.20
0.15
0.10
0.05
0.00
−0.05
0.025
0.050
0.075
SNP effect on M33165.metal.pos.txt.gz
SNP effect on Lower back pain or/and sciatica || id:finn−b−M13_LOWBACKPAINORANDSCIATICA

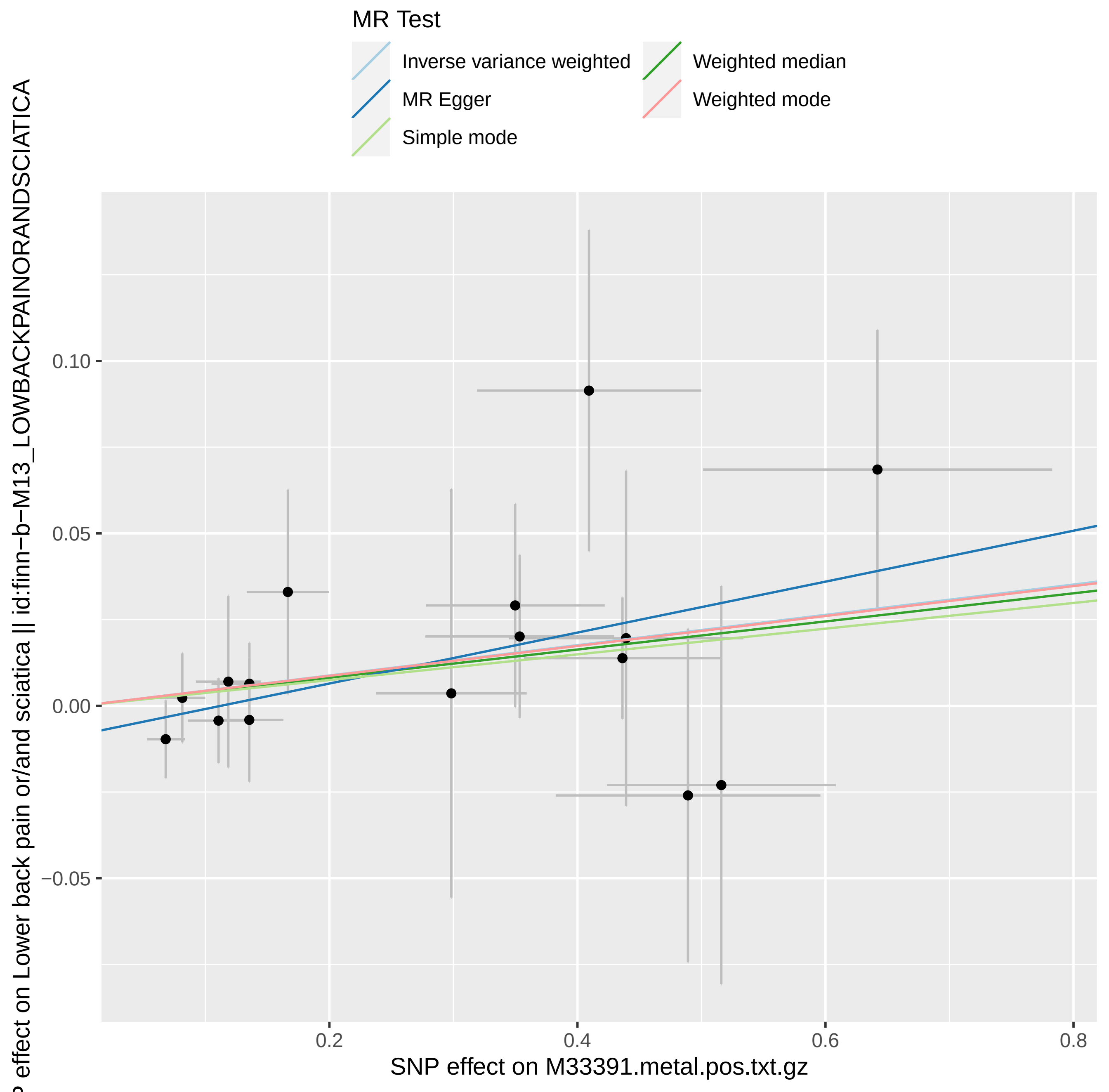
MR Test
Inverse variance weighted
MR Egger
Simple mode
Weighted median
Weighted mode
0.10
0.05
0.00
−0.05
0.2
0.4
0.6
0.8
SNP effect on M33391.metal.pos.txt.gz
SNP effect on Lower back pain or/and sciatica || id:finn−b−M13_LOWBACKPAINORANDSCIATICA

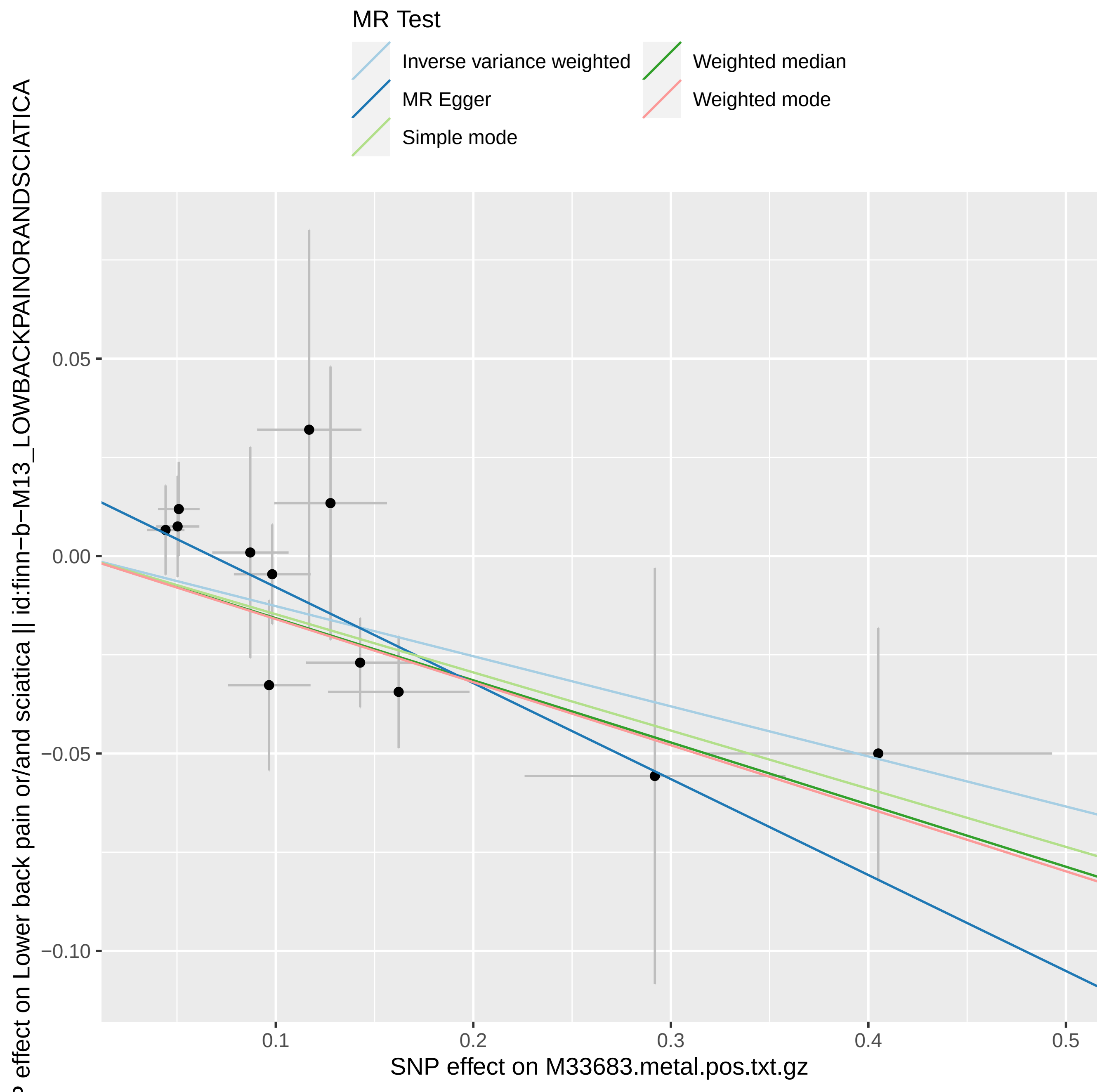
MR Test
Inverse variance weighted
MR Egger
Simple mode
Weighted median
Weighted mode
0.05
0.00
−0.05
−0.10
0.1
0.2
0.3
0.4
0.5
SNP effect on M33683.metal.pos.txt.gz
P effect on Lower back pain or/and sciatica || id:finn−b−M13_LOWBACKPAINORANDSCIATICA

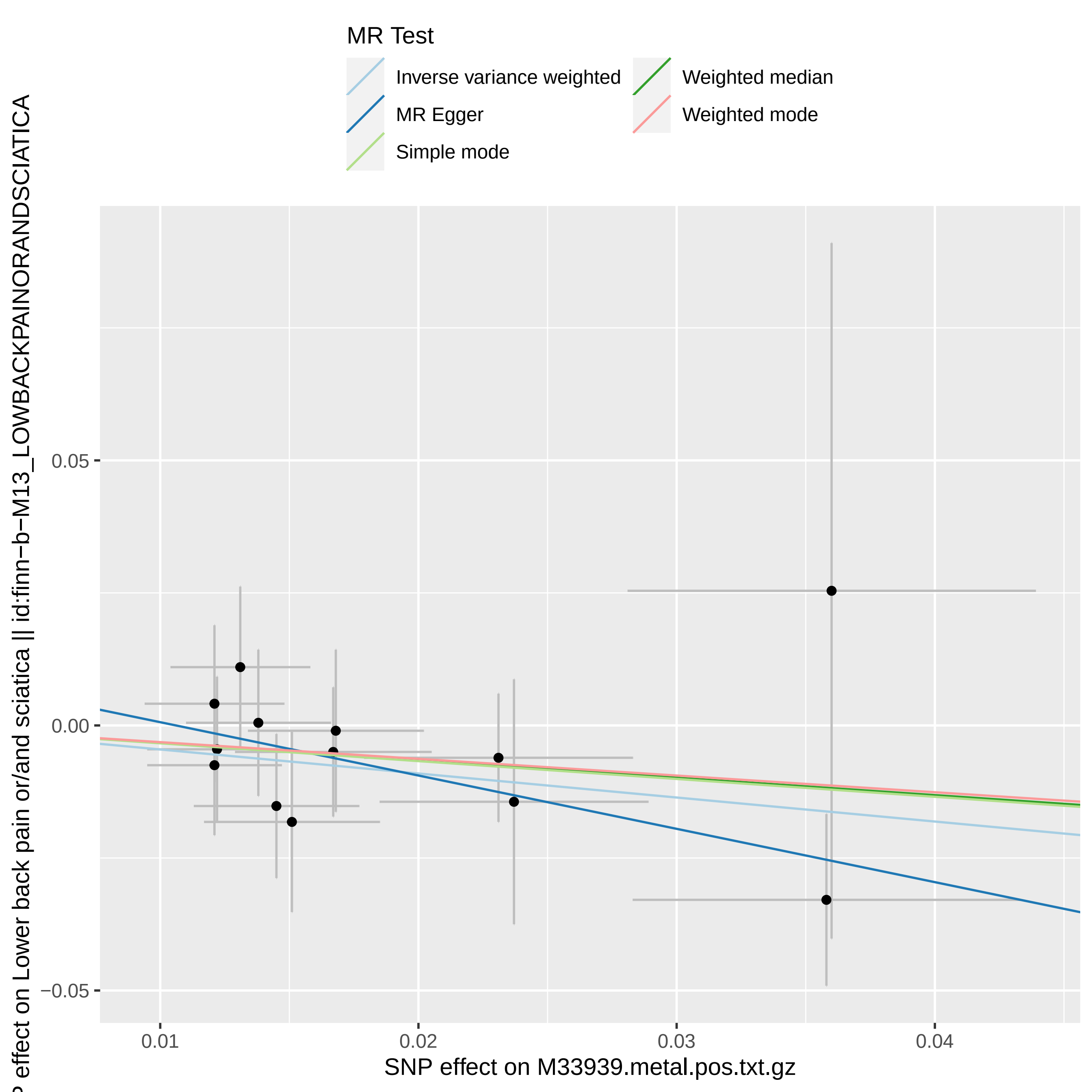

MR Test
Inverse variance weighted
MR Egger
Simple mode
Weighted median
Weighted mode
0.05
0.00
−0.05
0.01
0.02
0.03
0.04
SNP effect on M33939.metal.pos.txt.gz
P effect on Lower back pain or/and sciatica || id:finn−b−M13_LOWBACKPAINORANDSCIATICA

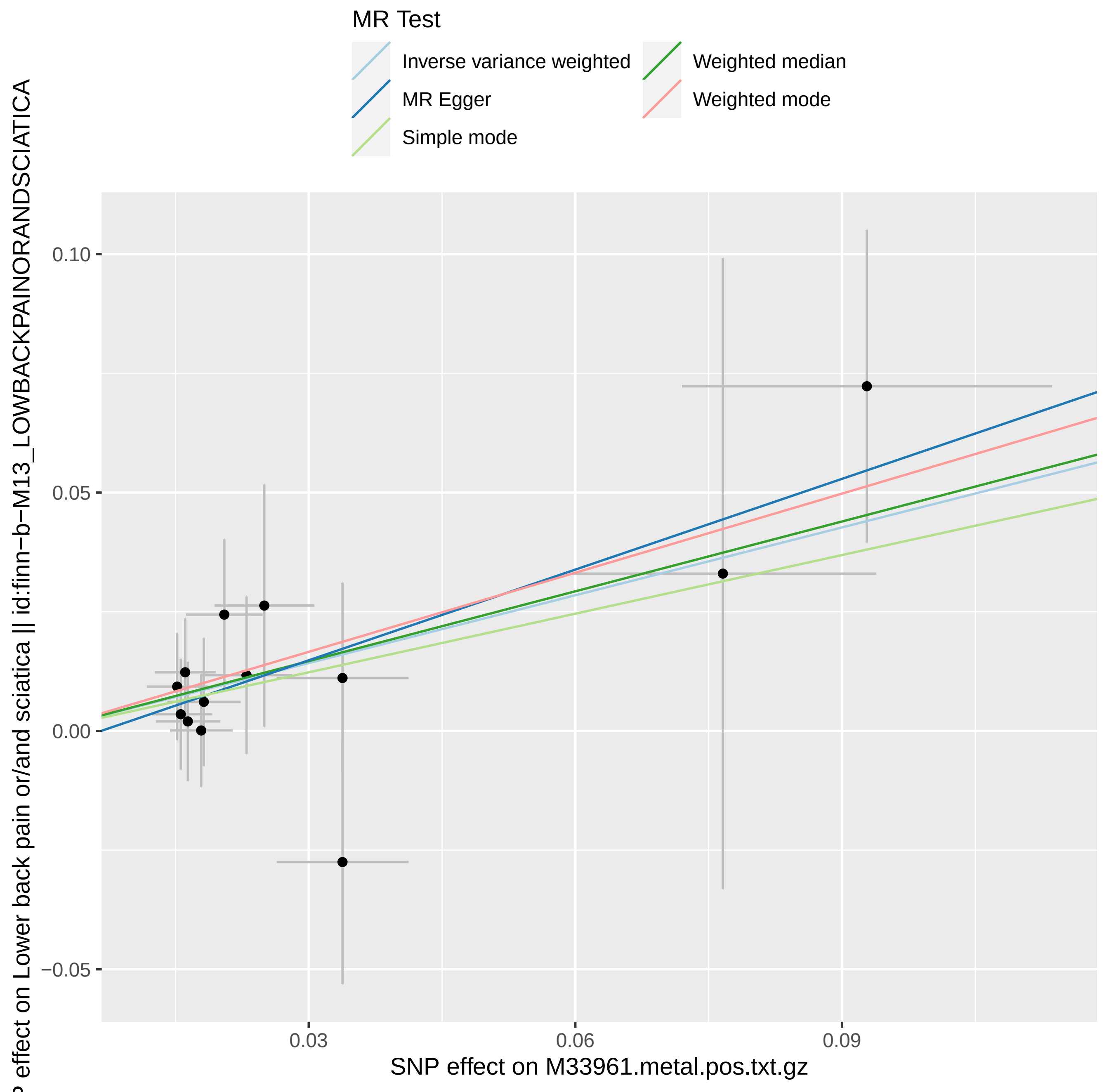

MR Test
Inverse variance weighted
MR Egger
Simple mode
Weighted median
Weighted mode
0.10
0.05
0.00
−0.05
0.03
0.06
0.09
SNP effect on M33961.metal.pos.txt.gz
SNP effect on Lower back pain or/and sciatica || id:finn−b−M13_LOWBACKPAINORANDSCIATICA

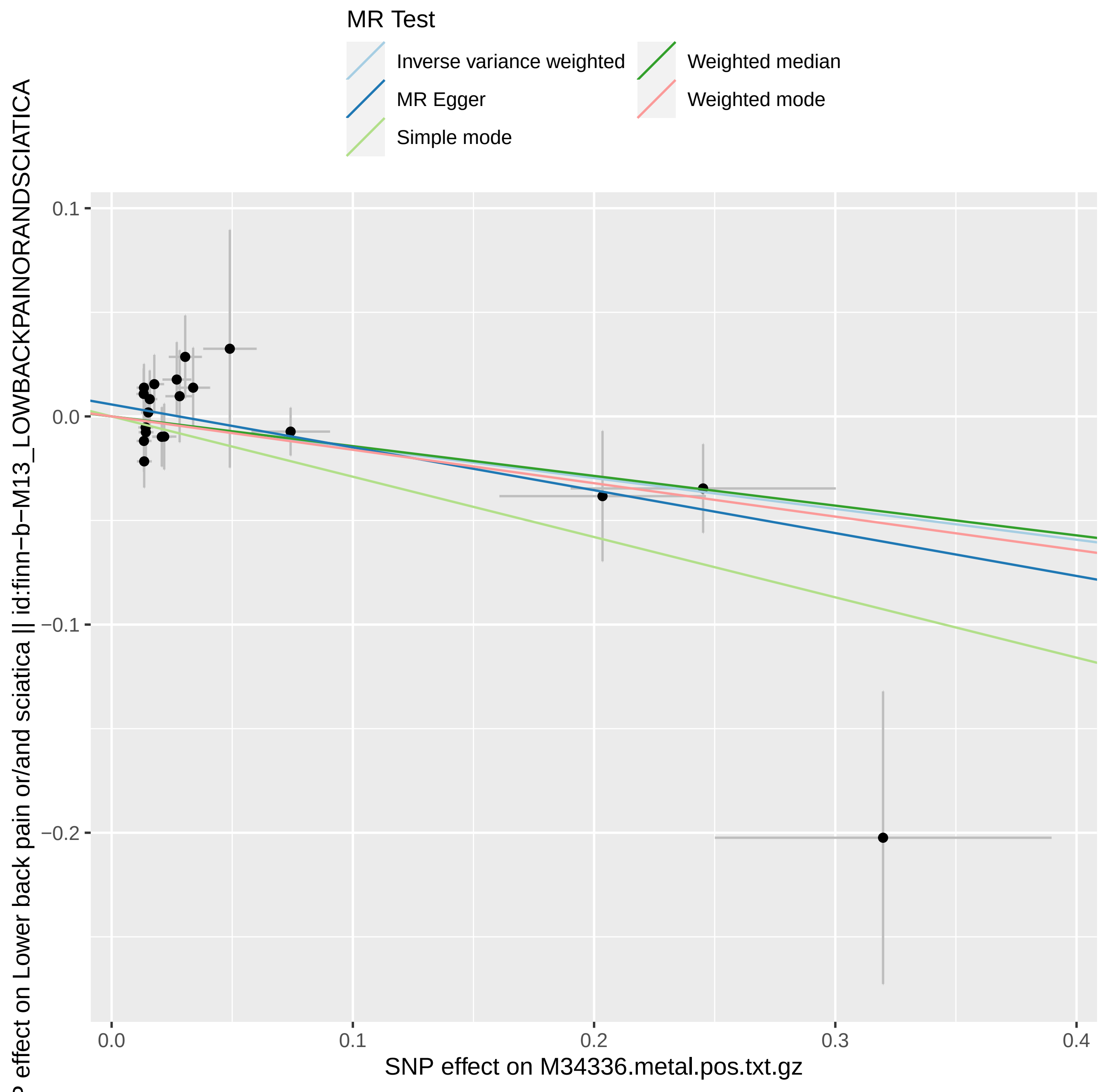

MR Test
Inverse variance weighted
MR Egger
Simple mode
Weighted median
Weighted mode
0.1
0.0
−0.1
−0.2
0.0
0.1
0.2
0.3
0.4
SNP effect on M34336.metal.pos.txt.gz
P effect on Lower back pain or/and sciatica || id:finn−b−M13_LOWBACKPAINORANDSCIATICA

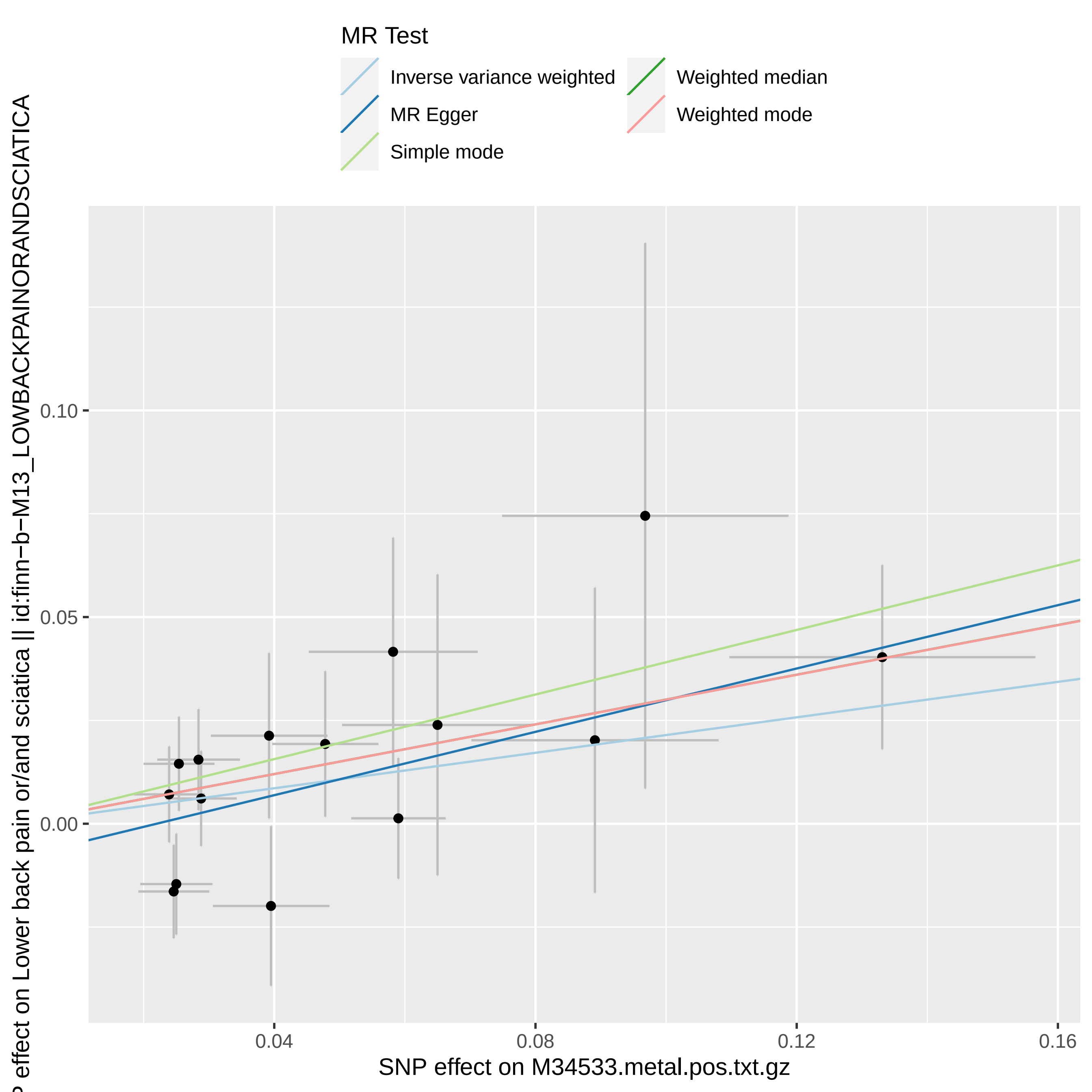
MR Test
Inverse variance weighted
MR Egger
Simple mode
Weighted median
Weighted mode
0.10
0.05
0.00
0.04
0.08
0.12
0.16
SNP effect on M34533.metal.pos.txt.gz
SNP effect on Lower back pain or/and sciatica || id:finn−b−M13_LOWBACKPAINORANDSCIATICA

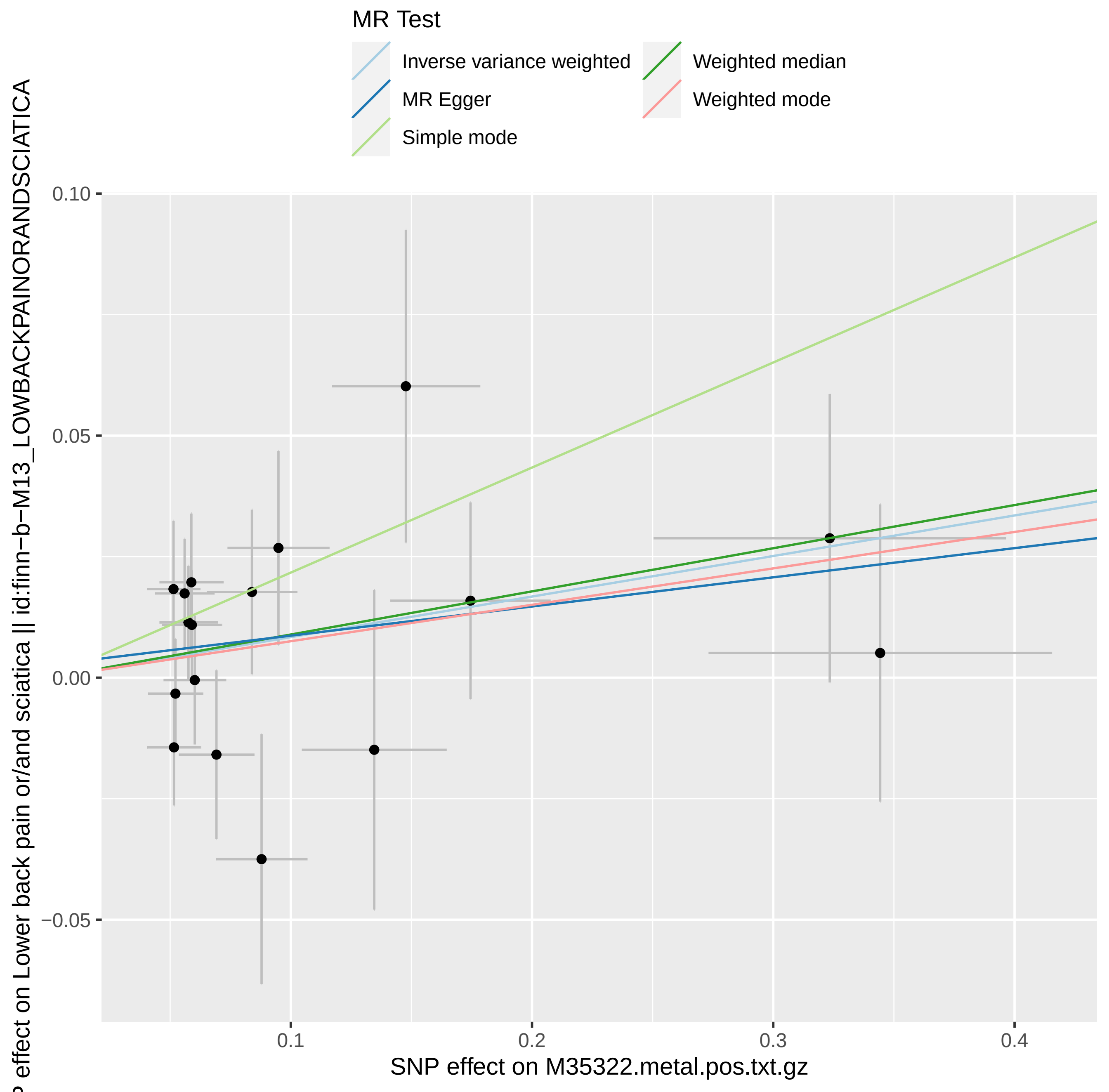

MR Test
Inverse variance weighted
MR Egger
Simple mode
Weighted median
Weighted mode
0.10
0.05
0.00
−0.05
0.1
0.2
0.3
0.4
SNP effect on M35322.metal.pos.txt.gz
P effect on Lower back pain or/and sciatica || id:finn−b−M13_LOWBACKPAINORANDSCIATICA

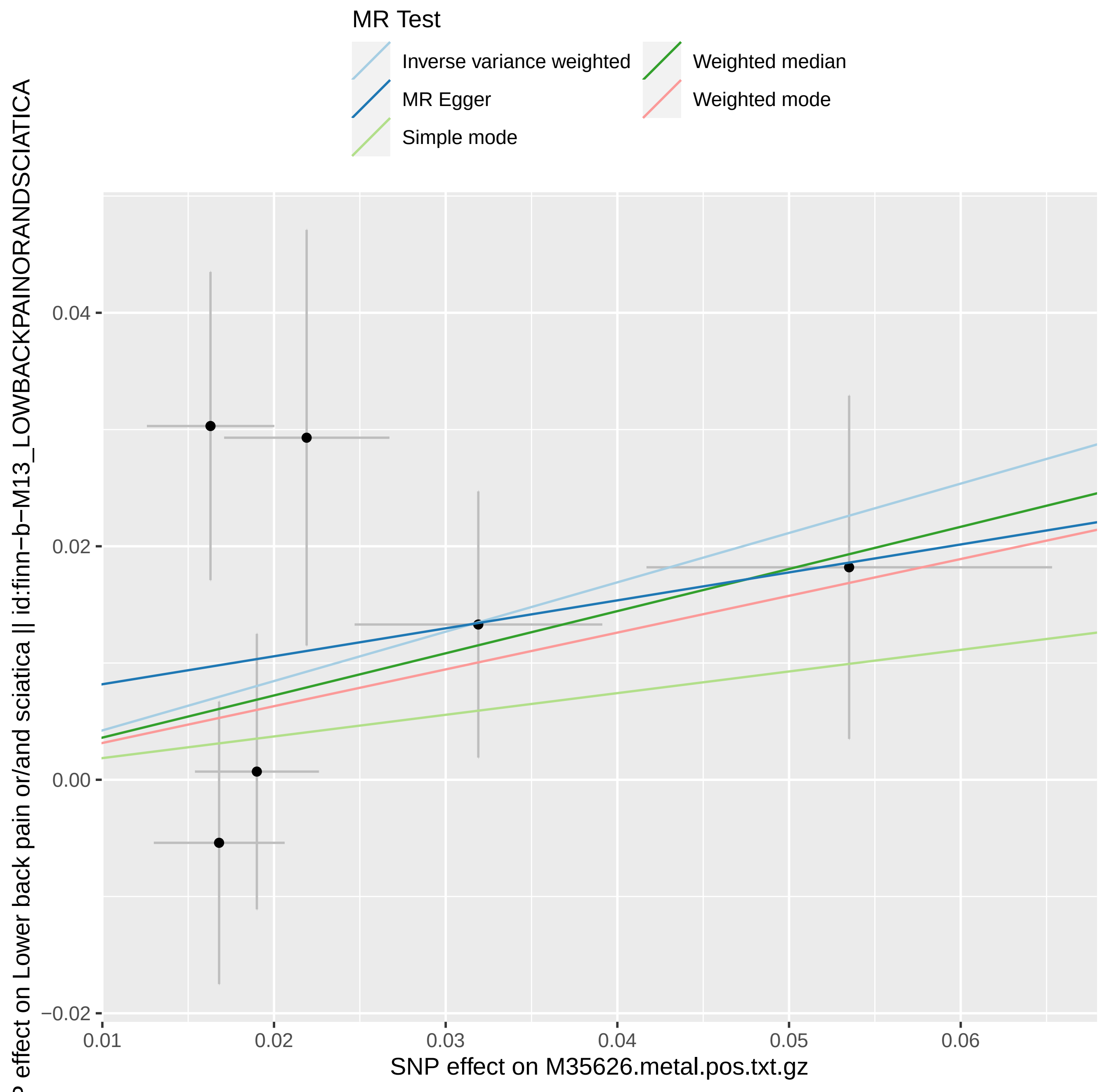

MR Test
Inverse variance weighted
MR Egger
Simple mode
Weighted median
Weighted mode
0.04
0.02
0.00
−0.02
0.01
0.02
0.03
0.04
0.05
0.06
SNP effect on M35626.metal.pos.txt.gz
SNP effect on Lower back pain or/and sciatica || id:finn−b−M13_LOWBACKPAINORANDSCIATICA

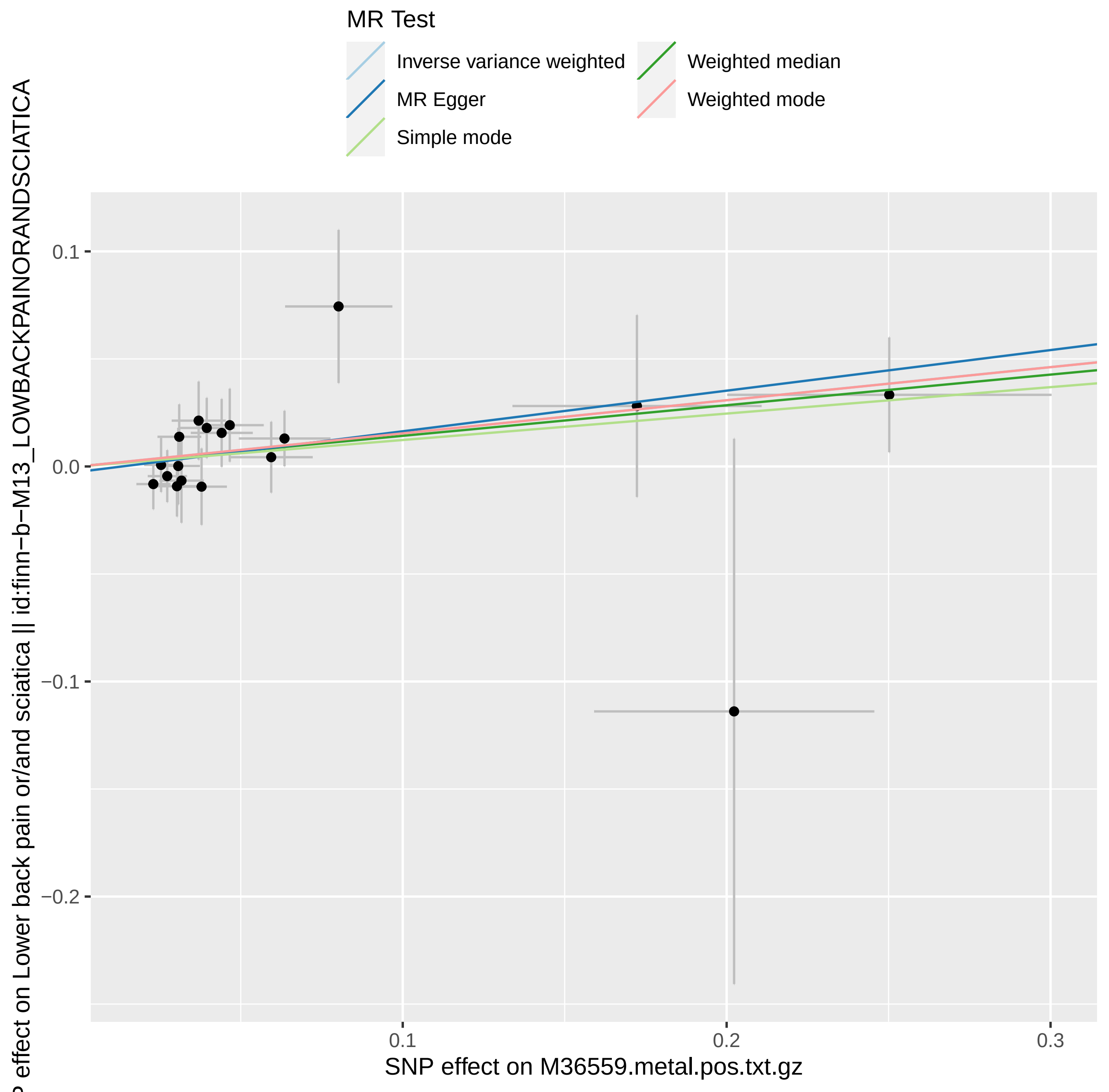
MR Test
Inverse variance weighted
MR Egger
Simple mode
Weighted median
Weighted mode
0.1
0.0
−0.1
−0.2
0.1
0.2
0.3
SNP effect on M36559.metal.pos.txt.gz
P effect on Lower back pain or/and sciatica || id:finn−b−M13_LOWBACKPAINORANDSCIATICA
