# Supplementary material for: Causality of genetically determined serum metabolites on lower back pain or/and sciatica: a comprehensive Mendelian randomized study
Source: Front Pain Res (Lausanne). 2024 Sep 25;5:1370704. doi: 10.3389/fpain.2024.1370704 (PMC11461461; doi:10.3389/fpain.2024.1370704)
Supplement: Supplementary Figure S2 — All rest funnel plot maps of 28 metabolites with potential causal relationship with sciatica or/and lower back pain. [file Datasheet5.pdf]

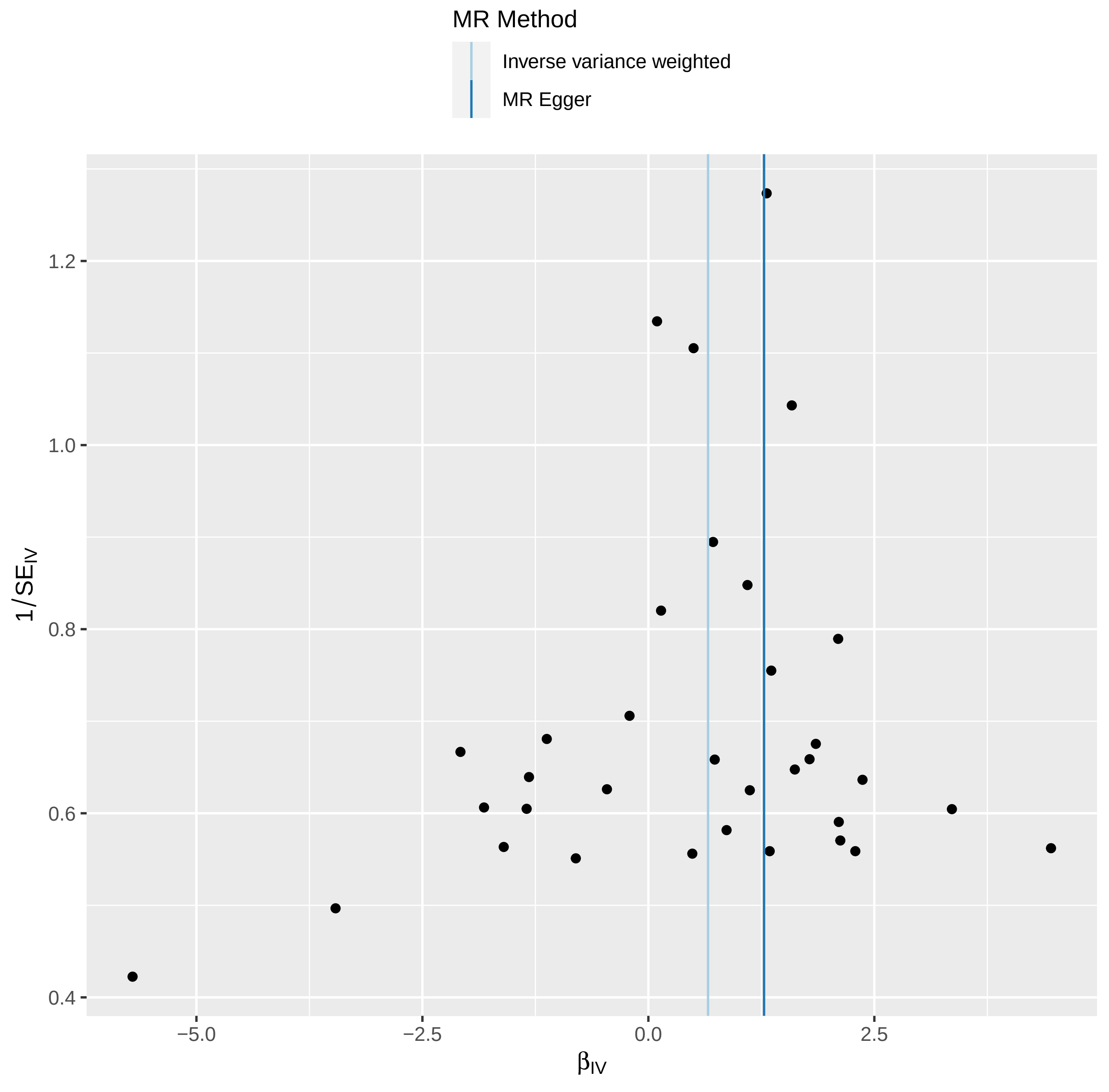
MR Method
Inverse variance weighted
MR Egger
1/SE_{IV}
1.2
1.0
0.8
0.6
0.4
−5.0
−2.5
0.0
2.5
β_{IV}

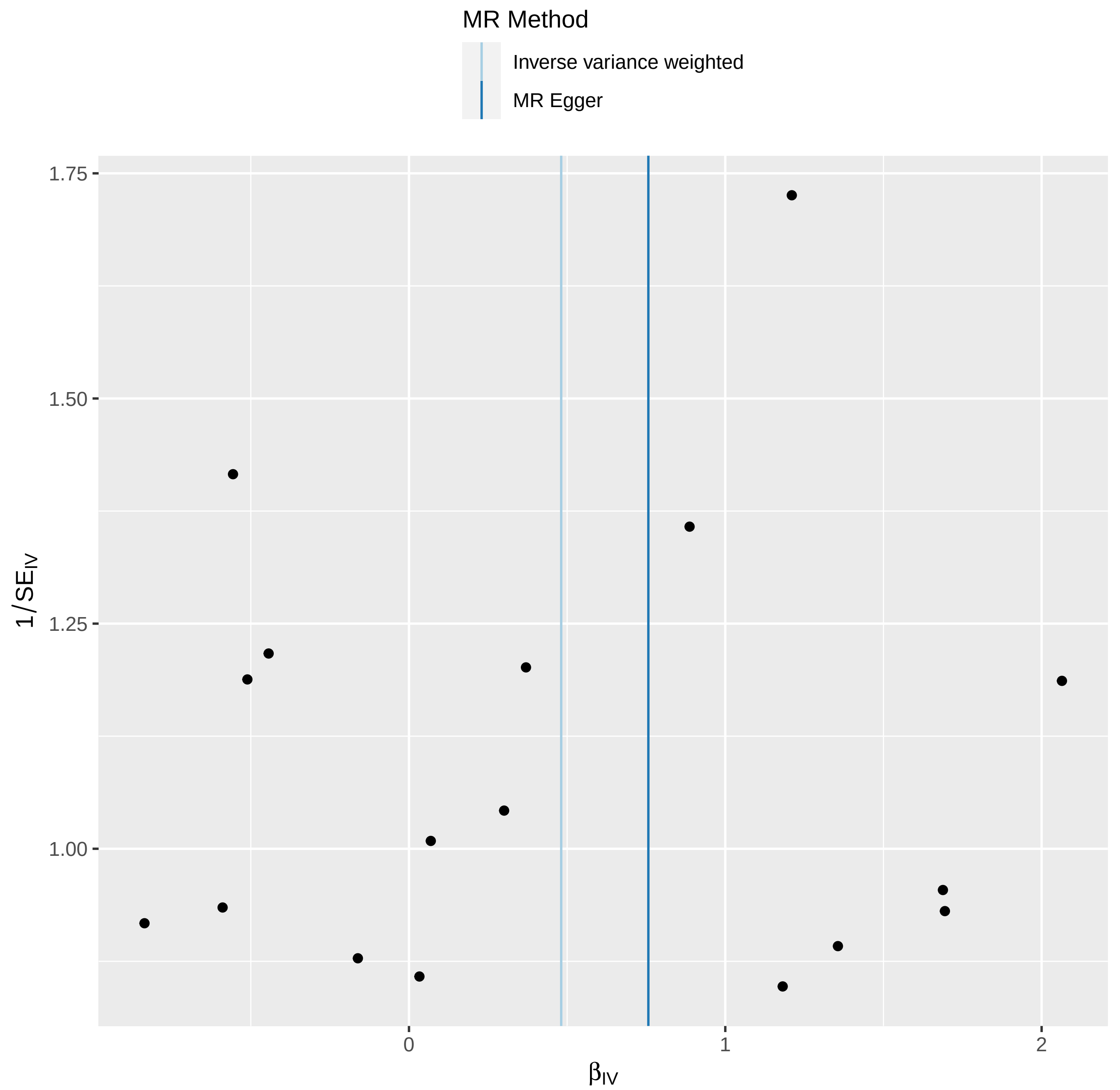
MR Method
Inverse variance weighted
MR Egger
1.75
1.50
1.25
1.00
$1/SE_{IV}$
0
1
2
β_{IV}

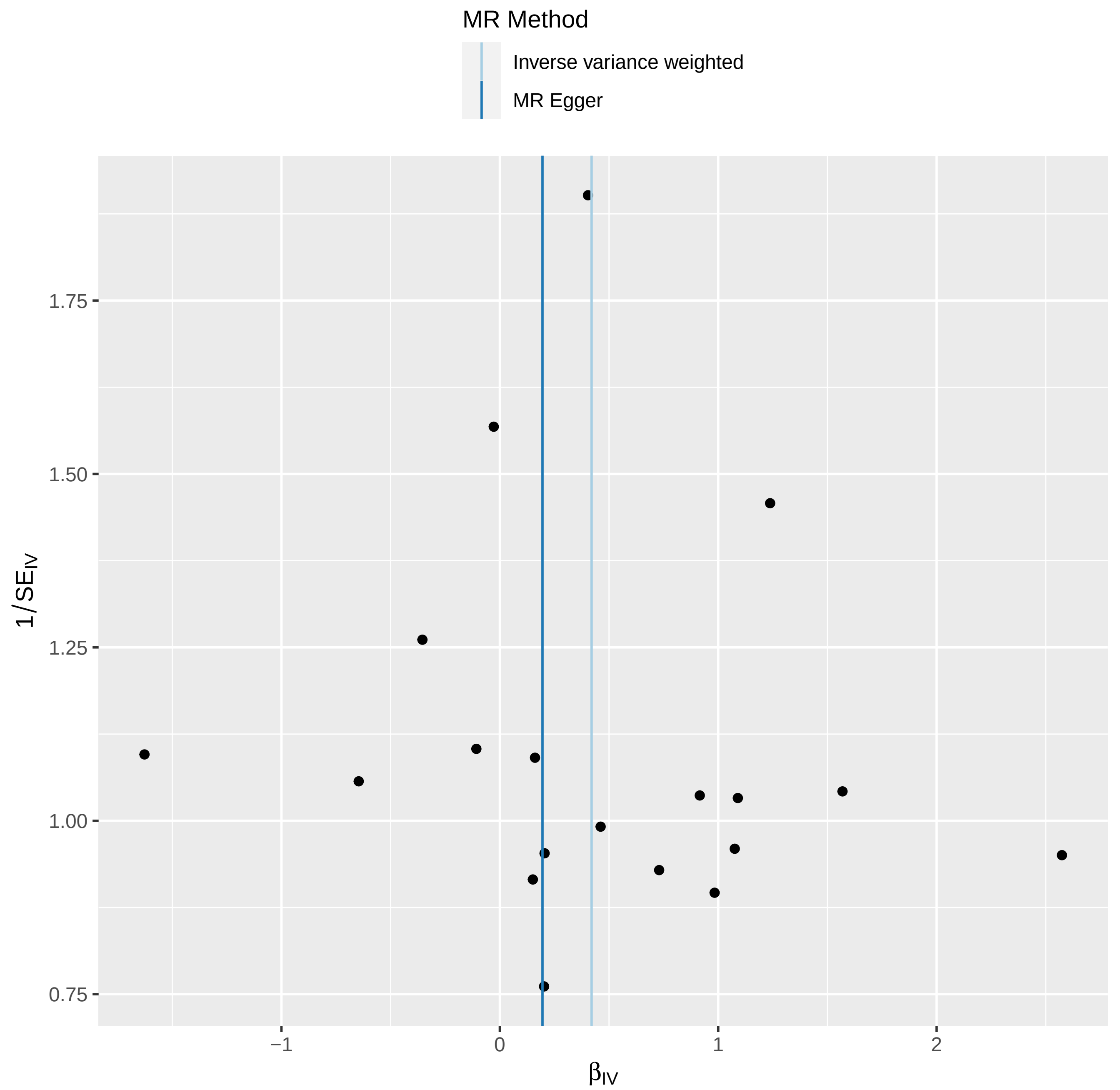

MR Method
Inverse variance weighted
MR Egger
1.75
1.50
1.25
1.00
0.75
1/SE_IV
−1
0
1
2
β_IV

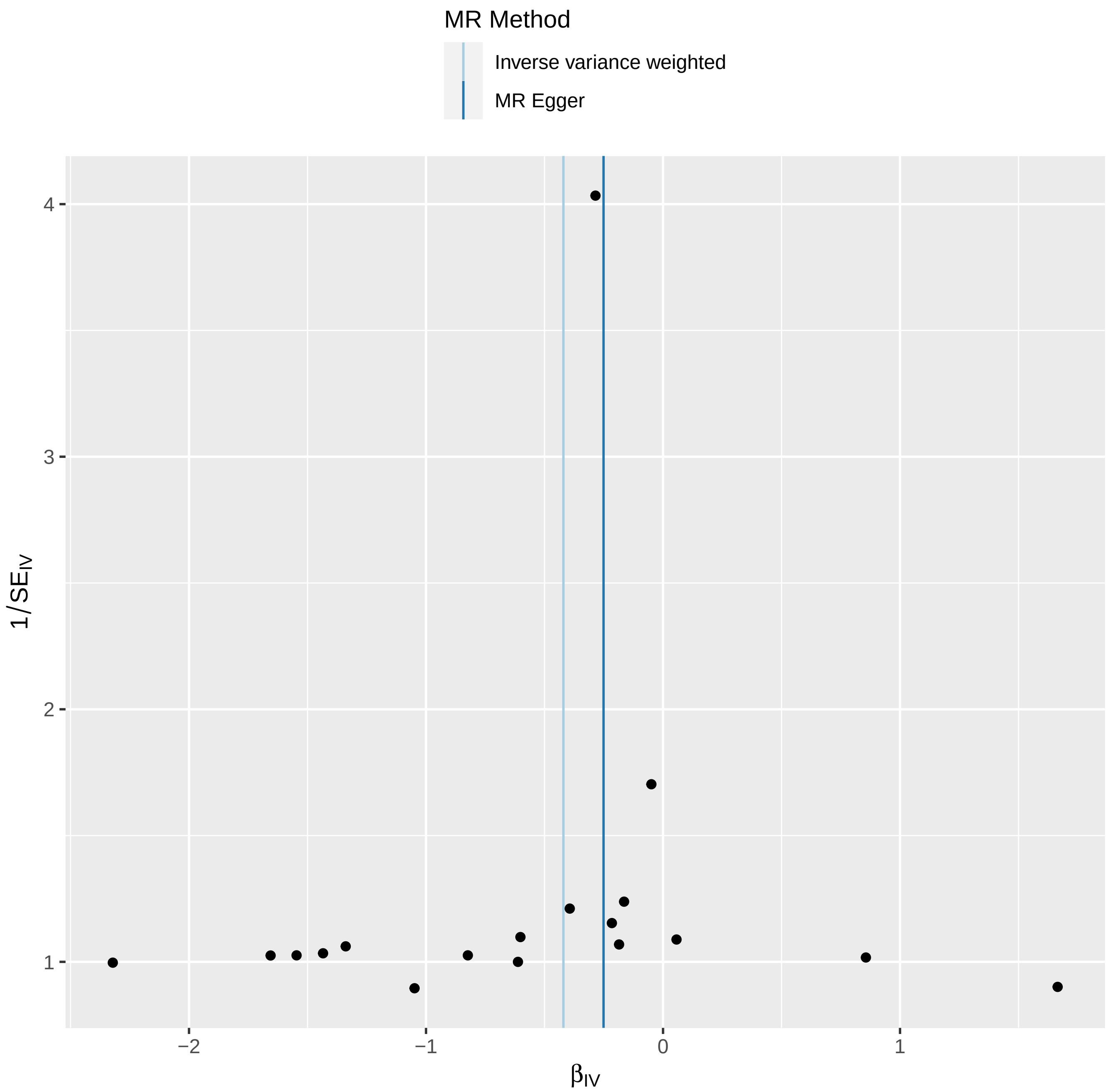
MR Method
Inverse variance weighted
MR Egger
4
3
2
1
−2
−1
0
1
β_{IV}
$1/SE_{IV}$

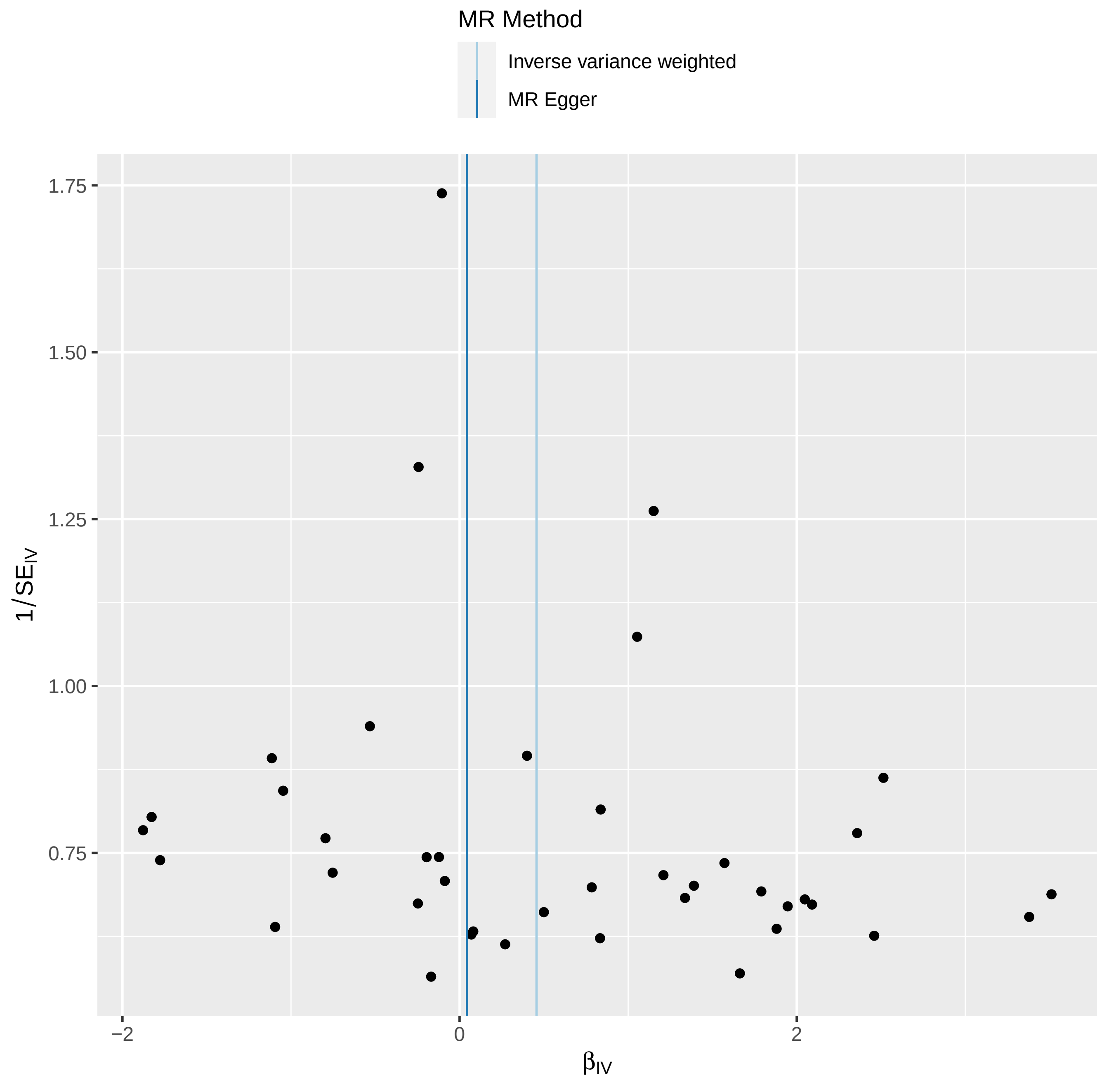

MR Method
Inverse variance weighted
MR Egger
1.75
1.50
1.25
1.00
0.75
1/SE_IV
−2
0
2
β_IV

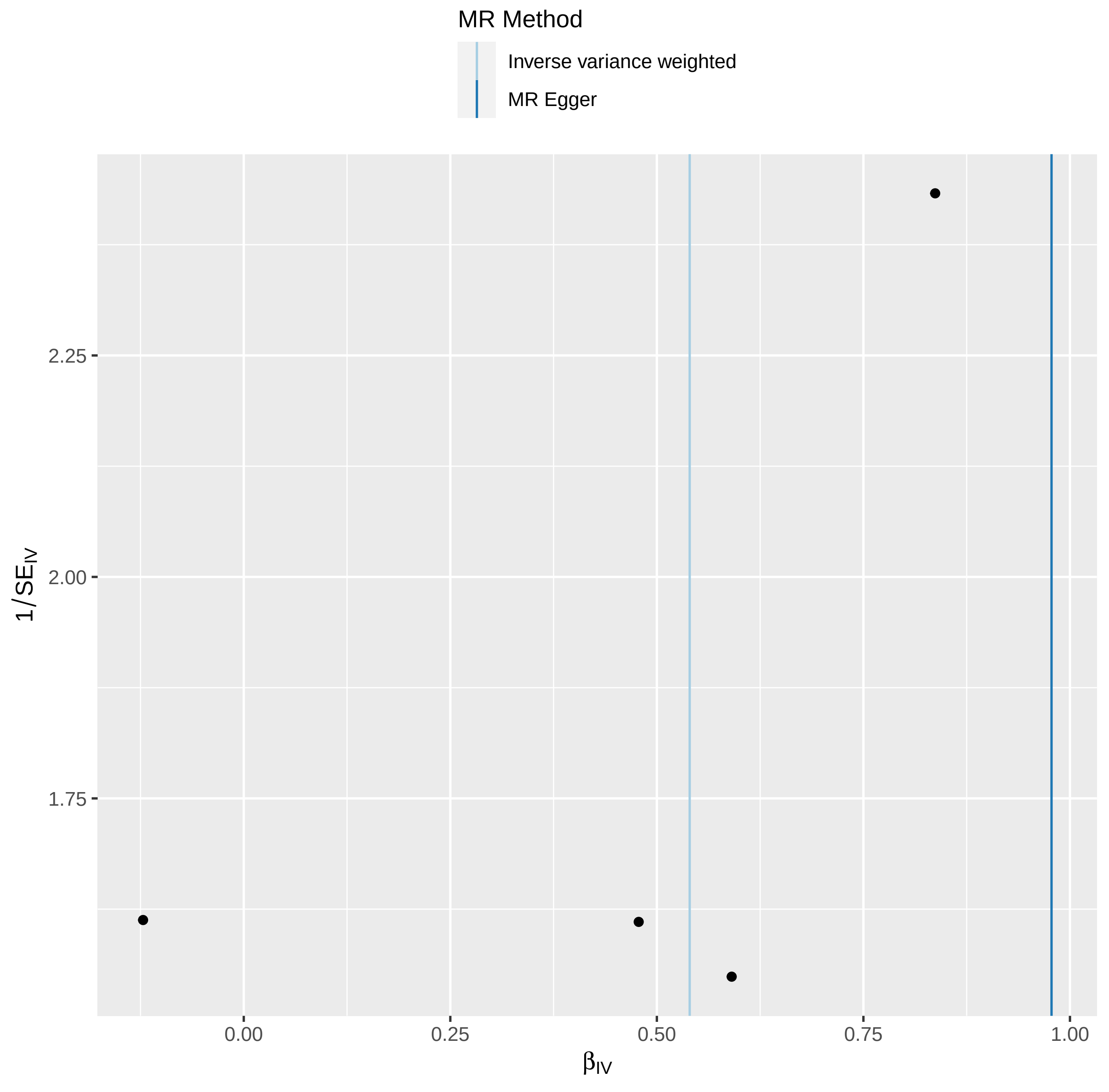

MR Method
Inverse variance weighted
MR Egger
2.25
2.00
1.75
$1/SE_{IV}$
0.00
0.25
0.50
0.75
1.00
β_{IV}

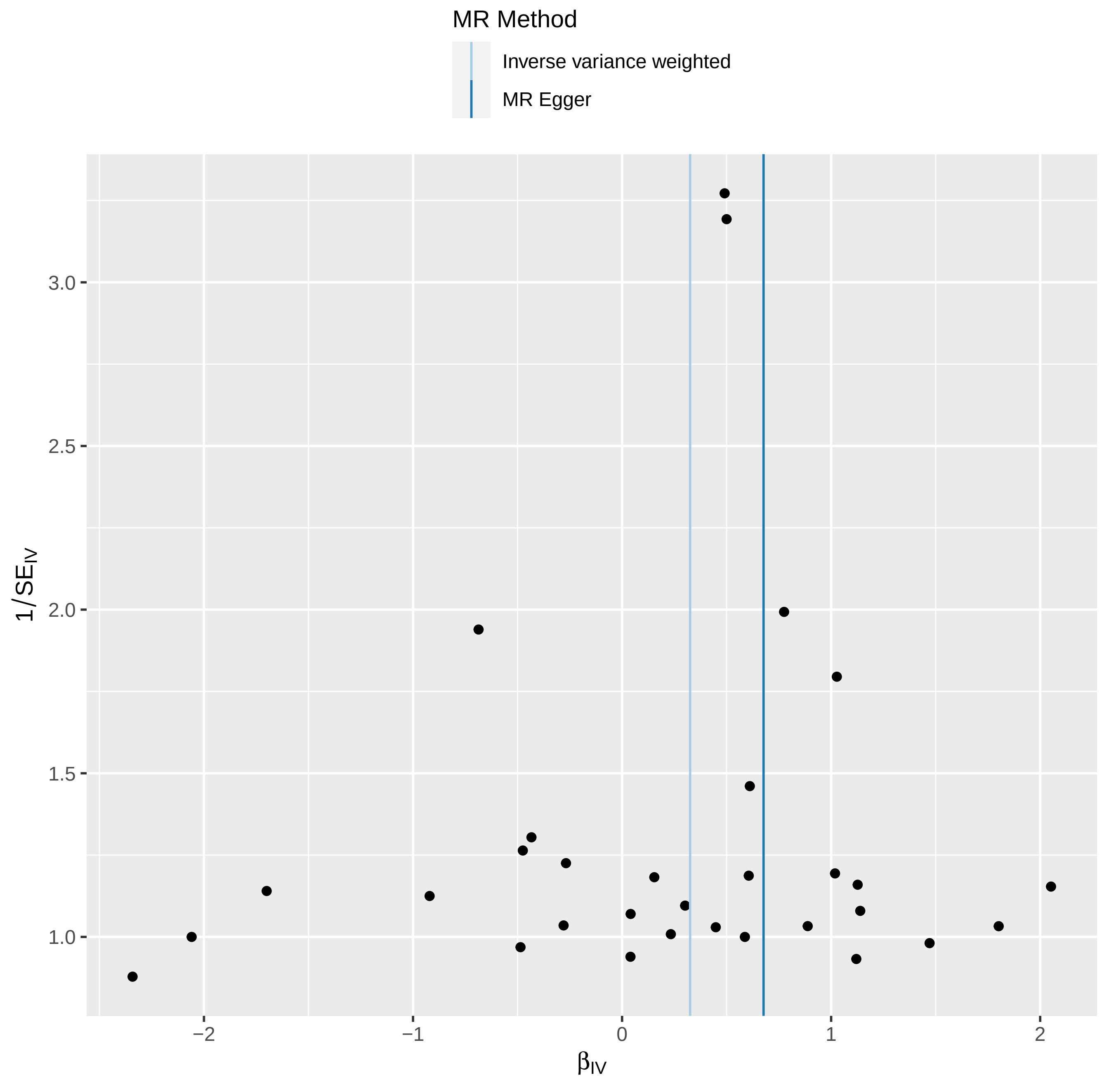
MR Method
Inverse variance weighted
MR Egger
1/SE_IV
β_IV
3.0
2.5
2.0
1.5
1.0
−2
−1
0
1
2

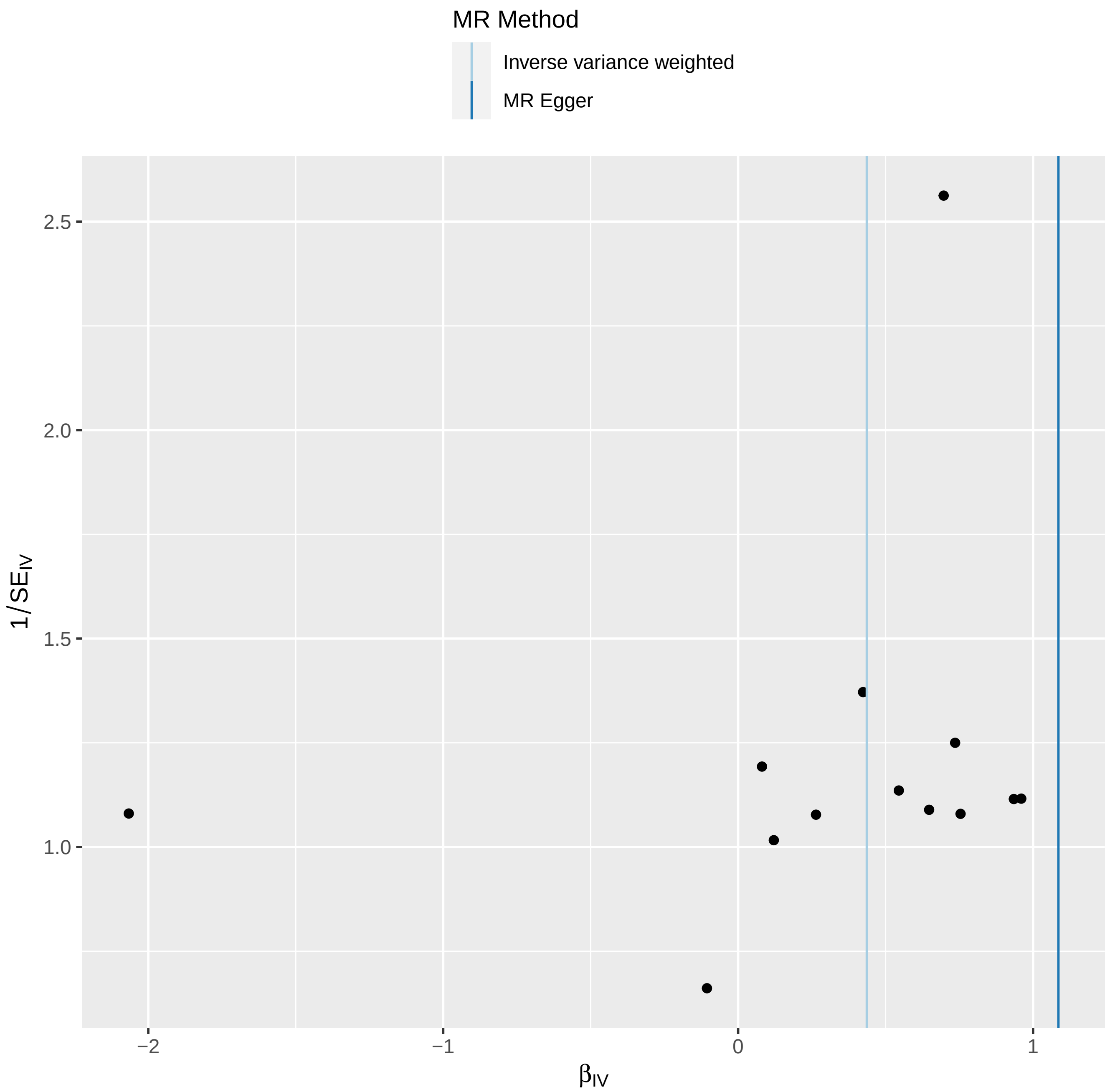

MR Method
Inverse variance weighted
MR Egger
2.5
2.0
1.5
1.0
1/SE_IV
−2
−1
0
1
β_IV

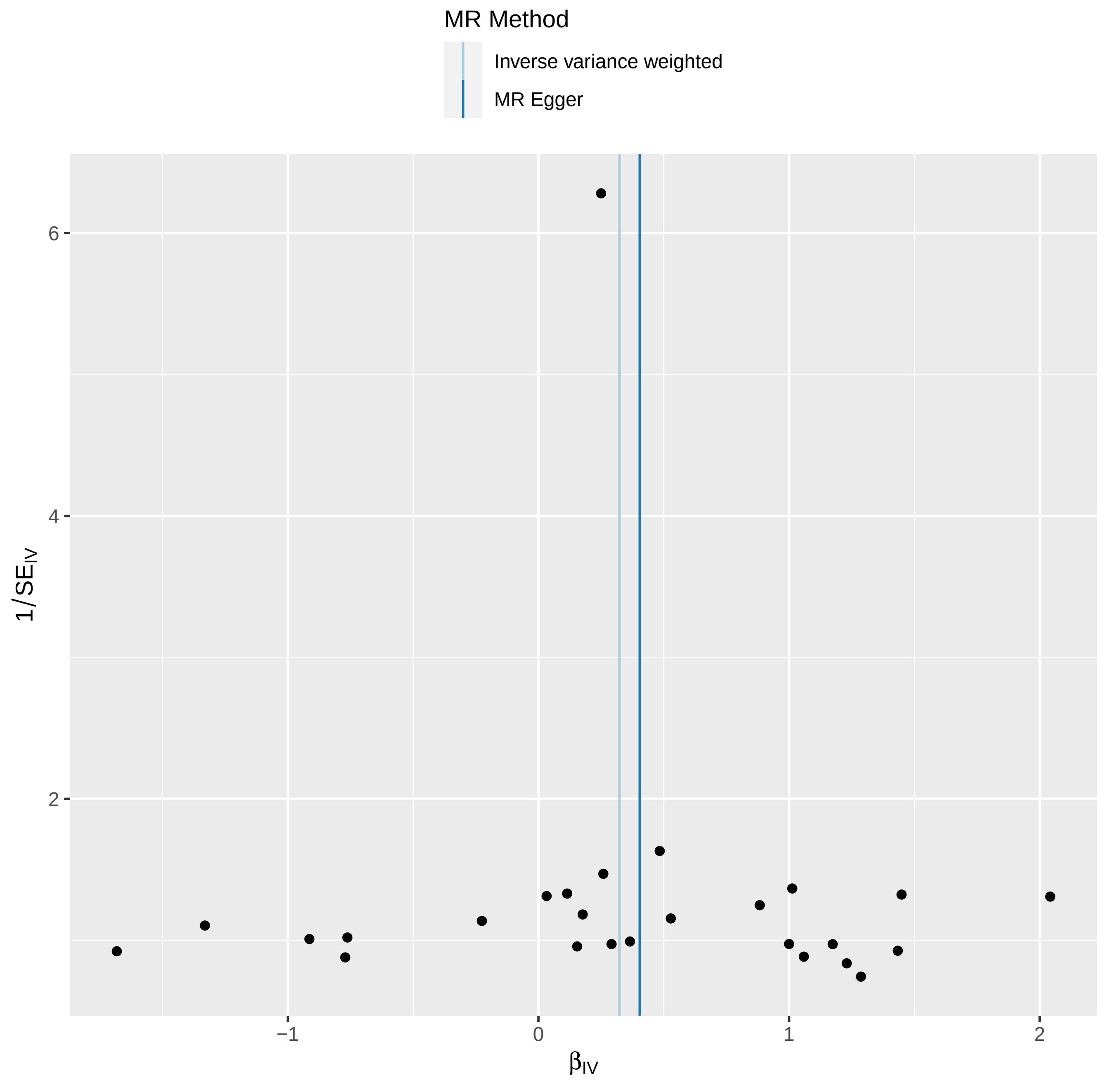
MR Method
Inverse variance weighted
MR Egger
1/SE_IV
6
4
2
−1
0
1
2
β_IV

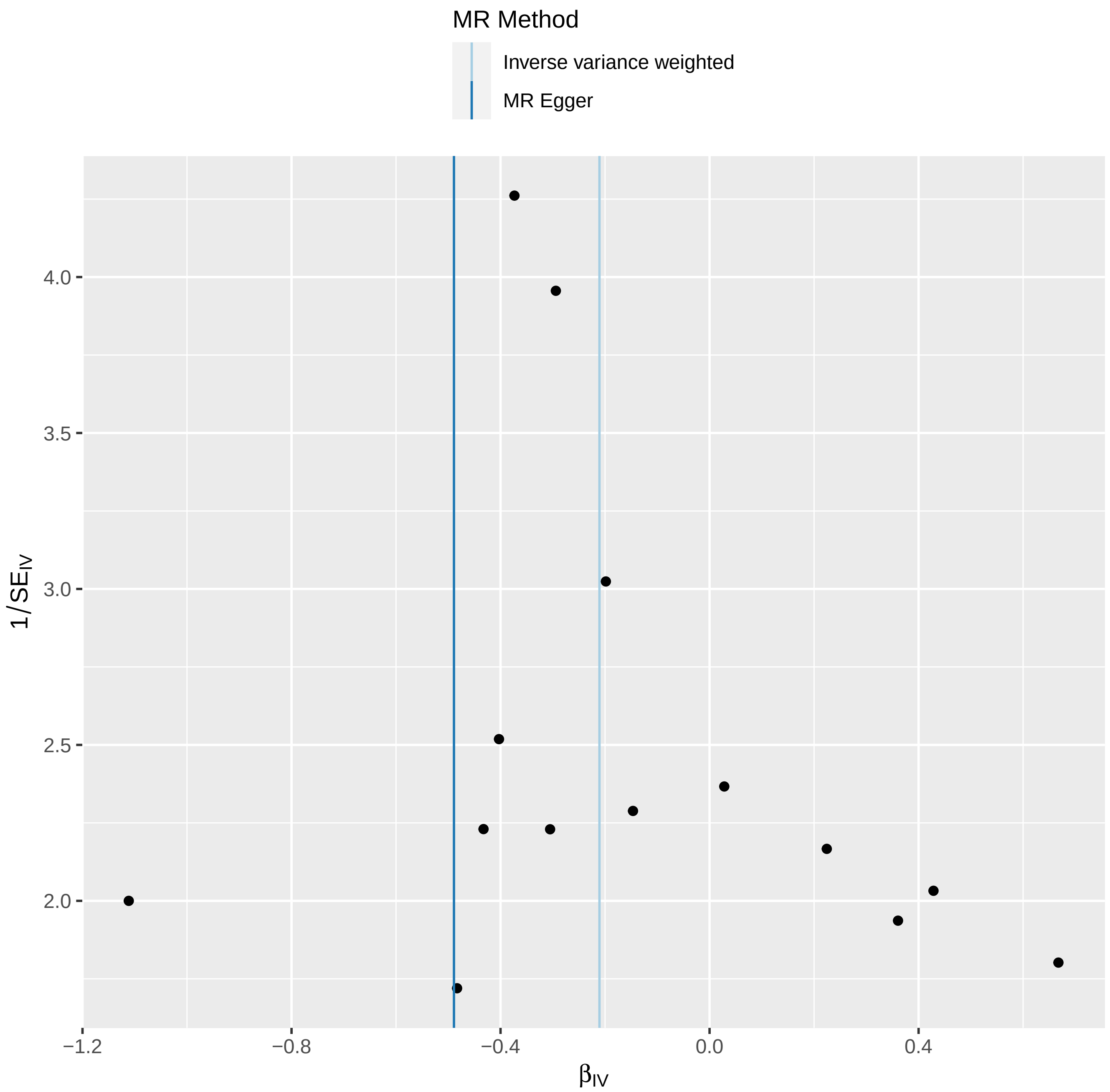
MR Method
Inverse variance weighted
MR Egger
4.0
3.5
3.0
2.5
2.0
1/SE_IV
−1.2
−0.8
−0.4
0.0
0.4
β_IV

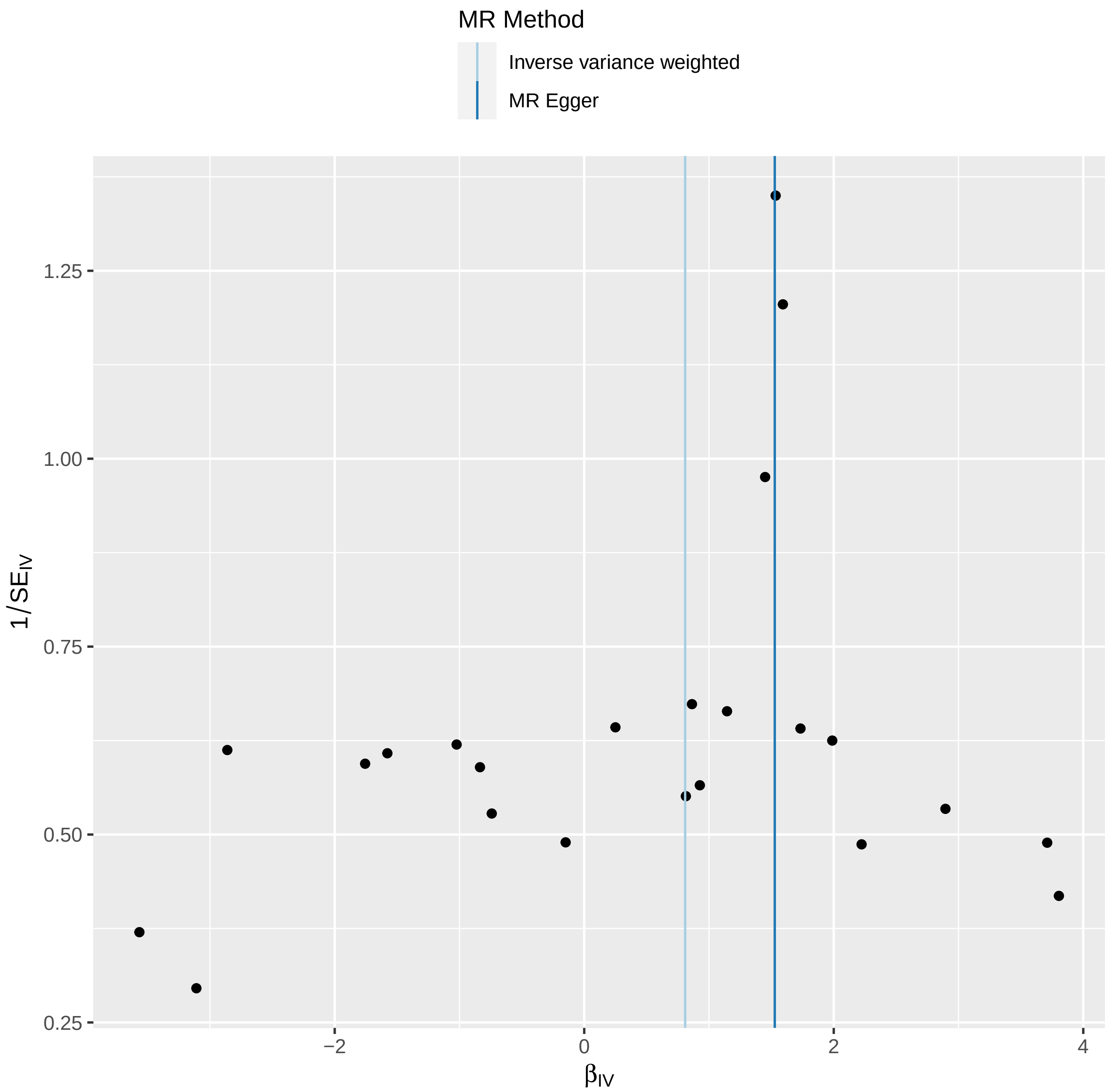

MR Method
Inverse variance weighted
MR Egger
1.25
1.00
0.75
0.50
0.25
1/SE_IV
−2
0
2
4
β_IV

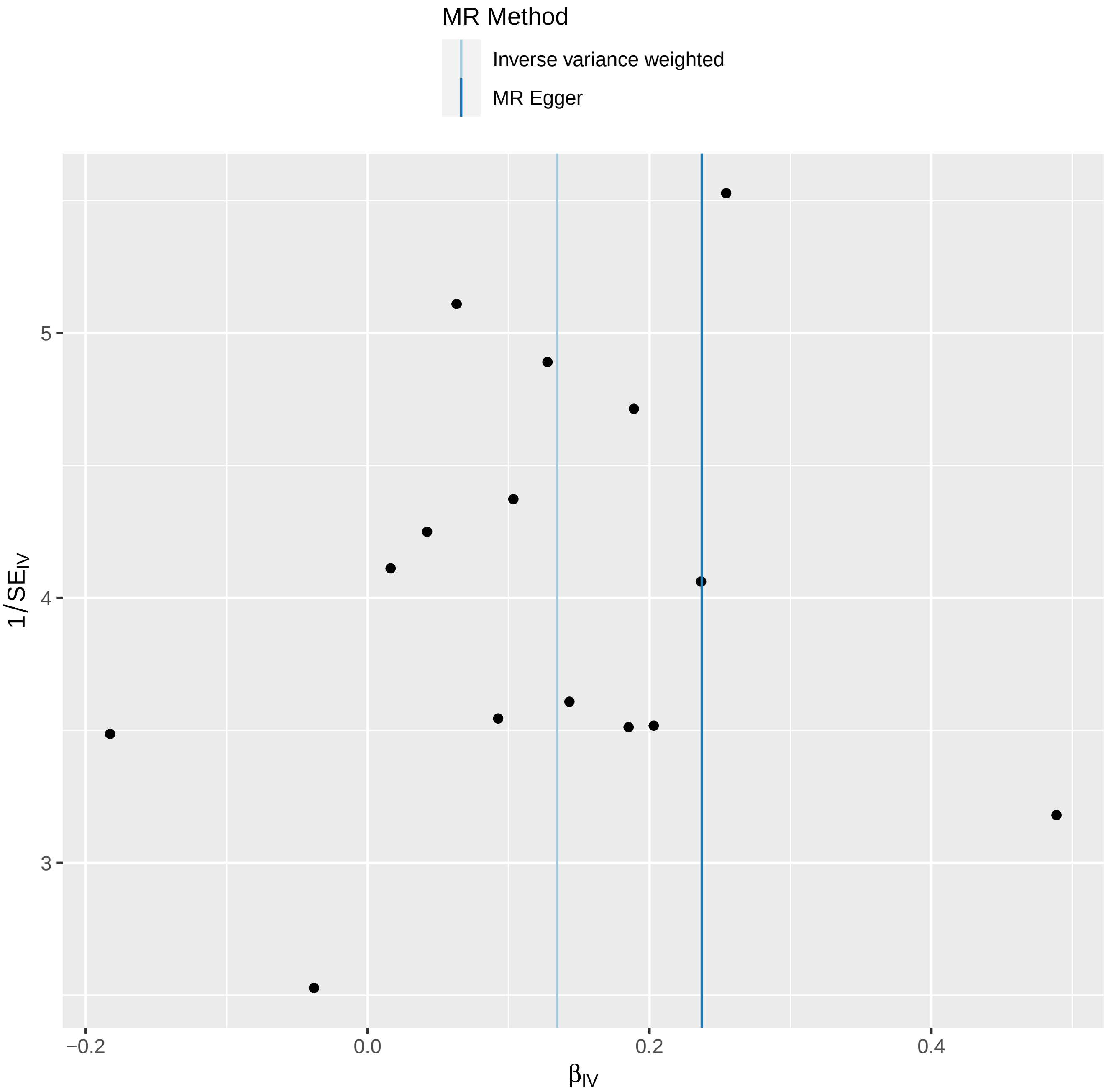
MR Method
Inverse variance weighted
MR Egger
1/SE_IV
5
4
3
−0.2
0.0
0.2
0.4
β_IV

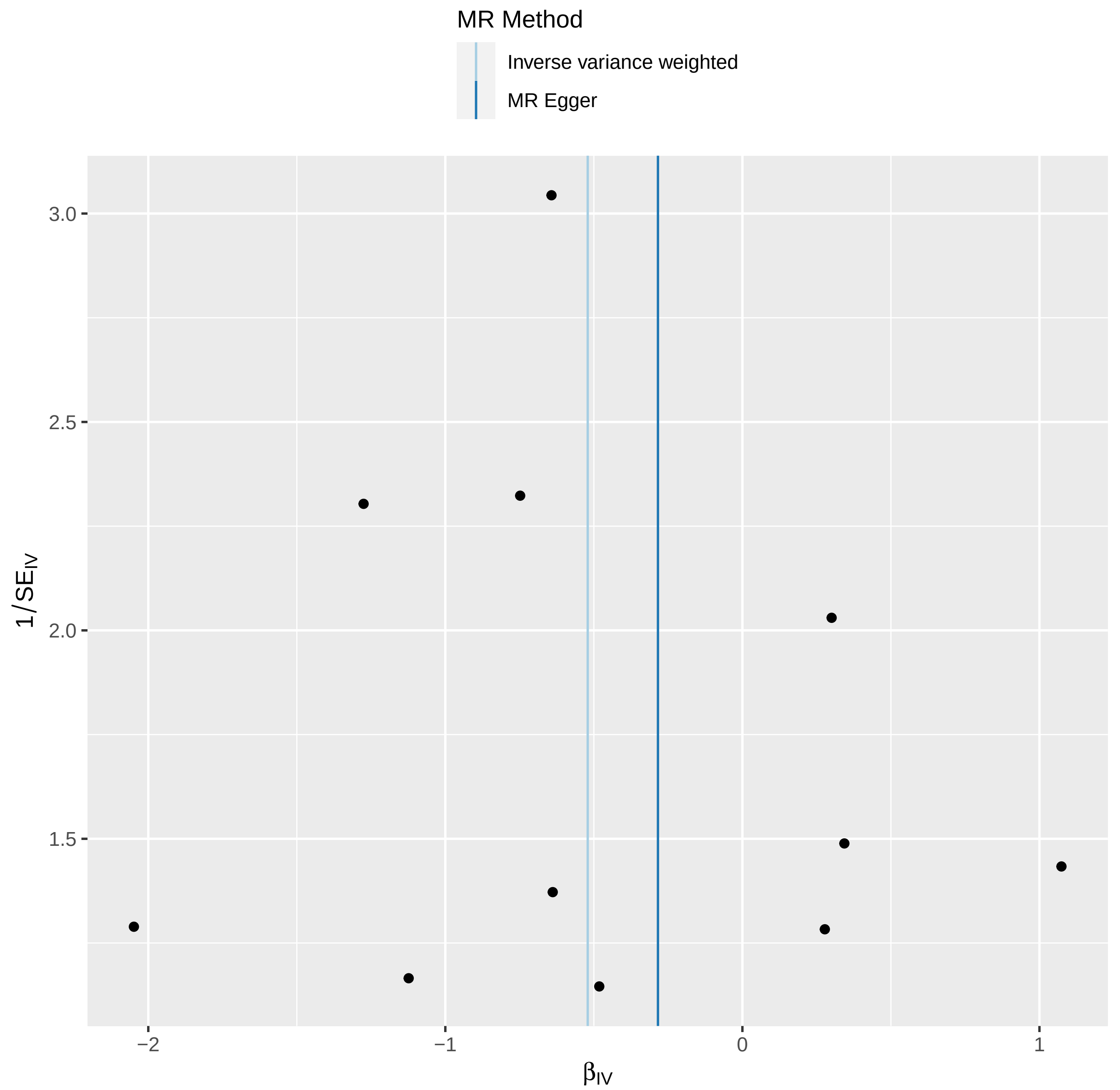

MR Method
Inverse variance weighted
MR Egger
3.0
2.5
2.0
1.5
−2
−1
0
1
$1/SE_{IV}$
β_{IV}

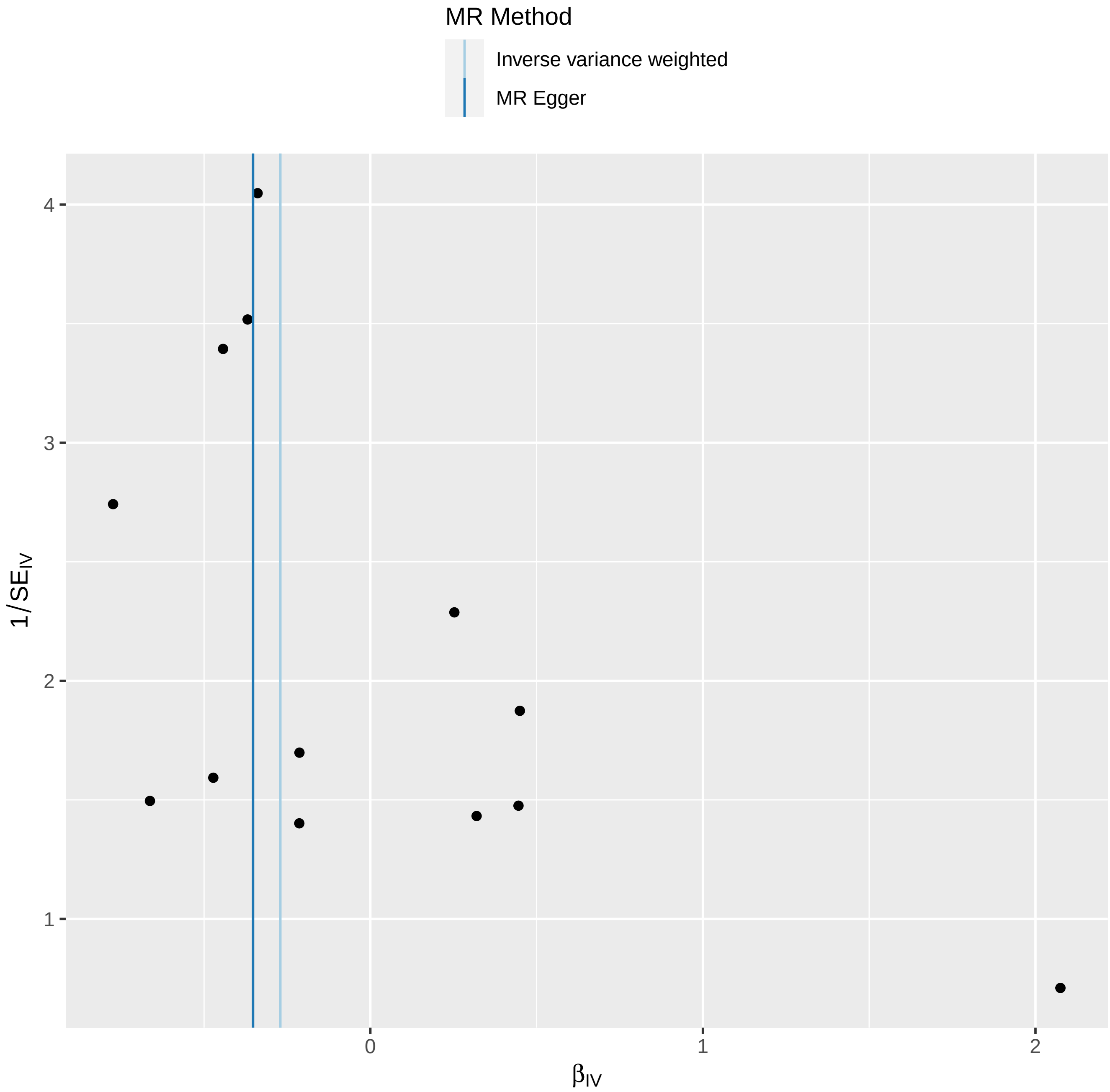
MR Method
Inverse variance weighted
MR Egger
1/SE_IV
β_IV
4
3
2
1
0
1
2

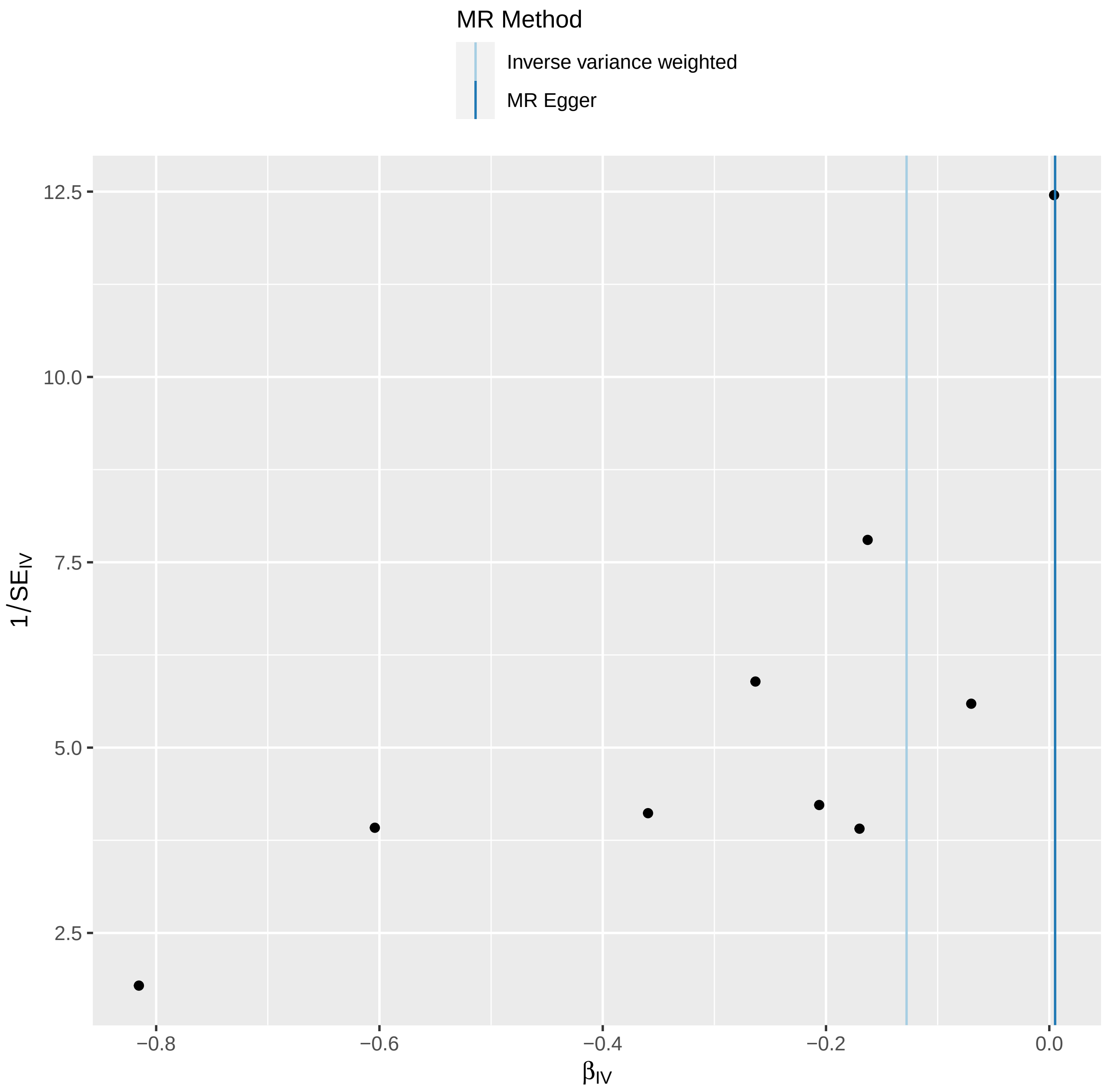
MR Method
Inverse variance weighted
MR Egger
12.5
10.0
7.5
5.0
2.5
1/SE$_{IV}$
−0.8
−0.6
−0.4
−0.2
0.0
β$_{IV}$

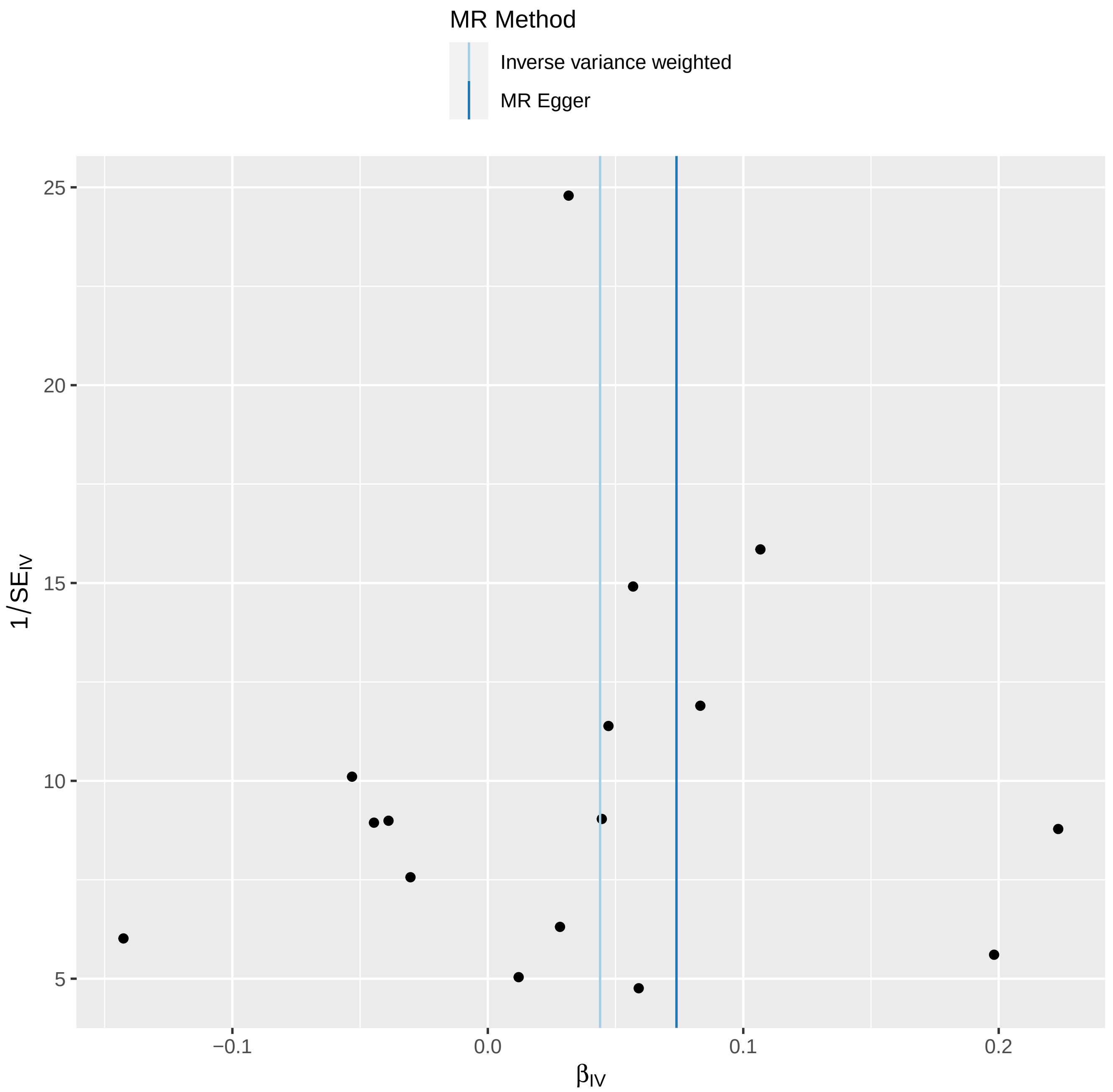
MR Method
Inverse variance weighted
MR Egger
25
20
15
10
5
1/SE_IV
−0.1
0.0
0.1
0.2
β_IV

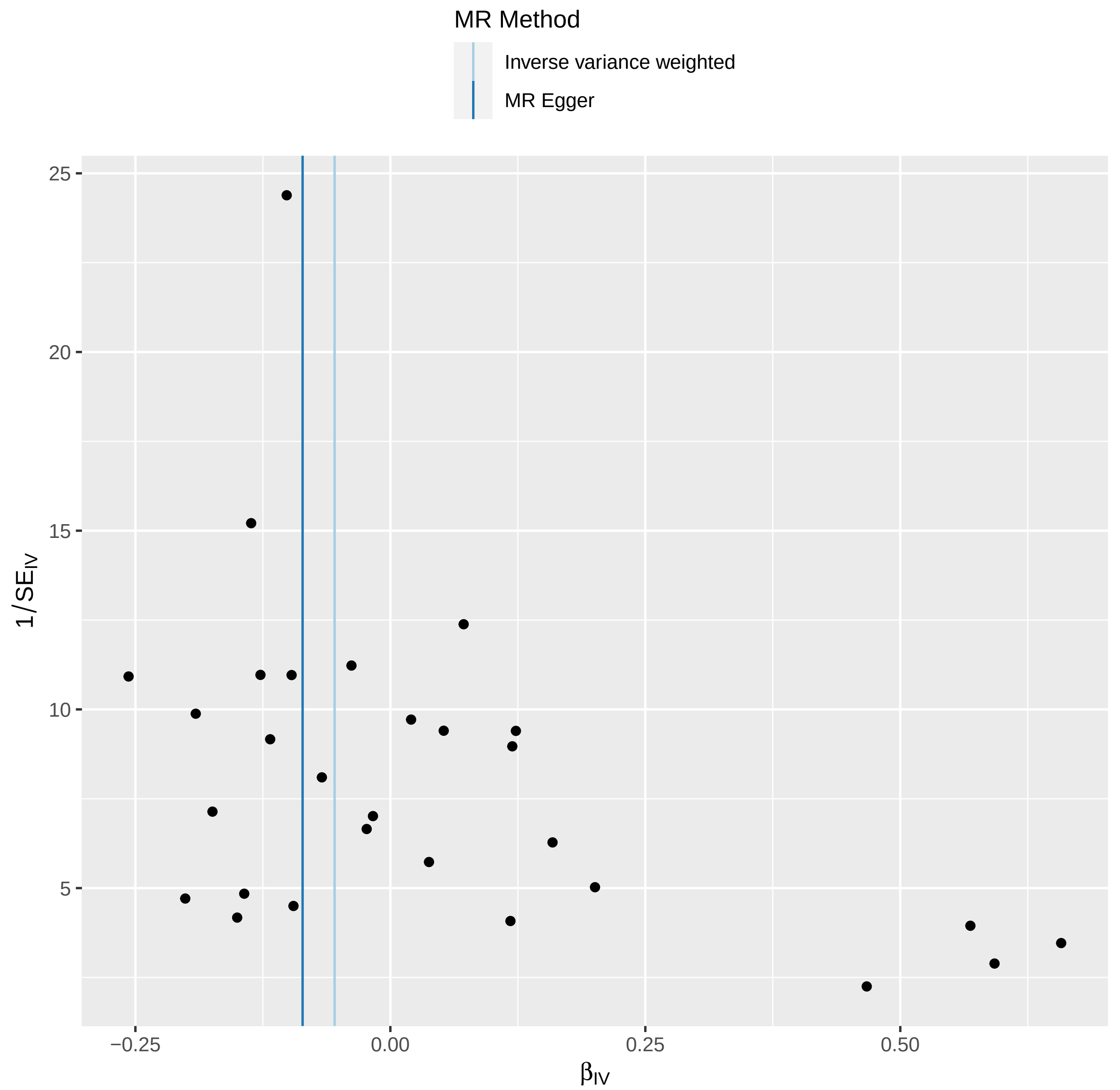

MR Method
Inverse variance weighted
MR Egger
25
20
15
10
5
1/SE_IV
−0.25
0.00
0.25
0.50
β_IV

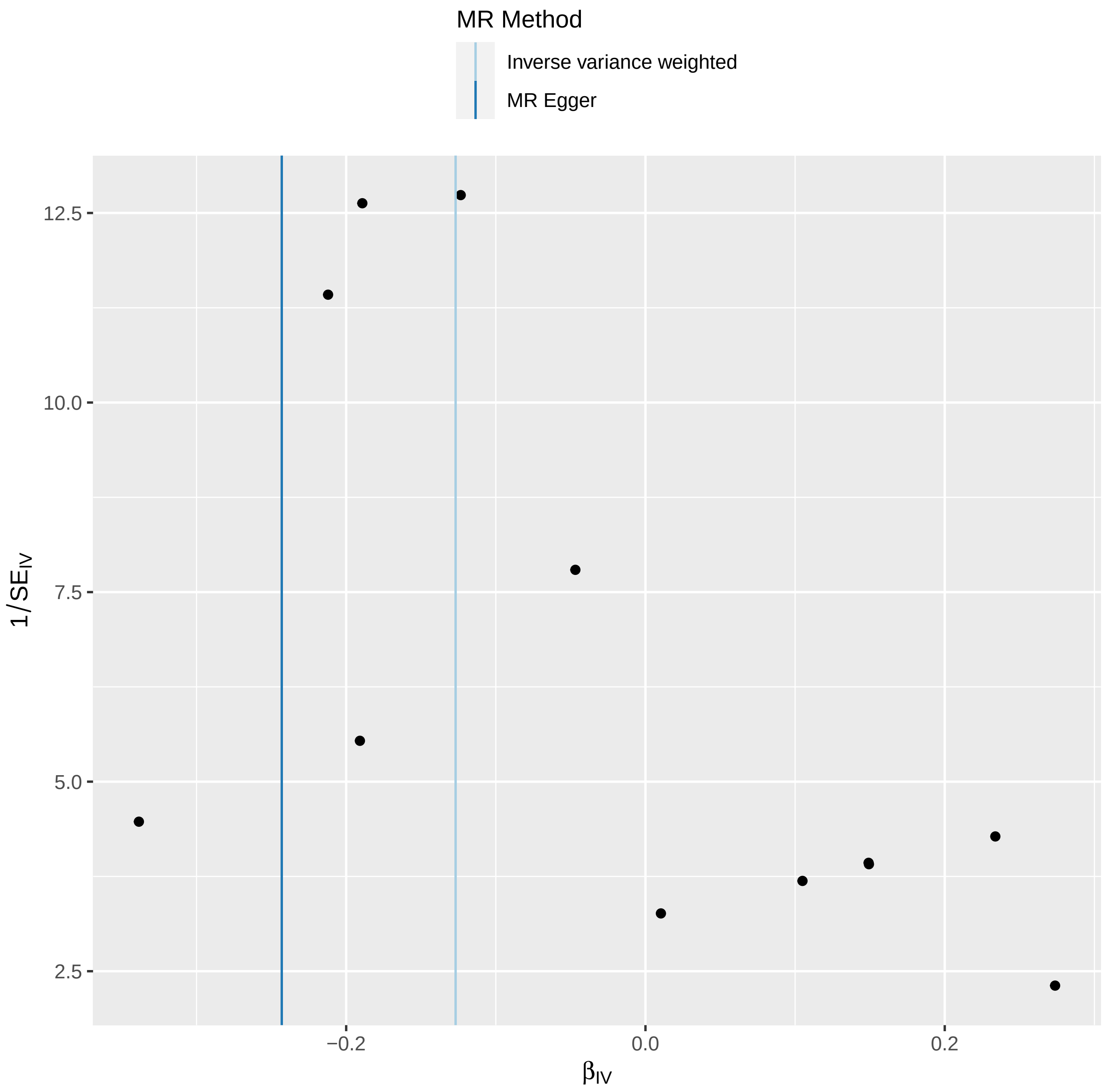

MR Method
Inverse variance weighted
MR Egger
12.5
10.0
7.5
5.0
2.5
$1/SE_{IV}$
−0.2
0.0
0.2
β_{IV}

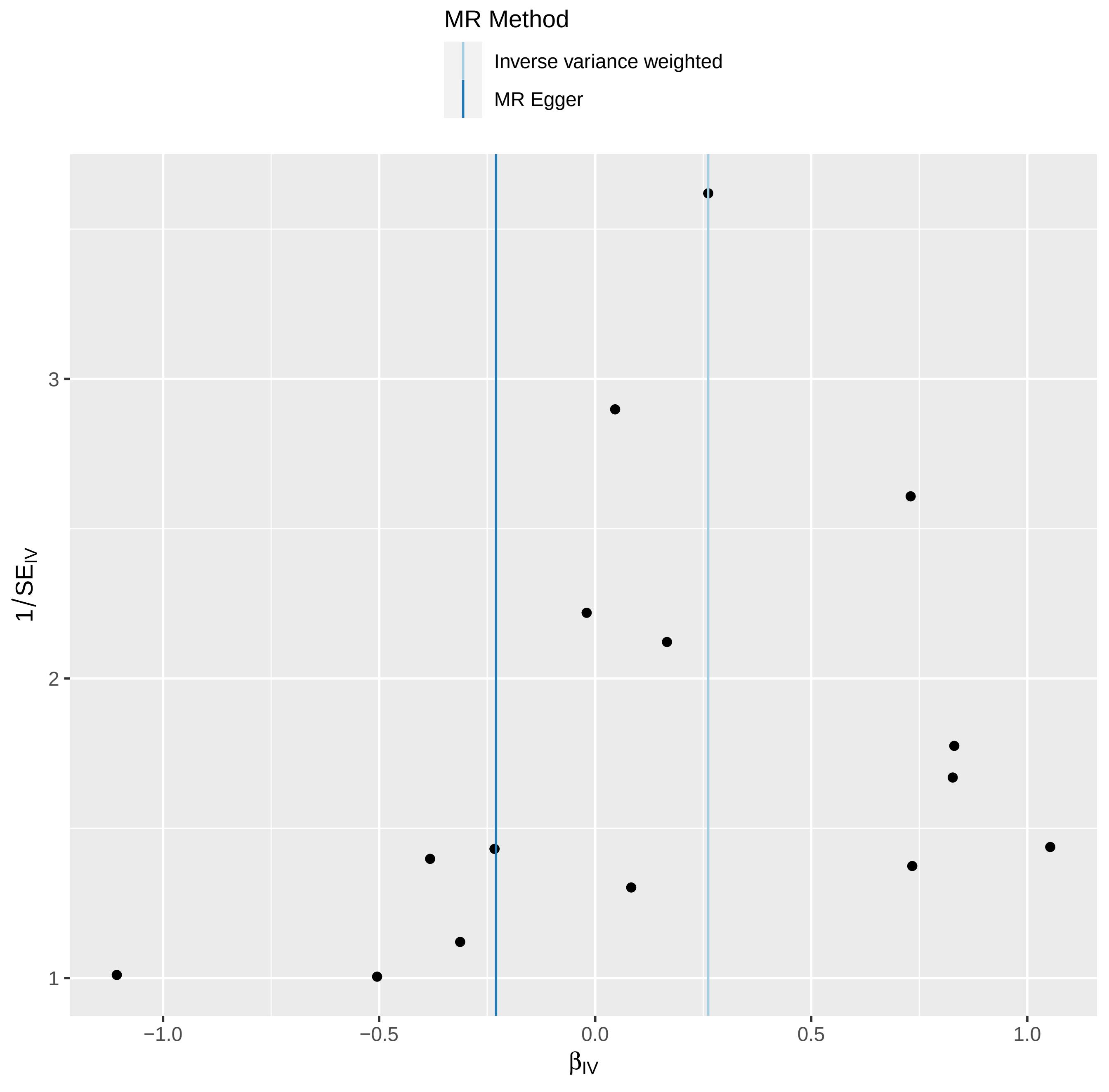
MR Method
Inverse variance weighted
MR Egger
1/SE$_{IV}$
3
2
1
−1.0
−0.5
0.0
0.5
1.0
β$_{IV}$

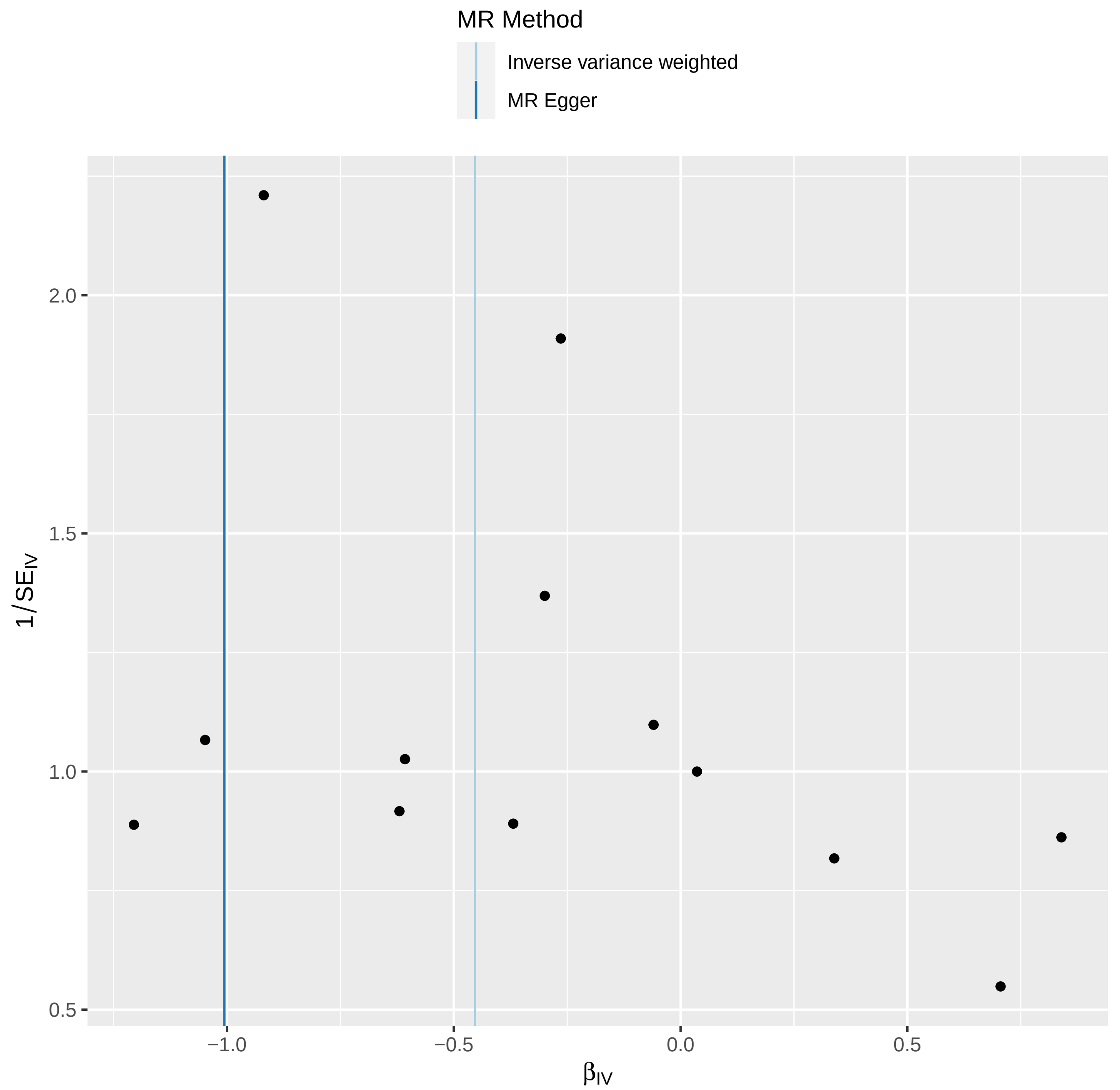

MR Method
Inverse variance weighted
MR Egger
2.0
1.5
1.0
0.5
$1/SE_{IV}$
−1.0
−0.5
0.0
0.5
β_{IV}

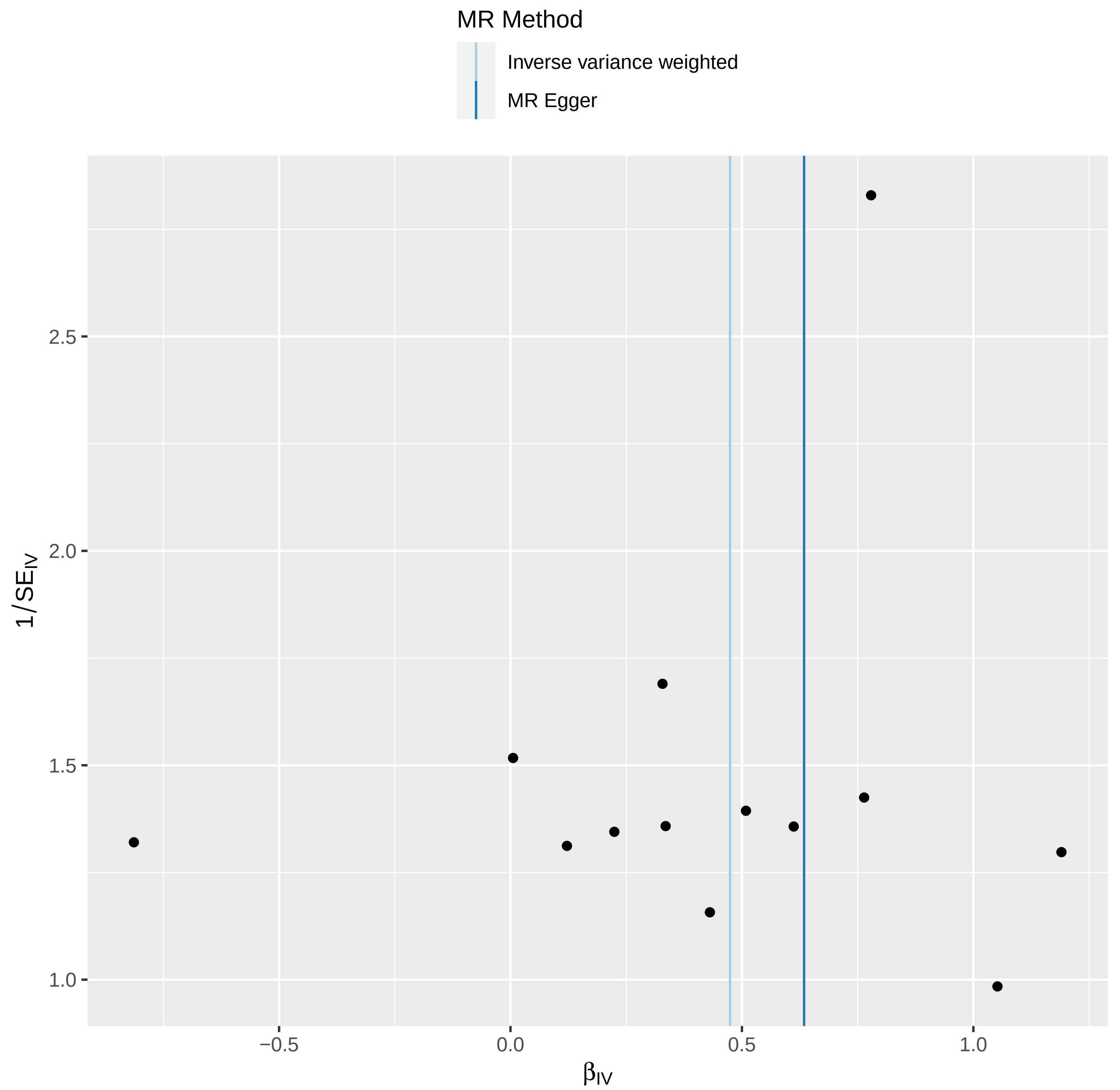

MR Method
Inverse variance weighted
MR Egger
2.5
2.0
1.5
1.0
1/SE$_{IV}$
−0.5
0.0
0.5
1.0
β$_{IV}$

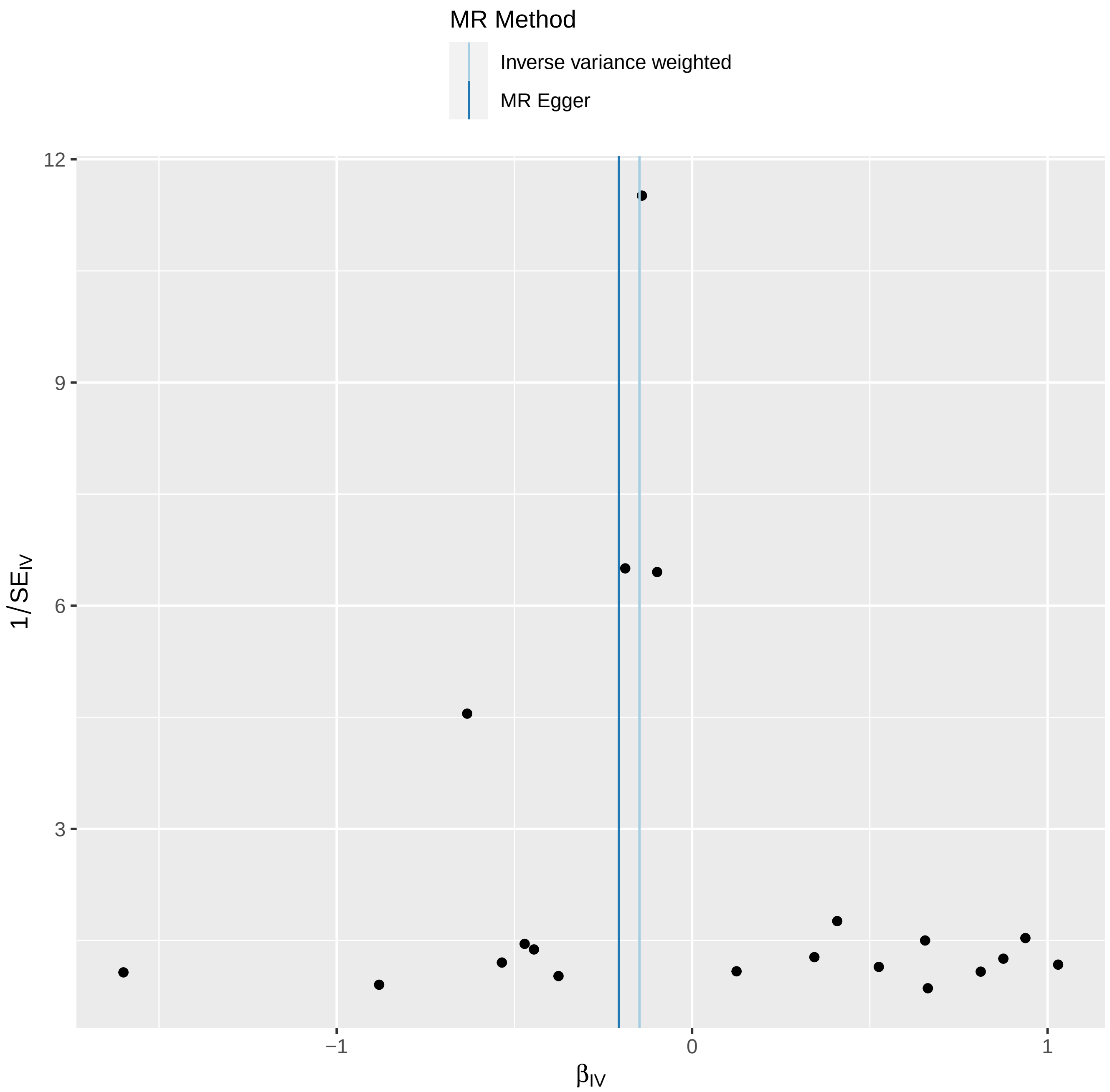

MR Method
Inverse variance weighted
MR Egger
12
9
6
3
1/SE_IV
−1
0
1
β_IV

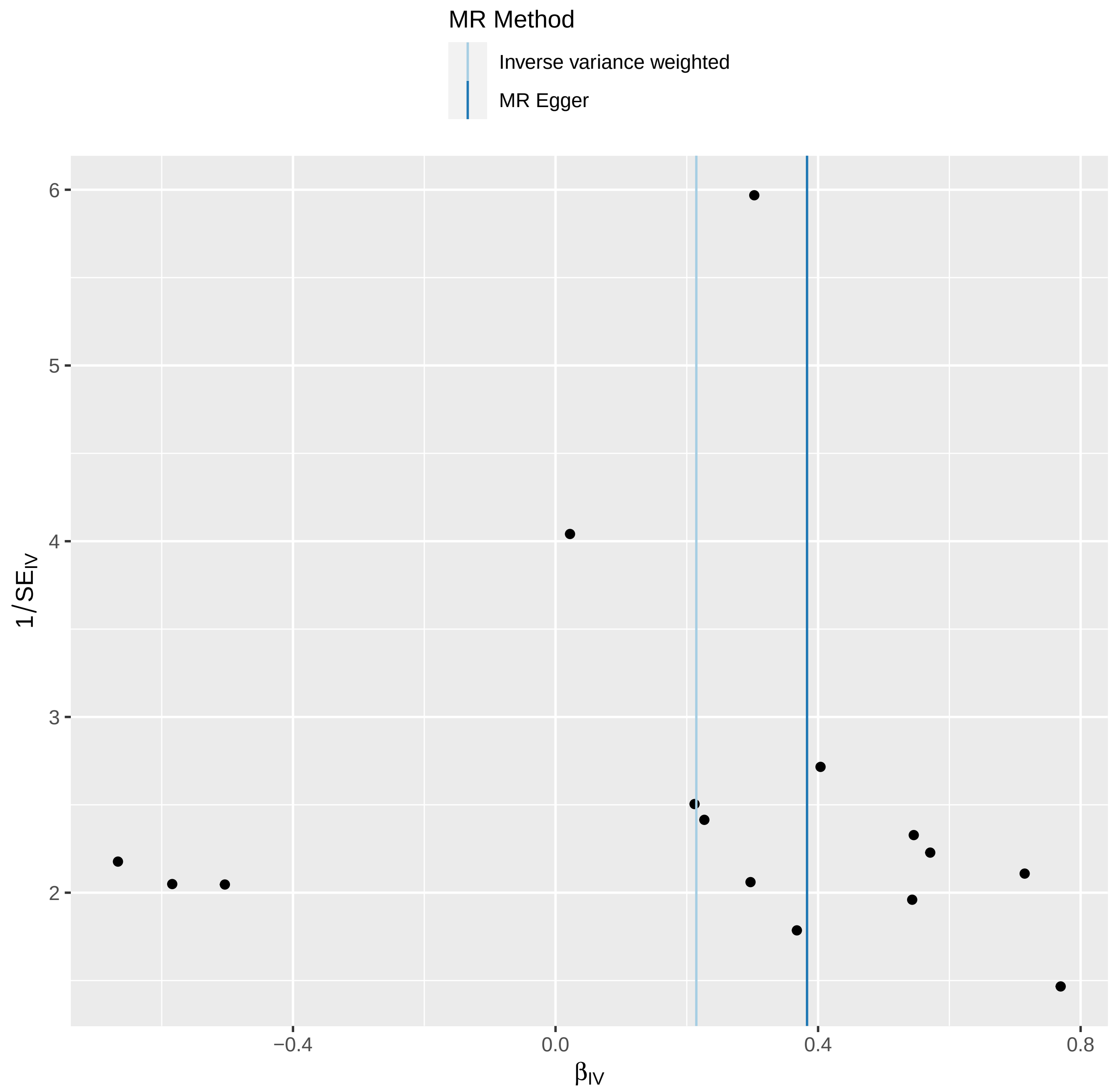

MR Method
Inverse variance weighted
MR Egger
6
5
4
3
2
1/SE_IV
−0.4
0.0
0.4
0.8
β_IV

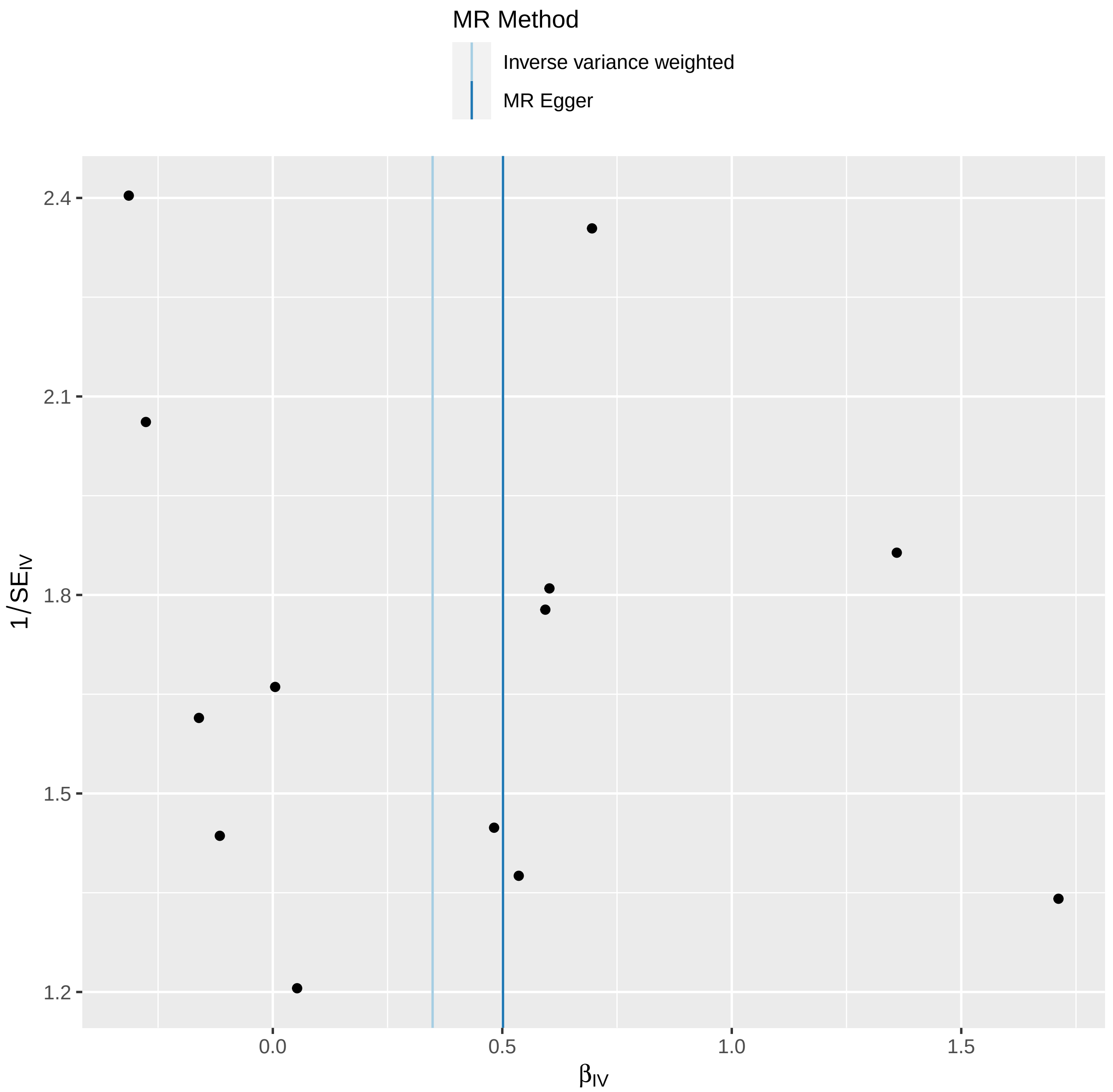
MR Method
Inverse variance weighted
MR Egger
2.4
2.1
1.8
1.5
1.2
1/SE_IV
0.0
0.5
1.0
1.5
β_IV

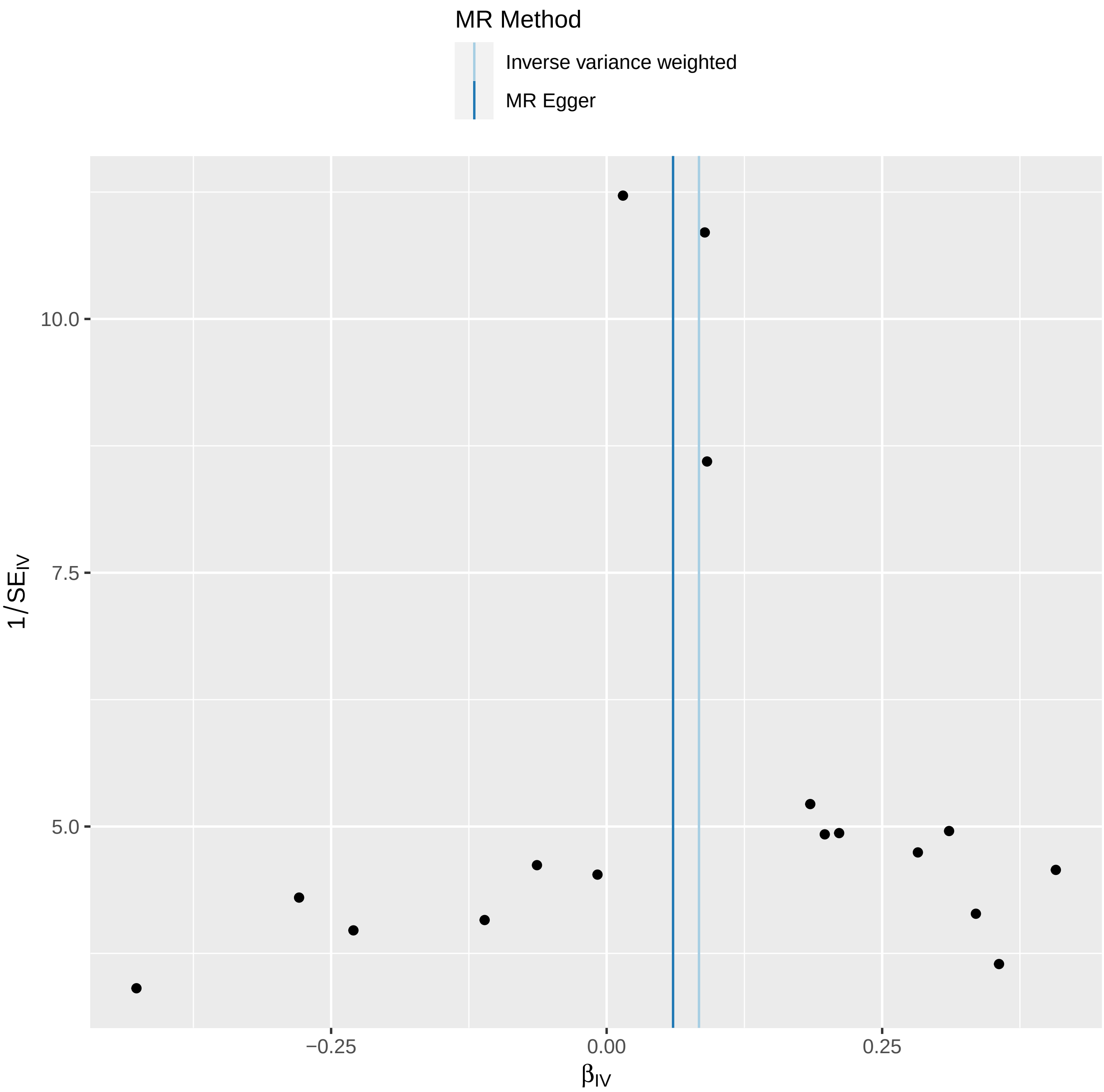
MR Method
Inverse variance weighted
MR Egger
10.0
7.5
5.0
1/SE_IV
−0.25
0.00
0.25
β_IV

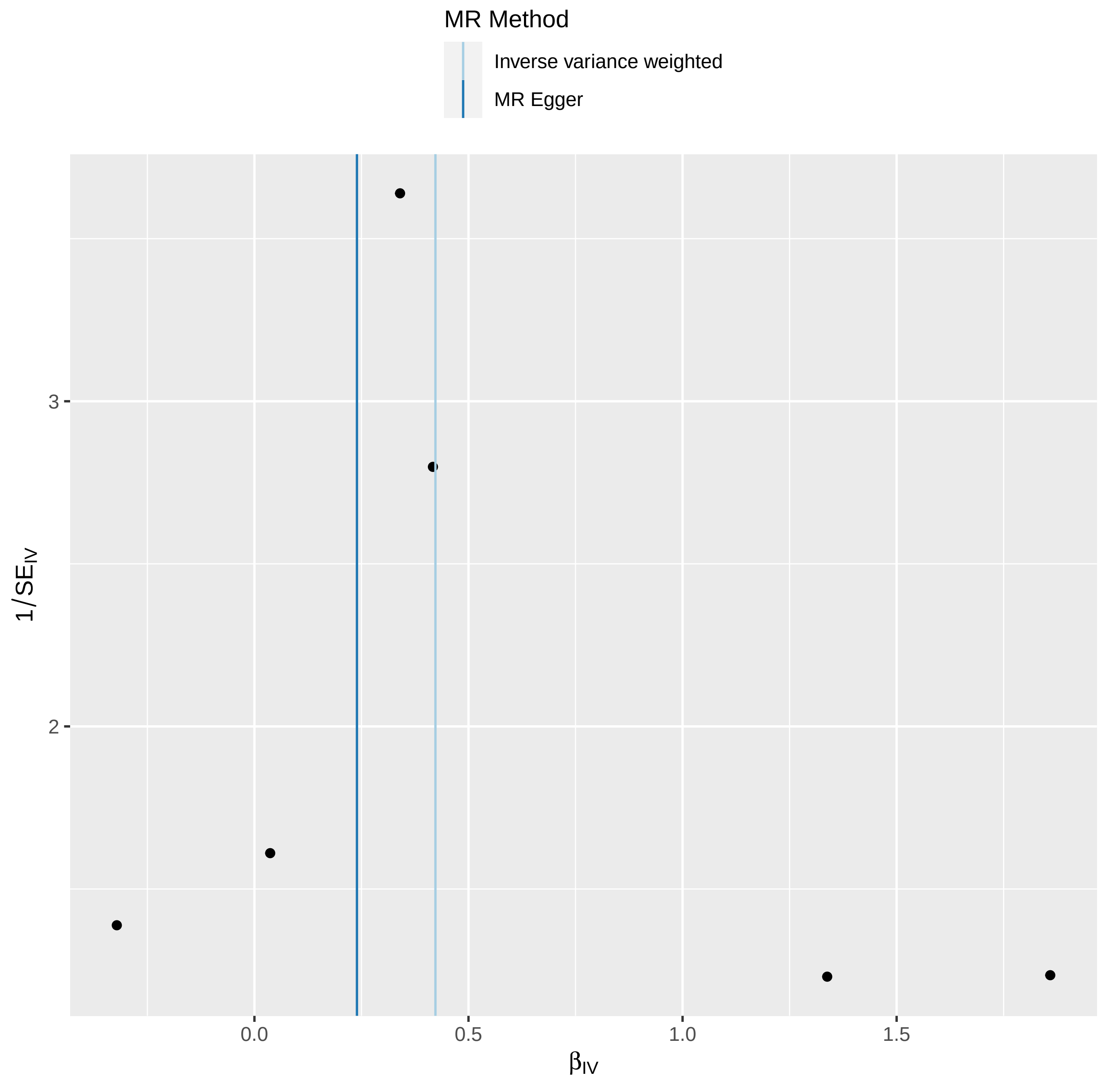
MR Method
Inverse variance weighted
MR Egger
3
2
1/SE_IV
0.0
0.5
1.0
1.5
β_IV
