# Supplementary material for: Causality of genetically determined serum metabolites on lower back pain or/and sciatica: a comprehensive Mendelian randomized study
Source: Front Pain Res (Lausanne). 2024 Sep 25;5:1370704. doi: 10.3389/fpain.2024.1370704 (PMC11461461; doi:10.3389/fpain.2024.1370704)
Supplement: Supplementary Figure S3 — All rest leave-one-out forest maps of 28 metabolites with potential causal relationship with sciatica or/and lower back pain. [file Datasheet6.pdf]

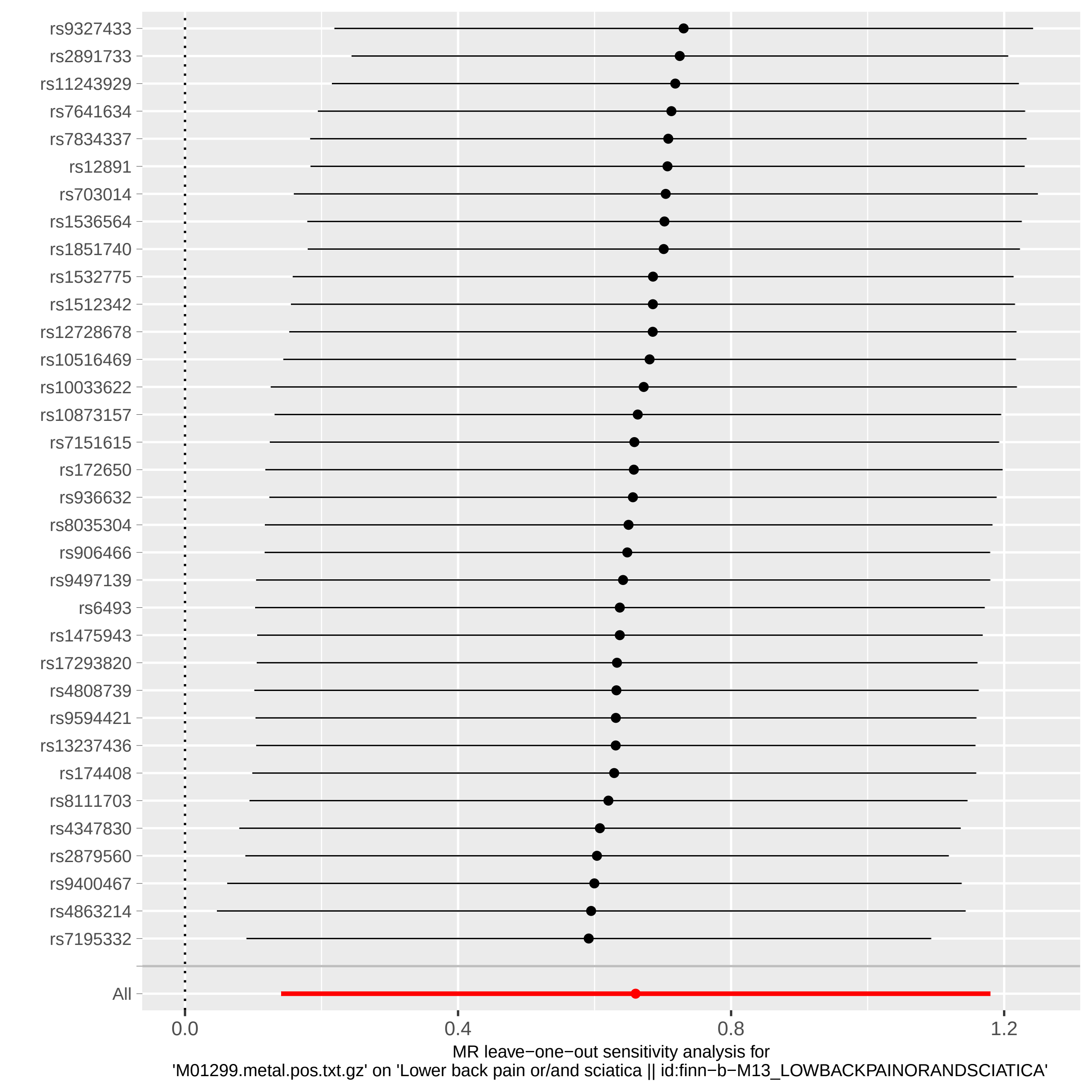
rs9327433
rs2891733
rs11243929
rs7641634
rs7834337
rs12891
rs703014
rs1536564
rs1851740
rs1532775
rs1512342
rs12728678
rs10516469
rs10033622
rs10873157
rs7151615
rs172650
rs936632
rs8035304
rs906466
rs9497139
rs6493
rs1475943
rs17293820
rs4808739
rs9594421
rs13237436
rs174408
rs8111703
rs4347830
rs2879560
rs9400467
rs4863214
rs7195332
All
0.0
0.4
0.8
1.2
MR leave−one−out sensitivity analysis for
'M01299.metal.pos.txt.gz' on 'Lower back pain or/and sciatica || id:finn−b−M13_LOWBACKPAINORANDSCIATICA'

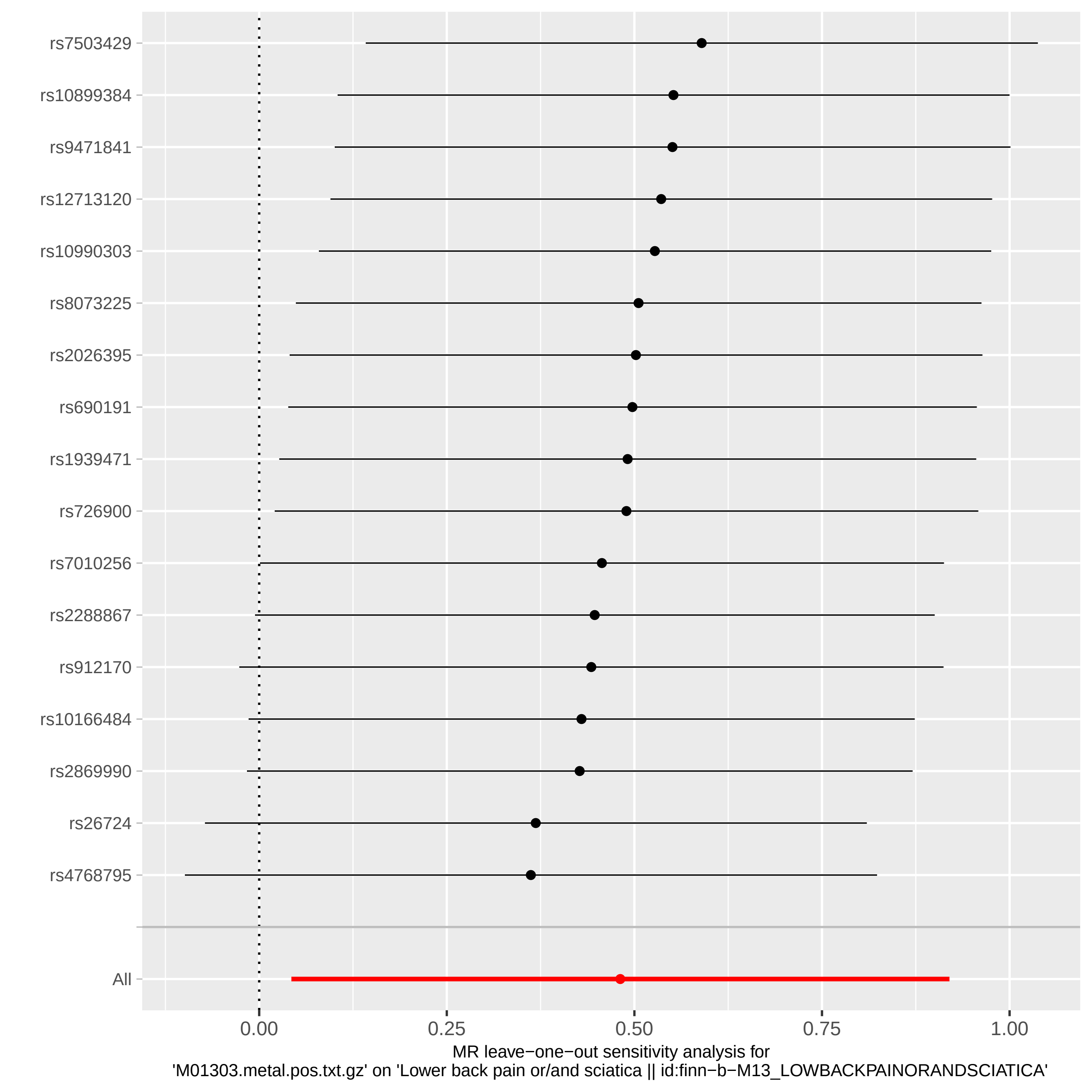
rs7503429
rs10899384
rs9471841
rs12713120
rs10990303
rs8073225
rs2026395
rs690191
rs1939471
rs726900
rs7010256
rs2288867
rs912170
rs10166484
rs2869990
rs26724
rs4768795
All
0.00
0.25
0.50
0.75
1.00
MR leave−one−out sensitivity analysis for
'M01303.metal.pos.txt.gz' on 'Lower back pain or/and sciatica || id:finn−b−M13_LOWBACKPAINORANDSCIATICA'

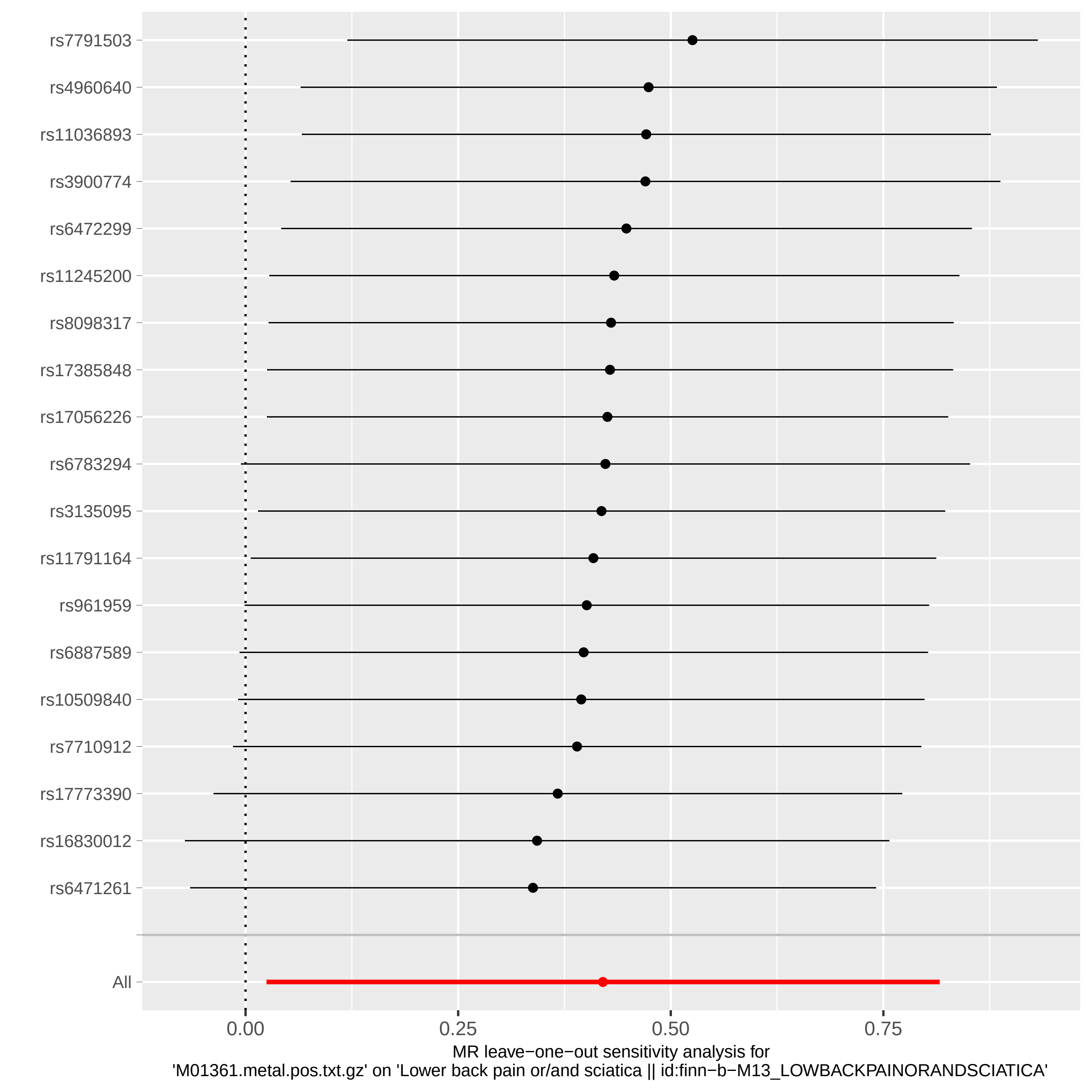
rs7791503
rs4960640
rs11036893
rs3900774
rs6472299
rs11245200
rs8098317
rs17385848
rs17056226
rs6783294
rs3135095
rs11791164
rs961959
rs6887589
rs10509840
rs7710912
rs17773390
rs16830012
rs6471261
All
0.00
0.25
0.50
0.75
MR leave−one−out sensitivity analysis for
'M01361.metal.pos.txt.gz' on 'Lower back pain or/and sciatica || id:finn−b−M13_LOWBACKPAINORANDSCIATICA'

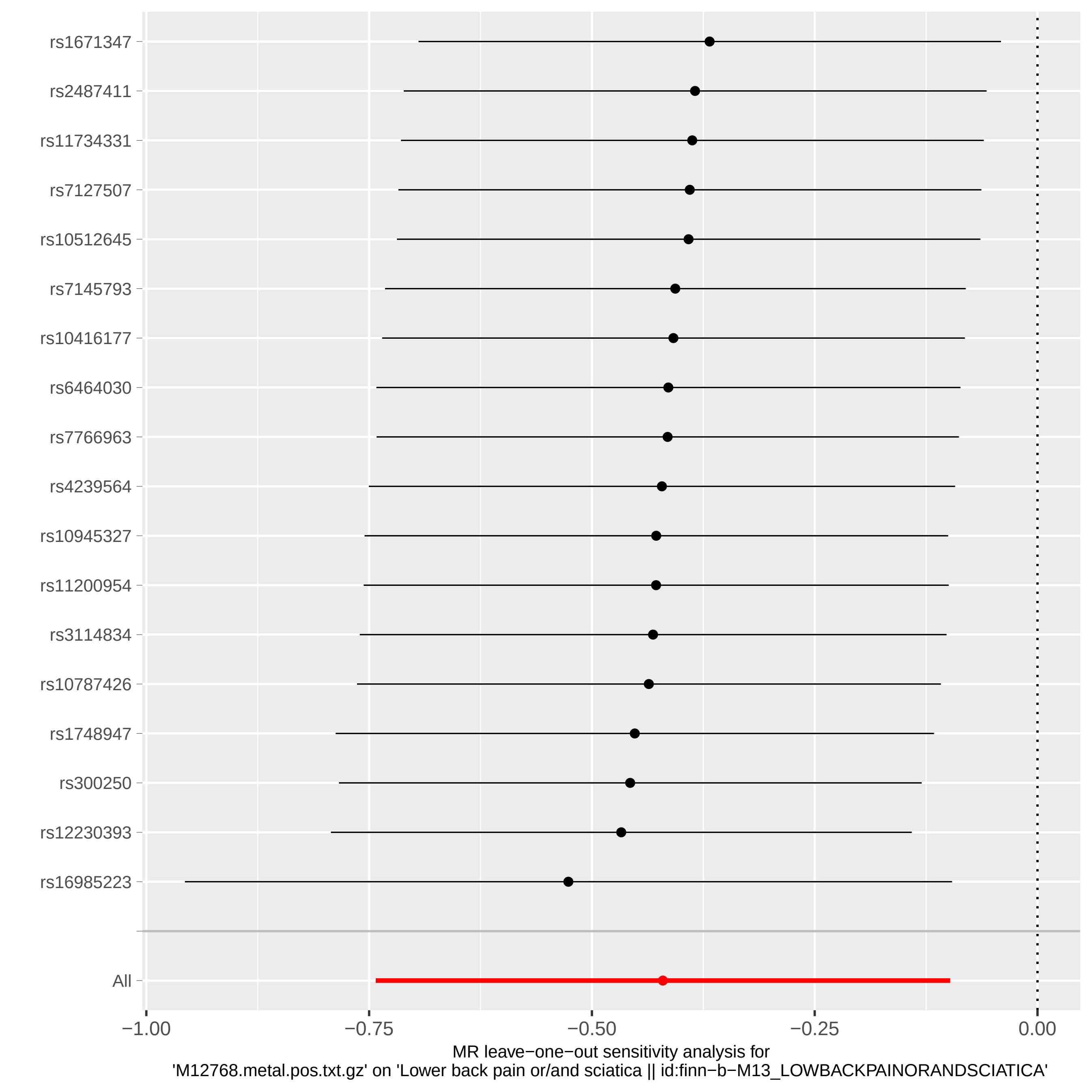
rs1671347
rs2487411
rs11734331
rs7127507
rs10512645
rs7145793
rs10416177
rs6464030
rs7766963
rs4239564
rs10945327
rs11200954
rs3114834
rs10787426
rs1748947
rs300250
rs12230393
rs16985223
All
−1.00
−0.75
−0.50
−0.25
0.00
MR leave−one−out sensitivity analysis for
'M12768.metal.pos.txt.gz' on 'Lower back pain or/and sciatica || id:finn−b−M13_LOWBACKPAINORANDSCIATICA'

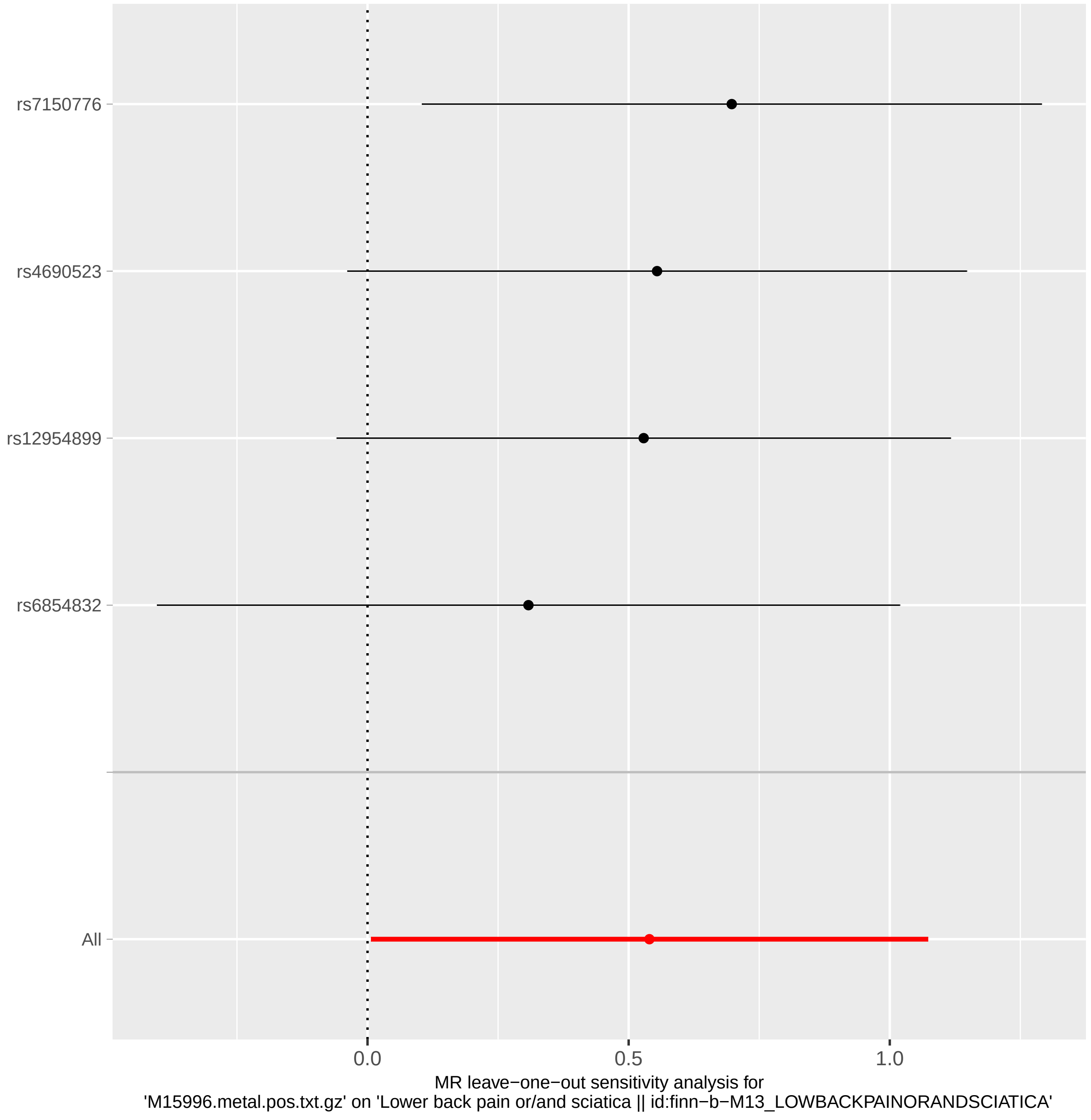
rs7150776
rs4690523
rs12954899
rs6854832
All
0.0
0.5
1.0
MR leave−one−out sensitivity analysis for
'M15996.metal.pos.txt.gz' on 'Lower back pain or/and sciatica || id:finn−b−M13_LOWBACKPAINORANDSCIATICA'

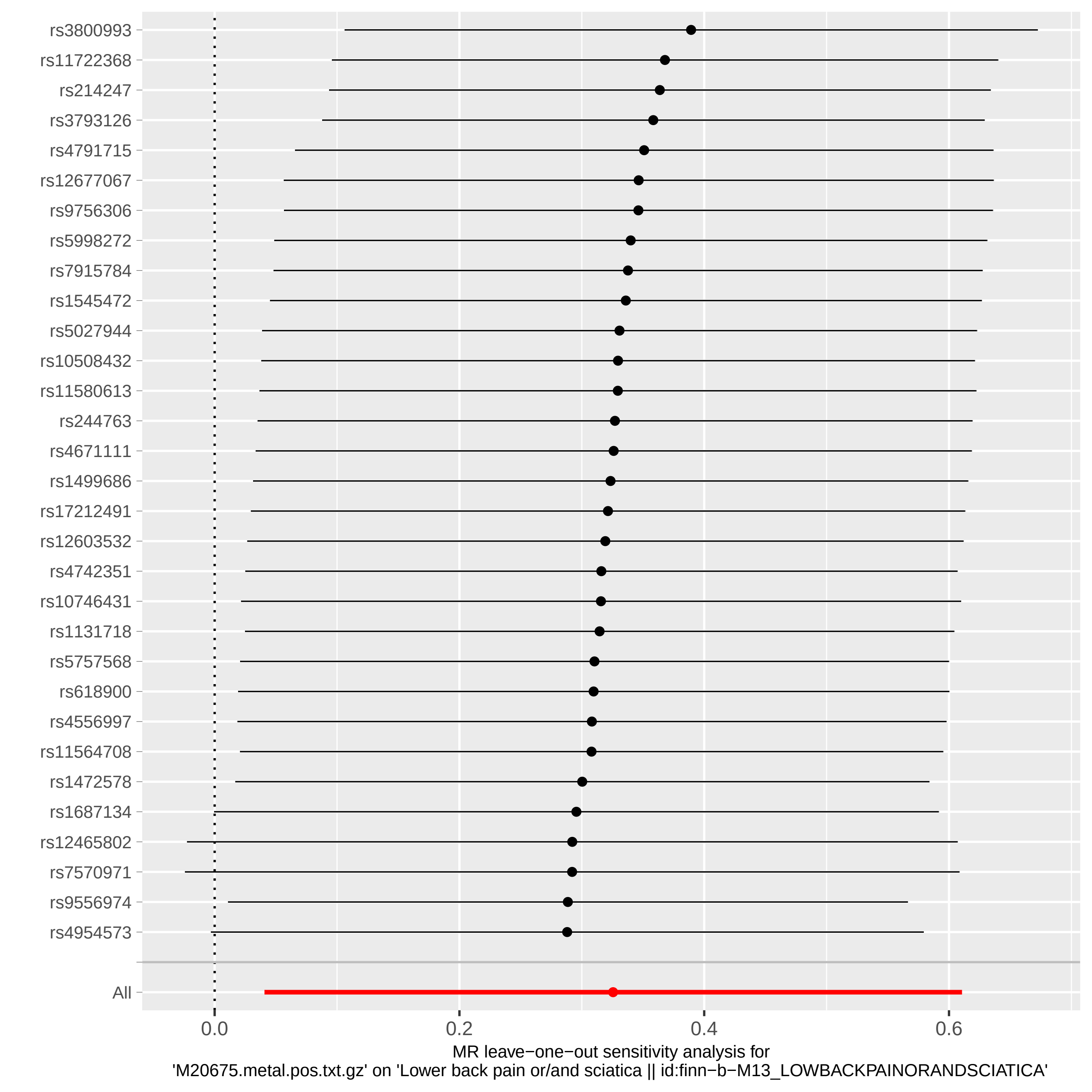
rs3800993
rs11722368
rs214247
rs3793126
rs4791715
rs12677067
rs9756306
rs5998272
rs7915784
rs1545472
rs5027944
rs10508432
rs11580613
rs244763
rs4671111
rs1499686
rs17212491
rs12603532
rs4742351
rs10746431
rs1131718
rs5757568
rs618900
rs4556997
rs11564708
rs1472578
rs1687134
rs12465802
rs7570971
rs9556974
rs4954573
All
0.0
0.2
0.4
0.6
MR leave−one−out sensitivity analysis for
'M20675.metal.pos.txt.gz' on 'Lower back pain or/and sciatica || id:finn−b−M13_LOWBACKPAINORANDSCIATICA'

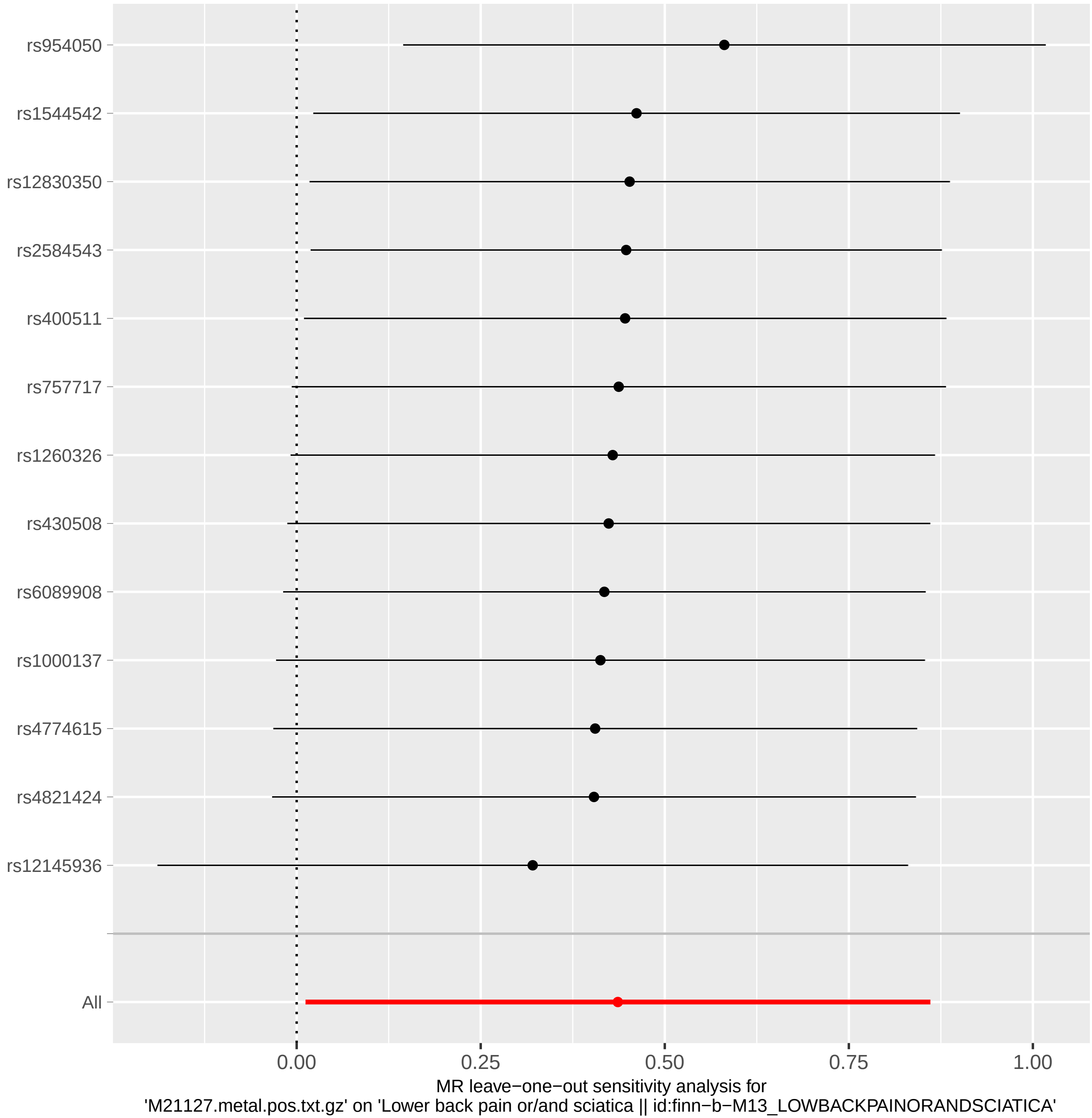
rs954050
rs1544542
rs12830350
rs2584543
rs400511
rs757717
rs1260326
rs430508
rs6089908
rs1000137
rs4774615
rs4821424
rs12145936
All
0.00
0.25
0.50
0.75
1.00
MR leave−one−out sensitivity analysis for
'M21127.metal.pos.txt.gz' on 'Lower back pain or/and sciatica || id:finn−b−M13_LOWBACKPAINORANDSCIATICA'

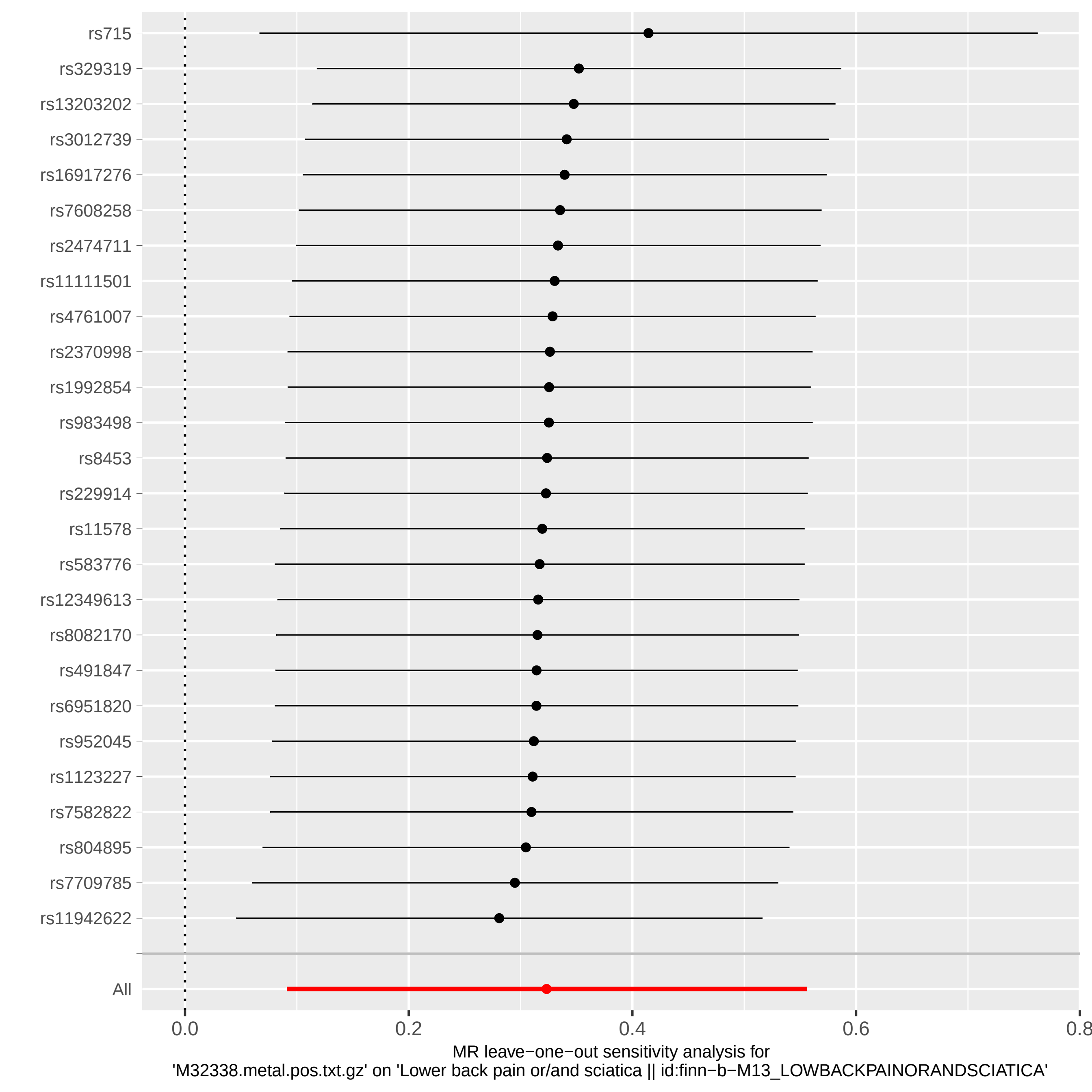

rs715
rs329319
rs13203202
rs3012739
rs16917276
rs7608258
rs2474711
rs11111501
rs4761007
rs2370998
rs1992854
rs983498
rs8453
rs229914
rs11578
rs583776
rs12349613
rs8082170
rs491847
rs6951820
rs952045
rs1123227
rs7582822
rs804895
rs7709785
rs11942622
All
0.0
0.2
0.4
0.6
0.8
MR leave−one−out sensitivity analysis for
'M32338.metal.pos.txt.gz' on 'Lower back pain or/and sciatica || id:finn−b−M13_LOWBACKPAINORANDSCIATICA'

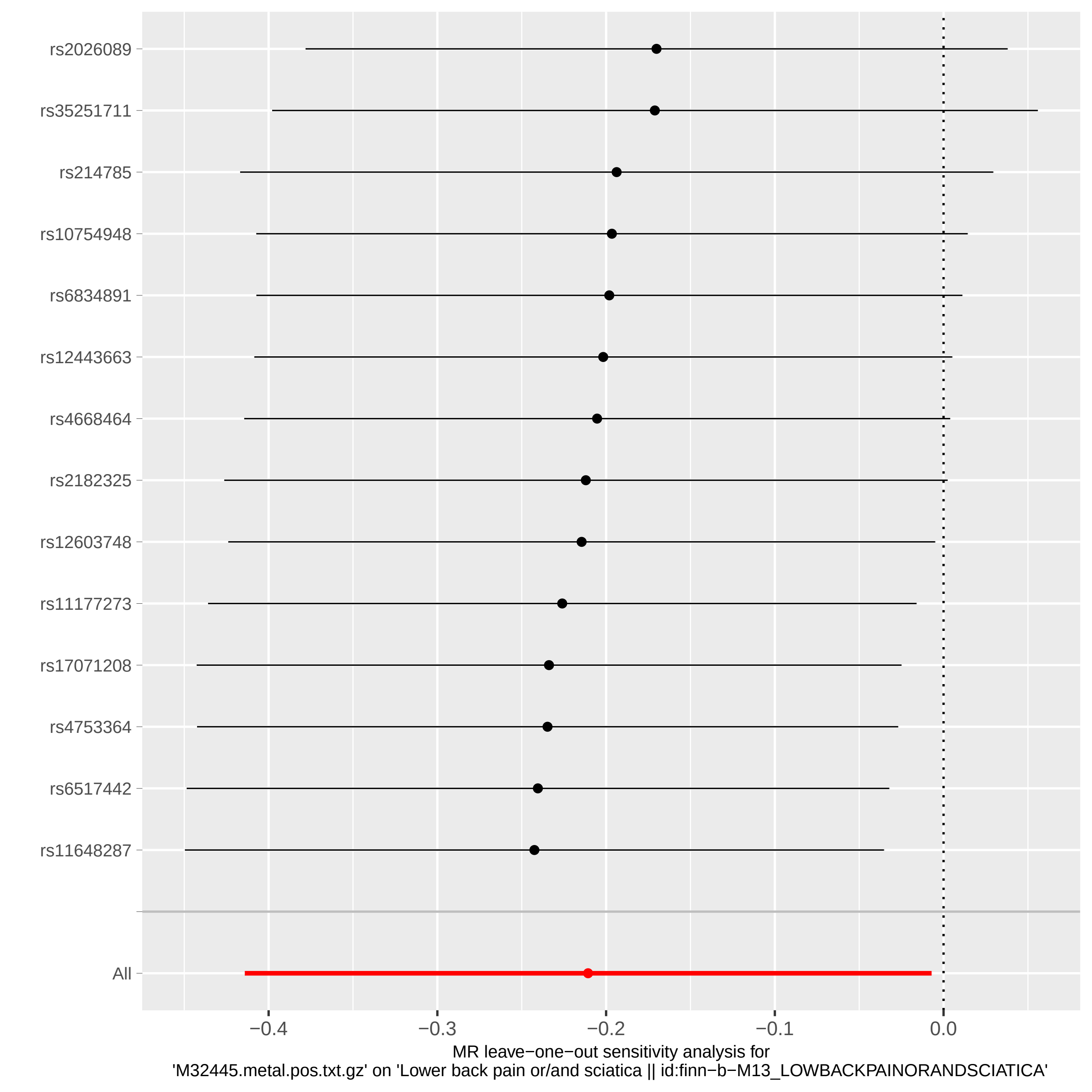
rs2026089
rs35251711
rs214785
rs10754948
rs6834891
rs12443663
rs4668464
rs2182325
rs12603748
rs11177273
rs17071208
rs4753364
rs6517442
rs11648287
All
−0.4
−0.3
−0.2
−0.1
0.0
MR leave−one−out sensitivity analysis for
'M32445.metal.pos.txt.gz' on 'Lower back pain or/and sciatica || id:finn−b−M13_LOWBACKPAINORANDSCIATICA'

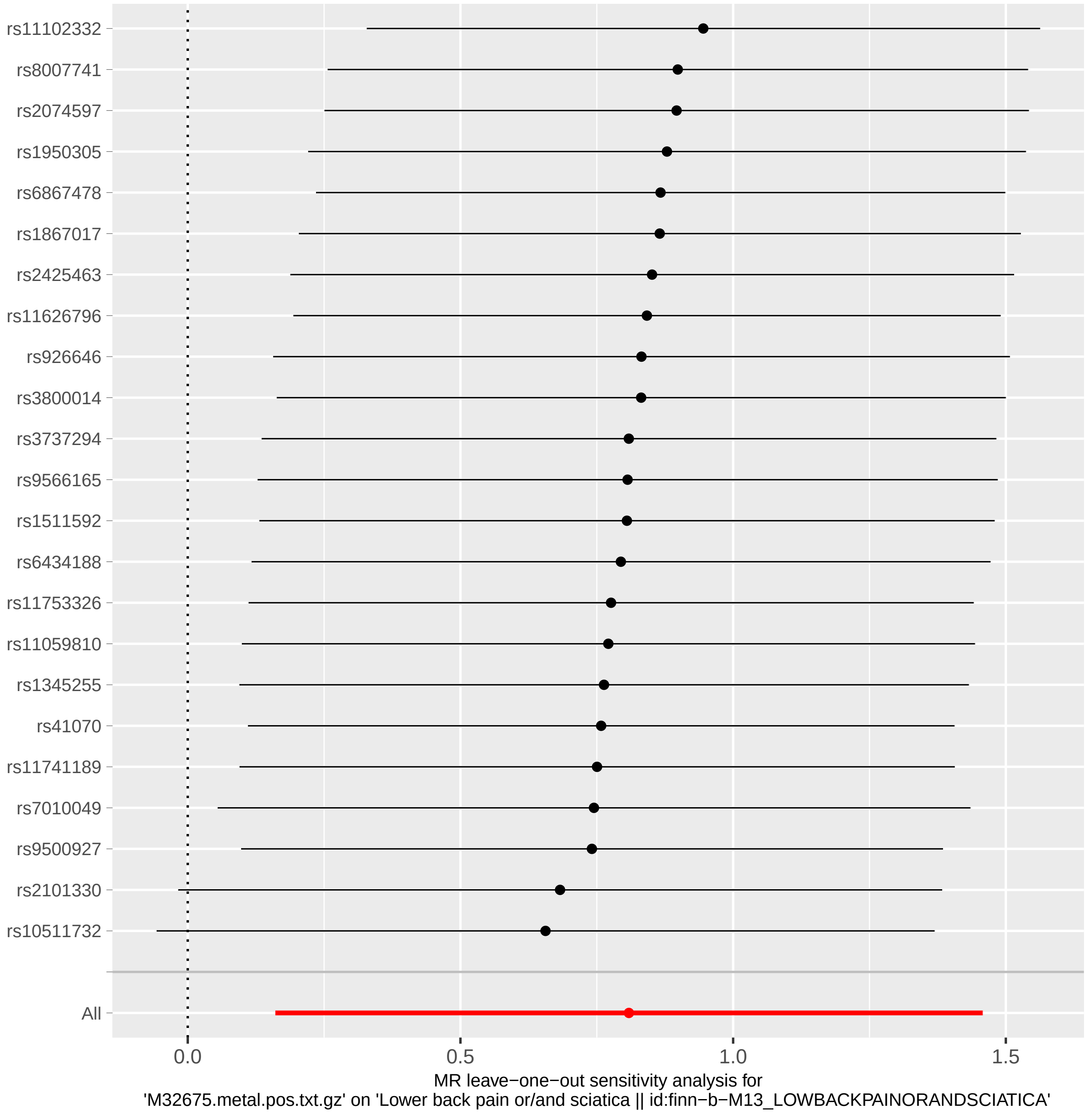

rs11102332
rs8007741
rs2074597
rs1950305
rs6867478
rs1867017
rs2425463
rs11626796
rs926646
rs3800014
rs3737294
rs9566165
rs1511592
rs6434188
rs11753326
rs11059810
rs1345255
rs41070
rs11741189
rs7010049
rs9500927
rs2101330
rs10511732
All
0.0
0.5
1.0
1.5
MR leave−one−out sensitivity analysis for
'M32675.metal.pos.txt.gz' on 'Lower back pain or/and sciatica || id:finn−b−M13_LOWBACKPAINORANDSCIATICA'

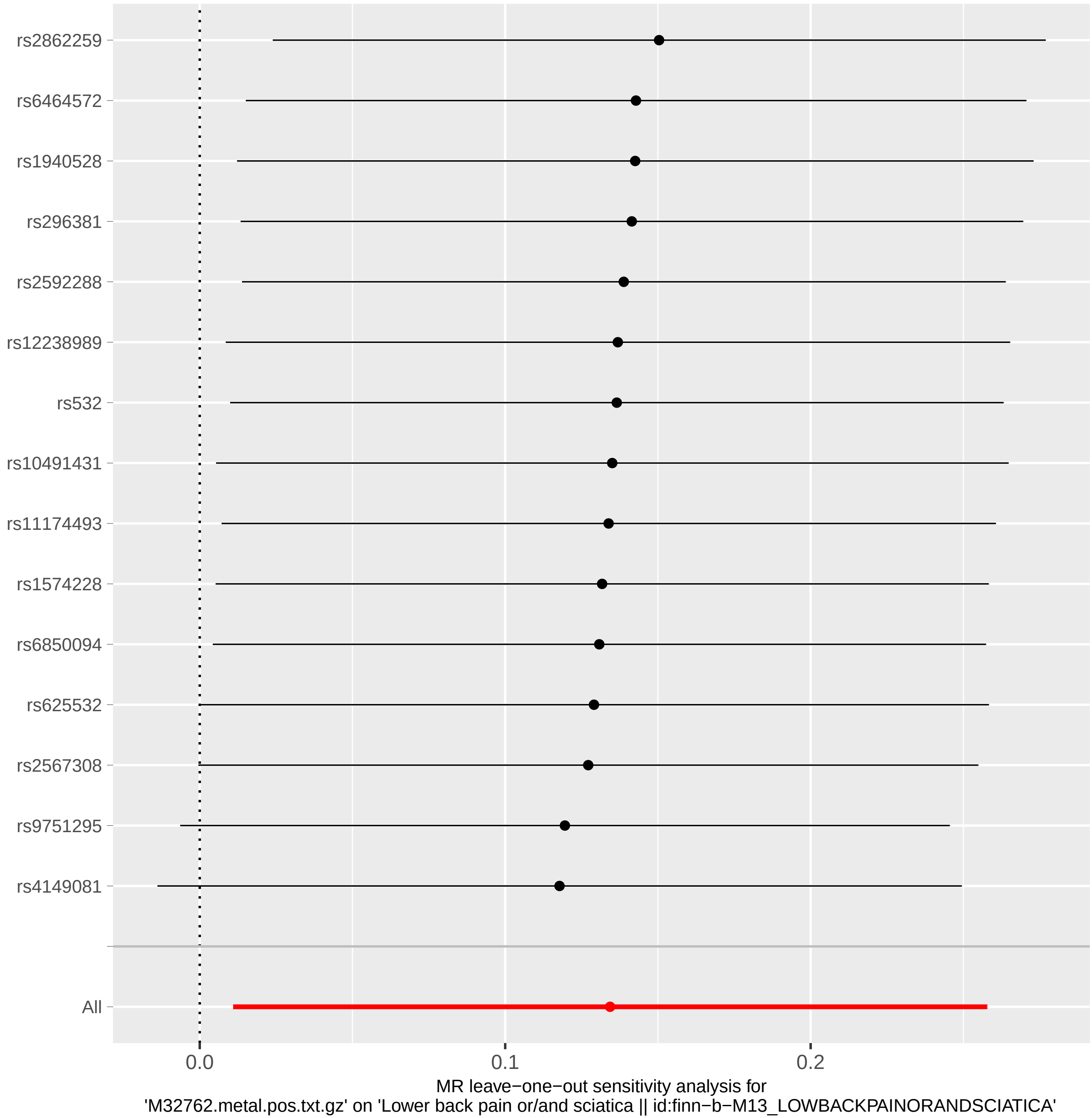
rs2862259
rs6464572
rs1940528
rs296381
rs2592288
rs12238989
rs532
rs10491431
rs11174493
rs1574228
rs6850094
rs625532
rs2567308
rs9751295
rs4149081
All
0.0
0.1
0.2
MR leave−one−out sensitivity analysis for
'M32762.metal.pos.txt.gz' on 'Lower back pain or/and sciatica || id:finn−b−M13_LOWBACKPAINORANDSCIATICA'

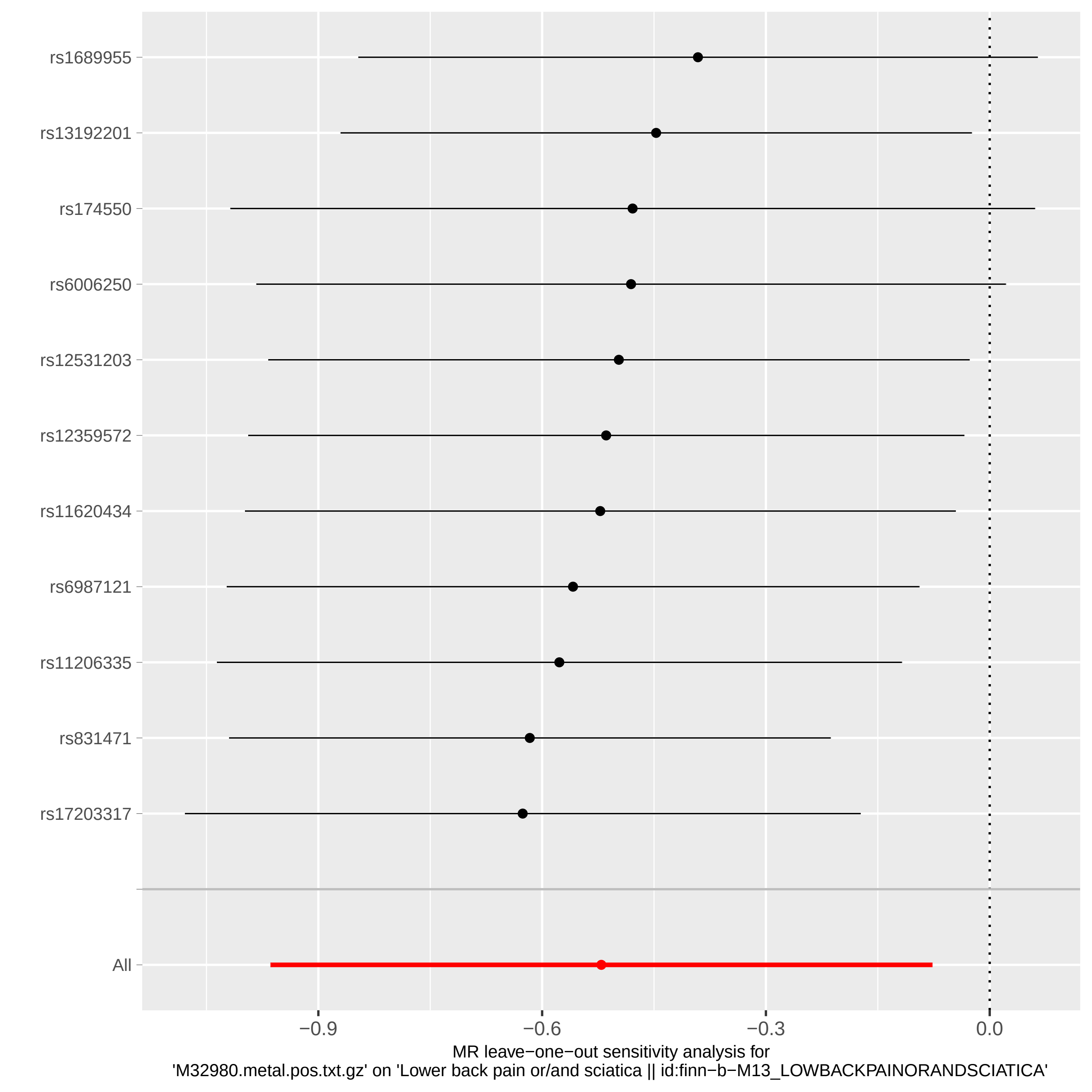
rs1689955
rs13192201
rs174550
rs6006250
rs12531203
rs12359572
rs11620434
rs6987121
rs11206335
rs831471
rs17203317
All
−0.9
−0.6
−0.3
0.0
MR leave−one−out sensitivity analysis for
'M32980.metal.pos.txt.gz' on 'Lower back pain or/and sciatica || id:finn−b−M13_LOWBACKPAINORANDSCIATICA'

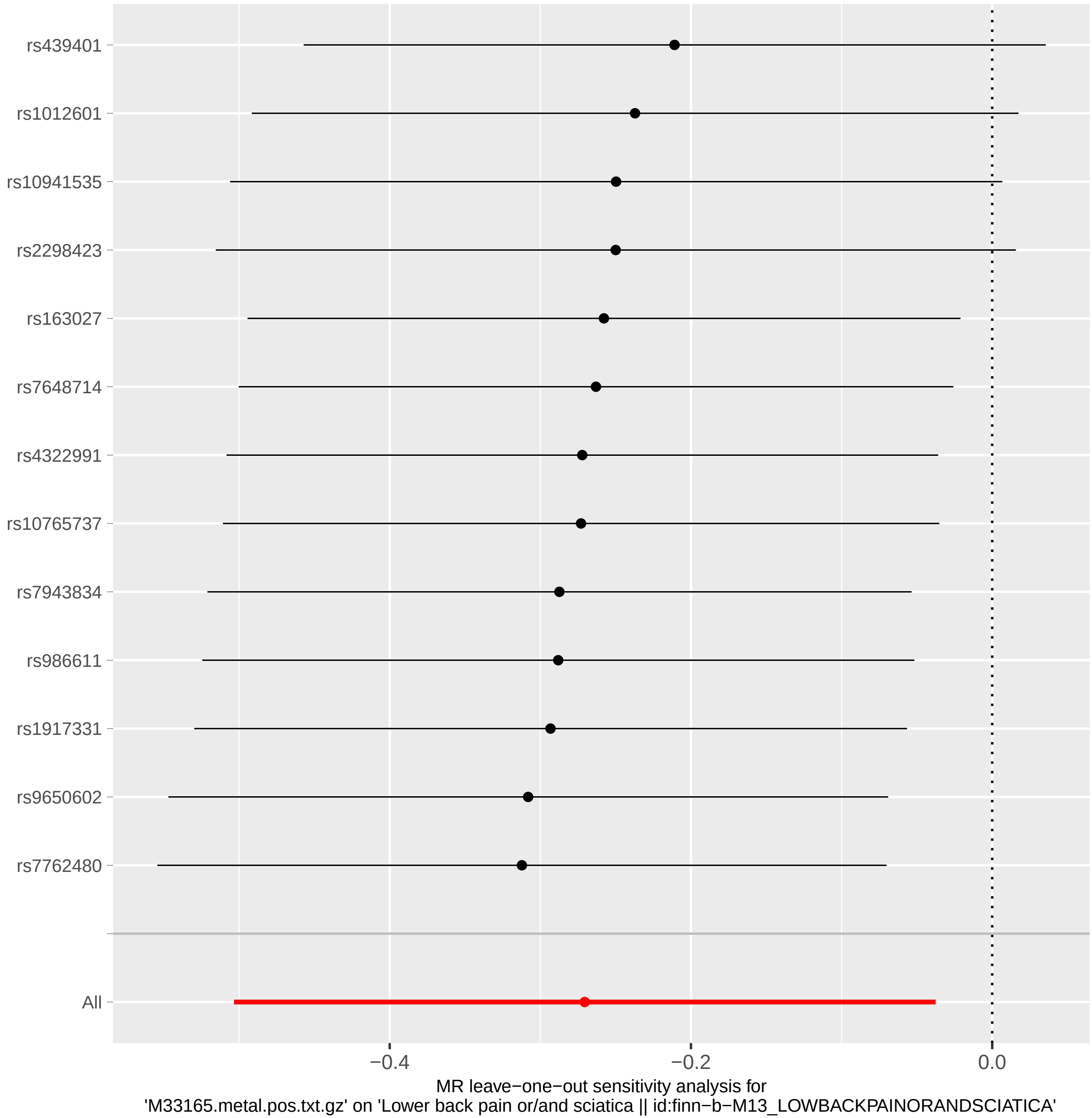
rs439401
rs1012601
rs10941535
rs2298423
rs163027
rs7648714
rs4322991
rs10765737
rs7943834
rs986611
rs1917331
rs9650602
rs7762480
All
−0.4
−0.2
0.0
MR leave−one−out sensitivity analysis for
'M33165.metal.pos.txt.gz' on 'Lower back pain or/and sciatica || id:finn−b−M13_LOWBACKPAINORANDSCIATICA'

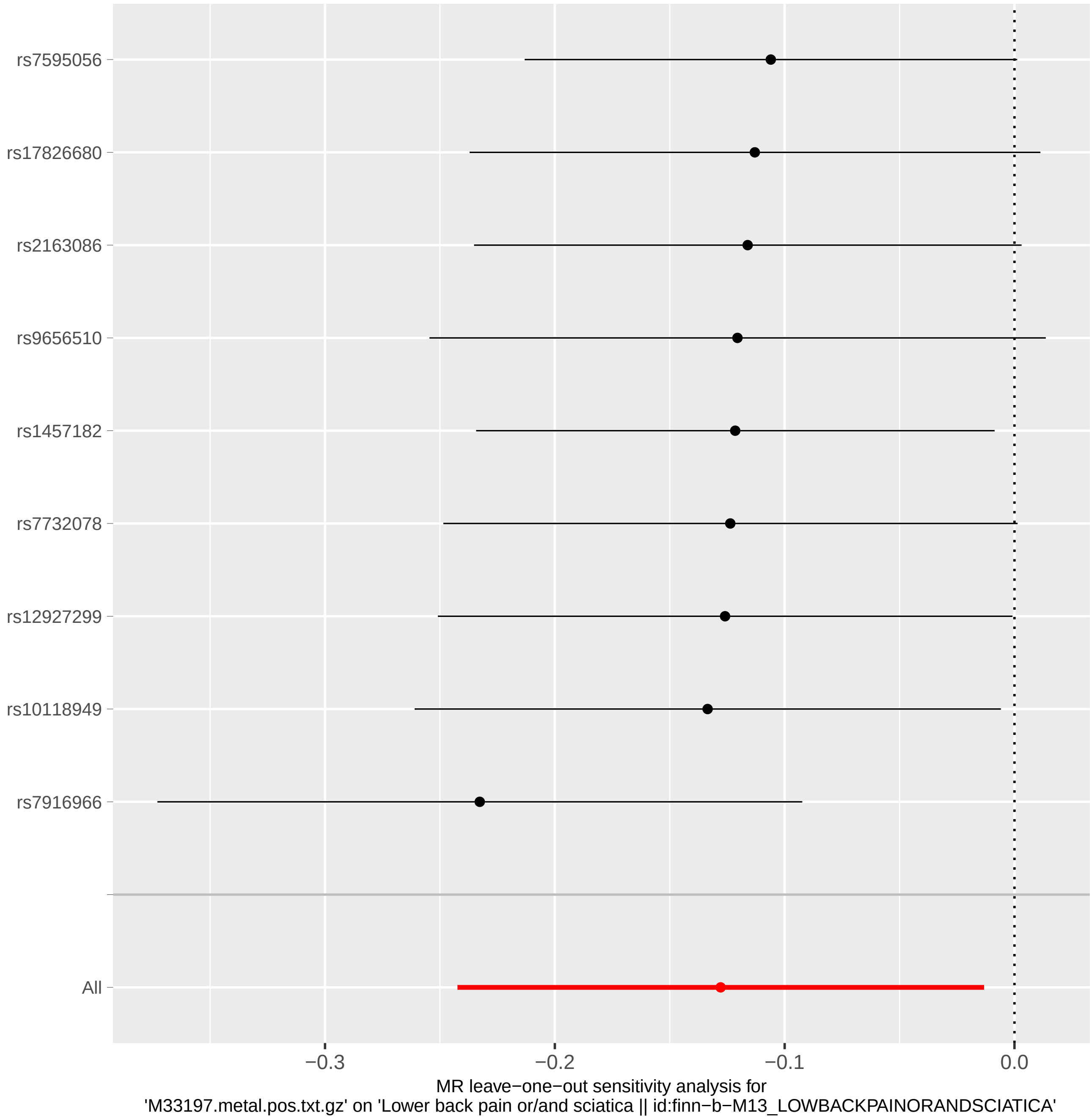
rs7595056
rs17826680
rs2163086
rs9656510
rs1457182
rs7732078
rs12927299
rs10118949
rs7916966
All
−0.3
−0.2
−0.1
0.0
MR leave−one−out sensitivity analysis for
'M33197.metal.pos.txt.gz' on 'Lower back pain or/and sciatica || id:finn−b−M13_LOWBACKPAINORANDSCIATICA'

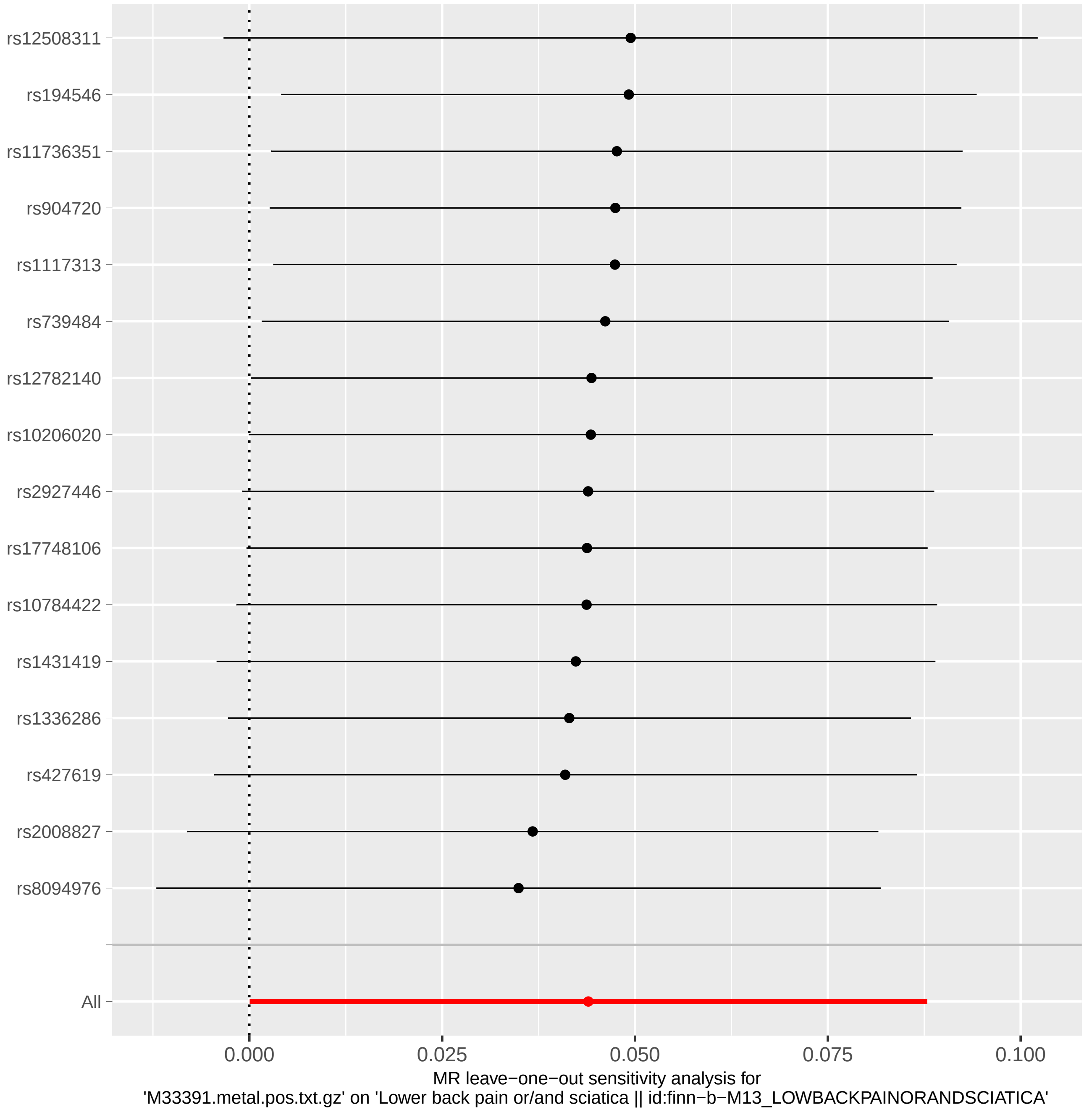
rs12508311
rs194546
rs11736351
rs904720
rs1117313
rs739484
rs12782140
rs10206020
rs2927446
rs17748106
rs10784422
rs1431419
rs1336286
rs427619
rs2008827
rs8094976
All
0.000
0.025
0.050
0.075
0.100
MR leave−one−out sensitivity analysis for
'M33391.metal.pos.txt.gz' on 'Lower back pain or/and sciatica || id:finn−b−M13_LOWBACKPAINORANDSCIATICA'

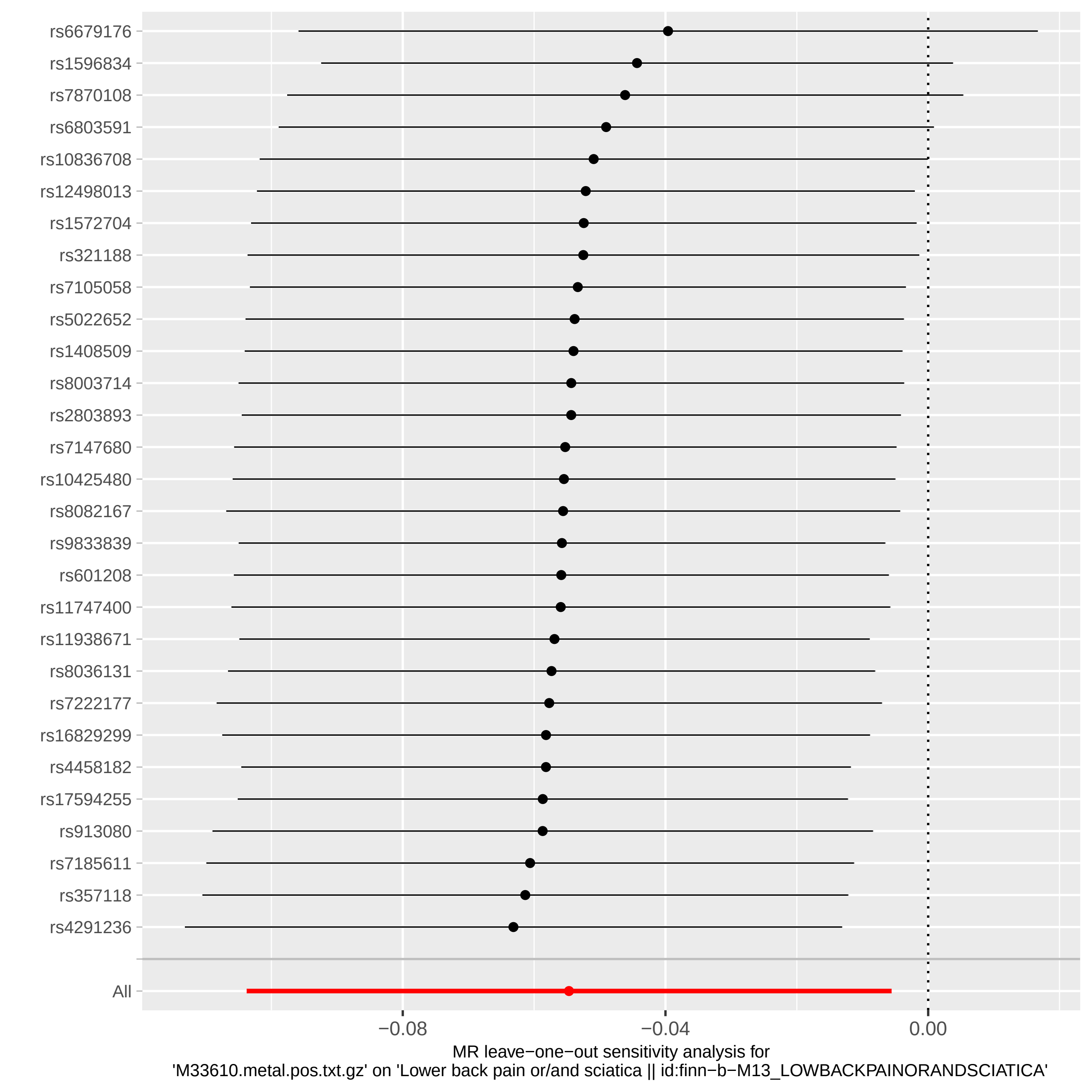
rs6679176
rs1596834
rs7870108
rs6803591
rs10836708
rs12498013
rs1572704
rs321188
rs7105058
rs5022652
rs1408509
rs8003714
rs2803893
rs7147680
rs10425480
rs8082167
rs9833839
rs601208
rs11747400
rs11938671
rs8036131
rs7222177
rs16829299
rs4458182
rs17594255
rs913080
rs7185611
rs357118
rs4291236
All
−0.08
−0.04
0.00
MR leave−one−out sensitivity analysis for
'M33610.metal.pos.txt.gz' on 'Lower back pain or/and sciatica || id:finn−b−M13_LOWBACKPAINORANDSCIATICA'

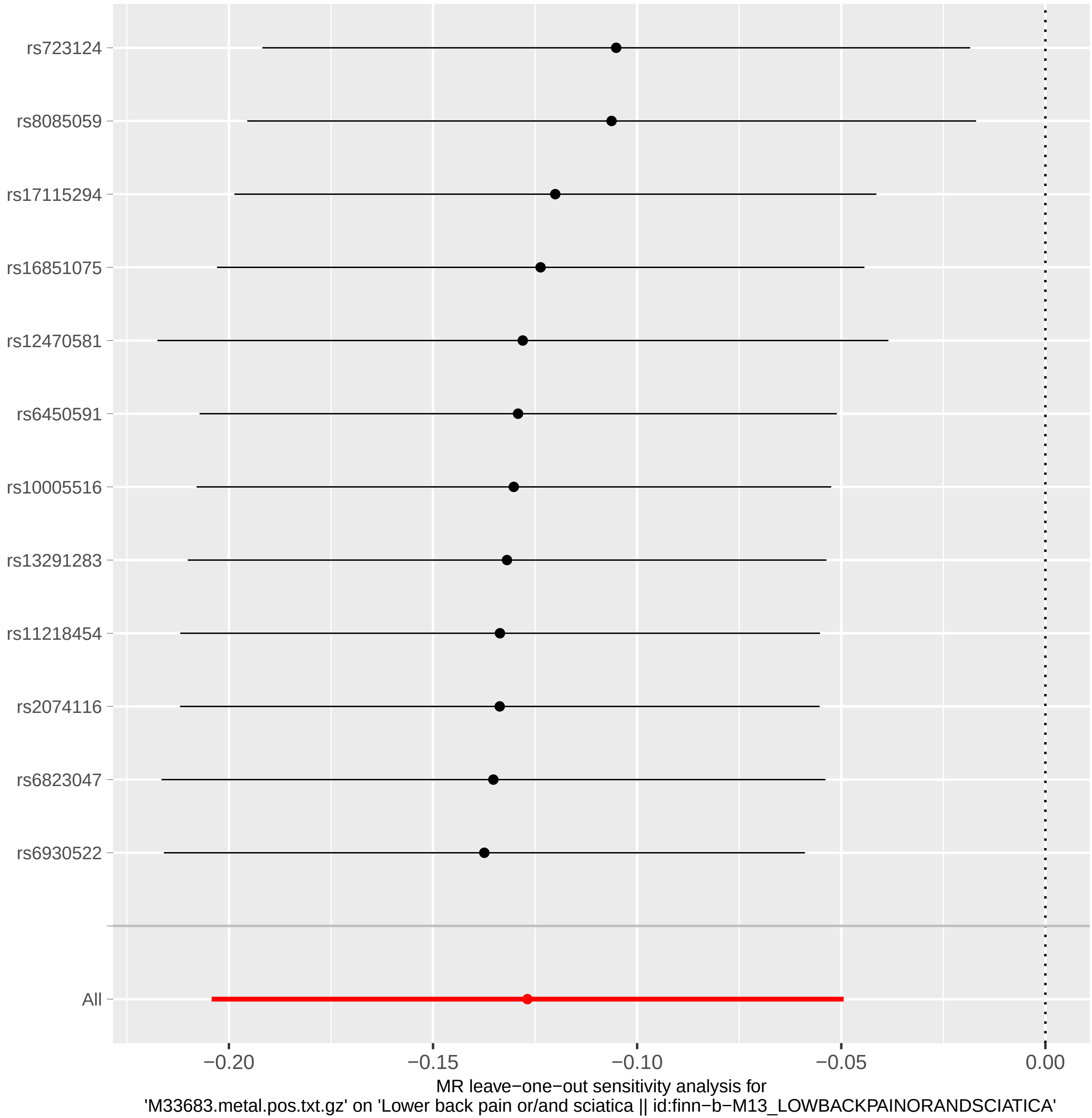
rs723124
rs8085059
rs17115294
rs16851075
rs12470581
rs6450591
rs10005516
rs13291283
rs11218454
rs2074116
rs6823047
rs6930522
All
−0.20
−0.15
−0.10
−0.05
0.00
MR leave−one−out sensitivity analysis for
'M33683.metal.pos.txt.gz' on 'Lower back pain or/and sciatica || id:finn−b−M13_LOWBACKPAINORANDSCIATICA'

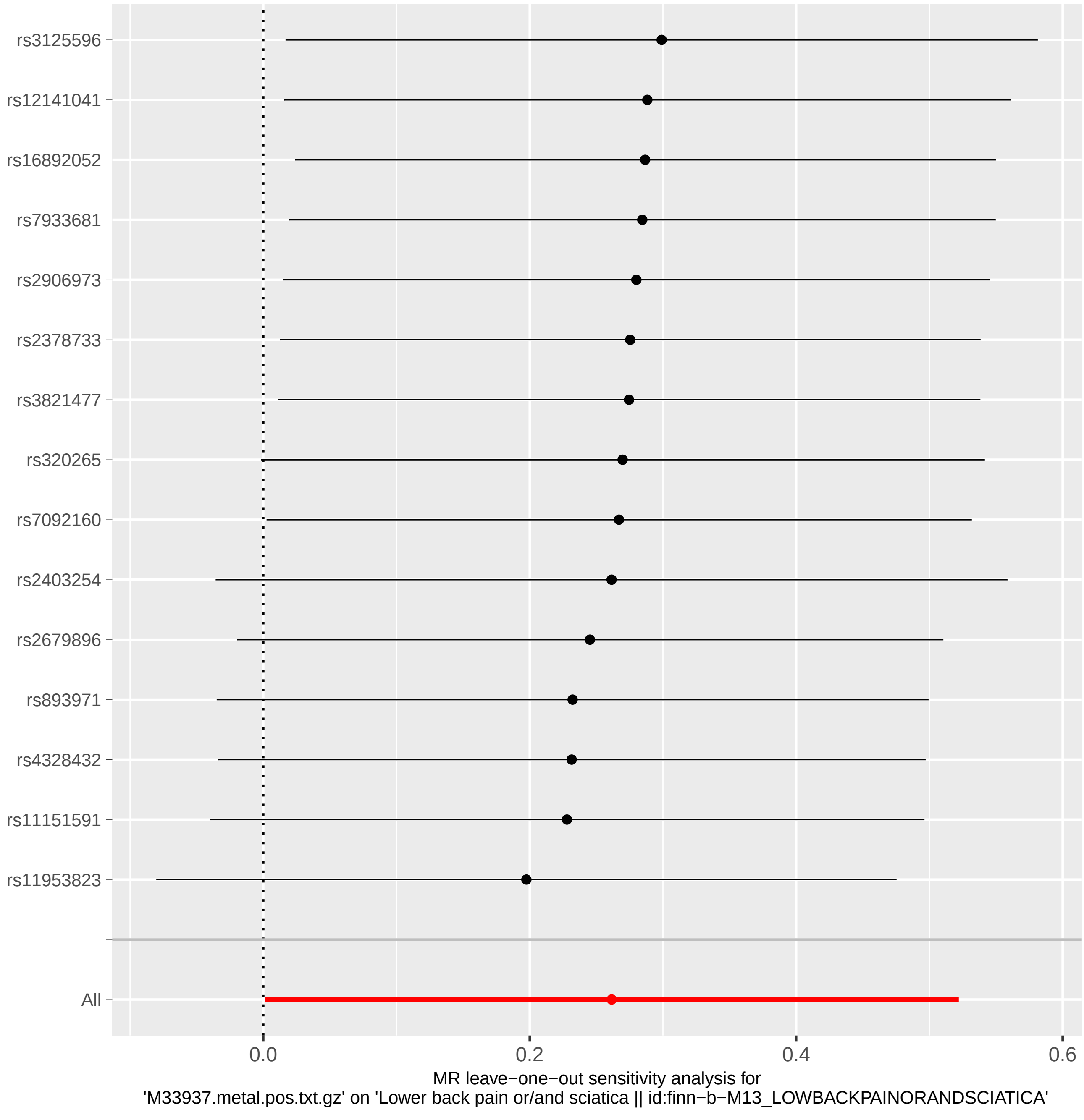

rs3125596
rs12141041
rs16892052
rs7933681
rs2906973
rs2378733
rs3821477
rs320265
rs7092160
rs2403254
rs2679896
rs893971
rs4328432
rs11151591
rs11953823
All
0.0
0.2
0.4
0.6
MR leave−one−out sensitivity analysis for
'M33937.metal.pos.txt.gz' on 'Lower back pain or/and sciatica || id:finn−b−M13_LOWBACKPAINORANDSCIATICA'

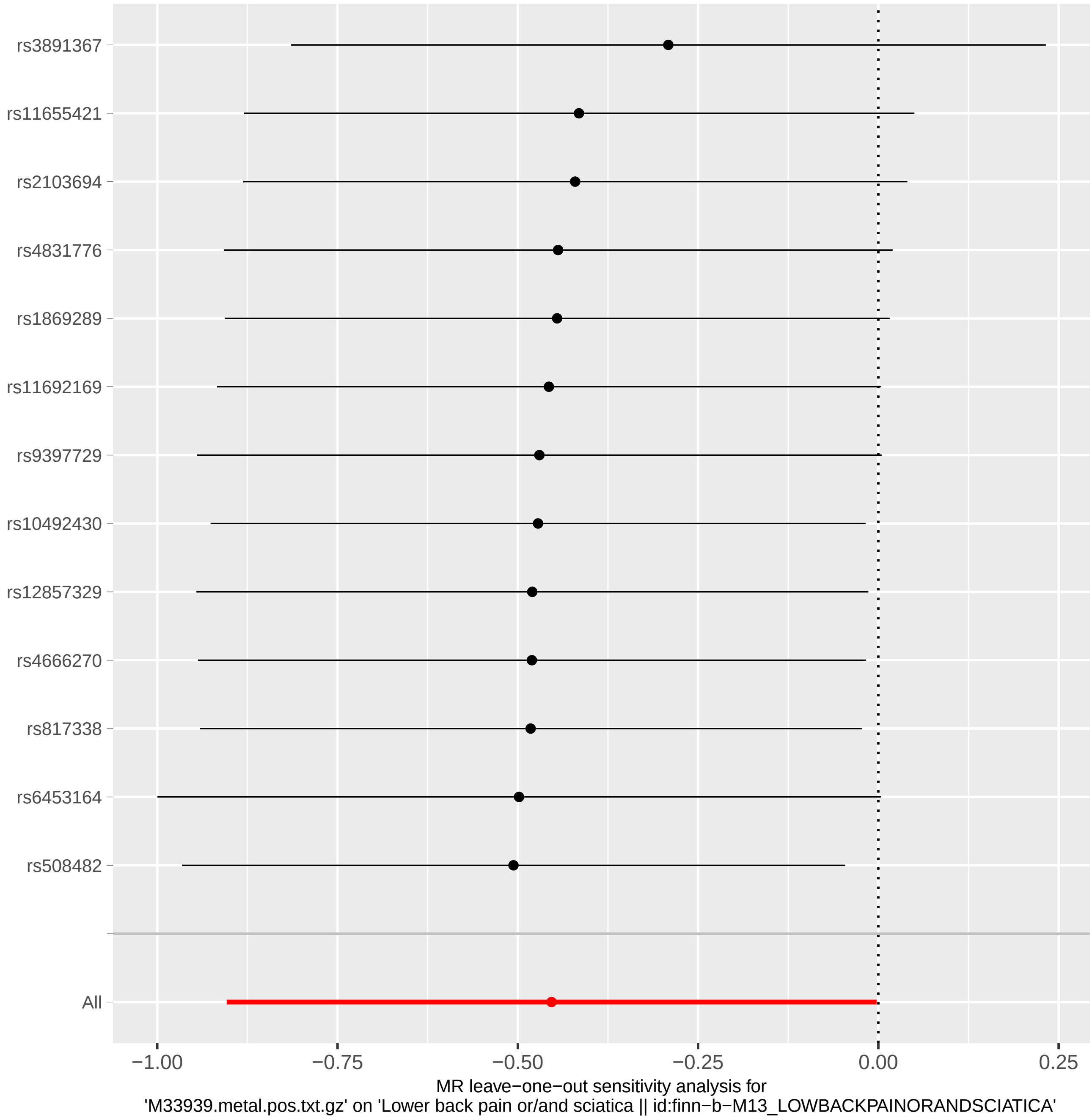
rs3891367
rs11655421
rs2103694
rs4831776
rs1869289
rs11692169
rs9397729
rs10492430
rs12857329
rs4666270
rs817338
rs6453164
rs508482
All
−1.00
−0.75
−0.50
−0.25
0.00
0.25
MR leave−one−out sensitivity analysis for
'M33939.metal.pos.txt.gz' on 'Lower back pain or/and sciatica || id:finn−b−M13_LOWBACKPAINORANDSCIATICA'

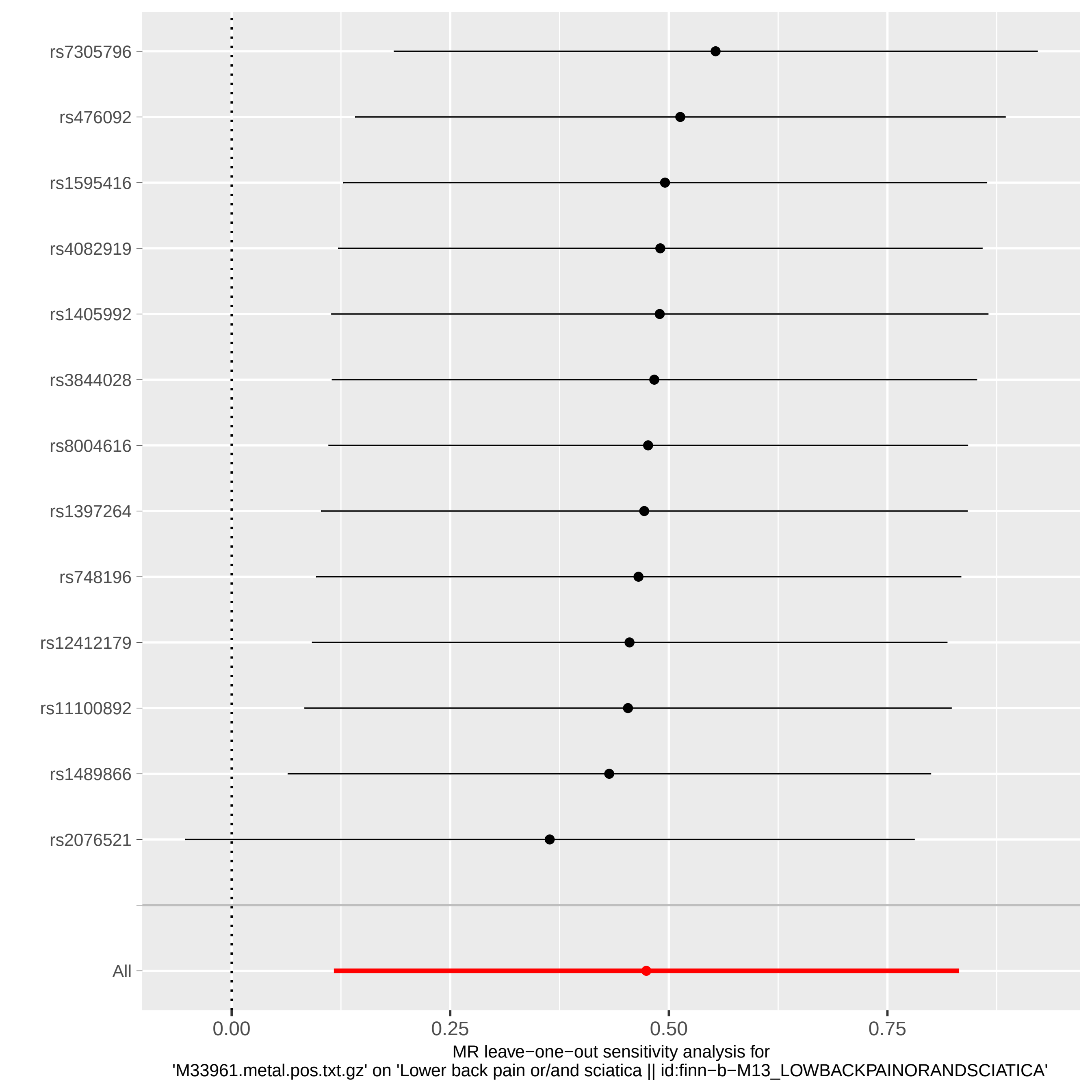

rs7305796
rs476092
rs1595416
rs4082919
rs1405992
rs3844028
rs8004616
rs1397264
rs748196
rs12412179
rs11100892
rs1489866
rs2076521
All
0.00
0.25
0.50
0.75
MR leave−one−out sensitivity analysis for
'M33961.metal.pos.txt.gz' on 'Lower back pain or/and sciatica || id:finn−b−M13_LOWBACKPAINORANDSCIATICA'

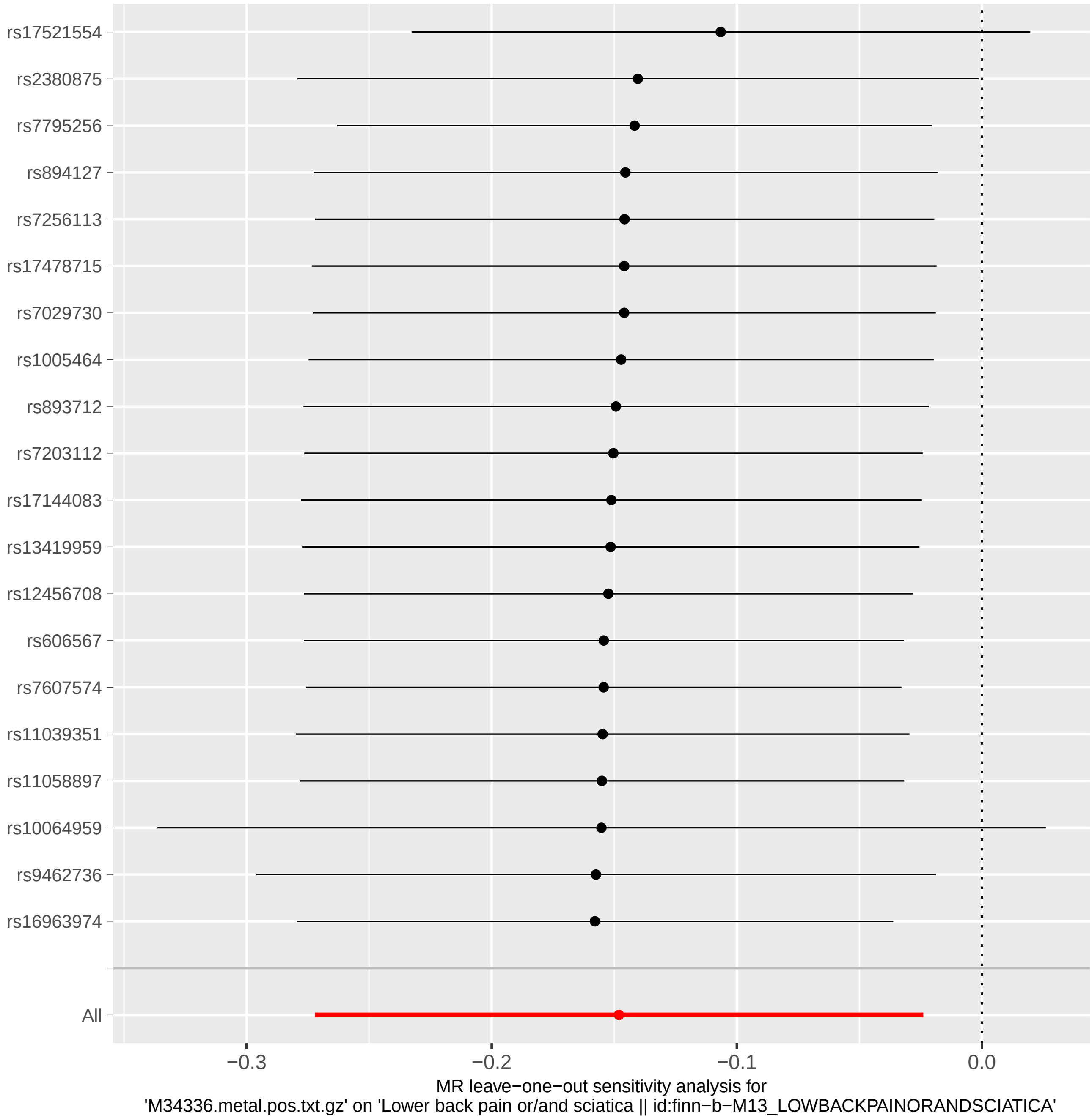
rs17521554
rs2380875
rs7795256
rs894127
rs7256113
rs17478715
rs7029730
rs1005464
rs893712
rs7203112
rs17144083
rs13419959
rs12456708
rs606567
rs7607574
rs11039351
rs11058897
rs10064959
rs9462736
rs16963974
All
−0.3
−0.2
−0.1
0.0
MR leave−one−out sensitivity analysis for
'M34336.metal.pos.txt.gz' on 'Lower back pain or/and sciatica || id:finn−b−M13_LOWBACKPAINORANDSCIATICA'

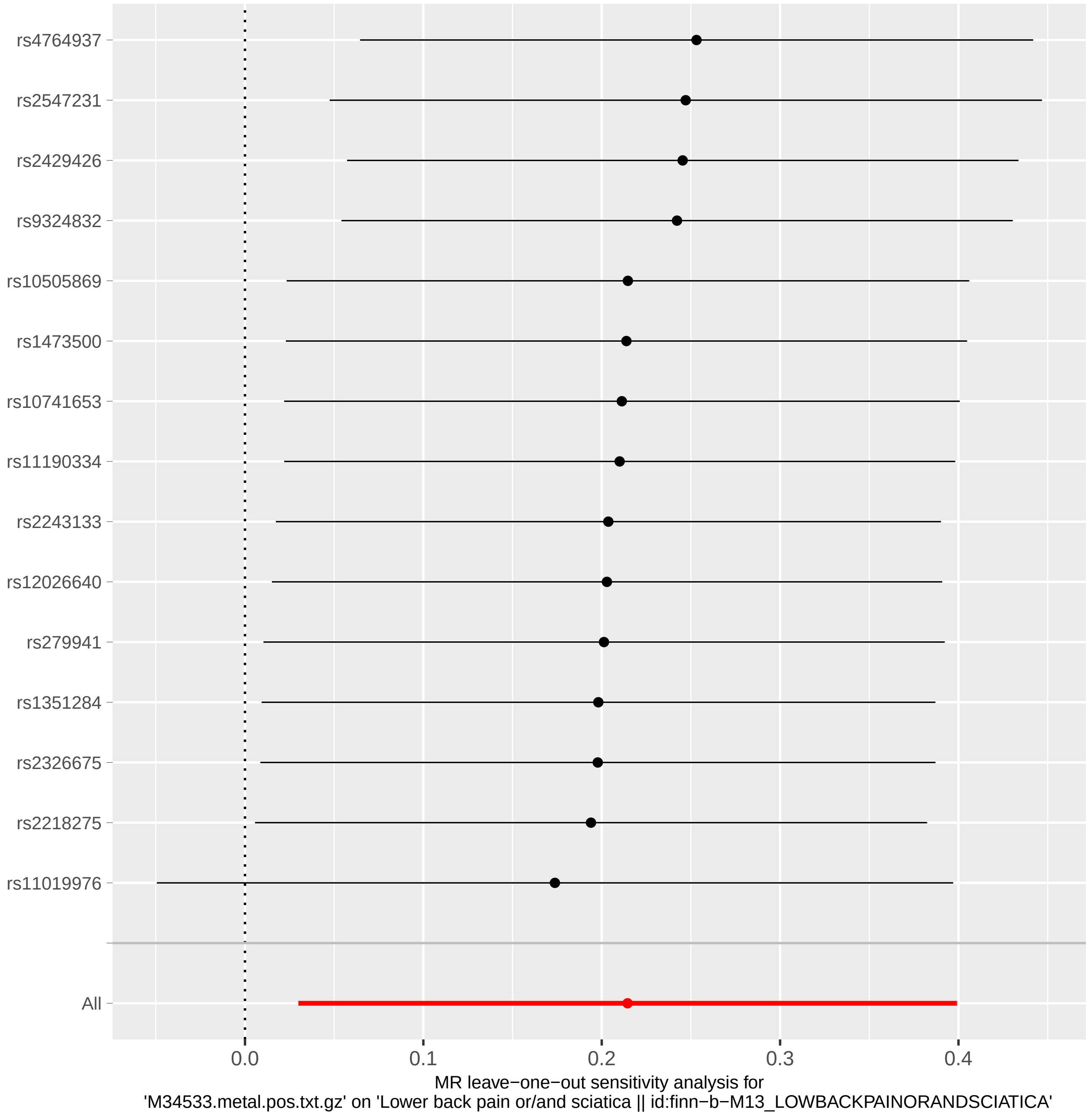

rs4764937
rs2547231
rs2429426
rs9324832
rs10505869
rs1473500
rs10741653
rs11190334
rs2243133
rs12026640
rs279941
rs1351284
rs2326675
rs2218275
rs11019976
All
0.0
0.1
0.2
0.3
0.4
MR leave−one−out sensitivity analysis for
'M34533.metal.pos.txt.gz' on 'Lower back pain or/and sciatica || id:finn−b−M13_LOWBACKPAINORANDSCIATICA'

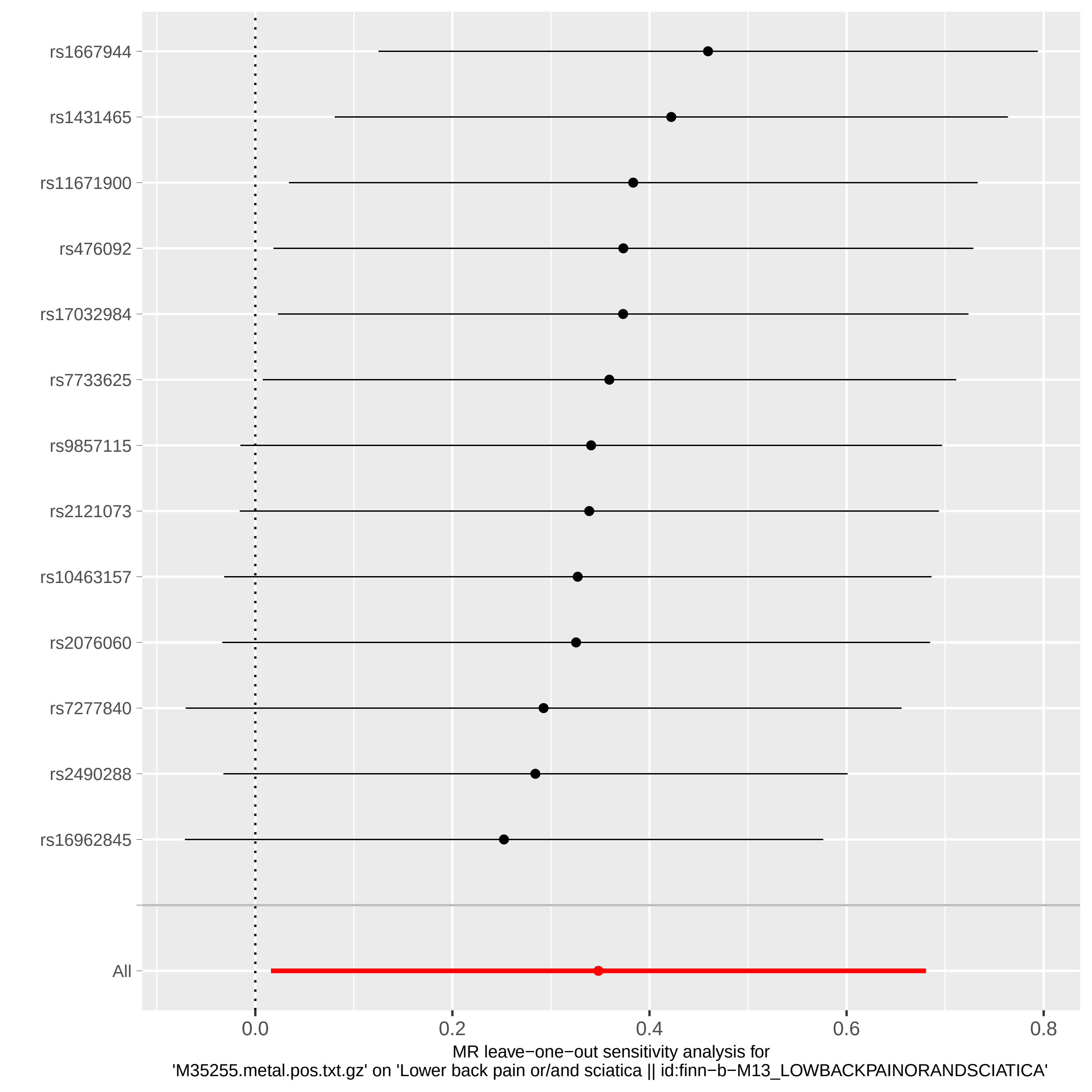
rs1667944
rs1431465
rs11671900
rs476092
rs17032984
rs7733625
rs9857115
rs2121073
rs10463157
rs2076060
rs7277840
rs2490288
rs16962845
All
0.0
0.2
0.4
0.6
0.8
MR leave−one−out sensitivity analysis for
'M35255.metal.pos.txt.gz' on 'Lower back pain or/and sciatica || id:finn−b−M13_LOWBACKPAINORANDSCIATICA'

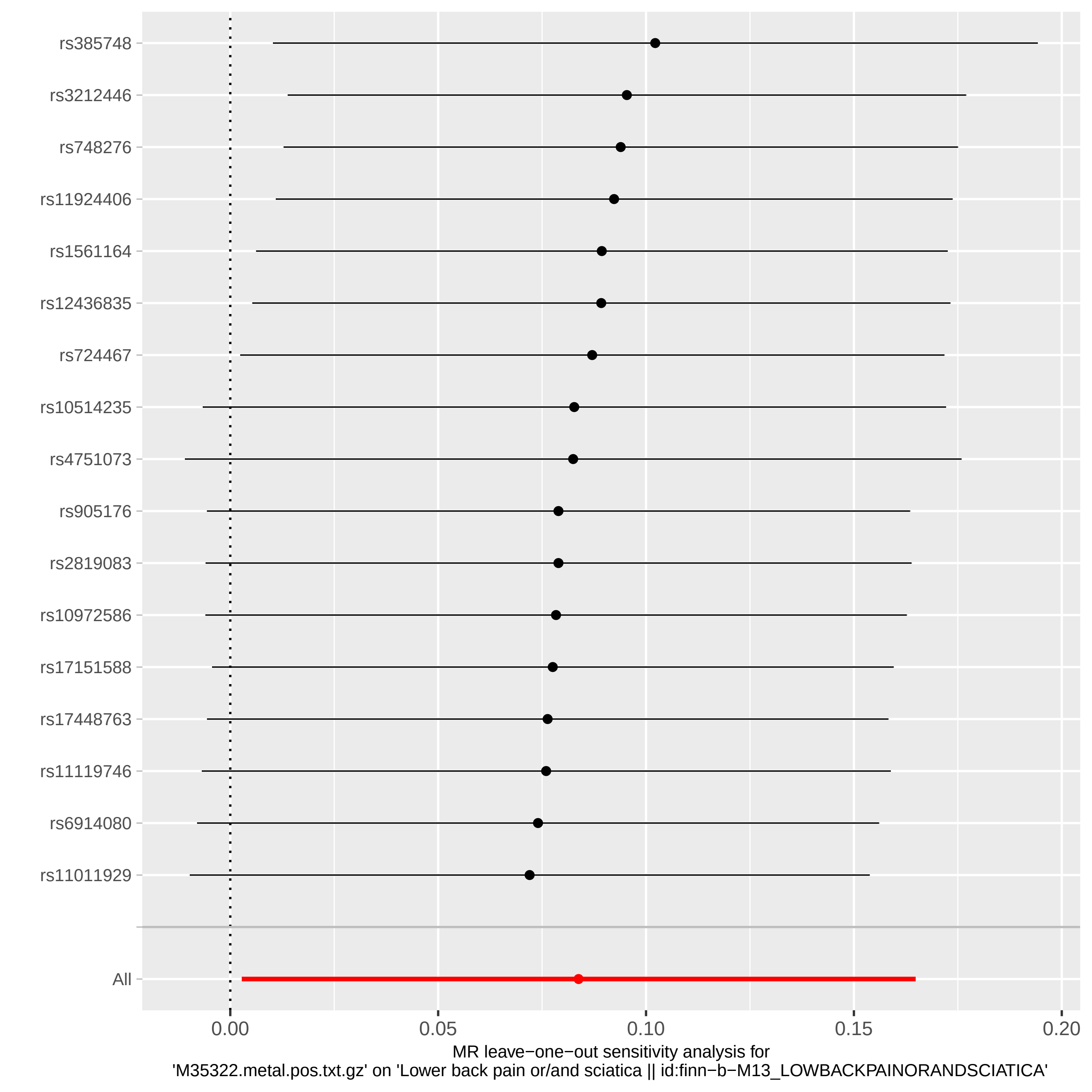
rs385748
rs3212446
rs748276
rs11924406
rs1561164
rs12436835
rs724467
rs10514235
rs4751073
rs905176
rs2819083
rs10972586
rs17151588
rs17448763
rs11119746
rs6914080
rs11011929
All
0.00
0.05
0.10
0.15
0.20
MR leave−one−out sensitivity analysis for
'M35322.metal.pos.txt.gz' on 'Lower back pain or/and sciatica || id:finn−b−M13_LOWBACKPAINORANDSCIATICA'

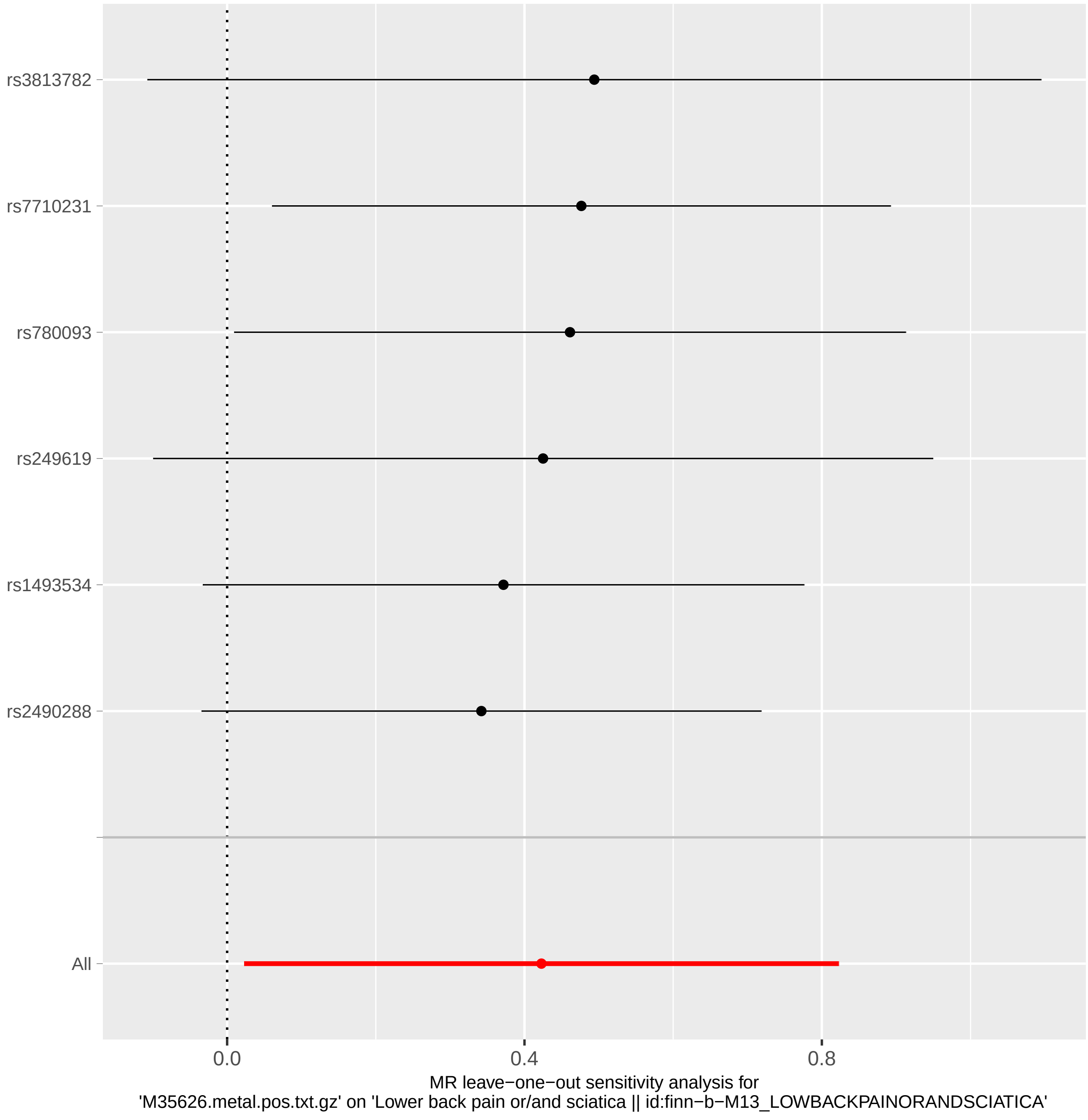

rs3813782
rs7710231
rs780093
rs249619
rs1493534
rs2490288
All
0.0
0.4
0.8
MR leave−one−out sensitivity analysis for
'M35626.metal.pos.txt.gz' on 'Lower back pain or/and sciatica || id:finn−b−M13_LOWBACKPAINORANDSCIATICA'

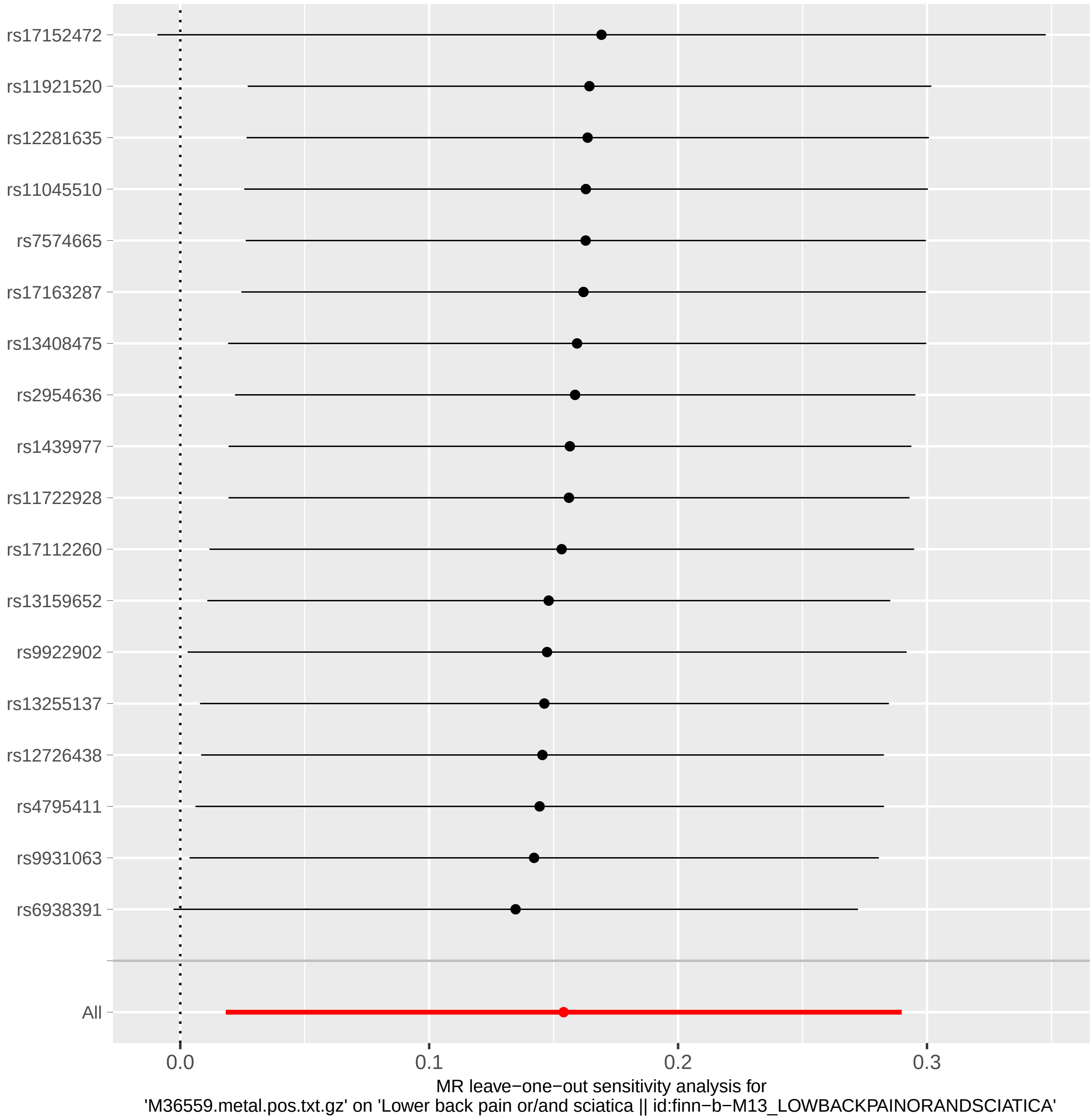
rs17152472
rs11921520
rs12281635
rs11045510
rs7574665
rs17163287
rs13408475
rs2954636
rs1439977
rs11722928
rs17112260
rs13159652
rs9922902
rs13255137
rs12726438
rs4795411
rs9931063
rs6938391
All
0.0
0.1
0.2
0.3
MR leave−one−out sensitivity analysis for
'M36559.metal.pos.txt.gz' on 'Lower back pain or/and sciatica || id:finn−b−M13_LOWBACKPAINORANDSCIATICA'
