# Supplementary material for: Causality of genetically determined serum metabolites on lower back pain or/and sciatica: a comprehensive Mendelian randomized study
Source: Front Pain Res (Lausanne). 2024 Sep 25;5:1370704. doi: 10.3389/fpain.2024.1370704 (PMC11461461; doi:10.3389/fpain.2024.1370704)
Supplement: Supplementary Table S1 — The STROBE-MR reporting specification. [file Table1.docx]

| Table 1. Causal effect of 28 metabolites on the risk of sciatica or/and lower back pain derived from IVW. | | | | | | | | |
| --- | --- | --- | --- | --- | --- | --- | --- | --- |
| Metabolite | Status | ID | Super-pathway | nsnp | Methods | P-value | OR(95% CI) | P_ivw_fdr |
| tyrosine | Known | M01299 | Amino acid | 34 | IVW | 0.012722851 | 1.94 (1.15- 3.25) | 0.04971104 |
| malate | Known | M01303 | Energy | 17 | IVW | 0.031354846 | 1.62 (1.04- 2.51) | 0.04971104 |
| pentadecanoate (15:0) | Known | M01361 | Lipid | 19 | IVW | 0.037335145 | 1.52 (1.02-2.26) | 0.04971104 |
| X-03088 | Unknown | M12768 |  | 18 | IVW | 0.010528692 | 0.66 (0.48-0.91) | 0.04971104 |
| benzoate | Known | M15778 | Xenobiotics | 41 | IVW | 0.022693254 | 1.58 (1.07-2.34) | 0.04971104 |
| aspartate | Known | M15996 | Amino acid | 4 | IVW | 0.047249796 | 1.72 (1.01- 2.92) | 0.04971104 |
| 1,5-anhydroglucitol (1,5-AG) | Known | M20675 | Carbohydrate | 31 | IVW | 0.025052913 | 1.38 (1.04-1.84) | 0.04971104 |
| 1-palmitoylglycerol (1-monopalmitin) | Known | M21127 | Lipid | 13 | IVW | 0.043766282 | 1.55 (1.01- 2.36) | 0.04971104 |
| levulinate (4-oxovalerate) | Known | M22177 | Amino acid | 58 | IVW | 0.041658466 | 0.71 (0.52-0.99) | 0.04971104 |
| glycine | Known | M32338 | Amino acid | 26 | IVW | 0.006365201 | 1.38 (1.10-1.74) | 0.04971104 |
| 3-methylxanthine | Known | M32445 | Xenobiotics | 14 | IVW | 0.042353222 | 0.81 (0.66-0.99) | 0.04971104 |
| C-glycosyltryptophan* | Known | M32675 | Amino acid | 23 | IVW | 0.014432888 | 2.25 (1.17- 4.29) | 0.04971104 |
| X-11445--5-alpha-pregnan-3beta,20alpha-disulfate | Identified | M32762 | Lipid | 15 | IVW | 0.032827454 | 1.14 (1.01-1.29) | 0.04971104 |
| adrenate (22:4n6) | Known | M32980 | Lipid | 11 | IVW | 0.021400436 | 0.59 (0.38-0.93) | 0.04971104 |
| X-11820 | Unknown | M33165 |  | 13 | IVW | 0.022723437 | 0.76 (0.60-0.96) | 0.04971104 |
| X-11852 | Unknown | M33197 |  | 9 | IVW | 0.028584098 | 0.88 (0.78-0.99) | 0.04971104 |
| X-12040 | Unknown | M33391 |  | 16 | IVW | 0.049711039 | 1.04 (1.00-1.09) | 0.04971104 |
| X-12189 | Unknown | M33610 |  | 29 | IVW | 0.028910647 | 0.95 (0.90-0.99) | 0.04971104 |
| X-12261 | Unknown | M33683 |  | 12 | IVW | 0.001304213 | 0.88 (0.82-0.95) | 0.03651797 |
| alpha-hydroxyisovalerate | Known | M33937 | Amino acid | 15 | IVW | 0.049135231 | 1.30 (1.00-1.69) | 0.04971104 |
| N-acetylthreonine | Known | M33939 | Amino acid | 13 | IVW | 0.048624068 | 0.64 (0.41-1.00) | 0.04971104 |
| 1-stearoylglycerophosphocholine | Known | M33961 | Lipid | 13 | IVW | 0.009284667 | 1.61 (1.12-2.30) | 0.04971104 |
| X-12726 | Unknown | M34336 |  | 20 | IVW | 0.019264956 | 0.86 (0.76-0.98) | 0.04971104 |
| X-12850 | Unknown | M34533 |  | 15 | IVW | 0.022709535 | 1.24 (1.03-1.49) | 0.04971104 |
| 2-stearoylglycerophosphocholine* | Known | M35255 | Lipid | 13 | IVW | 0.039937189 | 1.42 (1.02-1.97) | 0.04971104 |
| hydroquinone sulfate | Known | M35322 | Xenobiotics | 17 | IVW | 0.04261585 | 1.09 (1.00-1.18) | 0.04971104 |
| 1-myristoylglycerophosphocholine | Known | M35626 | Lipid | 6 | IVW | 0.038201587 | 1.53 (1.02-2.28) | 0.04971104 |
| X-14632 | Unknown | M36559 |  | 18 | IVW | 0.026127176 | 1.17 (1.02-1.34) | 0.04971104 |

IVW, Inverse variance weighted; OR, odds ratio; CI, confidence interval; fdr, false discovery rate.
