# Supplementary material for: Causality of genetically determined serum metabolites on lower back pain or/and sciatica: a comprehensive Mendelian randomized study
Source: Front Pain Res (Lausanne). 2024 Sep 25;5:1370704. doi: 10.3389/fpain.2024.1370704 (PMC11461461; doi:10.3389/fpain.2024.1370704)
Supplement: Supplementary Table S2 — The information on instrumental variables and SNPs identified by the Mendelian randomization estimates. [file Table2.docx]

| Table 2. Five MR models estimate the causal relationships between 17 known metabolites and the risk of sciatica or/and lower back pain and tests for heterogeneity and horizontal pleiotropy. | | | | | | | | |
| --- | --- | --- | --- | --- | --- | --- | --- | --- |
| Metabolite | Methods | SNP (N) | OR (95% CI) | P | Heterogeneity | P | Pleiotropy | P |
|  |  |  |  |  | Q value (I^2^) |  | Intercept |  |
| tyrosine | IVW | 34 | 1.94(1.15- 3.25) | 0.0127 | 41.137532 | 0.1562 | -0.00624461 | 0.5391 |
|  | WM | 34 | 2.19(1.11- 4.32) | 0.0233 |  |  |  |  |
|  | MR Egger | 34 | 3.60(0.47-27.25) | 0.2245 | 40.64795696 | 0.1404 |  |  |
|  | Simple mode | 34 | 3.74(1.16-12.10) | 0.0345 |  |  |  |  |
|  | Weighted mode | 34 | 2.89(1.06- 7.86) | 0.0458 |  |  |  |  |
| malate | IVW | 17 | 1.62(1.04- 2.51) | 0.0313 | 16.94279894 | 0.3893 | -0.00428789 | 0.5391 |
|  | WM | 17 | 1.38(0.74- 2.57) | 0.3099 |  |  |  |  |
|  | MR Egger | 17 | 2.13(0.38-11.97) | 0.4035 | 16.82488428 | 0.3294 |  |  |
|  | Simple mode | 17 | 0.79(0.22- 2.83) | 0.7202 |  |  |  |  |
|  | Weighted mode | 17 | 0.75(0.22- 2.57) | 0.6478 |  |  |  |  |
| pentadecanoate (15:0) | IVW | 19 | 1.52(1.02-2.26) | 0.0373 | 16.82066286 | 0.5354 | 0.00390550 | 0.6647 |
|  | WM | 19 | 1.38(0.78-2.44) | 0.2747 |  |  |  |  |
|  | MR Egger | 19 | 1.22(0.41-3.56) | 0.7260 | 16.62615904 | 0.4799 |  |  |
|  | Simple mode | 19 | 1.30(0.50-3.38) | 0.5937 |  |  |  |  |
|  | Weighted mode | 19 | 1.27(0.55-2.91) | 0.5792 |  |  |  |  |
| benzoate | IVW | 41 | 1.58(1.07-2.34) | 0.0226 | 43.06676712 | 0.3414 | 0.00487946 | 0.4455 |
|  | WM | 41 | 1.07(0.60-1.91) | 0.8187 |  |  |  |  |
|  | MR Egger | 41 | 1.05(0.34-3.20) | 0.9383 | 42.42081944 | 0.3257 |  |  |
|  | Simple mode | 41 | 1.02(0.27-3.85) | 0.9743 |  |  |  |  |
|  | Weighted mode | 41 | 0.92(0.36-2.37) | 0.8642 |  |  |  |  |
| aspartate | IVW | 4 | 1.72(1.01- 2.92) | 0.0472 | 1.677104801 | 0.6420 | -0.01298943 | 0.6053 |
|  | WM | 4 | 1.80(0.88- 3.68) | 0.1093 |  |  |  |  |
|  | MR Egger | 4 | 2.66(0.59-12.04) | 0.3322 | 1.308132089 | 0.5199 |  |  |
|  | Simple mode | 4 | 1.8 (0.71- 4.63) | 0.3009 |  |  |  |  |
|  | Weighted mode | 4 | 2.05(0.86- 4.87) | 0.2035 |  |  |  |  |
| 1,5-anhydroglucitol (1,5-AG) | IVW | 31 | 1.38(1.04-1.84) | 0.0250 | 40.20802274 | 0.1008 | -0.00746007 | 0.2695 |
|  | WM | 31 | 1.64(1.16-2.32) | 0.0053 |  |  |  |  |
|  | MR Egger | 31 | 1.97(1.00-3.86) | 0.0587 | 38.52508271 | 0.1110 |  |  |
|  | Simple mode | 31 | 1.75(0.89-3.40) | 0.1127 |  |  |  |  |
|  | Weighted mode | 31 | 1.72(1.14-2.61) | 0.0158 |  |  |  |  |
| 1-palmitoylglycerol (1-monopalmitin) | IVW | 13 | 1.55(1.01- 2.36) | 0.0437 | 9.177034605 | 0.6877 | -0.01080479 | 0.3418 |
|  | WM | 13 | 1.95(1.08- 3.53) | 0.0278 |  |  |  |  |
|  | MR Egger | 13 | 2.96(0.77-11.43) | 0.1432 | 8.190221082 | 0.6961 |  |  |
|  | Simple mode | 13 | 1.98(0.89- 4.43) | 0.1218 |  |  |  |  |
|  | Weighted mode | 13 | 1.98(1.00- 3.92) | 0.0735 |  |  |  |  |
| levulinate (4-oxovalerate) | IVW | 58 | 0.71(0.52-0.99) | 0.0416 | 52.28226369 | 0.6523 | -0.00262907 | 0.6129 |
|  | WM | 58 | 0.74(0.46-1.20) | 0.2175 |  |  |  |  |
|  | MR Egger | 58 | 0.91(0.34-2.45) | 0.8540 | 52.02351508 | 0.6261 |  |  |
|  | Simple mode | 58 | 1.37(0.48-3.92) | 0.5590 |  |  |  |  |
|  | Weighted mode | 58 | 1.21(0.49-3.00) | 0.6879 |  |  |  |  |
| glycine | IVW | 26 | 1.38(1.10-1.74) | 0.0063 | 23.82460719 | 0.5295 | -0.00230829 | 0.5915 |
|  | WM | 26 | 1.29(0.96-1.72) | 0.0924 |  |  |  |  |
|  | MR Egger | 26 | 1.50(1.03-2.17) | 0.0436 | 23.52878324 | 0.4887 |  |  |
|  | Simple mode | 26 | 1.50(0.72-3.12) | 0.2836 |  |  |  |  |
|  | Weighted mode | 26 | 1.31(0.98-1.76) | 0.0830 |  |  |  |  |
| 3-methylxanthine | IVW | 14 | 0.81(0.66-0.99) | 0.0423 | 11.22630354 | 0.5918 | 0.01109903 | 0.3165 |
|  | WM | 14 | 0.74(0.56-0.98) | 0.0357 |  |  |  |  |
|  | MR Egger | 14 | 0.61(0.35-1.07) | 0.1128 | 10.13397786 | 0.6042 |  |  |
|  | Simple mode | 14 | 0.72(0.47-1.11) | 0.1598 |  |  |  |  |
|  | Weighted mode | 14 | 0.72(0.53-1.00) | 0.0708 |  |  |  |  |
| C-glycosyltryptophan* | IVW | 23 | 2.25(1.17- 4.29) | 0.0144 | 25.18286258 | 0.2884 | -0.00822356 | 0.4247 |
|  | WM | 23 | 4.27(1.77-10.29) | 0.0012 |  |  |  |  |
|  | MR Egger | 23 | 4.61(0.73-29.25) | 0.1203 | 24.41234694 | 0.2735 |  |  |
|  | Simple mode | 23 | 3.46(0.72-16.71) | 0.1361 |  |  |  |  |
|  | Weighted mode | 23 | 4.04(1.49-10.99) | 0.0120 |  |  |  |  |
| alpha-hydroxyisovalerate | IVW | 15 | 1.30(1.00-1.69) | 0.0491 | 10.22633811 | 0.7454 | 0.01512764 | 0.1892 |
|  | WM | 15 | 1.22(0.85-1.74) | 0.2789 |  |  |  |  |
|  | MR Egger | 15 | 0.79(0.38-1.67) | 0.5547 | 8.307204266 | 0.8230 |  |  |
|  | Simple mode | 15 | 0.95(0.55-1.66) | 0.8710 |  |  |  |  |
|  | Weighted mode | 15 | 1.17(0.77-1.80) | 0.4736 |  |  |  |  |
| N-acetylthreonine | IVW | 13 | 0.64(0.41-1.00) | 0.0486 | 4.628821118 | 0.9692 | 0.01067013 | 0.3897 |
|  | WM | 13 | 0.72(0.39-1.32) | 0.2853 |  |  |  |  |
|  | MR Egger | 13 | 0.37(0.10-1.33) | 0.1549 | 3.82715867 | 0.9747 |  |  |
|  | Simple mode | 13 | 0.71(0.30-1.72) | 0.4661 |  |  |  |  |
|  | Weighted mode | 13 | 0.73(0.34-1.56) | 0.4304 |  |  |  |  |
| 1-stearoylglycerophosphocholine | IVW | 13 | 1.61(1.12-2.30) | 0.0092 | 5.960087592 | 0.9180 | -0.00420964 | 0.6361 |
|  | WM | 13 | 1.63(0.98-2.71) | 0.0597 |  |  |  |  |
|  | MR Egger | 13 | 1.89(0.90-3.94) | 0.1197 | 5.723386705 | 0.8911 |  |  |
|  | Simple mode | 13 | 1.51(0.71-3.20) | 0.3076 |  |  |  |  |
|  | Weighted mode | 13 | 1.74(0.92-3.28) | 0.1132 |  |  |  |  |
| 2-stearoylglycerophosphocholine* | IVW | 13 | 1.42(1.02-1.97) | 0.0399 | 13.83377936 | 0.3114 | -0.00361926 | 0.8609 |
|  | WM | 13 | 1.34(0.86-2.10) | 0.2005 |  |  |  |  |
|  | MR Egger | 13 | 1.65(0.30-9.14) | 0.5772 | 13.79345246 | 0.2446 |  |  |
|  | Simple mode | 13 | 1.04(0.45-2.40) | 0.9287 |  |  |  |  |
|  | Weighted mode | 13 | 0.89(0.39-2.05) | 0.7973 |  |  |  |  |
| hydroquinone sulfate | IVW | 17 | 1.09(1.00-1.18) | 0.0426 | 16.28328708 | 0.4333 | 0.00265168 | 0.6987 |
|  | WM | 17 | 1.09(0.97-1.23) | 0.1294 |  |  |  |  |
|  | MR Egger | 17 | 1.06(0.92-1.23) | 0.4230 | 16.11607698 | 0.3743 |  |  |
|  | Simple mode | 17 | 1.24(1.04-1.48) | 0.0297 |  |  |  |  |
|  | Weighted mode | 17 | 1.08(0.96-1.22) | 0.2353 |  |  |  |  |
| 1-myristoylglycerophosphocholine | IVW | 6 | 1.53(1.02-2.28) | 0.0382 | 5.957023329 | 0.3104 | 0.00578761 | 0.7165 |
|  | WM | 6 | 1.43(0.90-2.30) | 0.1334 |  |  |  |  |
|  | MR Egger | 6 | 1.27(0.46-3.53) | 0.6695 | 5.73908646 | 0.2194 |  |  |
|  | Simple mode | 6 | 1.20(0.65-2.24) | 0.5836 |  |  |  |  |
|  | Weighted mode | 6 | 1.37(0.88-2.14) | 0.2238 |  |  |  |  |

MR, Mendelian randomization; SNP, single nucleotide polymorphism; WM, weighted median;

IVW, inverse variance weighted; OR, odds ratio; CI, confidence interval.
