# Supplementary material for: Causality of genetically determined serum metabolites on lower back pain or/and sciatica: a comprehensive Mendelian randomized study
Source: Front Pain Res (Lausanne). 2024 Sep 25;5:1370704. doi: 10.3389/fpain.2024.1370704 (PMC11461461; doi:10.3389/fpain.2024.1370704)
Supplement: Supplementary Table S3 — The all p-values after FDR corrections of all 486 serum metabolites. [file Table3.docx]

| Table 3. Complementary reverse MR analyses of sciatica or/and lower back pain for causal association with blood metabolites. | | | | | | | | |
| --- | --- | --- | --- | --- | --- | --- | --- | --- |
| method | nsnp | b | se | p-val | or | or_lci95 | or_uci95 | metabolite |
| MR Egger | 5 | -0.0854972 | 0.19588729 | 0.69200543 | 0.91805572 | 0.6253547 | 1.34775721 | tyrosine |
| Weighted median | 5 | -0.0023432 | 0.02254382 | 0.91721674 | 0.99765953 | 0.95453679 | 1.04273042 | tyrosine |
| Inverse variance weighted | 5 | -0.0039803 | 0.01796736 | 0.82467866 | 0.99602758 | 0.96156187 | 1.03172865 | tyrosine |
| Simple mode | 5 | -0.0358256 | 0.0349957 | 0.36384022 | 0.96480855 | 0.90084941 | 1.0333087 | tyrosine |
| Weighted mode | 5 | 0.00381592 | 0.02988523 | 0.9045594 | 1.00382321 | 0.94671316 | 1.06437841 | tyrosine |
| MR Egger | 5 | 0.33504399 | 0.3862299 | 0.44950429 | 1.39800189 | 0.65575593 | 2.98039131 | malate |
| Weighted median | 5 | -0.0044629 | 0.03783116 | 0.90609198 | 0.99554703 | 0.92439853 | 1.07217164 | malate |
| Inverse variance weighted | 5 | -0.0155535 | 0.0398748 | 0.69649392 | 0.98456686 | 0.91054852 | 1.06460213 | malate |
| Simple mode | 5 | -0.021905 | 0.04830062 | 0.67369343 | 0.97833319 | 0.88996406 | 1.07547693 | malate |
| Weighted mode | 5 | 0.00166031 | 0.04030993 | 0.96911937 | 1.00166169 | 0.92556848 | 1.08401071 | malate |
| MR Egger | 5 | -0.177975 | 0.31347336 | 0.60991571 | 0.83696337 | 0.45276468 | 1.54717829 | pentadecanoate (15:0) |
| Weighted median | 5 | -0.0511608 | 0.03564277 | 0.15117991 | 0.95012589 | 0.88601569 | 1.01887497 | pentadecanoate (15:0) |
| Inverse variance weighted | 5 | -0.0211619 | 0.0301038 | 0.48207703 | 0.9790604 | 0.92296369 | 1.03856661 | pentadecanoate (15:0) |
| Simple mode | 5 | -0.0466761 | 0.05043052 | 0.40706366 | 0.95439647 | 0.86457266 | 1.05355243 | pentadecanoate (15:0) |
| Weighted mode | 5 | -0.0434617 | 0.04680136 | 0.40563986 | 0.95746927 | 0.8735479 | 1.04945292 | pentadecanoate (15:0) |
| MR Egger | 5 | -0.4961554 | 0.32110831 | 0.22002515 | 0.60886701 | 0.3244812 | 1.14249775 | X-03088 |
| Weighted median | 5 | -0.0557931 | 0.03804009 | 0.14246014 | 0.94573483 | 0.87778673 | 1.0189427 | X-03088 |
| Inverse variance weighted | 5 | -0.0456434 | 0.03178195 | 0.15096226 | 0.95538263 | 0.89768507 | 1.01678863 | X-03088 |
| Simple mode | 5 | -0.0589901 | 0.05266659 | 0.32539219 | 0.94271606 | 0.85025697 | 1.04522938 | X-03088 |
| Weighted mode | 5 | -0.0589901 | 0.05071665 | 0.30943361 | 0.94271606 | 0.85351277 | 1.04124226 | X-03088 |
| MR Egger | 5 | -0.4991369 | 0.3160821 | 0.21242377 | 0.60705437 | 0.32671801 | 1.12792988 | benzoate |
| Weighted median | 5 | -0.0507307 | 0.03268731 | 0.12066189 | 0.95053457 | 0.89154633 | 1.0134257 | benzoate |
| Inverse variance weighted | 5 | -0.0402525 | 0.03862357 | 0.29733051 | 0.96054685 | 0.89051556 | 1.0360855 | benzoate |
| Simple mode | 5 | -0.0690743 | 0.05442734 | 0.27322199 | 0.93325736 | 0.8388261 | 1.03831927 | benzoate |
| Weighted mode | 5 | -0.0608969 | 0.04737793 | 0.2680446 | 0.94092023 | 0.85747979 | 1.03248016 | benzoate |
| MR Egger | 5 | 0.6209912 | 0.53332726 | 0.32845148 | 1.86077153 | 0.65420781 | 5.29261595 | aspartate |
| Weighted median | 5 | 0.08706447 | 0.05780041 | 0.13199141 | 1.09096702 | 0.97411655 | 1.22183432 | aspartate |
| Inverse variance weighted | 5 | 0.06567136 | 0.05454746 | 0.22861637 | 1.06787571 | 0.95959721 | 1.18837209 | aspartate |
| Simple mode | 5 | 0.11205619 | 0.08097641 | 0.23862332 | 1.11857571 | 0.95441416 | 1.31097345 | aspartate |
| Weighted mode | 5 | 0.09798253 | 0.07807862 | 0.27782555 | 1.10294352 | 0.94643635 | 1.28533144 | aspartate |
| MR Egger | 5 | 0.33547239 | 0.33755401 | 0.39356003 | 1.39860091 | 0.72170886 | 2.71035126 | 1,5-anhydroglucitol (1,5-AG) |
| Weighted median | 5 | 0.05197192 | 0.04177948 | 0.21351505 | 1.05334616 | 0.97052718 | 1.14323242 | 1,5-anhydroglucitol (1,5-AG) |
| Inverse variance weighted | 5 | 0.04379101 | 0.03249313 | 0.17775477 | 1.04476398 | 0.98030108 | 1.11346586 | 1,5-anhydroglucitol (1,5-AG) |
| Simple mode | 5 | 0.08387898 | 0.06064425 | 0.23881558 | 1.08749727 | 0.96562109 | 1.22475609 | 1,5-anhydroglucitol (1,5-AG) |
| Weighted mode | 5 | 0.07372053 | 0.0565583 | 0.26238365 | 1.07650591 | 0.96354727 | 1.20270693 | 1,5-anhydroglucitol (1,5-AG) |
| MR Egger | 5 | 0.6485749 | 0.33090886 | 0.14485497 | 1.91281293 | 0.99999354 | 3.65887694 | 1-palmitoylglycerol (1-monopalmitin) |
| Weighted median | 5 | 0.02532746 | 0.04437735 | 0.56818307 | 1.02565092 | 0.94020989 | 1.11885636 | 1-palmitoylglycerol (1-monopalmitin) |
| Inverse variance weighted | 5 | 0.06325774 | 0.03547732 | 0.07457862 | 1.06530138 | 0.99374185 | 1.14201392 | 1-palmitoylglycerol (1-monopalmitin) |
| Simple mode | 5 | 0.02588095 | 0.05395567 | 0.65651051 | 1.02621876 | 0.92323435 | 1.14069082 | 1-palmitoylglycerol (1-monopalmitin) |
| Weighted mode | 5 | 0.0165372 | 0.04994652 | 0.75718925 | 1.01667469 | 0.92186363 | 1.12123681 | 1-palmitoylglycerol (1-monopalmitin) |
| MR Egger | 5 | 0.24073975 | 0.3003398 | 0.48142156 | 1.2721899 | 0.70615095 | 2.29195634 | levulinate (4-oxovalerate) |
| Weighted median | 5 | -0.0118012 | 0.02996496 | 0.69370522 | 0.98826821 | 0.93189748 | 1.04804882 | levulinate (4-oxovalerate) |
| Inverse variance weighted | 5 | -0.0122676 | 0.02743208 | 0.65473076 | 0.98780732 | 0.93609857 | 1.04237238 | levulinate (4-oxovalerate) |
| Simple mode | 5 | 0.00693379 | 0.03506074 | 0.85287241 | 1.00695789 | 0.94008473 | 1.07858808 | levulinate (4-oxovalerate) |
| Weighted mode | 5 | -0.0051937 | 0.03484832 | 0.88873592 | 0.99481975 | 0.92913947 | 1.06514293 | levulinate (4-oxovalerate) |
| MR Egger | 5 | -0.6388703 | 0.28739732 | 0.11273307 | 0.52788846 | 0.30054162 | 0.92721341 | glycine |
| Weighted median | 5 | -0.0192975 | 0.03796007 | 0.61119866 | 0.9808875 | 0.91055658 | 1.05665075 | glycine |
| Inverse variance weighted | 5 | 0.00860266 | 0.03951999 | 0.82767951 | 1.00863977 | 0.9334606 | 1.08987372 | glycine |
| Simple mode | 5 | -0.036068 | 0.05403701 | 0.54100761 | 0.96457468 | 0.86763812 | 1.07234145 | glycine |
| Weighted mode | 5 | -0.0338114 | 0.04423844 | 0.48728637 | 0.9667538 | 0.88646046 | 1.0543199 | glycine |
| MR Egger | 5 | 1.10992895 | 0.60460703 | 0.16372331 | 3.03414281 | 0.92764994 | 9.92402648 | 3-methylxanthine |
| Weighted median | 5 | -0.0910748 | 0.07700952 | 0.23695066 | 0.91294945 | 0.78504565 | 1.06169203 | 3-methylxanthine |
| Inverse variance weighted | 5 | -0.0441794 | 0.06484606 | 0.49568352 | 0.95678227 | 0.84258755 | 1.08645363 | 3-methylxanthine |
| Simple mode | 5 | -0.13045 | 0.11589221 | 0.32329504 | 0.87770041 | 0.69935367 | 1.10152852 | 3-methylxanthine |
| Weighted mode | 5 | -0.1134378 | 0.10929198 | 0.35792231 | 0.89275969 | 0.72061511 | 1.10602714 | 3-methylxanthine |
| MR Egger | 5 | 0.29521241 | 0.17356194 | 0.1875181 | 1.34341168 | 0.95602713 | 1.88776541 | C-glycosyltryptophan* |
| Weighted median | 5 | 0.01994233 | 0.02046947 | 0.32993367 | 1.0201425 | 0.98002438 | 1.0619029 | C-glycosyltryptophan* |
| Inverse variance weighted | 5 | 0.00221394 | 0.01768024 | 0.90034839 | 1.00221639 | 0.96808118 | 1.03755525 | C-glycosyltryptophan* |
| Simple mode | 5 | 0.02099756 | 0.027108 | 0.48182146 | 1.02121956 | 0.96837669 | 1.07694598 | C-glycosyltryptophan* |
| Weighted mode | 5 | 0.02131383 | 0.02519105 | 0.44515055 | 1.02154259 | 0.97232941 | 1.07324663 | C-glycosyltryptophan* |
| MR Egger | 5 | 0.63072079 | 1.07913247 | 0.59995231 | 1.87896442 | 0.22664308 | 15.5773887 | X-11445--5-alpha-pregnan-3beta,20alpha-disulfate |
| Weighted median | 5 | 0.16613334 | 0.13232569 | 0.20930159 | 1.18073053 | 0.91098849 | 1.5303427 | X-11445--5-alpha-pregnan-3beta,20alpha-disulfate |
| Inverse variance weighted | 5 | 0.10063878 | 0.10364484 | 0.33155002 | 1.1058771 | 0.90257354 | 1.35497453 | X-11445--5-alpha-pregnan-3beta,20alpha-disulfate |
| Simple mode | 5 | 0.15340235 | 0.17484278 | 0.42982117 | 1.16579394 | 0.8275469 | 1.64229426 | X-11445--5-alpha-pregnan-3beta,20alpha-disulfate |
| Weighted mode | 5 | 0.1598392 | 0.1498807 | 0.34631408 | 1.17332218 | 0.87465394 | 1.57397672 | X-11445--5-alpha-pregnan-3beta,20alpha-disulfate |
| MR Egger | 5 | 0.48584294 | 0.35953189 | 0.26946376 | 1.62554467 | 0.80345061 | 3.28880884 | adrenate (22:4n6) |
| Weighted median | 5 | 0.00386839 | 0.045319 | 0.93197596 | 1.00387588 | 0.91855191 | 1.09712555 | adrenate (22:4n6) |
| Inverse variance weighted | 5 | 0.00090973 | 0.0385908 | 0.9811926 | 1.00091014 | 0.92799565 | 1.07955368 | adrenate (22:4n6) |
| Simple mode | 5 | 0.00163621 | 0.05976755 | 0.97947099 | 1.00163755 | 0.89091328 | 1.12612283 | adrenate (22:4n6) |
| Weighted mode | 5 | 0.01317398 | 0.0551778 | 0.82302885 | 1.01326114 | 0.90939613 | 1.12898891 | adrenate (22:4n6) |
| MR Egger | 5 | -0.4643047 | 0.56018623 | 0.46798065 | 0.62857198 | 0.20965966 | 1.88449576 | X-11820 |
| Weighted median | 5 | 0.00763336 | 0.05865949 | 0.89646358 | 1.00766257 | 0.89822091 | 1.13043889 | X-11820 |
| Inverse variance weighted | 5 | -0.0336233 | 0.05295978 | 0.52550455 | 0.9669357 | 0.87160019 | 1.07269899 | X-11820 |
| Simple mode | 5 | 0.03928611 | 0.08875141 | 0.68089993 | 1.04006801 | 0.87400721 | 1.23768026 | X-11820 |
| Weighted mode | 5 | 0.04758269 | 0.07346755 | 0.55249607 | 1.04873291 | 0.90808826 | 1.2111606 | X-11820 |
| MR Egger | 5 | -1.3506516 | 1.1660258 | 0.33056242 | 0.25907139 | 0.02635593 | 2.54659954 | X-11852 |
| Weighted median | 5 | 0.10292783 | 0.1413394 | 0.46647178 | 1.10841141 | 0.84021506 | 1.46221593 | X-11852 |
| Inverse variance weighted | 5 | 0.06101057 | 0.11801931 | 0.60518816 | 1.06291015 | 0.84340562 | 1.33954288 | X-11852 |
| Simple mode | 5 | 0.08582335 | 0.20889075 | 0.70223727 | 1.08961383 | 0.72353777 | 1.64090714 | X-11852 |
| Weighted mode | 5 | 0.11923981 | 0.18863434 | 0.56163553 | 1.12664006 | 0.77842432 | 1.63062457 | X-11852 |
| MR Egger | 5 | 0.11505894 | 2.25374941 | 0.96249296 | 1.12193956 | 0.01353752 | 92.9821733 | X-12040 |
| Weighted median | 5 | -0.1471823 | 0.20516339 | 0.47313294 | 0.86313657 | 0.57735233 | 1.29038148 | X-12040 |
| Inverse variance weighted | 5 | -0.0526043 | 0.20035729 | 0.79289553 | 0.94875536 | 0.64062912 | 1.40508244 | X-12040 |
| Simple mode | 5 | -0.1550528 | 0.27922501 | 0.60828134 | 0.85636995 | 0.49542771 | 1.48027547 | X-12040 |
| Weighted mode | 5 | -0.1711649 | 0.25239378 | 0.534871 | 0.84268262 | 0.51383314 | 1.38199336 | X-12040 |
| MR Egger | 5 | -0.0882998 | 2.25048741 | 0.97116737 | 0.91548635 | 0.01111727 | 75.3885571 | X-12189 |
| Weighted median | 5 | -0.1133583 | 0.1543023 | 0.4625522 | 0.89283069 | 0.65981828 | 1.20813059 | X-12189 |
| Inverse variance weighted | 5 | -0.1549373 | 0.19972658 | 0.43789831 | 0.85646886 | 0.57902976 | 1.26684147 | X-12189 |
| Simple mode | 5 | -0.0958242 | 0.17866862 | 0.62017194 | 0.9086237 | 0.64017433 | 1.28964406 | X-12189 |
| Weighted mode | 5 | -0.1019418 | 0.1643682 | 0.56871028 | 0.90308207 | 0.65435614 | 1.24635069 | X-12189 |
| MR Egger | 5 | -0.7382044 | 1.10346773 | 0.55138214 | 0.47797138 | 0.05496816 | 4.15616314 | X-12261 |
| Weighted median | 5 | -0.0943967 | 0.14542014 | 0.51625365 | 0.90992169 | 0.68425802 | 1.21000772 | X-12261 |
| Inverse variance weighted | 5 | -0.0687457 | 0.11458425 | 0.54853428 | 0.93356403 | 0.74577536 | 1.1686385 | X-12261 |
| Simple mode | 5 | 0.02713586 | 0.19351119 | 0.89525696 | 1.0275074 | 0.7031774 | 1.50142972 | X-12261 |
| Weighted mode | 5 | -0.1658632 | 0.17136157 | 0.38789831 | 0.84716211 | 0.60548113 | 1.18531132 | X-12261 |
| MR Egger | 5 | 0.24950691 | 0.37903353 | 0.55737107 | 1.28339244 | 0.61054773 | 2.69773526 | alpha-hydroxyisovalerate |
| Weighted median | 5 | 0.01250205 | 0.047939 | 0.79425385 | 1.01258053 | 0.92177106 | 1.11233621 | alpha-hydroxyisovalerate |
| Inverse variance weighted | 5 | -0.0038674 | 0.04021529 | 0.9233885 | 0.99614011 | 0.92063713 | 1.07783522 | alpha-hydroxyisovalerate |
| Simple mode | 5 | 0.01460613 | 0.06235864 | 0.82630846 | 1.01471332 | 0.89797164 | 1.14663213 | alpha-hydroxyisovalerate |
| Weighted mode | 5 | 0.01602177 | 0.05829982 | 0.79706734 | 1.01615081 | 0.90642601 | 1.13915803 | alpha-hydroxyisovalerate |
| MR Egger | 5 | -0.1848115 | 0.26889881 | 0.54126594 | 0.831261 | 0.49073396 | 1.40808441 | N-acetylthreonine |
| Weighted median | 5 | -0.0196203 | 0.03543331 | 0.57976796 | 0.98057096 | 0.91478196 | 1.05109136 | N-acetylthreonine |
| Inverse variance weighted | 5 | -0.0326356 | 0.02748315 | 0.23503973 | 0.9678912 | 0.9171332 | 1.02145837 | N-acetylthreonine |
| Simple mode | 5 | -0.0129995 | 0.04539257 | 0.7888086 | 0.98708467 | 0.90305763 | 1.07893019 | N-acetylthreonine |
| Weighted mode | 5 | -0.0164282 | 0.04286705 | 0.72104061 | 0.98370596 | 0.90443245 | 1.0699278 | N-acetylthreonine |
| MR Egger | 5 | -0.4286023 | 0.37742755 | 0.33864412 | 0.65141896 | 0.31087628 | 1.36500174 | 1-stearoylglycerophosphocholine |
| Weighted median | 5 | -0.0167922 | 0.05167574 | 0.74521578 | 0.98334796 | 0.88862789 | 1.08816437 | 1-stearoylglycerophosphocholine |
| Inverse variance weighted | 5 | -0.0288126 | 0.03902191 | 0.4602901 | 0.97159855 | 0.9000585 | 1.04882487 | 1-stearoylglycerophosphocholine |
| Simple mode | 5 | 0.01787019 | 0.07482616 | 0.8229799 | 1.01803082 | 0.87915938 | 1.1788383 | 1-stearoylglycerophosphocholine |
| Weighted mode | 5 | 0.01103125 | 0.0723071 | 0.8861306 | 1.01109232 | 0.87748918 | 1.16503737 | 1-stearoylglycerophosphocholine |
| MR Egger | 5 | -0.1544371 | 0.36988976 | 0.70437803 | 0.8568974 | 0.41502313 | 1.76923428 | X-12726 |
| Weighted median | 5 | -0.0617819 | 0.04398202 | 0.16010816 | 0.9400879 | 0.86244262 | 1.02472355 | X-12726 |
| Inverse variance weighted | 5 | -0.0517978 | 0.03419749 | 0.12985665 | 0.94952082 | 0.88796328 | 1.01534581 | X-12726 |
| Simple mode | 5 | -0.0693098 | 0.05335253 | 0.26373407 | 0.93303757 | 0.8403971 | 1.03589018 | X-12726 |
| Weighted mode | 5 | -0.0732971 | 0.0419769 | 0.15571874 | 0.92932469 | 0.8559256 | 1.00901806 | X-12726 |
| MR Egger | 5 | 1.3530209 | 0.81860035 | 0.1969322 | 3.86909604 | 0.77768339 | 19.2493557 | X-12850 |
| Weighted median | 5 | 0.13693158 | 0.08734561 | 0.1169515 | 1.14674969 | 0.96631461 | 1.3608765 | X-12850 |
| Inverse variance weighted | 5 | 0.07485632 | 0.09815148 | 0.4456655 | 1.07772929 | 0.88912222 | 1.30634507 | X-12850 |
| Simple mode | 5 | 0.17305012 | 0.11938774 | 0.22079862 | 1.18892569 | 0.94087043 | 1.50237936 | X-12850 |
| Weighted mode | 5 | 0.17817705 | 0.10452879 | 0.16347647 | 1.19503688 | 0.9736539 | 1.46675645 | X-12850 |
| MR Egger | 5 | -0.2510884 | 0.40019817 | 0.57489627 | 0.77795356 | 0.35505703 | 1.704548 | 2-stearoylglycerophosphocholine* |
| Weighted median | 5 | 0.0055393 | 0.04994732 | 0.91169335 | 1.00555467 | 0.91177918 | 1.10897487 | 2-stearoylglycerophosphocholine* |
| Inverse variance weighted | 5 | 0.01253503 | 0.04129185 | 0.76145423 | 1.01261393 | 0.93388966 | 1.09797443 | 2-stearoylglycerophosphocholine* |
| Simple mode | 5 | -0.0429219 | 0.07713854 | 0.60757805 | 0.95798618 | 0.82356445 | 1.11434815 | 2-stearoylglycerophosphocholine* |
| Weighted mode | 5 | -0.0087549 | 0.07052316 | 0.90719113 | 0.99128332 | 0.86331099 | 1.13822554 | 2-stearoylglycerophosphocholine* |
| MR Egger | 5 | -0.8312457 | 1.15281263 | 0.5229851 | 0.43550642 | 0.04546745 | 4.17146383 | hydroquinone sulfate |
| Weighted median | 5 | -0.0307505 | 0.1503818 | 0.83797601 | 0.96971745 | 0.722167 | 1.30212533 | hydroquinone sulfate |
| Inverse variance weighted | 5 | 0.01299601 | 0.1232785 | 0.91604261 | 1.01308082 | 0.79562298 | 1.28997374 | hydroquinone sulfate |
| Simple mode | 5 | -0.0273272 | 0.17153903 | 0.88114802 | 0.97304281 | 0.69520834 | 1.36191161 | hydroquinone sulfate |
| Weighted mode | 5 | -0.0298236 | 0.18318561 | 0.87856561 | 0.97061674 | 0.67782416 | 1.38988387 | hydroquinone sulfate |
| MR Egger | 5 | 0.18616996 | 0.45615675 | 0.71056165 | 1.20462698 | 0.49267908 | 2.94537804 | 1-myristoylglycerophosphocholine |
| Weighted median | 5 | 0.02206363 | 0.04878892 | 0.65110604 | 1.02230883 | 0.92907794 | 1.12489523 | 1-myristoylglycerophosphocholine |
| Inverse variance weighted | 5 | 0.01007614 | 0.04246149 | 0.81242362 | 1.01012707 | 0.9294629 | 1.09779174 | 1-myristoylglycerophosphocholine |
| Simple mode | 5 | 0.01356518 | 0.05666959 | 0.82258151 | 1.0136576 | 0.90709579 | 1.13273786 | 1-myristoylglycerophosphocholine |
| Weighted mode | 5 | 0.02290476 | 0.0535431 | 0.69083529 | 1.02316909 | 0.92123537 | 1.13638167 | 1-myristoylglycerophosphocholine |
| MR Egger | 5 | 0.94831621 | 0.58697287 | 0.20459493 | 2.58135954 | 0.81697188 | 8.15623799 | X-14632 |
| Weighted median | 5 | -0.0544851 | 0.08240836 | 0.50851008 | 0.94697265 | 0.80573091 | 1.11297357 | X-14632 |
| Inverse variance weighted | 5 | -0.0270763 | 0.068096 | 0.69091093 | 0.973287 | 0.85167998 | 1.11225767 | X-14632 |
| Simple mode | 5 | -0.0981962 | 0.12411776 | 0.4731228 | 0.90647105 | 0.71072693 | 1.15612585 | X-14632 |
| Weighted mode | 5 | -0.1148048 | 0.11591728 | 0.37804111 | 0.89154016 | 0.71034631 | 1.11895261 | X-14632 |

MR, Mendelian randomization; se, standard error; or, odds ratio; ci, confidence interval.
