# Supplementary material for: Causality of genetically determined serum metabolites on lower back pain or/and sciatica: a comprehensive Mendelian randomized study
Source: Front Pain Res (Lausanne). 2024 Sep 25;5:1370704. doi: 10.3389/fpain.2024.1370704 (PMC11461461; doi:10.3389/fpain.2024.1370704)
Supplement: Supplementary file 7 [file Table4.docx]

| Table 4. The colocalization analysis illustrating the associations between 28 metabolites and sciatica or/and lower back pain. | | | | | | | | |
| --- | --- | --- | --- | --- | --- | --- | --- | --- |
| metabolites | leadsnp | leadsnp-pos | n-snps | PP.H0.abf | PP.H1.abf | PP.H2.abf | PP.H3.abf | PP.H4.abf |
| tyrosine | rs9400467 | 111530708 | 22636 | 2.33E-08 | 7.38E-08 | 0.23837271 | 0.75620936 | 0.00541784 |
| malate | rs7503429 | 77784714 | 35386 | 6.16E-05 | 0.00034437 | 0.15128202 | 0.84623243 | 0.00207962 |
| pentadecanoate (15:0) | rs6887589 | 107039458 | 26230 | 0.02758996 | 0.0172441 | 0.58033314 | 0.36271448 | 0.01211832 |
| X-03088 | rs7915053 | 122897295 | 21047 | 0.27629586 | 0.11711713 | 0.40023426 | 0.16964433 | 0.03670841 |
| benzoate | rs247616 | 55547091 | 36802 | 0.08423923 | 0.12862998 | 0.29845603 | 0.45572821 | 0.03294654 |
| aspartate | rs7150776 | 92183574 | 24425 | 0.14165978 | 0.21887516 | 0.2458586 | 0.37986799 | 0.01373847 |
| 1,5-anhydroglucitol (1,5-AG) | rs7570971 | 135554376 | 15681 | 4.45E-38 | 2.06E-38 | 0.66915196 | 0.30928693 | 0.02156111 |
| 1-palmitoylglycerol (1-monopalmitin) | rs4774615 | 50320652 | 30195 | 0.01079206 | 0.13472718 | 0.0315126 | 0.39335417 | 0.42961399 |
| levulinate (4-oxovalerate) | rs13278849 | 26770791 | 38663 | 7.52E-07 | 0.00010635 | 0.00702298 | 0.99271482 | 0.0001551 |
| glycine | rs715 | 211251300 | 3598 | 3.78E-139 | 2.94E-140 | 0.92170244 | 0.07170822 | 0.00658935 |
| 3-methylxanthine | rs10754948 | 15223189 | 36656 | 0.01716998 | 0.03205128 | 0.32556308 | 0.60772877 | 0.0174869 |
| C-glycosyltryptophan* | rs6867478 | 92148101 | 24565 | 0.04908301 | 0.07591868 | 0.29987615 | 0.46381238 | 0.11130978 |
| X-11445--5-alpha-pregnan-3beta,20alpha-disulfate | rs10491431 | 36003757 | 37681 | 4.56E-06 | 1.39E-05 | 0.2455182 | 0.74882144 | 0.00564188 |
| adrenate (22:4n6) | rs174550 | 61328054 | 31601 | 8.93E-18 | 1.02E-17 | 0.45886563 | 0.52313537 | 0.017999 |
| X-11820 | rs439401 | 50106291 | 29657 | 9.98E-13 | 4.67E-10 | 0.00196463 | 0.91885843 | 0.07917694 |
| X-11852 | rs7808781 | 52200991 | 33618 | 0.24172243 | 0.15339929 | 0.34379363 | 0.21817088 | 0.04291377 |
| X-12040 | rs11736351 | 81820692 | 32291 | 0.03218087 | 0.00699219 | 0.70630957 | 0.15345576 | 0.10106161 |
| X-12189 | rs8082167 | 49849155 | 29342 | 5.98E-06 | 0.01547868 | 0.0003801 | 0.98361662 | 0.00051862 |
| X-12261 | rs8085059 | 13482909 | 38035 | 0.05475098 | 0.01621125 | 0.20419575 | 0.0604152 | 0.66442682 |
| alpha-hydroxyisovalerate | rs2403254 | 18281722 | 37686 | 2.51E-24 | 6.92E-24 | 0.26079274 | 0.72025696 | 0.0189503 |
| N-acetylthreonine | rs12857329 | 19736486 | 41762 | 0.38555856 | 0.07835232 | 0.42810682 | 0.08699769 | 0.02098461 |
| 1-stearoylglycerophosphocholine | rs476092 | 127624474 | 21498 | 0.02083686 | 0.14402321 | 0.10301523 | 0.71203131 | 0.0200934 |
| X-12726 | rs13419959 | 208457252 | 3608 | 0.047941 | 0.01245081 | 0.73115353 | 0.18987013 | 0.01858453 |
| X-12850 | rs2547231 | 53076869 | 35661 | 9.16E-10 | 6.15E-10 | 0.59209697 | 0.39771701 | 0.01018601 |
| 2-stearoylglycerophosphocholine* | rs2121073 | 75735493 | 31124 | 0.00644736 | 0.00215302 | 0.73144399 | 0.24425555 | 0.01570008 |
| hydroquinone sulfate | rs10514235 | 73039466 | 32355 | 0.00231155 | 0.15100163 | 0.01265641 | 0.8267767 | 0.0072537 |
| 1-myristoylglycerophosphocholine | rs780093 | 27596107 | 36086 | 0.00208826 | 0.10347737 | 0.01755898 | 0.87007992 | 0.00679546 |
| X-14632 | rs17152472 | 13000217 | 36939 | 0.40237235 | 0.15492452 | 0.30319159 | 0.11673553 | 0.02277602 |

snp, single nucleotide polymorphism; pos, position; PP, posterior probability.
