# Supplementary material for: Causality of genetically determined serum metabolites on lower back pain or/and sciatica: a comprehensive Mendelian randomized study
Source: Front Pain Res (Lausanne). 2024 Sep 25;5:1370704. doi: 10.3389/fpain.2024.1370704 (PMC11461461; doi:10.3389/fpain.2024.1370704)
Supplement: Supplementary file 8 [file Table5.docx]

| Table 3. Signifcant Metabolic Pathways Involved in sciatica or/and lower back pain. | | | | | | |
| --- | --- | --- | --- | --- | --- | --- |
| Metabolite set | Total | Expected | Hits | p-value | Holm P | FDR |
| Aminoacyl-tRNA biosynthesis | 48 | 0.12387 | 3 | 0.00010925 | 0.009177 | 0.009177 |
| Glyoxylate and dicarboxylate metabolism | 32 | 0.082581 | 2 | 0.0024154 | 0.20048 | 0.10145 |
| Phenylalanine, tyrosine and tryptophan biosynthesis | 4 | 0.010323 | 1 | 0.010293 | 0.84399 | 0.28819 |
| Ubiquinone and other terpenoid-quinone biosynthesis | 9 | 0.023226 | 1 | 0.023046 | 1 | 0.38941 |
| Phenylalanine metabolism | 10 | 0.025806 | 1 | 0.025582 | 1 | 0.38941 |
| Arginine biosynthesis | 14 | 0.036129 | 1 | 0.035677 | 1 | 0.38941 |
| Nicotinate and nicotinamide metabolism | 15 | 0.03871 | 1 | 0.038188 | 1 | 0.38941 |
| Histidine metabolism | 16 | 0.04129 | 1 | 0.040694 | 1 | 0.38941 |
| Pantothenate and CoA biosynthesis | 19 | 0.049032 | 1 | 0.048184 | 1 | 0.38941 |

FDR, false discovery rate.
