# Supplementary material for: Causality of genetically determined serum metabolites on lower back pain or/and sciatica: a comprehensive Mendelian randomized study
Source: Front Pain Res (Lausanne). 2024 Sep 25;5:1370704. doi: 10.3389/fpain.2024.1370704 (PMC11461461; doi:10.3389/fpain.2024.1370704)
Supplement: Supplementary file 9 [file Datasheet1.docx]

**STROBE-MR checklist of recommended items to address in reports of Mendelian randomization studies**^1^ ^2^

| **Item No.** | **Section** | **Checklist item** | **Page No.** | **Relevant text from manuscript** |
| --- | --- | --- | --- | --- |
| 1 | **TITLE and ABSTRACT** | Indicate Mendelian randomization (MR) as the study’s design in the title and/or the abstract if that is a main purpose of the study | 1 | Causality of genetically determined serum metabolites on lower back pain or/and sciatica: A comprehensive Mendelian randomized study  This study aimed to conduct a two sample bidirectional Mendelian randomization (MR) analysis to explore the causal link between 486 serum metabolites and lower back pain or/and sciatica. |
|  | **INTRODUCTION** |  |  |  |
| 2 | **Background** | Explain the scientific background and rationale for the reported study. What is the exposure? Is a potential causal relationship between exposure and outcome plausible? Justify why MR is a helpful method to address the study question | 3 | However, with the development of metabolomics, the idea of identifying specific small molecule biomarkers in the serum of patients with sciatica or/and lower back pain and identifying potential causes has become possible.  Mendelian randomization (MR) is a form of instrumental variable (IV) analysis that employs genetic variations as an IV to identify and measure causality. Due to its ability to mitigate the impact of potential confounding variables and reverse causality, MR has gained popularity in observational research in recent times. Moreover, non-experimental investigations frequently incorporate possible variables that may distort the results and the possibility of cause and effect being mistakenly identified. |
| 3 | **Objectives** | State specific objectives clearly, including pre-specified causal hypotheses (if any). State that MR is a method that, under specific assumptions, intends to estimate causal effects | 5 | Thus, the objective of this study was to elucidate the genetic connection and potential causative link between 486 human serum metabolites and risks of sciatica or/and lower back pain through a bidirectional MR validation. |
|  | **METHODS** |  |  |  |
| 4 | **Study design and data sources** | Present key elements of the study design early in the article. Consider including a table listing sources of data for all phases of the study. For each data source contributing to the analysis, describe the following: |  |  |
|  | a) | Setting: Describe the study design and the underlying population, if possible. Describe the setting, locations, and relevant dates, including periods of recruitment, exposure, follow-up, and data collection, when available. | 6 | This study used two sample bidirectional Mendelian randomization method to explore the causal relationship between 486 human serum metabolites and risks of sciatica or/and lower back pain. First, the association data of human serum metabolites (exposure factor) were obtained from a GWAS, so as to clarify the single nucleotide polymorphism (SNP) site associated with human serum metabolites, and treat it as gene instrumental variables estimation. Subsequently, another GWAS study was conducted to obtain association data with sciatica or/and lower back pain (outcome) and clarify the existence of relevant SNPs; Finally, through the screened SNPs and various statistical methods, we can comprehensively judge the causal relationship between 486 human serum metabolites and risks of sciatica or/and lower back pain. |
|  | b) | Participants: Give the eligibility criteria, and the sources and methods of selection of participants. Report the sample size, and whether any power or sample size calculations were carried out prior to the main analysis | 6,7 | We obtained summary association data from the most extensive genetic investigation into human metabolism, which was openly accessible on the Metabolomics GWAS Server (website: http://metabolomics.helmholtz-muenchen.de/gwas/). Utilizing a dataset comprising 7,824 individuals from two European population cohorts, a comprehensive set of 486 metabolites was measured using the MS (Metabolon) platform. Subsequently, GWAS analyses were conducted utilizing the genotype dataset imputed based on HapMap2. Finally, the GWAS meta-analysis encompassed approximately 2.1 million SNPs. Data related to sciatica or/and lower back pain is derived from the GWAS dataset (https://gwas.mrcieu.ac.uk/datasets/finn-b-M13_LOWBACKPAINORANDSCIATICA/). The sample size of this dataset is 218,792(ncase=19,509, ncontrol=199,283), including Europeans with a SNP count of 16,380,466. |
|  | c) | Describe measurement, quality control and selection of genetic variants | 8 | To identify instrumental variables (IVs) for the 486 metabolites, certain procedures were undertaken to ensure the validity of the first assumption. Initially, genetic variants were extracted using association thresholds of P<1×10–5, which are commonly employed in MR analysis to capture greater variability when limited SNPs are available for exposure. Subsequently, independent variants were identified through a clumping procedure implemented in R software, with a linkage-disequilibrium threshold of r2<0.01 within a 500 kb window based on the European 1000 Genomes Project Phase 3 reference panel. |
|  | d) | For each exposure, outcome, and other relevant variables, describe methods of assessment and diagnostic criteria for diseases | 3 | Sciatica or/and lower back pain is not a medical diagnosis, but rather a symptom secondary to degenerative diseases of the lumbar spine such as disc herniation and spinal stenosis. |
|  | e) | Provide details of ethics committee approval and participant informed consent, if relevant | 6 | All published GWAS received ethical approval from the appropriate institutional review boards. This study utilized only summary-level data, eliminating the need for additional ethical approval. |
| 5 | **Assumptions** | Explicitly state the three core IV assumptions for the main analysis (relevance, independence and exclusion restriction) as well assumptions for any additional or sensitivity analysis | 8 | Based on the requirements of MR analysis, genetic variants as instrumental variables (IVs) need to fulfill the following three fundamental assumptions: (I) a robust correlation exists between instrumental variables and the exposure factor X; (II) instrumental variables are not linked to any confounding factors affecting the association between exposure and outcome; and (III) instrumental variables do not influence outcome Y, except for a possible indirect impact through their association with exposure X |
| 6 | **Statistical methods: main analysis** | Describe statistical methods and statistics used | 9 | In this study, five statistical methods, simple mode, weighted mode, inverse varianceweighted analysis (IVW), weighted median method and MR-Egger regression, were used to analyze the causal association between human serum metabolites and risks of sciatica or/and lower back pain. |
|  | a) | Describe how quantitative variables were handled in the analyses (i.e., scale, units, model) | 9 | As the most well-established research approach in Mendelian randomization, the IVW analysis method (utilizing a random effects model when heterogeneity exists and a fixed effects model when there is no heterogeneity) conducts weighted analysis on genetic variants that satisfy the criteria of valid instrumental variables. |
|  | b) | Describe how genetic variants were handled in the analyses and, if applicable, how their weights were selected | 9 | the IVW analysis method (utilizing a random effects model when heterogeneity exists and a fixed effects model when there is no heterogeneity) conducts weighted analysis on genetic variants that satisfy the criteria of valid instrumental variables. The weighted midpoint approach necessitates that a minimum of 50% of SNPs fulfill the requirement of being effective instrumental variables. |
|  | c) | Describe the MR estimator (e.g. two-stage least squares, Wald ratio) and related statistics. Detail the included covariates and, in case of two-sample MR, whether the same covariate set was used for adjustment in the two samples | 8 | MR Steiger filters were used to exclude SNPs with an incorrect causal direction. |
|  | d) | Explain how missing data were addressed | none | none |
|  | e) | If applicable, indicate how multiple testing was addressed | none | none |
| 7 | **Assessment of assumptions** | Describe any methods or prior knowledge used to assess the assumptions or justify their validity | 8 | Typically, a threshold of F>10 is recommended for further MR analysis. |
| 8 | **Sensitivity analyses and additional analyses** | Describe any sensitivity analyses or additional analyses performed (e.g. comparison of effect estimates from different approaches, independent replication, bias analytic techniques, validation of instruments, simulations) | 10 | The leave-one-out method was employed to assess the sensitivity of the Mendelian randomization findings. After sequentially removing the incorporated SNPs, the IVW technique was employed to compute the effect of analysis on the remaining all SNPs, and the impact of an individual SNP on the estimation of causal judgment was determined accordingly. The asymmetry plot from the funnel plot was utilized to identify any heterogeneity, and a symmetric plot confirmed the lack of heterogeneity. An additional indication that horizontal multiplicity is absent can be observed through the symmetry of the funnel plot. |
| 9 | **Software and pre-registration** |  |  |  |
|  | a) | Name statistical software and package(s), including version and settings used | 10 | The statistical analysis, employing the Two Sample MR 0.5.6 and MR-PRESSO packages, was conducted using R software version 3.6.0. |
|  | b) | State whether the study protocol and details were pre-registered (as well as when and where) | none | none |
|  | **RESULTS** |  |  |  |
| 10 | **Descriptive data** |  |  |  |
|  | a) | Report the numbers of individuals at each stage of included studies and reasons for exclusion. Consider use of a flow diagram |  | Figure 1 |
|  | b) | Report summary statistics for phenotypic exposure(s), outcome(s), and other relevant variables (e.g. means, SDs, proportions) |  | Table 1 and Table 2 |
|  | c) | If the data sources include meta-analyses of previous studies, provide the assessments of heterogeneity across these studies |  | Table 1 and Table 2 |
|  | d) | For two-sample MR:  i.  Provide justification of the similarity of the genetic variant-exposure associations between the exposure and outcome samples  ii.  Provide information on the number of individuals who overlap between the exposure and outcome studies |  | Table 1 and Table 2 |
| 11 | **Main results** |  |  |  |
|  | a) | Report the associations between genetic variant and exposure, and between genetic variant and outcome, preferably on an interpretable scale | 13-15 | Table 1 and Table 2 |
|  | b) | Report MR estimates of the relationship between exposure and outcome, and the measures of uncertainty from the MR analysis, on an interpretable scale, such as odds ratio or relative risk per SD difference | 13-15 | Table 1 and Table 2 |
|  | c) | If relevant, consider translating estimates of relative risk into absolute risk for a meaningful time period | none |  |
|  | d) | Consider plots to visualize results (e.g. forest plot, scatterplot of associations between genetic variants and outcome versus between genetic variants and exposure) | 15 | Figure 2-Figure 4 |
| 12 | **Assessment of assumptions** |  |  |  |
|  | a) | Report the assessment of the validity of the assumptions | 12,13 | The IVW approach was utilized to validate the causal relationship between the 480 metabolites and sciatica or/and lower back pain. In total, 28 distinct metabolites were confirmed with a PIVW value of less than 0.05 and FDR < 0.05 |
|  | b) | Report any additional statistics (e.g., assessments of heterogeneity across genetic variants, such as *I^2^*, Q statistic or E-value) | 12 | Furthermore, all IVs were adequately effective for conducting MR analysis on the 480 metabolites (F-statistic > 10). |
| 13 | **Sensitivity analyses and additional analyses** |  |  |  |
|  | a) | Report any sensitivity analyses to assess the robustness of the main results to violations of the assumptions | 14,15 | The comparison between serum metabolites and sciatica or/and lower back pain was examined using Cochran's Q test for heterogeneity. The results indicated that there was no significant heterogeneity, with IVW and MR Egger. Consequently, the fixed-effects model was chosen for MR analysis. Only two comparisons (adrenate (22:4n6) and X-12189 on sciatica or/and lower back pain) showed horizontal pleiotropy causing that P values were＜0.05 in MR-Egger and IVW Cochran's Q tests. The analysis outcomes for heterogeneity and pluripotency are displayed in Table 2. To evaluate the MR analysis results, five methods were employed, and scatter plots were produced (Fig. 2). Among these methods, MR-Egger was utilized to assess the pluripotency of IVs. The intercept of MR-Egger did not significantly differ from the zero intercept of IVW, indicating the absence of horizontal pluripotency. Furthermore, as demonstrated by the funnel plot (Fig. 3), there was no apparent symmetry in the variation of effect size around the estimated point. This lack of symmetry can be attributed to the limited sample size of the SNP and should not be interpreted as an absence of horizontal pleiotropy. |
|  | b) | Report results from other sensitivity analyses or additional analyses | 16 | For the 28 identified metabolites linked to sciatica or/and lower back pain risk in this study, we performed a colocalization analysis to evaluate their relationship with sciatica or/and lower back pain outcomes. This analysis used exposure data from a 1,000-kb region around the lead SNP to ensure that the observed associations were not influenced by the same causal variant within that area. This strategy aimed to reduce the chance of a false association between the two phenotypes (see Table 4). The results of the colocalization analysis showed that the associations between sciatica or/and lower back pain and the 28 identified metabolites were not due to shared causal variant sites. |
|  | c) | Report any assessment of direction of causal relationship (e.g., bidirectional MR) | 15,16 | Reverse MR analysis was subsequently conducted to assess the impact of sciatica and/or lower back pain on the 28 candidate serum metabolites identified in the forward MR analysis. SNPs that met a significance threshold of P<1Figure×10−5 were selected as genetic instruments closely linked to sciatica and/or lower back pain and utilized for LD clumping. However, no association was found between genetic predisposition to sciatica and/or lower back pain and changes in the concentrations of the candidate metabolites in the IVW analysis, as well as in the simple mode, weighted mode, weighted median, and MR-Egger analyses (Table 3). |
|  | d) | When relevant, report and compare with estimates from non-MR analyses | none | none |
|  | e) | Consider additional plots to visualize results (e.g., leave-one-out analyses) | 15 | We performed an analysis called leave-one-out to compute the MR result of the remaining IVs when each one was removed individually. Removing each SNP did not have a significant impact on the overall error line, as shown in Figure 4. Hence, the correlation analysis results from the two-sample MR study were relatively dependable. |
|  | **DISCUSSION** |  |  |  |
| 14 | **Key results** | Summarize key results with reference to study objectives | 17 | In this bidirectional comparison between two sets of samples, sciatica or/and lower back pain was found to have a suggestive connection with levels of some serum metabolites. We identifed 28 metabolites (18 known metabolites, 1 identified metabolites and 9 unknown metabolites) relevant to the risk of sciatica or/and lower back pain after using genetic variants as probes at PIVW<0.05 and FDR < 0.05. Among them, 8 serum metabolites decreased risk of sciatica or/and lower back pain significantly (P<0.05), and 14 serum metabolites increased risk of sciatica or/and lower back pain significantly (P<0.05). Our study did not identify any blood metabolites with bidirectional effects. Furthermore, GWAS-GWAS co-localization analysis provided robust evidence of the causal relationships unaffected by overlapping SNPs. Pathway enrichment analysis identifed 11 signifcant metabolic pathways, which are mainly involved in the pathological mechanism of sciatica or/and lower back pain (P<0.05). |
| 15 | **Limitations** | Discuss limitations of the study, taking into account the validity of the IV assumptions, other sources of potential bias, and imprecision. Discuss both direction and magnitude of any potential bias and any efforts to address them | 24,25 | However, our research does have certain limitations. Primarily, the majority of these GWAS data originate from populations in Europe. It is crucial to ascertain whether the conclusions we outlined remain applicable to other individuals. Secondly, we employed FDR multiple analysis correction for all 486 serum metabolites, and the all p-values after FDR corrections are greater than 0.05, which is because a large number of samples undergoing FDR correction simultaneously can result in false negatives. The primary objective of our study was to identify potential biomarkers for lower back pain and/or sciatica, as well as potential targets for treatment. However, implementing strict corrections such as Bonferroni or FDR correction could have resulted in the exclusion of significant findings. Therefore, this study only adjusted the P-values of 28 identified serum metabolites for FDR, and the adjusted P-values were all less than 0.05, which is consistent with previous results. Thus,we believe that such positive results are meaningful. Lastly, while this study encompassed a relatively extensive range of metabolites, the functions and mechanisms of some of these metabolites in the context of the disease were not entirely elucidated. Consequently, our interpretation of the results obtained from this MR analysis was limited. |
| 16 | **Interpretation** |  |  |  |
|  | a) | Meaning: Give a cautious overall interpretation of results in the context of their limitations and in comparison with other studies | 23 | Our research has many positive aspects. This analysis is the initial thorough MR investigation of lower back pain or/and sciatica with blood metabolites, examining proof of the bidirectional cause-and-effect relationship between blood metabolites on lower back pain or/and sciatica. |
|  | b) | Mechanism: Discuss underlying biological mechanisms that could drive a potential causal relationship between the investigated exposure and the outcome, and whether the gene-environment equivalence assumption is reasonable. Use causal language carefully, clarifying that IV estimates may provide causal effects only under certain assumptions | 18,19 | The pathological mechanism of sciatica or/and lower back pain is complex.......pain hypersensitivity responses. |
|  | c) | Clinical relevance: Discuss whether the results have clinical or public policy relevance, and to what extent they inform effect sizes of possible interventions | 20-23 | In this MR analysis, specific amino acid metabolites such as tyrosine, aspartate, and glycine were identified as neurotransmitters associated with sciatica or/and lower back pain - findings consistent with previous research. .......the mechanisms and drug targets associated with lower back pain and/or sciatica. |
| 17 | **Generalizability** | Discuss the generalizability of the study results (a) to other populations, (b) across other exposure periods/timings, and (c) across other levels of exposure | 20-23 | In this MR analysis, specific amino acid metabolites such as tyrosine, aspartate, and glycine were identified as neurotransmitters associated with sciatica or/and lower back pain - findings consistent with previous research. .......the mechanisms and drug targets associated with lower back pain and/or sciatica. |
|  | **OTHER INFORMATION** |  |  |  |
| 18 | **Funding** | Describe sources of funding and the role of funders in the present study and, if applicable, sources of funding for the databases and original study or studies on which the present study is based | 26 | This research is supported by Tianjin Science and Technology Major Special Project (21ZXJBSY00080), Tianjin Natural Science Foundation's Diversified Investment Youth Project (21JCQNJC01300), and National Natural Science Foundation Youth Program (82102563 and 82102671). |
| 19 | **Data and data sharing** | Provide the data used to perform all analyses or report where and how the data can be accessed, and reference these sources in the article. Provide the statistical code needed to reproduce the results in the article, or report whether the code is publicly accessible and if so, where | 6,7 | We obtained summary association data from the most extensive genetic investigation into human metabolism, which was openly accessible on the Metabolomics GWAS Server (website: http://metabolomics.helmholtz-muenchen.de/gwas/)Data related to sciatica or/and lower back pain is derived from the GWAS dataset (https://gwas.mrcieu.ac.uk/datasets/finn-b-M13_LOWBACKPAINORANDSCIATICA/). |
| 20 | **Conflicts of Interest** | All authors should declare all potential conflicts of interest | none | none |

This checklist is copyrighted by the Equator Network under the Creative Commons Attribution 3.0 Unported (CC BY 3.0) license.

1. Skrivankova VW, Richmond RC, Woolf BAR, Yarmolinsky J, Davies NM, Swanson SA, et al. Strengthening the Reporting of Observational Studies in Epidemiology using Mendelian Randomization (STROBE-MR) Statement. JAMA. 2021;under review.

2. Skrivankova VW, Richmond RC, Woolf BAR, Davies NM, Swanson SA, VanderWeele TJ, et al. Strengthening the Reporting of Observational Studies in Epidemiology using Mendelian Randomisation (STROBE-MR): Explanation and Elaboration. BMJ. 2021;375:n2233.
